# Safety, pharmacokinetics, and immunological activities of multiple intravenous or subcutaneous doses of an anti-HIV monoclonal antibody, VRC01, administered to HIV-uninfected adults: Results of a phase 1 randomized trial

**DOI:** 10.1371/journal.pmed.1002435

**Published:** 2017-11-14

**Authors:** Kenneth H. Mayer, Kelly E. Seaton, Yunda Huang, Nicole Grunenberg, Abby Isaacs, Mary Allen, Julie E. Ledgerwood, Ian Frank, Magdalena E. Sobieszczyk, Lindsey R. Baden, Benigno Rodriguez, Hong Van Tieu, Georgia D. Tomaras, Aaron Deal, Derrick Goodman, Robert T. Bailer, Guido Ferrari, Ryan Jensen, John Hural, Barney S. Graham, John R. Mascola, Lawrence Corey, David C. Montefiori, on behalf of the HVTN 104 Protocol Team, and the NIAID HIV Vaccine Trials Network

**Affiliations:** 1 Fenway Health, Beth Israel Deaconess Medical Center, Harvard Medical School, Boston, Massachusetts, United States of America; 2 Duke Human Vaccine Institute, Duke University Medical Center, Durham, North Carolina, United States of America; 3 Vaccine and Infectious Disease Division, Fred Hutchinson Cancer Research Center, Seattle, Washington, United States of America; 4 Division of AIDS, NIAID, Bethesda, Maryland, United States of America; 5 Vaccine Research Center, NIAID, NIH, Bethesda, Maryland, United States of America; 6 Perelman School of Medicine, University of Pennsylvania, Philadelphia, Pennsylvania, United States of America; 7 College of Physicians and Surgeons, Columbia University Medical Center, New York, New York, United States of America; 8 Brigham and Women’s Hospital, Harvard Medical School, Boston, Massachusetts, United States of America; 9 Case Western University, Cleveland, Ohio, United States of America; 10 New York Blood Center, New York, New York, United States of America; 11 Department of Surgery, Duke University Medical Center, Durham, North Carolina, United States of America; Desmond Tutu HIV Centre, SOUTH AFRICA

## Abstract

**Background:**

VRC01 is an HIV-1 CD4 binding site broadly neutralizing antibody (bnAb) that is active against a broad range of HIV-1 primary isolates in vitro and protects against simian-human immunodeficiency virus (SHIV) when delivered parenterally to nonhuman primates. It has been shown to be safe and well tolerated after short-term administration in humans; however, its clinical and functional activity after longer-term administration has not been previously assessed.

**Methods and findings:**

HIV Vaccine Trials Network (HVTN) 104 was designed to evaluate the safety and tolerability of multiple doses of VRC01 administered either subcutaneously or by intravenous (IV) infusion and to assess the pharmacokinetics and in vitro immunologic activity of the different dosing regimens. Additionally, this study aimed to assess the effect that the human body has on the functional activities of VRC01 as measured by several in vitro assays. Eighty-eight healthy, HIV-uninfected, low-risk participants were enrolled in 6 United States clinical research sites affiliated with the HVTN between September 9, 2014, and July 15, 2015. The median age of enrollees was 27 years (range, 18–50); 52% were White (non-Hispanic), 25% identified as Black (non-Hispanic), 11% were Hispanic, and 11% were non-Hispanic people of diverse origins. Participants were randomized to receive the following: a 40 mg/kg IV VRC01 loading dose followed by five 20 mg/kg IV VRC01 doses every 4 weeks (treatment group 1 [T1], *n* = 20); eleven 5 mg/kg subcutaneous (SC) VRC01 (treatment group 3 [T3], *n* = 20); placebo (placebo group 3 [P3], *n* = 4) doses every 2 weeks; or three 40 mg/kg IV VRC01 doses every 8 weeks (treatment group 2 [T2], *n* = 20). Treatment groups T4 and T5 (*n* = 12 each) received three 10 or 30 mg/kg IV VRC01 doses every 8 weeks, respectively. Participants were followed for 32 weeks after their first VRC01 administration and received a total of 249 IV infusions and 208 SC injections, with no serious adverse events, dose-limiting toxicities, nor evidence for anti-VRC01 antibodies observed. Serum VRC01 levels were detected through 12 weeks after final administration in all participants who received all scheduled doses. Mean peak serum VRC01 levels of 1,177 μg/ml (95% CI: 1,033, 1,340) and 420 μg/ml (95% CI: 356, 494) were achieved 1 hour after the IV infusion series of 30 mg/kg and 10 mg/kg doses, respectively. Mean trough levels at week 24 in the IV infusion series of 30 mg/kg and 10 mg/kg doses, respectively, were 16 μg/ml (95% CI: 10, 27) and 6 μg/ml (95% CI: 5, 9) levels, which neutralize a majority of circulating strains in vitro (50% inhibitory concentration [IC50] > 5 μg/ml). Post-infusion/injection serum VRC01 retained expected functional activity (virus neutralization, antibody-dependent cellular cytotoxicity, phagocytosis, and virion capture). The limitations of this study include the relatively small sample size of each VRC01 administration regimen and missing data from participants who were unable to complete all study visits.

**Conclusions:**

VRC01 administered as either an IV infusion (10–40 mg/kg) given monthly or bimonthly, or as an SC injection (5 mg/kg) every 2 weeks, was found to be safe and well tolerated. In addition to maintaining drug concentrations consistent with neutralization of the majority of tested HIV strains, VRC01 concentrations from participants’ sera were found to avidly capture HIV virions and to mediate antibody-dependent cellular phagocytosis, suggesting a range of anti-HIV immunological activities, warranting further clinical trials.

**Trial registration:**

Clinical Trials Registration: NCT02165267

## Abstract 2

In a phase 1 trial, Kenneth Mayer and colleagues investigate the safety and tolerability of VRC01, a broadly neutralizing anti-HIV antibody.

## Author summary

**Why was this study done?:**

- Despite improvements in HIV treatment and prevention, there were close to 2 million new infections worldwide in 2016. There remains hope that the right preventive tool or combination of tools will eliminate new HIV infections.
- Immunotherapy and vaccines have been responsible for eliminating several major viral diseases, such as smallpox, and potentially very powerful HIV preventive tools are under development.
- Researchers have discovered that some antibodies can effectively neutralize many strains of HIV and protect nonhuman animals from getting infected; these are called broadly neutralizing antibodies (bnAbs).
- This study was designed to evaluate the safety, pharmacological profile, and immune functions of one such antibody administered either subcutaneously or intravenously as a foundation for future efficacy trials of this and other HIV-1 bnAbs.

**What did the research do and find?:**

- VRC01, an anti-HIV antibody, is safe and well tolerated when administered multiple times either subcutaneously or intravenously.
- The drug levels achieved with the different dosing regimens produced antibody levels that lasted many weeks at concentrations that could inhibit HIV when tested in vitro.
- The antibody in serum after administration showed evidence of a number of immune functions that are known to inhibit HIV transmission and replication.

**What do these findings mean?:**

- The safety and tolerability of VRC01, administered multiple times either subcutaneously or intravenously, support moving forward with larger trials.
- The concentrations achieved when dosed at 10 mg/kg or 30 mg/kg every 8 weeks produced drug levels that are predicted to protect against the acquisition of HIV infection.
- These data helped inform the development of 2 large efficacy trials of VRC01 in high-risk people in the US, South America, and southern Africa that will establish whether intravenous administration is protective against HIV transmission (www.ampstudy.org, ampstudy.org.za). Importantly, this work establishes the framework for subsequent analyses of effective levels of VRC01 or other bnAbs as well as the neutralization titers necessary to prevent HIV, which future HIV vaccines must aim to induce.

## Introduction

Antiretroviral (ARV) therapy for HIV-1 treatment and prevention is increasingly available globally; nonetheless, about 2 million new HIV-1 infections occurred in 2016 [1]. Although ARV treatment can decrease HIV transmission [2] and the use of pre-exposure prophylaxis can decrease acquisition [3], the logistics and costs of scaling up these approaches remain daunting and expensive. Given the magnitude of ongoing HIV spread, the continued development of other novel, safe, and effective preventive strategies is urgently needed [1,4].

Vaccines that induce neutralizing antibodies are a high priority for HIV prevention but, to be successful, the antibodies will need to overcome the extraordinary genetic plasticity and antigenic variability of the virus [5–7]. A number of broadly neutralizing antibodies (bnAbs) have been isolated from HIV-infected individuals, and detailed information about their epitopes and other features is providing valuable insights for the design of immunogens that aim to induce similar antibodies by vaccination [8–12]. At least 5 sites on the trimeric HIV-1 envelope glycoprotein (Env) spike are susceptible to bnAbs [13–15]. The CD4 binding site (CD4bs) of glycoprotein 120 (gp120) is of particular interest because it is targeted by some of the most potent and broadly cross-reactive bnAbs described to date. One anti-CD4bs bnAb (N6) neutralized up to 98% of a large multi-subtype panel of HIV-1 Env-pseudotyped viruses [16].

The Vaccine Research Center (VRC) of the National Institutes of Health (NIH) has developed VRC01, a bnAb that binds the HIV-1 CD4bs [17–19] from an HIV-1-infected long-term non-progressor [20]. In vitro, VRC01 neutralizes the majority of a panel of 190 HIV-1 Env-pseudotyped viruses across multiple genetic subtypes of the virus [18] and protects against simian-human immunodeficiency virus (SHIV) infection upon passive administration in nonhuman primates (NHPs) [21–24]. The ontogeny, structure, and mode of binding of VRC01 have been well characterized [17,19,20,25–28].

The efficacy of passive transfer of monoclonal antibodies (mAbs) to prevent the establishment of serious viral and other infections has been previously demonstrated. Monthly injections of palivizumab, a mAb that neutralizes entry of respiratory syncytial virus (RSV), were found to be safe, well tolerated, and effective in protecting infants with underlying pulmonary disease from developing clinically significant infection [29]. The use of mAbs has also been found to be safe and well tolerated when used to treat *Clostridium difficile* colitis [30] and to prevent hepatitis C rebound in patients who received liver transplants [31,32].

VRC01 is not autoreactive or polyreactive and lacks antiphospholipid antibody activity [17]. It has been proven to be safe and well tolerated in single- and 2-dose intravenous (IV) and subcutaneous (SC) regimens up to 40 mg/kg in both HIV-uninfected and HIV-infected individuals, with no anti-VRC01 antibodies detected after VRC01 administration [33,34]. The bnAb demonstrated expected pharmacokinetics of similar immunoglobulin G1(IgG1) mAbs, with a terminal half-life of approximately 15 days [33,35]. Eight HIV-infected, treatment-naïve participants received single IV administrations of VRC01 with excellent tolerability and 1.1 to 1.8 log10 reductions in HIV plasma RNA concentrations [34]. Notably, 2 participants with plasma HIV RNA levels <1,000 copies/ml demonstrated virus suppression to undetectable levels for over 20 days without the use of ARVs after a single administration of VRC01.

HVTN 104 is an HIV Vaccine Trials Network (HVTN) phase 1 trial designed to evaluate and provide detailed characterization of the safety, tolerability, and pharmacokinetics of repeated doses of VRC01 administered via different dosing schedules, frequencies, and delivery routes in preparation for evaluation of long-term HIV-1 immunoprophylaxis. The current study also assessed whether VRC01 in participants’ sera maintained expected immunological activities, including its potency and breadth of neutralization against a panel of tier 2 virus reference strains, antibody-dependent cellular cytotoxicity (ADCC), phagocytic activity, and virion capture.

## Methods

### Trial design

**Fig 1:**
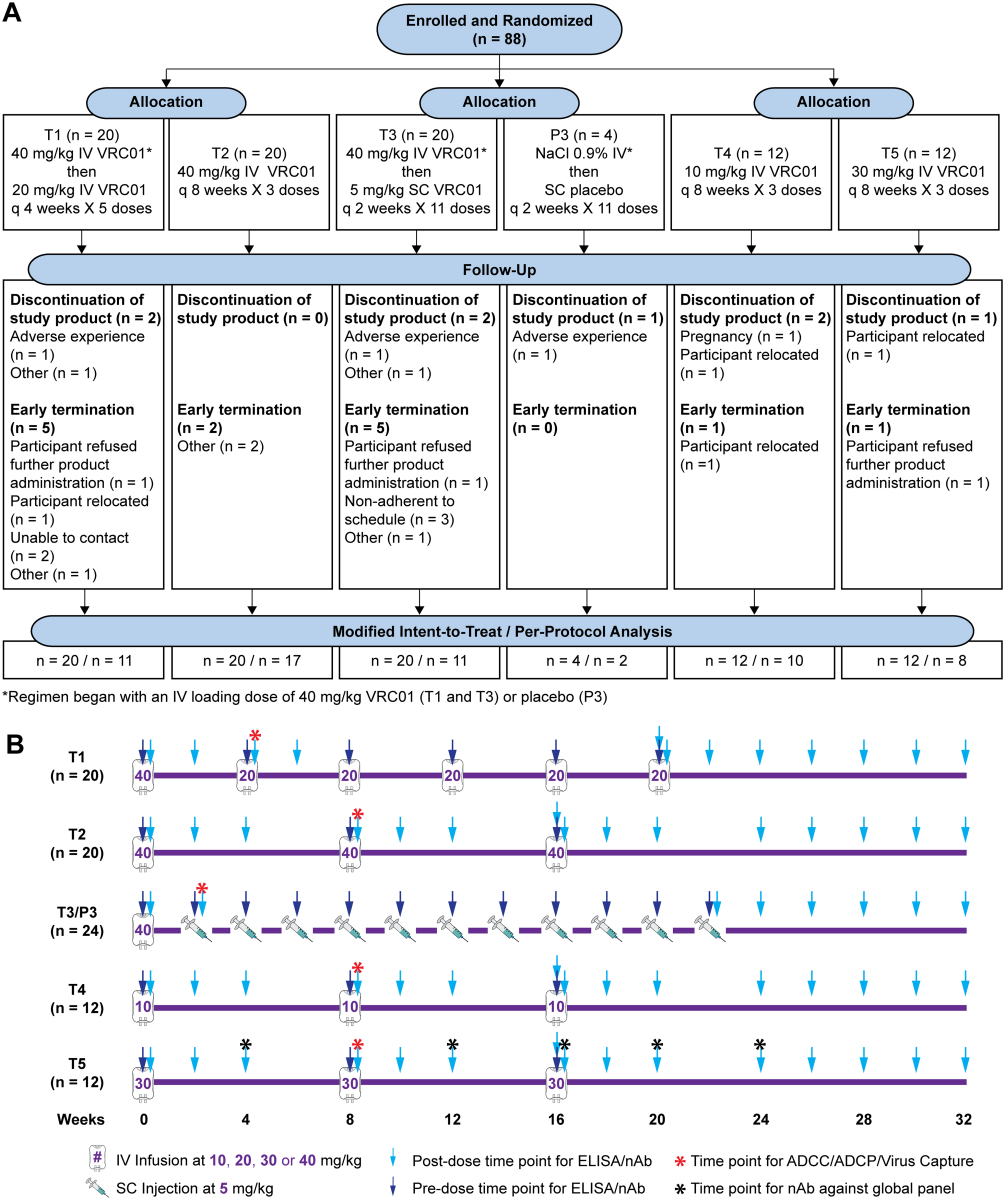
HVTN 104 CONSORT flow diagram (A) and specimen collection schedule (B). T1: 20 mg/kg IV q 4 weeks with 40 mg/kg IV loading; T2: 40 mg/kg IV q 8 weeks; T3 (P3): 5 mg/kg SC q 2 weeks with 40 mg/kg IV loading; T4: 10 mg/kg IV q 8 weeks; T5: 30 mg/kg IV q 8 weeks. ADCC, antibody-dependent cellular cytotoxicity; ADCP, antibody-dependent cellular phagocytosis; CONSORT, Consolidated Standards of Reporting Trials; HVTN, HIV Vaccine Trials Network; IV, intravenous; P3, placebo group 3; q, quodque; SC, subcutaneous; T1, treatment group 1; T2, treatment group 2; T3, treatment group 3; T4, treatment group 4; T5, treatment group 5.

HIV-uninfected, low-risk participants (Clinicaltrials.gov NCT02165267) were randomized in a 1:1 ratio to receive open-label IV VRC01 in treatment groups T1 or T2 (Fig 1A, and S1 CONSORT Checklist). Participants in treatment group T1 (*n* = 20) received VRC01 at 40 mg/kg IV at week 0 and then 20 mg/kg at weeks 4, 8, 12, 16, and 20; participants in treatment group T2 (*n* = 20) received VRC01 at 40 mg/kg IV at weeks 0, 8, and 16. Participants who consented to a more intensive visit schedule were assigned to a blinded third group in a 5:1 ratio to receive SC VRC01 (treatment group T3) or placebo (group 3, P3) injections. Participants in treatment/placebo group T3/P3 received SC injections of VRC01 (*n* = 20) every 2 weeks at 5 mg/kg or placebo (*n* = 4), following an IV loading dose of VRC01 at 40 mg/kg or IV placebo. Two additional dosing regimens were subsequently added via a protocol modification to evaluate potential candidate regimens for future efficacy trial testing. Participants in these latter 2 groups were randomized in a 1:1 ratio to open-label treatment groups T4 or T5 (*n* = 12 each) to receive IV infusions of VRC01 at 10 mg/kg and 30 mg/kg, respectively, at weeks 0, 8, and 16. Blood samples were collected before product administration and at 3 days, 2, 4 (except treatment/placebo group T3/P3), and 8 (treatment groups T2, T4, and T5 only) weeks after each infusion (or injection) as well as at 1 hour post last infusion, and at 10–16 weeks post last infusion/injection (Fig 1B). Specifically, trough measurements were taken at 4 weeks (for treatment group T1) or 8 weeks (for treatment groups T2, T4, and T5) following infusions and 2 weeks following SC injections (treatment/placebo group T3/P3), immediately prior to the next infusion or injection; peak measurements were taken at 1 hour post-infusion for IV groups (treatment groups T1, T2, T4, and T5) and 3 days after injection for the SC group (treatment/placebo group T3/P3).

### Participants

A total of 88 participants were each enrolled into 1 of the 5 treatment groups in HVTN 104. Eligibility criteria included being low risk, HIV uninfected, aged 18 to 50 years; weighing between 53 kg and 115 kg; being deemed healthy based upon medical history, physical examination, and laboratory tests; having access to a participating HVTN clinical research site for the planned duration of the study; and demonstrating understanding of the study. Volunteers who were born female and had reproductive potential were screened for pregnancy and excluded if pregnant, and were required to agree to use effective contraception for the duration of the study. The Investigational New Drug (IND) application was sponsored by the National Institute of Allergy and Infectious Diseases (NIAID) Division of AIDS (DAIDS). The study was conducted in compliance with the principles of GCP (ICH E6) and according to DAIDS and HVTN policies and procedures. Clinical research site staff completed enrollment procedures and obtained written informed consent from all participants. The study was approved by the local institutional review boards at all participating sites: Brigham, Partners Human Research Committee; Fenway, Fenway Health IRB; Cleveland, University Hospitals, Cleveland Medical Center IRB; New York Blood Center, New York Blood Center IRB; College of Physicians & Surgeons, Columbia University IRB; Philadelphia, University of Pennsylvania IRB (Clinicaltrials.gov NCT02165267).

### Interventions

VRC01 (VRC-HIVMAB060-00-AB) was formulated at a concentration of 100 (±10) mg/ml suspended in formulation buffer (25 mM sodium citrate, 50 mM sodium chloride, and 150 mM L-arginine hydrochloride at pH 5.8). The placebo for SC administration was VRC-PLAMAB068-00-AB, a sterile buffered aqueous solution of 25 mM sodium citrate, 50 mM sodium chloride, 150 mM L-arginine hydrochloride, 10% dextran 40 (w/w), and 0.005% polysorbate 80 (w/w) at pH 5.8. The purified product vials and the placebo vials were filled and labeled at the VRC Vaccine Pilot Plant operated by Leidos Biomedical Research, Inc. (Frederick, MD). The placebo was matched for color and viscosity to mimic the interventional product to avoid unintentional unblinding. The placebo for IV infusions was sodium chloride for injection 0.9%, USP, obtained locally by the sites. IV infusions were administered via an IV pump over 60 minutes for the first administration, then over at least 30 minutes for subsequent infusions. For each SC administration, up to 4 injections of 2 mL of the study product could be administered, with abdominal sites being used most frequently.

### Outcomes

The prespecified primary clinical objectives of the study were to evaluate the safety and tolerability of VRC01 administered intravenously and subcutaneously at multiple time points. Primary endpoints were local and systemic reactogenicity signs and symptoms, laboratory measures of safety, adverse events (AEs) and serious AEs (SAEs), early discontinuation of infusions and reason(s) for discontinuation, and early study termination. The primary laboratory objective was to evaluate the serum levels of VRC01 at week 24, following IV and SC administration in 3 different regimens, with the primary end points being serum concentrations of VRC01 in each group at week 24. Secondary objectives included the determination of serum levels of VRC01 over time and whether anti-idiotypic antibodies could be detected. Exploratory objectives included the evaluation of in vitro functional humoral activities of VRC01 in serum and mucosal compartments. Specimens from participants were also tested for neutralizing activity against a panel of circulating and laboratory strains of HIV. Assessment of the binding of VRC01 to multiple Env proteins as well as the determination of VRC01 concentrations and functional activity in genital, rectal, and oral secretions are still underway and will be reported in a future manuscript.

### Sample size

The sample size of the study was determined to provide reasonable precision in the estimation of safety event rates and the mean trough drug concentration after the administration series for each active treatment arm. Specifically, for an active arm of *n* = 12, there is a 90% chance of observing at least 1 safety event if the true rate of such an event is 17.5% or more, and there is a 90% chance of observing no events if the true rate is 0.88% or less. For each active arm of *n* = 20, there is a 90% chance of observing at least 1 event if the true rate of such an event is 10.9% or more, and there is a 90% chance of observing no events if the true rate is 0.52% or less. In addition, the chosen sample sizes provide reasonable precision for the estimation of serum concentrations, assuming a normal distribution for the log-transformed trough levels. Further discussion of the statistical considerations involved in the selection of sample sizes is provided in the full protocol (S1 Protocol).

### Randomization

The randomization sequence was obtained by computer-generated random numbers and provided to each HVTN clinical research site through the Statistical Data Monitoring Center (SDMC) of the HVTN via a web-based randomization system. The randomization was conducted in blocks to ensure balance between T1 and T2, between T4 and T5, and within T3/P3 treatment groups. At each institution, the pharmacist with primary responsibility for dispensing study products was charged with maintaining security of the treatment assignments.

### Blinding

Participants and site staff were unblinded as to participant treatment assignments among treatment groups T1, T2, T4, and T5, but blinded, except for site pharmacists, as to treatment assignment (active versus placebo) for treatment/placebo group T3/P3. Study product assignments were accessible to study pharmacists, contract monitors, and SDMC staff, who are required to know this information in order to ensure proper trial conduct.

### Clinical assessments

Participants were monitored for type 1 hypersensitivity reactions, cytokine release syndrome (CRS), and serum sickness during and post infusion. Reactogenicity assessments were performed at baseline (prior to each study product administration) and following each IV infusion/SC injection at the early assessment time point (within 25–120 minutes) and daily for the subsequent 3 days. Participants were instructed to record symptoms using a postproduct symptom log and to contact the site daily during the assessment period. Local injection site reactions of pain, tenderness, erythema, and induration and systemic symptoms of malaise and/or fatigue, myalgia, headache, nausea, vomiting, chills, arthralgia, and body temperature were assessed. Following the first study infusion, safety monitoring included the performance of abbreviated physical exams and the assessment of AEs and concomitant medications at each study visit. Clinical laboratory testing included the collection of complete blood count and chemistry panel at 2 weeks (in all groups), then monthly until month 6 (in treatment and placebo groups T1 and T3/P3) and at month 2, 3, 4, and 6 (treatment groups T2, T4, and T5) and urine dipstick/urinalyses at 2 weeks and 3 and 6 months in all groups. Pregnancy testing was performed in female participants and results confirmed as negative prior to each study product administration. AEs were reported for the duration of the study. Safety reports were monitored daily by the HVTN Core for events meeting safety pause criteria and prompt Protocol Safety Review Team (PSRT) AE review rules. Weekly safety data reviews by the PSRT led to teleconferences when indicated, and the independent HVTN Safety Monitoring Board (SMB) reviewed unblinded safety data thrice yearly. Research sites reported clinical safety data to the Statistical Center for HIV/AIDS Research & Prevention (SCHARP) at the Fred Hutchinson Cancer Research Center. The number and percentage of participants experiencing each type of reactogenicity sign or symptom was tabulated by maximum severity and treatment arm and the percentages were displayed graphically by arm. Severity of reactogenicity and AEs was graded using the DAIDS US NIH Table for Grading the Severity of Adult and Pediatric Adverse Events, Version 1.0, December, 2004; clarification August, 2009 [36].

### Pharmacokinetic analysis

VRC01 concentration in participants’ sera was quantified using the murine anti-VRC01 mAb 5C9 as previously described [33]. Briefly, 5C9 was coated onto Immulon-4HXB microtiter plates overnight at 4°C. Plates were washed and then blocked with 10% fetal bovine serum in phosphate-buffered saline. Duplicate serial 3-fold dilutions covering the range of 1:100–1:24,300 of the test sample were added and incubated for 2 hours at 37°C, followed by the addition of horseradish peroxidase-labeled goat antihuman antibody and TMB substrate. Color development was stopped by the addition of sulfuric acid, and plates were read within 30 minutes at 450 nm via the Molecular Devices Paradigm plate reader. Four-parameter logistic curve regression of a standard curve of VRC01 covering the range from 0.98 to 1,000 ng/ml was utilized to quantitate sample concentrations based upon the average of sample dilutions within the range of the assay. Concentration values below the limit of quantification (1.10 μg/ml) were replaced by 0.55 μg/ml in all calculations.

### Neutralization assay

Neutralizing activity was measured against HIV Env-pseudotyped viruses as a function of reductions in Tat-regulated luciferase (Luc) reporter gene expression in TZM-bl cells as described [37,38]. Assays for measurements of serum VRC01 concentration were performed with 3 Env-pseudotyped viruses that exhibit either a highly sensitive tier 1A phenotype (clade B: MN.3, clade C: MW965.26) or a moderately sensitive tier 2 phenotype that is typical of most circulating strains (clade B: PVO.4). As these 3 strains are highly sensitive to neutralization by VRC01, they were selected for optimal sensitivity for the assays quantifying levels of the antibody. The 50% inhibitory concentration (IC50) and 80% inhibitory concentration (IC80) of the clinical lot of VRC01 were determined against each virus. The stock concentration of VRC01 used in this analysis (0.34 mg/ml) was determined by the same ELISA used for pharmacokinetic (PK) measurements. The concentration of VRC01 in serum samples was calculated by multiplying the 50% infectious dose (ID50) (or 80% infectious dose [ID80]) neutralization titer (as a dilution factor) of the serum sample against each isolate by the IC50 (or IC80) of the clinical lot VRC01 (in μg/ml) against the corresponding isolate, in which the IC50 and IC80 of the clinical lot of VRC01 was the geometric mean value obtained in repeated assays. Additional assays were performed with a global panel of 11 tier 2 Env-pseudotyped reference strains [39] to assess the magnitude and breadth of serum neutralizing activity in greater detail. These latter results were expressed as the serum dilution that resulted in either a 50% or 80% reduction in infectivity (ID50 and ID80 neutralization titers). Assays with this global panel of tier 2 viruses were performed for 6 participants from treatment group T5 (selected based upon sample availability for all time points) at 4 weeks following each infusion and at 1 hour and 8 weeks following the last infusion.

### ADCC assay

ADCC activity against CEM.NKRCCR5 cells infected with the subtype C HIV-1-infected CH505 transmitted/founder infectious molecular clone (IMC) (provided by Dr. Ochsenbauer, University of Alabama–Birmingham) was measured in 96-well plates as a function of reductions in Renilla Luc reporter gene expression as previously described [40,41]. One positive control in duplicate and 1 standardized negative control in duplicate were used per plate. The readout was reduction in Relative Luminescence Units (RLU), referred to as percentage specific killing. For each sample, percent specific killing was measured in 2 wells at a dilution of 1:25. The CD4bs bnAbs 3BNC117 and CH31 (both generated by Duke Human Vaccine Institute, Protein Production Facility, Dr. Haynes) and the clinical product VRC01-13-123 were also tested starting at the concentration of 50 μg/ml. The anti-RSV Synagis mAb (MedImmune, LLC, Gaithersburg, MD) was utilized as a negative control. The analyses of plasma ADCC responses examined peak percent specific killing (%SK), defined as the maximum %SK across the 6 dilution levels (“peak %SK”) and averaged over the 2 replicates. A positive response is defined as an increase in peak percentage over baseline greater than or equal to 10%.

### Antibody-dependent cellular phagocytosis assay

Antibody-dependent cellular phagocytosis (ADCP) was measured as previously described [42,43], with the following modifications. Briefly, biotinylated ConS gp140CF antigen (Duke Human Vaccine Institute, Protein Production Facility, Dr. Haynes) [44] was conjugated to neutravidin fluorescent microspheres and 9×10^5^ microspheres equivalent were mixed with 10 μl of 25 μg/ml mAbs or 25 μg/ml purified plasma immunoglobulin G (IgG); VRC01 represented approximately 1% of the total purified plasma IgG when quantified by Binding Antibody Multiplex Assay (BAMA). After incubation, 50,000 (THP-1) or 500,000 (monocyte) cells were added to each well and spinoculated. Cells were fixed and phagocytosis was analyzed by flow cytometry from at least 2,000 cell events per sample. A phagocytic score was determined by the ratio of samples (percent bead positive multiplied by the MFI bead positive values for samples) to the no-bead antibody controls. Samples were considered positive if the average phagocytic score was(1) greater than the 95th percentile of the score for all baseline visits tested (visit 2) and (2) the follow-up visit response value was ≥3 times the baseline visit response value. Data presented are representative of 2 independent experiments. Positive controls included the VRC01 clinical lot, another VRC01-class CD4bs bnAb, VRC-CH31 [27], and the Env glycan-specific bnAb 2G12 [45] and negative controls included palivizumab (RSV fusion protein-specific mAb) [46] and CH65 (H1N1-specific mAb) [47].

### Infectious virion capture assay

Infectious virion capture assay (IVCA) was measured as previously described [48,49]. Briefly, 25 μg of total purified IgG was mixed with BaL.LucR stock at a final concentration of 20 ug/mL to form antibody–virion immune complexes (ICs), which were passed through a protein G column. The infectivity of the flow-through was measured by a TZM-bl infectivity assay. The percentage of captured infectious virions (iVirion) was calculated as follows: iVirion = [(100—flow-through infectivity) / (virus no-Ab infectivity)] × 100%. VRC01 clinical lot, VRC-CH31 [27], and 2G12 [45] were used as positive controls. Negative controls included CH65 [47] and palivizumab [46] as well as a no-Ab antibody control. Samples were considered positive if the average IVCA was(1) greater than the 95th percentile of the score for all baseline visits tested (visit 2) and (2) follow-up visit response value was ≥3 times the baseline visit response value.

### Antidrug antibody analysis

Anti-VRC01 antibodies were measured by using the Meso Scale Discovery (MSD) platform via electro-chemiluminescence as described [33]. Specifically, serial dilutions of serum samples were incubated with optimized concentrations of biotinylated VRC01 and SULFO-TAG labeled VRC01 at 37°C for 2 hours. Samples were transferred to a previously blocked Streptavidin-coated MSD plate and incubated for 3 hours at room temperature on a plate shaker. Plates were washed on an automated plate washer, read-buffer was added, and samples were read with the MSD-2400 plate reader. Mean signals from replicate wells were evaluated and the endpoint dilution was defined as the greatest sample dilution with a response above the positivity threshold for the assay. The positivity threshold is the lower limit of the linear range for VRC01 in the assay. Serial dilutions of the VRC01-specific monoclonal antibody 5C9 were utilized to establish the linear range of 5C9 (0.01221 to 3.5 μg/mL). Samples were initially tested in quadruplicate wells with an initial dilution of 1:4. Any samples positive at 1:4 were further tested with 8 serial 4-fold dilutions (covering a dilution range of 4 to 65,536) and reported as one of the following: <4; 4; 16; 64; 256; 1,024; 4,096; 16,384; or >65,536.

Antidrug antibody (ADA) assays were performed on randomly selected baseline samples from 10 VRC01 participants and on select post-infusion/injection time points from all VRC01 participants. The post-IV infusion time points were week 24 (4 weeks after the final IV infusion in T1; 8 weeks after the final IV infusion in T2, T4, and T5; 2 weeks after the final injection in T3) and week 32 (12 weeks after the final IV infusion in T1; 16 weeks after the final IV infusion in T2, T4, and T5; and 10 weeks after the final SC injection in T3).

### Statistical methods

The modified intent-to-treat (MITT) cohort included all enrolled HIV-uninfected participants receiving the first infusion and was analyzed according to participants’ assigned treatment. The per-protocol (PP) cohort included participants who received all assigned treatments within the expected visit windows. Safety evaluation was done in the MITT cohort. The number and percentage of participants experiencing each AE or reactogenicity symptom and sign were analyzed by severity and treatment group. VRC01 levels were evaluated in both the MITT and PP cohorts. Geometric means and associated 95% confidence intervals of VRC01 levels were based on a t distribution. Functional activities and their correlations with VRC01 levels were evaluated in the PP cohort.

Individual-level non-compartmental pharmacokinetics analysis was performed based on the ELISA-based serum concentration-time data in the PP cohort. Specifically, VRC01 accumulation was computed based on the ratio of trough drug level measurements and the ratio of the area under the curve (AUC) within the infusion interval for each participant, after the third and second infusions, as compared to the first in groups T4 and T5. The estimates were crude due to the relatively sparse time points. The trough levels were either observed or predicted as the estimated mean concentration at 8 weeks post infusion based on the fitted individual-level log-linear line through the observed concentrations at day 28 and day 56 after the first infusion, at day 84 and day 112 after the second infusion, and at day 140 and day 168 after the third and last infusion. AUC was calculated using the linear trapezoidal method. The expected visit dates were used in these calculations. Lastly, the terminal elimination rate and half-life were calculated based on the log-linear portion (28 to 84 days after the last infusion) of the time-concentration curve for each participant in groups T4 and T5. The terminal rate (slope) was determined by fitting a linear regression line to the log-transformed concentrations over time. The half-life was estimated as ln2 (= 0.693) divided by the terminal slope. Analyses were performed using SAS and R 3.1.1 [50].

## Results

### Safety and adherence

Eighty-eight healthy, HIV-uninfected, low-risk participants were enrolled in 6 US clinical research sites affiliated with the HVTN between September 9, 2014, and July 15, 2015. Participants were followed for 32 weeks after the initial study infusion, and all participants completed the trial by February 2016. The median age of enrollees was 27 years (range, 18–50); 52% were White (non-Hispanic), 25% identified as Black (non-Hispanic), 11% were Hispanic, and 11% were non-Hispanic people of diverse origins (Table 1). Volunteers received the following: T1, single IV infusion of 40 mg/kg VRC01 followed by 5 IV infusions of 20 mg/kg VRC01 every 4 weeks; T2, 3 IV infusions of 40 mg/kg VRC01 every 8 weeks; T3 (P3), single IV infusion of 40 mg/kg VRC01 (or placebo) followed by 11 SC injections of 5 mg/kg VRC01 (or placebo) every 2 weeks; T4, 3 IV infusions of 10 mg/kg VRC01 every 8 weeks; and T5, 3 IV infusions of 30 mg/kg VRC01 every 8 weeks (Fig 1).

**Table 1:** Demographics and study product administration frequencies by group in the MITT cohort.

	T1 (*N* = 20)	T2 (*N* = 20)	T4 (*N* = 12)	T5 (*N* = 12)	T3 (*N* = 20)	P3 (*N* = 4)	Total (*N* = 88)
**Sex at birth**							
**Male**	11 (55%)	9 (45%)	6 (50%)	6 (50%)	10 (50%)	2 (50%)	44 (50%)
**Female**	9 (45%)	11 (55%)	6 (50%)	6 (50%)	10 (50%)	2 (50%)	44 (50%)
**Race/ethnicity**							
**White–non-Hispanic**	12 (60%)	12 (60%)	7 (58%)	5 (42%)	8 (40%)	2 (50%)	46 (52%)
**Black–non-Hispanic**	5 (25%)	6 (30%)	2 (17%)	3 (25%)	5 (25%)	1 (25%)	22 (25%)
**Hispanic**	1 (5%)	1 (5%)	3 (25%)	3 (25%)	2 (10%)	0	10 (11%)
**All other**^A^	2 (10%)	1 (5%)	0	1 (8%)	5 (25%)	1 (25%)	10 (11%)
**Age (years)**							
**Median**	29.5	27.5	26.5	29.0	23.5	42.0	27.0
**Range**	18–50	18–50	21–43	24–42	18–49	29–44	18–50
**SPA frequencies**							
**Day 0**	20(100%)	20(100%)	12 (100%)	12(100%)	20 (100%)	4(100%)	88 (100%)
**Day 14**	N/A	N/A	N/A	N/A	19 (95%)	4(100%)	23 (96%)
**Day 28**	19 (95%)	N/A	N/A	N/A	18 (90%)	3 (75%)	40 (91%)
**Day 42**	N/A	N/A	N/A	N/A	17 (85%)	3 (75%)	20 (83%)
**Day 56**	18 (90%)	19 (95%)	10 (83%)	9 (75%)	17 (85%)	3 (75%)	76 (86%)
**Day 70**	N/A	N/A	N/A	N/A	16 (80%)	3 (75%)	19 (79%)
**Day 84**	16 (80%)	N/A	N/A	N/A	14 (70%)	3 (75%)	33 (75%)
**Day 98**	N/A	N/A	N/A	N/A	14 (70%)	3 (75%)	17 (71%)
**Day 112**	15 (75%)	18 (90%)	10 (83%)	10 (83%)	16 (80%)	3 (75%)	72 (82%)
**Day 126**	N/A	N/A	N/A	N/A	17 (85%)	3 (75%)	20 (83%)
**Day 140**	17 (85%)	N/A	N/A	N/A	15 (75%)	3 (75%)	35 (80%)
**Day 154**	N/A	N/A	N/A	N/A	12 (60%)	2 (50%)	14 (58%)

^A^Includes Asian-Pacific Islander, Native American, those who selected other, and multiracial individuals.

**Abbreviations**: MITT, modified intent-to-treat; N/A, nonapplicable; SPA, study product administration.

**Fig 2:**
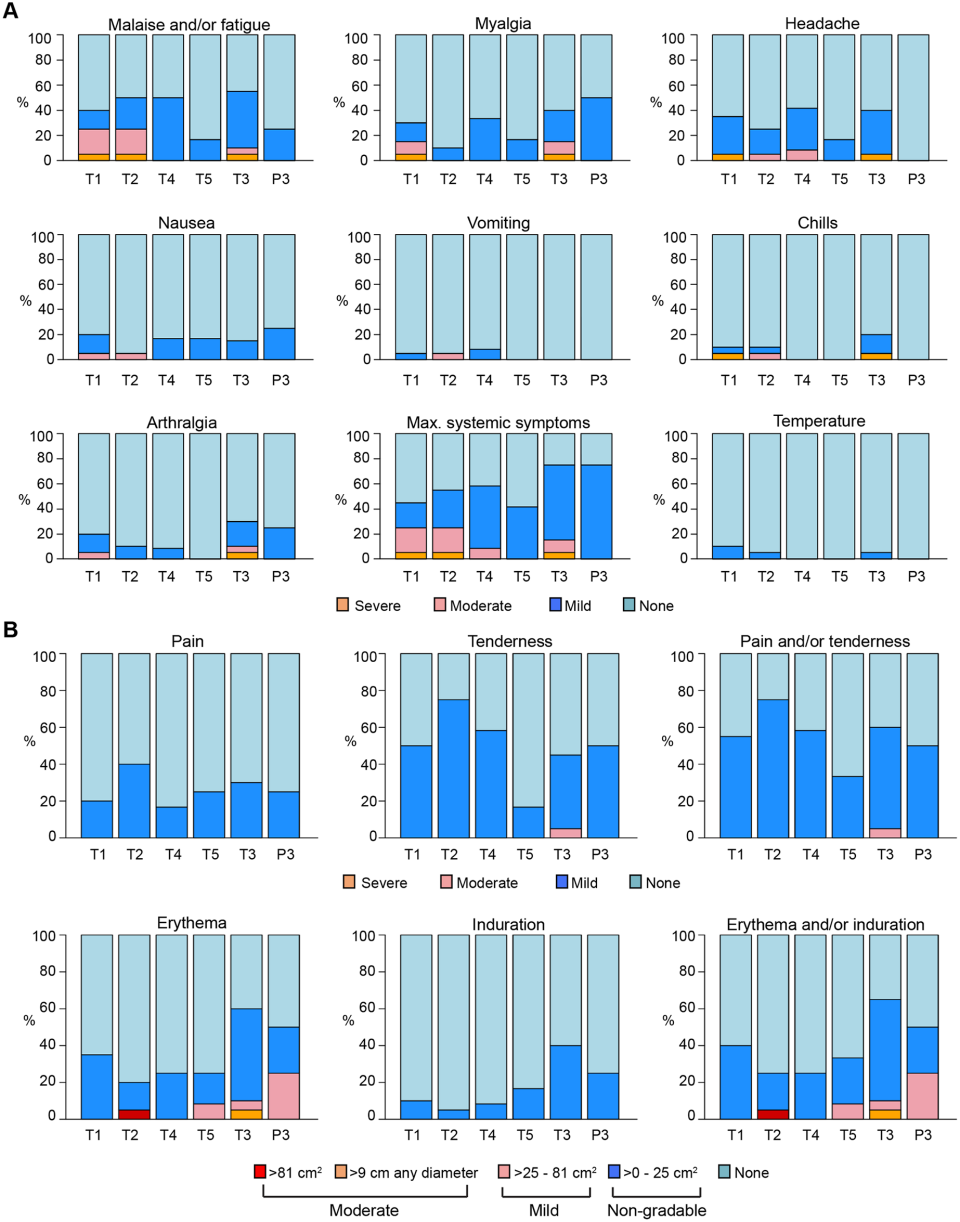
Frequency of maximum severity of systemic (A) and local (B) reactogenicity symptoms by group in the MITT cohort. T1: 20 mg/kg IV q 4 weeks with 40 mg/kg IV loading; T2: 40 mg/kg IV q 8 weeks; T3 (P3): 5 mg/kg SC q 2 weeks with 40 mg/kg IV loading; T4: 10 mg/kg IV q 8 weeks; T5: 30 mg/kg IV q 8 weeks. Grading per DAIDS Table for Grading the Severity of Adult and Pediatric Adverse Events, Version 1.0, December 2004; clarification August 2009 [36]. DAIDS, Division of AIDS; MITT, modified intent-to-treat; P3, placebo group 3; q, quodque; SC, subcutaneous; T1, treatment group 1; T2, treatment group 2; T3, treatment group 3; T4, treatment group 4; T5, treatment group 5.

In the MITT cohort of 88 participants, 249 IV infusions and 208 SC injections were administered and were generally well tolerated, with 28% (95% CI: 23%, 34%) of infusions and 14% (95% CI: 10%, 19%) of injections associated with mild pain or tenderness. Fifty-five percent (95% CI: 44%, 65%) of VRC01 and 50% (95% CI: 15%, 85%) of placebo recipients experienced mild pain and/or tenderness at the infusion or injection site sometime during the trial, 1 person (in T3) experienced moderate tenderness following one of the SC injections, and no participants reported severe pain and/or tenderness. Very few of the erythema/induration reactions that occurred met the NIH’s DAIDS criteria for mild reactions (>25–81 cm^2^); these included 2 participants following receipt of IV VRC01 (1 in T3 and 1 in T5) and 1 participant following IV placebo administration (P3). Only 2 met the criteria for moderate reactions (>9 cm in diameter or >81 cm^2^); these included 1 participant following IV VRC01 (T2) and 1 participant following an SC injection (T3) (Fig 2). For 76% of the infusions and injections administered in the trial, no systemic symptoms were reported. Fifty-six percent (95% CI: 45%, 66%) of VRC01 and 75% (95% CI: 30%, 95%) of placebo recipients experienced systemic reactogenicity symptoms following at least 1 study product administration. The symptoms were all graded as mild in the placebo recipients, while, of those VRC01 recipients who experienced any systemic symptoms, the maximum severity was mild in 70%, moderate in 23%, and severe in 6% (3 participants). In the 3 participants with severe reactogenicity symptoms, 2 had concurrent viral infections, while 1 reported severe malaise lasting 1 day, with no sequelae. The most commonly reported systemic symptoms were malaise/fatigue, headaches, or myalgias.

There were 235 AEs occurring in 70 participants (79.5%), with similar rates of occurrence across all treatment groups. Seventy-four percent of AEs were graded as mild, 22.5% moderate, and 3.4% severe. There were 9 AEs (3.8% of all AEs, 95% CI: 2%, 7%) occurring in 8 participants that were deemed product related by the investigators; all were mild and transient, and those occurring following VRC01 administration included elevations of hepatic transaminases (aspartate transaminase [AST] and alanine transaminase [ALT] elevation in 1 participant), elevated creatinine, neutropenia, localized injection site pruritus, diarrhea, generalized rash, and varicella zoster virus reactivation. An AE of chest tightness deemed related to study product occurred in a placebo recipient. No VRC01-related hypersensitivity reactions or CRS symptoms were observed during the study.

Study product was discontinued in 8 participants, including 2 participants who relocated from study sites, 1 participant who became pregnant, 1 who did not adhere to study visits, and 1 who was unable to be contacted. One participant developed a mild, generalized, pruritic, maculopapular rash 3 days after receiving the first SC injection of VRC01, which resolved less than 4 hours after applying inert lotion and was deemed related to the study product. Another participant had a brief syncopal episode several hours after receiving a VRC01 infusion, deemed not related to the study product. A third participant had mild, brief chest tightness following the first SC injection that resolved spontaneously; this was deemed related to the study product; however, this participant was determined after unblinding to be a placebo recipient.

Regarding adherence, 35 out of 44 (80%) participants in groups T2, T4, and T5 received all expected 8-weekly infusions, with 90% complete adherence in T2, 75% in T4, and 83% in T5. Eleven of 20 participants (55%) received all expected 4-weekly infusions in group T1, while 13 out of 24 (54%) participants received all biweekly injections in group T3/P3 (Table 1). Twenty-nine participants (33%) missed at least 1 infusion or injection during the study. The most commonly cited reasons for missed visits were travel, relocation, or loss to follow-up. The study product was not administered for 11 study visits, affecting 9 participants in the first 3 groups, due to the observation of frozen study product at the end of a thawing period of less than 1 hour, leading to revision of pharmacy instructions to request a thawing period of greater than 1 hour, without further abnormalities observed. The 59 participants (57 VRC01 recipients and 2 placebo recipients) who received all study product administrations comprise the PP cohort and, unless otherwise stated, were the focus of PK and immunological assessments.

### Pharmacokinetics

**Fig 3:**
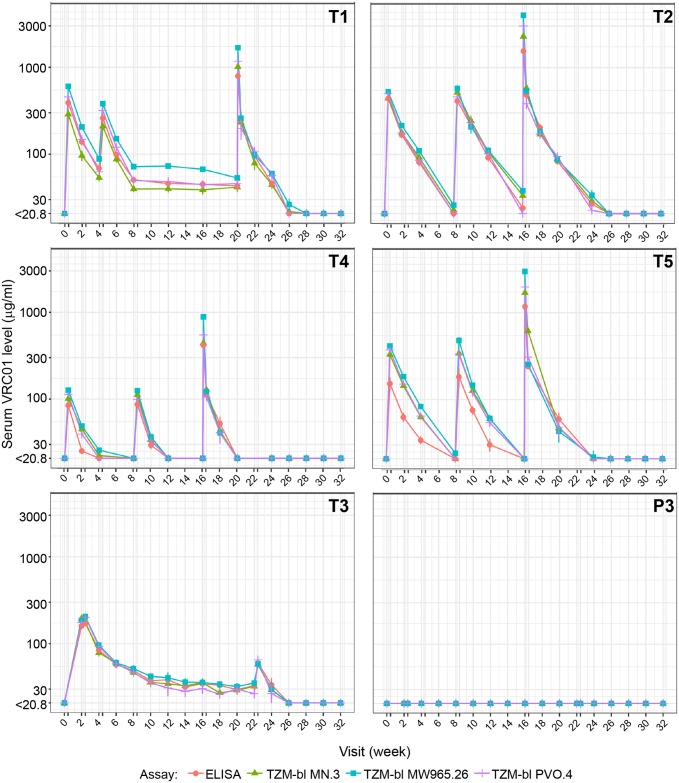
VRC01 serum concentrations over time in the PP cohort, measured by ELISA and TZM-bl assays. ELISA measured anti-idiotypic binding. TZM-bl measured neutralization against 3 Env-pseudotyped virus strains (MN.3, MW965.26, and PVO.4). Each dot (bar) indicates the geometric mean (± standard error) concentrations across participants in each group. A double tick on the x-axis denotes a time interval of 3 days, representing visit days 3, 17, 31, 59, 115, and/or 157. Peak concentrations were measured at 1 hour after the last IV infusion in treatment groups T1, T2, T4, and T5 and 3 days after the first and last SC injections in group T3/P3. Concentrations were truncated at the limit of quantification of the least sensitive assay. T1: 20 mg/kg IV q 4 weeks with 40 mg/kg IV loading; T2: 40 mg/kg IV q 8 weeks; T3 (P3): 5 mg/kg SC q 2 weeks with 40 mg/kg IV loading; T4: 10 mg/kg IV q 8 weeks; T5: 30 mg/kg IV q 8 weeks. Env, HIV-1 envelope glycoprotein; PP, per-protocol; P3, placebo group 3; q, quodque; SC, subcutaneous; T1, treatment group 1; T2, treatment group 2; T3, treatment group 3; T4, treatment group 4; T5, treatment group 5.

Fig 3 and Table 2 depict the group-level geometric mean serum VRC01 concentrations measured by ELISA and neutralization assays for participants in the PP cohort. Individual-level VRC01 concentrations measured by ELISA are shown in S1 Fig for participants in the MITT (S1 Fig, panel A) and PP (S1 Fig, panel B) cohorts; summaries of group-level serum concentrations by time point are provided in S1 Table (MITT cohort) and S2 Table (PP cohort). Detailed population pharmacokinetics modeling of serum concentration data in the MITT cohort has been previously described [35]. In the PP cohort (S2 Table), as measured by ELISA, average (geometric mean) serum VRC01 levels in T1 3 days after the first (40 mg/kg) and second (20 mg/kg) IV infusion were 422 and 260 μg/ml, respectively. Average peak levels 1 hour after the last infusion were 796 μg/ml. Average trough levels 28 days post infusion were 69 μg/ml after the first infusion and 51, 46, 45, 43, and 46 μg/ml after infusions 2–6, respectively. VRC01 remained detectable (≥1.1 μg/ml) in 6/9 (67%) of PP participants 12 weeks after the last IV infusion in T1.

**Table 2:** Summary of serum concentration levels (μg/ml) in the PP cohort. Shown are geometric mean, 95% CI around the mean, and proportion of observations above the LLoQ of the ELISA assay.

Group	Peak^A^ post last dose		Week 24 trough		Week 32^B^	
	Mean (95% CI)	Percent above LLoQ^C^	mean (95% CI); *n*	% above LLoQ^C^	Mean (95% CI)	Percent above LLoQ^C^
**T1: 20 mg/ kg IV q 4 weeks**^D^	796 (615, 1,030)	7/7 (100%)	46 (38, 56)	10/10 (100%)	2 (<1.1, 4.4)	6/9 (67%)
**T2: 40 mg/kg IV q 8 weeks**	1549 (1,306, 1,838)	14/14 (100%)	27 (20, 37)	17/17 (100%)	2.4 (1.5, 3.9)	12/15 (80%)
**T4: 10 mg/kg IV q 8 weeks**	420 (356, 494)	9/9 (100%)	6 (5, 9)	10/10 (100%)	<1.1 (NA)	0/9 (0%)
**T5: 30 mg/kg IV q 8 weeks**	1,177 (1,033, 1,340)	8/8 (100%)	16 (10, 27)	8/8 (100%)	1.2 (<1.1, 3)	3/8 (38%)
**T3: 5 mg/kg SC q 2 weeks**^D^	60 (50, 72)	10/10 (100%)	34 (25, 46)	11/11 (100%)	1.8 (<1.1, 3.9)	6/10 (60%)
**P3 placebo**	<1.1	0/2 (0%)	<1.1	0/2 (0%)	<1.1	0/2 (0%)

^A^Peak was measured at 1 hour after IV infusion in groups T1, T2, T4, and T5 and 3 days after SC injection in group T3/P3.

^B^Week 32 is 12 weeks after the final IV infusion in T1, 16 weeks after the final IV infusion in T2, T4, and T5, and 10 weeks after the final SC injection in T3.

^C^LLoQ of the assay was 1.1 μg/mL.

^D^A loading dose of 40 mg/kg VRC01 was administered in T1 and T3.

**Abbreviations**: LLoQ, lower limit of quantification; PP, per-protocol; P3, placebo group 3; q, quodque; SC, subcutaneous; T1, treatment group 1; T2, treatment group 2; T3, treatment group 3; T4, treatment group 4; T5, treatment group 5.

In T2, average serum VRC01 levels 3 days after each of the 3 bimonthly IV infusions of 40 mg/kg were 441, 414, and 486 μg/ml, respectively. The corresponding day 3 post-infusion levels in T5 (3 bimonthly IV infusions of 30 mg/kg) were 153, 183, and 242 μg/ml and in T4 (3 bimonthly IV infusions of 10 mg/kg) were 84, 87, and 113 μg/ml. Average peak levels 1 hour after the last infusion were 1,549; 1,177; and 420 μg/ml in T2, T5, and T4, respectively. Average trough levels 56 days after the first, second, and third IV infusion in T2 were 20, 24, and 27 μg/ml, respectively. In T5, these trough values were 12, 13, and 16 μg/ml. In T4, these trough values were 4, 4, and 6 μg/ml. VRC01 remained detectable (≥1.1 μg/ml) 16 weeks after the last infusion in 80%, 38%, and 0% of participants in T2, T5, and T4, respectively. The estimated mean ± standard deviation (SD) serum half-life of VRC01 in T4 and T5 was 11.4 ± 4.3 days (median = 11.0 days) and 13.7 ± 6.5 days (median = 10.2 days), respectively. Limited levels of VRC01 accumulation were observed in T4 and T5 (S3 Table). These results are consistent with previously reported half-life and drug accumulation estimates for VRC01 [33] and are independent of dose levels [35].

In T3, the average serum VRC01 level 3 days after IV infusion of 40 mg/kg was 416 μg/ml. The 3-day levels after the first and last SC injection of 5 mg/kg were 170 and 60 μg/ml, respectively, the latter concentration being a more accurate reflection of levels achieved by low-dose SC injection, with minimal contribution from the high-dose IV loading. Similarly, average trough levels measured 14 days after each subsequent 2-weekly SC injection decreased steadily from 85 μg/ml after the first injection to 34 μg/ml after the final injection. VRC01 remained detectable (≥1.1 mg/ml) in 60% of participants 10 weeks after the last injection. No VRC01 was detected in the placebo group (P3). The relatively stable pharmacokinetics after multiple IV and SC dosing suggests the absence of anti-VRC01 antibodies, which was confirmed via ADA assay in a highly sensitive MSD platform (S4 Table).

### Immunologic activity of VRC01 post infusion

To test whether VRC01 retained expected neutralizing activity post infusion, serum samples from the same time points used for the ELISA-based PK analysis were assayed against 3 Env-pseudotyped viruses (tier 1A: MN.3, MW965.26; tier 2: PVO.4) in the TZM-bl neutralization assay. As shown in Fig 3, serum concentrations of VRC01 measured by neutralizing activity closely approximated the concentrations measured by ELISA, with even the lowest IV dose (10 mg/kg) demonstrating expected magnitude and breadth of neutralizing activity, regardless of whether neutralization was measured with the tier 1 viruses or the tier 2 virus. Similar results have been reported previously over a shorter duration and lower frequency of VRC01 administration [33].

**Fig 4:**
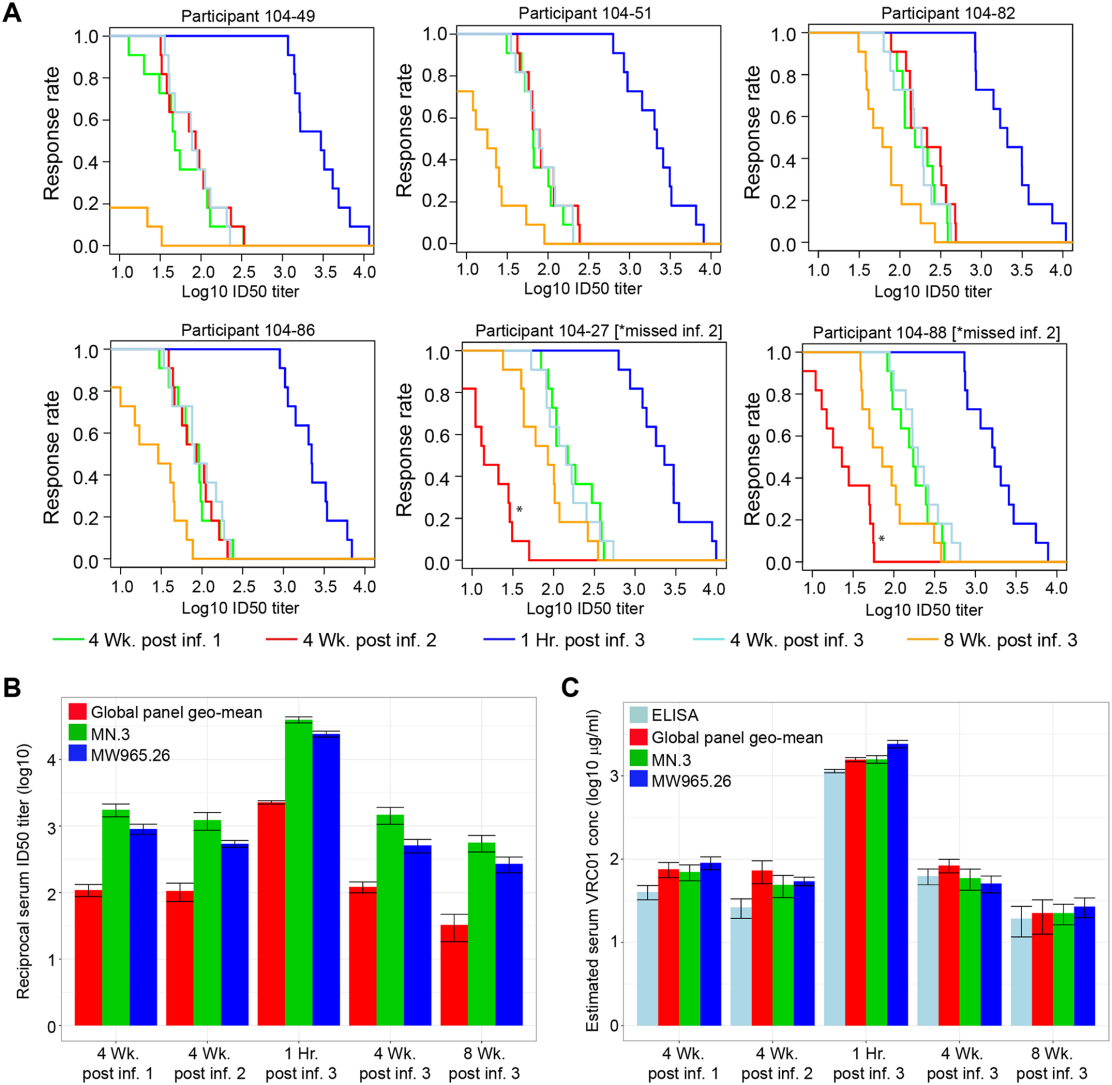
Serum neutralizing activity against multiple isolates at 4 weeks after the first, second, and third infusions and at 1 hour and 8 weeks following the third infusion in 6 T5 participants. **(A)** MB curves based on TZM-bl neutralization assay results against 11 tier 2 viruses for each of the 6 individuals in T5. Two individuals who missed their second infusion are indicated by an * at the red line (4 weeks post infusion 2). MB curves based on results with HIV-1 Env-pseudotyped viruses PVO.4, 398F1, CNE8, X2278, 246-F3, TRO.11, CNE55, CH119, 25710, X1632, and Ce0217. Response rate on the y-axis is the percent of the 11 viruses neutralized at serum dilutions (Log10 ID50 titer) shown on the x-axis. **(B)** Serum ID50 neutralization titers against the 11 tier 2 and 2 tier 1 isolates. Shown are the geometric mean and standard error of the mean over the ID50 titers of 4 T5 individuals, excluding the 2 individuals who missed their second infusion. **(C)** VRC01 serum concentration measured by ELISA and TZM-bl against tier 1 and tier 2 isolates. Shown are the geometric mean and standard error of the mean over the estimated concentration of VRC01 in 4 T5 individuals, excluding the 2 individuals who missed their second infusion. Individual level neutralization data for participants can be found in S5 Table. Env, HIV-1 envelope glycoprotein; ID50, 50% infectious dose; MB, magnitude-breadth; T5, treatment group 5.

To examine post-infusion neutralizing activity in greater detail, serum samples from 6 T5 (30 mg/kg) participants obtained 4 weeks after each of the 3 IV infusions every 8 weeks and 1 hour and 8 weeks after the third infusion were assessed for neutralizing activity against a multiclade panel of 11 additional tier 2-circulating strains of HIV-1 that represent globally circulating strains and exhibit a range of known sensitivities to VRC01. As shown in Fig 4A, very potent broadly neutralizing activity was seen at 1 hour post infusion in all 6 participants that subsequently declined in magnitude and breadth by 4 weeks post infusion. Notably, equivalent magnitude and breadth of neutralizing activity was seen 1 month post each infusion in the 4 participants who received all 3 infusions and at 4 weeks post first and third infusion in the 2 participants (104–27 and 104–88) who missed the second infusion. Results for these latter 2 participants also illustrate that tier 2 virus-neutralizing activity was detectable against >80% of the viruses 12 weeks after a single infusion. As expected, the level of activity present at 8 weeks post third infusion in all 6 participants was intermediate between the levels seen after 4 and 12 weeks. Fig 4B shows that the geometric mean serum neutralization ID50 titer against tier 2 viruses was approximately 1:100 at 4 weeks post each infusion and that this titer was approximately 1:30 at the trough time point (8 weeks post infusion, measured post final infusion only). S5 Table contains the ID50 titer data for each participant. Fig 4C illustrates the consistency of measuring VRC01 concentration in these 6 T5 participants via ELISA or via TZM-bl ID50 neutralization assay estimation using either tier 1A or a global panel of tier 2 HIV-1 Env-pseudoviruses. Overall, these results indicate no loss of expected VRC01 neutralizing activity after multiple IV infusions over a period of at least 24 weeks. Table 3 shows that midpoint and trough serum VRC01 levels achieved in this study following each group’s final dose are consistent with concentrations that are known to neutralize a majority of circulating strains of clade B and clade C HIV-1 in vitro.

**Table 3:** Predicted coverage based on in vitro neutralizing activity of VRC01.

	Percent breadth^A^			
	Clade B		Clade C	
	IC_50_	IC_80_	IC_50_	IC_80_
**High dose (30 mg/kg)**				
Midpoint (30 μg/ml)	94	93	84	75
Trough (12 μg/ml)	94	93	80	73
**Low dose (10 mg/kg)**				
Midpoint (16 μg/ml)	94	93	80	73
Trough (4 μg/ml)	93	82	75	58

^A^Shown are the percent of clade B (*n* = 56) and clade C (*n* = 200) HIV-1 Env-pseudotyped viruses neutralized at IC_50_/IC_80_ that correspond to the approximated midpoint (4 weeks post infusion) and trough (8 weeks post infusion) levels of VRC01 in participants who received IV infusions of either 30 mg/kg or 10 mg/kg of product. Values for clade B viruses were obtained by using the CATNAP tool in the Los Alamos National Laboratory HIV Sequence Database (https://www.hiv.lanl.gov/content/index). Values for the clade C viruses are from Wagh et al. [51].

**Abbreviations**: Env, HIV-1 envelope glycoprotein; IC_50_, 50% inhibitory concentration; IC_80_, 80% inhibitory concentration.

**Fig 5:**
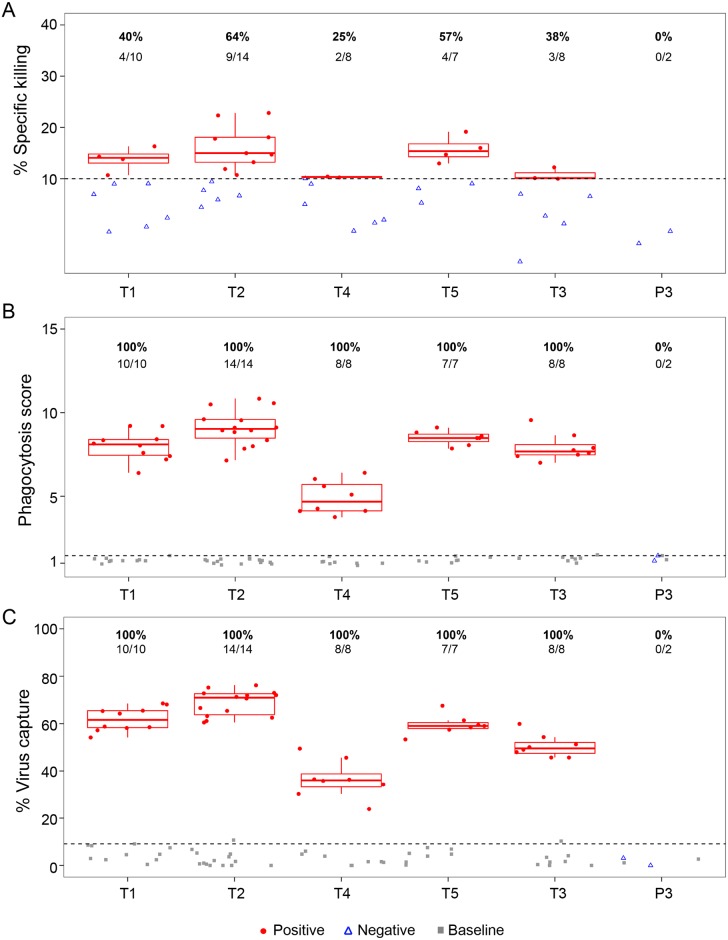
VRC01 mAb retains nonneutralizing Fc effector functions postintravenous or postsubcutaneous administration in the PP cohort. **(A)** ADCC using the Luciferase-HIV CH0505.LucR T2A.ecto/293T/17 assay, **(B)** average phagocytosis score, and **(C)** average virus capture percentage are presented (average of 2 replicate experiments). Grey squares indicate baseline (preadministration) time points; red circles represent positive responses at postadministration time points; open blue triangles represent negative responses at postadministration time points. Horizontal dashed lines represent the positivity cutoff based on the 95th percentile of all baseline visits. Percent responders and the number of positive responders/total number are shown above each treatment group. T1: 20 mg/kg IV q 4 weeks with 40 mg/kg IV loading; T2: 40 mg/kg IV q 8 weeks; T3 (P3): 5 mg/kg SC q 2 weeks with 40 mg/kg IV loading; T4: 10 mg/kg IV q 8 weeks; T5: 30 mg/kg IV q 8 weeks. ADCC, antibody-dependent cellular cytotoxicity; Fc, fragment crystallizable; mAb, monoclonal antibody; PP, per-protocol; P3, placebo group 3; q, quodque; SC, subcutaneous; T1, treatment group 1; T2, treatment group 2; T3, treatment group 3; T4, treatment group 4; T5, treatment group 5.

Non-neutralizing antibody effector functions were assessed in serum from study participants. ADCC, ADCP, and virion capture activities were assessed at 3 days post second administration. ADCC was assessed and found to be weak or absent (Fig 5A), which agrees with the low ADCC activity exhibited by non-infused VRC01 in this assay (S2 Fig). Despite this low ADCC effector function, 100% of serum samples from all 5 treatment groups exhibited ADCP activity against glycoprotein 140 (gp140)-coated microspheres (Fig 5B) and demonstrated capacity of the fragment crystallizable (Fc) portion of this mAb to engage monocyte effector cells. All specimens from PP cohort participants also demonstrated capacity to bind infectious virions (Fig 5C).

**Fig 6:**
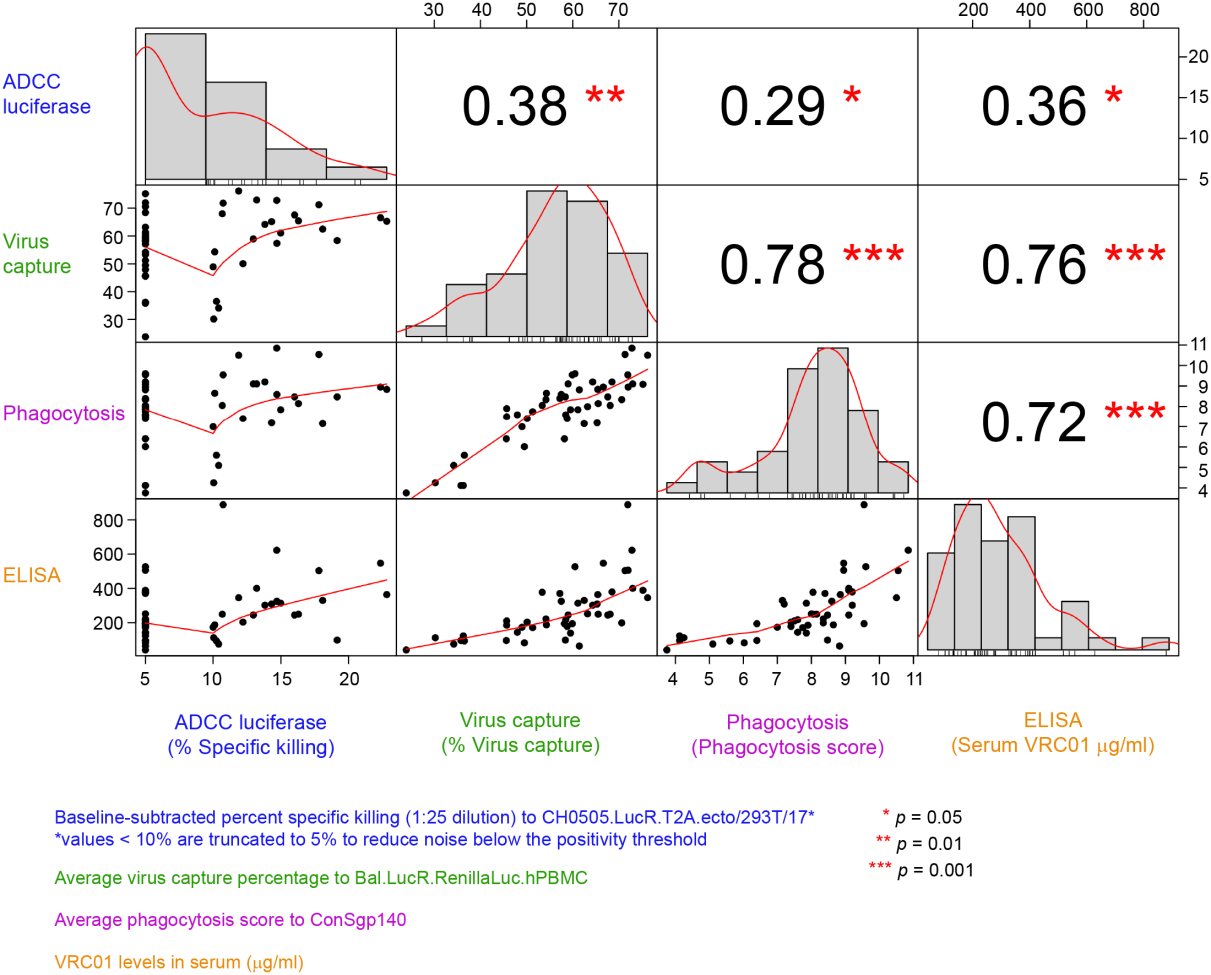
Correlations between functional activities and VRC01 level in the PP cohort (all treatment arms). Lower diagonal squares show scatterplots of each pair of assay variables. A Lowess smoother line using locally weighted polynomial regression with a span of 2/3 was added. Upper diagonal squares show rank-based Spearman correlation coefficients for each pair of assay variables. Diagonal squares show histograms of each variable. ADCC, antibody-dependent cellular cytotoxicity; PP, per-protocol.

Lastly, the correlations between the functional activities and VRC01 serum concentration measured at 3 days post the second infusion/injection in the PP cohort (Fig 6) were assessed. The strongest and most significant correlations were observed between ADCP and virion capture activities (Spearman ρ = 0.78) and between both ADCP and virus capture and VRC01 serum concentration (ρ = 0.72 and 0.76, respectively). Statistically significant but weaker correlations were observed between ADCC activity and ADCP activities (ρ = 0.29), ADCC and virion capture (ρ = 0.38), and ADCC and VRC01 serum concentration (ρ = 0.36).

## Discussion

The current study (HVTN 104) has expanded the safety, pharmacokinetic, and functional understanding of the bnAb VRC01 by evaluating multiple doses administered either intravenously or subcutaneously over a 16–22-week period of time in a total of 88 HIV-uninfected participants (including 4 who were randomized to placebo injections), 57 of whom completed all scheduled doses. Participants were followed for 32 weeks after their first VRC01 administration. No SAEs, dose-limiting toxicities, nor evidence for anti-VRC01 antibodies were observed. Serum VRC01 levels were detected through 12 weeks after final administration in all participants who received all scheduled doses. Mean trough concentrations after 3 IV infusions of 30 mg/kg and 10 mg/kg doses administered every 8 weeks were above levels known to neutralize a majority of circulating strains in vitro (IC50 > 5 μg/ml). Post-infusion/injection serum VRC01 retained expected functional activity including virus neutralization, ADCC, phagocytosis, and virion capture.

The study evaluated 5 different dosing regimens that were selected based upon previous clinical trials [33,34] as well as prior NHP challenge studies demonstrating efficacy in preventing SHIV acquisition [21–24] and aimed to achieve trough VRC01 levels well above the range shown to neutralize the majority of clade B and clade C HIV-1 strains in vitro [18]. The relevance of the current findings are that, although the use of anti-HIV antibodies for immunoprophylaxis has been studied in NHP models for more than 20 years [52–59], recent interest in their use for HIV prevention in humans has increased after studies identified multiple bnAbs that exhibit potent activity against a majority of HIV-1 strains [13–16]. Such bnAbs, alone or in combination, could provide a novel approach to anti-HIV-1 immunoprophylaxis, and insights from clinical trials of bnAbs could inform the development of immunogens and effective HIV-1 vaccines. VRC01 is among a class of potent bnAbs targeting the functionally conserved CD4bs region of HIV. VRC01 was previously shown to be safe, well tolerated, and non-immunogenic in a small dose-escalation trial in 28 HIV-uninfected participants who received 1 (20 mg/kg) or 2 IV infusions 4 weeks apart of either 5, 20, or 40 mg/kg or 2 SC injections of 5 mg/kg [33]. A similar trial was conducted in 23 HIV-infected participants [34].

HVTN 104 found that VRC01 administered subcutaneously and in different IV doses was safe and well tolerated, with no dose-associated toxicities. There were a limited number of local site reactions, but patterns of AEs did not differ significantly by route or frequency of product administration or the dose used. Peak serum VRC01 concentrations 1 hour after the final IV administration demonstrated dose proportionality, with the highest levels achieved following the largest dose (40 mg/kg every 8 weeks) and lowest levels after the lowest IV dosing regimen (10 mg/kg every 8 weeks) (Table 2). The trough following the final VRC01 administration for each dosing regimen ranged from 6 to 46 μg/ml (trough based on the interval between previous administrations). By quantitative assays to measure auto-antibodies and assessment of VRC01 concentrations and neutralization functionality over time, there was no evidence of the development of anti-idiotypic or other autoantibodies, nor was there significant drug accumulation after repeated dosing.

This trial also examined 2 doses of VRC01 (10mg/kg and 30 mg/kg) administered over a longer interval between IV infusions (8 weeks) that informed the design of 2 proof-of-concept efficacy trials (www.ampstudy.org, ampstudy.org.za)[60]. Of note, the range of VRC01 serum levels of participants receiving either the 10 mg/kg or the 30 mg/kg IV regimens every 8 weeks overlapped for more than 60% of the time (Fig 3). The evaluation of 11 SC injections received every 2 weeks is also relevant for the design of studies of post-exposure prophylaxis for infants born to HIV-infected mothers who were not virologically suppressed on ARV therapy [61]. Serum VRC01 concentrations remained at or above 30 μg/ml when 5 mg/kg was administered subcutaneously every 2 weeks through the final trough time point, tailing to 1.8 μg/ml by the final assessment point at week 32.

The viral neutralization activity based on the IC50 potency of trough levels 24 weeks after IV infusion with either 10 or 30 mg/kg of VRC01 suggests that, of HIV Env-pseudotyped virus strains evaluated, 93%–94% of tier 2 clade B strains and 75%–84% of tier 2 clade C strains would be neutralized; while coverage based on a more stringent IC80 potency suggests that 82%–93% of clade B strains and 58%–75% of clade C strains would be neutralized at trough time points (Table 3). Actual midpoint and trough levels measured were similar to what would be predicted based on in vitro neutralization assays.

The VRC01 levels present in the serum samples tested in this study demonstrated an ability to avidly capture virions in vitro and to mediate ADCP, suggesting functionalities in addition to neutralization, although limited ADCC activity was seen. In addition, the serum VRC01 levels measured by ELISA were found to be correlated with ADCP and virion capture activities but less with ADCC activity. This lack of correlation with ADCC activity and the overall low ADCC activity exhibited by VRC01 compared to other CD4bs bnAbs (S2 Fig) suggests differences in the ability of VRC01 to bind gp140 microspheres or cell surface Env versus infectious virus and differences in affinity of VRC01 binding to Fc receptor (FcR) expressed on the cell surface.

The current study has reported an estimated terminal half-life of about 15 days for different dose levels of VRC01, whether administered intravenously or subcutaneously. Prior studies of the pharmacokinetics of VRC01 in HIV-uninfected adults have compared multiple doses (5, 20, and 40 mg/kg) given intravenously at 0 and 28 days and a low dose (5 mg/kg) given subcutaneously at 0 and 28 days [33]. In those studies, IV infusion of VRC01 resulted in high peak serum levels (up to 1,000 μg/ml) that fell rapidly over a few days, with further slow decline over several weeks from catabolism. However, use of smaller SC doses that allowed for a slow diffusion of IgG into the vasculature and lymphatics resulted in stable higher trough IgG serum levels, which remained constant between consecutive SC IgG infusions [33]. Both routes of administration displayed similar half-lives. It is possible that trough levels rather than peak levels are of greatest importance for sufficient prevention of HIV acquisition when administering passive immunotherapy. In HVTN 104, the trough level found after 5 mg/kg SC dosing every 2 weeks was 34 μg/ml and ranged from 6 to 27 μg/ml when VRC01 was administered intravenously every 8 weeks at 10 mg/kg or 40 mg/kg, respectively. It is conceivable that smaller doses given more frequently via the SC route could have the potential to provide sufficient and consistent trough levels as compared to higher IV doses given less frequently. However, in HVTN 104, almost half of the missed visits occurred among those assigned to receive injections every 2 weeks; hence, the development of bnAbs with longer half-lives is clearly warranted. Recent studies have suggested that through modification of the Fc portion of the mAb to better bind the neonatal Fc receptor, bnAbs may be able to have extended half-lives [21,62,63], potentially allowing for product administration as infrequently as every 6 months.

The less than optimal adherence to the study schedule amongst those assigned to receive injections every 2 weeks or infusions every 4 weeks helped inform the selection of the every-8-week dosing frequency for the 2 Antibody Mediated Prevention (AMP) efficacy trials (www.ampstudy.org, ampstudy.org.za). Other bnAbs that have greater potency and breadth are under investigation [13–16] and could be more effective than VRC01 for protection against HIV. Combining bnAbs for prophylaxis could also expand breadth and help prevent viral escape by inhibiting HIV-1 at multiple sites of vulnerability via complementary mechanisms of action [51,64]. In addition, the development of synergistic bispecific antibodies [65,66] that could target more than 1 epitope is another intriguing approach. Finally, if the AMP studies demonstrate that VRC01 can protect against HIV-1 acquisition when administered either at 10 mg/kg or 30 mg/kg every 8 weeks, then antibody concentrations achieved in HVTN 104 may be able to be used to inform the assessment of the immune responses to new vaccine candidates to prevent HIV transmission.

The limitations of this study include the relative small sample size describing 5 different VRC01 administration regimens, missing data from participants who were not completely adherent to the study visit schedule, and limitations in the interpretation of the clinical significance of the in vitro findings. The pharmacokinetic data from this study must be interpreted cautiously, because the study windows were narrow and the PP cohort only included values from participants who received all of their study infusions, providing PK estimates that may not be generalizable to ongoing efficacy studies of VRC01 that have wider windows and will evaluate a larger number of infusions and longer periods of product exposure. Because of concerns early in this trial about potential particulate matter that turned out to be frozen product (resulting in longer thawing periods), some VRC01 infusions and injections were not administered, resulting in collecting less data than had initially been planned. The study had relatively sparse PK sampling immediately after infusion or injection, limiting inferences that can be drawn regarding early distribution of VRC01 in the blood. Studies of VRC01 concentrations in mucosal secretions and tissues are currently underway and should help further understanding of the penetration of systemically administered VRC01 in different body compartments.

## Conclusions

VRC01 administered intravenously or subcutaneously was safe and well tolerated. Product-related AEs were uncommon and generally transient and mild. After a 40 mg/kg IV loading dose, VRC01 levels were maintained at >30 μg/ml for several weeks through either IV or SC administration every 2 weeks. Trough levels of 4, 16, or 27 μg/ml were maintained with IV infusions of 10, 30, and 40 mg/kg every 8 weeks, respectively. The trough data support the rationale that VRC01 administered every 8 weeks intravenously should be evaluated in studies of HIV-1 immunoprophylaxis. The first efficacy trials of VRC01 are underway in the AMP studies (Clinicaltrials.gov NCT02716675 and NCT02568215, and www.ampstudy.org, ampstudy.org.za), evaluating anti-HIV-1 activity among 4,200 at-risk men and transgender people who have sex with men in North and South America and at-risk young women in sub-Saharan Africa. The findings should help inform the development of both passive and active immunization strategies to prevent HIV-1 infection. The trough levels seen after SC administered VRC01 every 2 weeks suggest that this approach may be particularly appropriate for immunoprophylaxis for infants born to HIV-infected mothers. Studies are being developed to test this hypothesis. In summary, HVTN 104 found that VRC01 delivered via IV or SC routes was safe and well tolerated, and results from in vitro assays suggest that the levels achieved in clinical specimens displayed a wide range of functional anti-HIV activities.

## Supporting information

S1 CONSORT Checklist HVTN 104 CONSORT checklist. CONSORT, Consolidated Standards of Reporting Trials; HVTN, HIV Vaccine Trials Network.
(DOC)

S1 Protocol HVTN 104 protocol, version 2. HVTN, HIV Vaccine Trials Network.
(PDF)

S1 Fig Individual-specific VRC01 serum concentration level. Data are presented for **(A)** the MITT cohort and **(B)** the PP cohort. A double tick on the x-axis denotes a time interval of 3 days, representing visit days 3, 17, 31, 59, 115, and/or 157. T1: 20 mg/kg IV q 4 weeks with 40 mg/kg IV loading; T2: 40 mg/kg IV q 8 weeks; T3/ (P3): 5 mg/kg SC q 2 weeks with 40 mg/kg IV loading; T4: 10 mg/kg IV q 8 weeks; T5: 30 mg/kg IV q 8 weeks. MITT, modified intent-to-treat; PP, per-protocol; P3, placebo group 3; q, quodque; SC, subcutaneous; T1, treatment group 1; T2, treatment group 2; T3, treatment group 3; T4, treatment group 4; T5, treatment group 5.
(TIF)

S2 Fig Comparative ADCC activity of CD4bs bnAbs. Shown is the percentage of specific killing using the Luciferase-HIV CH0505.LucR T2A.ecto/293T/17 assay. ADCC, antibody-dependent cellular cytotoxicity; bnAb, broadly neutralizing antibody; CD4bs, CD4 binding site.
(TIF)

S1 Table Summary of VRC01 serum concentration levels by group and by time point in the MITT cohort. T1: 20 mg/kg IV q 4 weeks with 40 mg/kg IV loading; T2: 40 mg/kg IV q 8 weeks; T3 (P3): 5 mg/kg SC q 2 weeks with 40 mg/kg IV loading; T4: 10 mg/kg IV q 8 weeks; T5: 30 mg/kg IV q 8 weeks. MITT, modified intent-to-treat; P3, placebo group 3; q, quodque; SC, subcutaneous; T1, treatment group 1; T2, treatment group 2; T3, treatment group 3; T4, treatment group 4; T5, treatment group 5.
(DOCX)

S2 Table Summary of VRC01 serum concentration levels by group and by time point in the PP cohort. T1: 20 mg/kg IV q 4 weeks with 40 mg/kg IV loading; T2: 40 mg/kg IV q 8 weeks; T3 (P3): 5 mg/kg SC q 2 weeks with 40 mg/kg IV loading; T4: 10 mg/kg IV q 8 weeks; T5: 30 mg/kg IV q 8 weeks. PP, per-protocol; P3, placebo group 3; q, quodque; SC, subcutaneous; T1, treatment group 1; T2, treatment group 2; T3, treatment group 3; T4, treatment group 4; T5, treatment group 5.
(DOCX)

S3 Table Noncompartmental pharmacokinetic analysis of VRC01 levels at second and third peak, trough, and AUC in T4 and T5 PP participants. T4: 10 mg/kg IV q 8 weeks; T5: 30 mg/kg IV q 8 weeks. AUC, area under the curve; PP, per-protocol; q, quodque; T4, treatment group 4; T5, treatment group 5.
(DOCX)

S4 Table Lack of detectable ADA in VRC01 recipients. Serum samples were assayed at a 1:4 dilution in quadruplicate. The data provided are mean ECL units with standard deviation (SD) and percent relative SD (%RSD). Each assay plate had plate-dependent positivity criteria, which is provided for each sample and was determined by using a reference standard that defined the lower limit of quantification. ADA, antidrug antibody; ECL, electrochemiluminescence.
(XLSX)

S5 Table Individual-level serum ID50 neutralization titers against the 11 tier 2 and 2 tier 1 isolates. ID50, 50% infectious dose.
(XLSX)

S1 Data Deidentified data underlying the reported findings in HVTN 104. Participant characteristics and study/treatment adherence: participant_details.csv (masked participant details including demographics, treatment group, and reasons for treatment discontinuation and study termination); treatment.csv (received treatment details by SPA visit). Safety: reactogenicity_listing.csv (all local and systemic reactogenicity events); reactogenicity_summary.csv (reactogenicity events summarized at the maximum reportable severity grade by participant and visit number); ae_listing.csv (all nonreactogenicity AEs); ae_summary.csv (AEs summarized at the maximum severity and relatedness by participant, MedDRA Preferred Term, and System Organ Class); safety_laboratory_graded.csv (safety laboratory results and corresponding grades are listed by participant and visit collected [prior to study product administration]). Drug level and immune responses (functional activities): ada_assay.csv (processed ADA assay data, as described in the methods section, for ADA activity [end point variable name: *result*]); aly_assay.csv (processed ELISA assay data, as described in the methods section, for ELISA-based VRC01 serum-concentration [end point variable name: *result*]); nab_assay.csv (processed nAb assay data, as described in the methods section, for TZM-bl neutralization assay-based VRC01 serum concentration including both ID50 and ID80 values [end point variable names: *concentration50* and *concentration 80*]); phagocytosis_assay.csv (processed ADCP assay data, as described in the methods section, for ADCP functional activity [end point variable names: *avg_phagocytosis_score* and *response*]); virus_capture_assay.csv (processed IVCA data, as described in the methods section, for infectious virion capture functional activity [end point variable names: *avg_capture_percentage* and *response*]); adcc_assay.csv (processed ADCC assay data, as described in the methods section, for ADCC functional activity [end point variable names: *blsub_pct_specific_killing* and *response*]). ADA, antidrug antibody; ADCC, antibody-dependent cellular cytotoxicity; ADCP, antibody-dependent cellular phagocytosis; AE, adverse event; HVTN, HIV Vaccine Trials Network; ID50, 50% infectious dose; ID80, 80% infectious dose; IVCA, infectious virion capture assay; nAb, neutralizing antibody; SPA, study product administration.
(ZIP)

## Abbreviations

ADA: antidrug antibody
ADCC: antibody-dependent cellular cytotoxicity
ADCP: antibody-dependent cellular phagocytosis
AE: adverse event
ALT: alanine transaminase
AMP: Antibody Mediated Prevention
ARV: antiretroviral
AST: aspartate transaminase
AUC: area under the curve
BAMA: Binding Antibody Multiplex Assay
bnAb: broadly neutralizing antibody
CD4: cluster of differentiation 4
CD4bs: CD4 binding site
CONSORT: Consolidated Standards of Reporting Trials
CRS: cytokine release syndrome
DAIDS: Division of AIDS
Env: HIV-1 envelope glycoprotein
Fc: Fragment crystallizable
FcR: Fc receptor
gp120: glycoprotein 120
gp140: glycoprotein 140
HVTN: HIV Vaccine Trials Network
IC: immune complex
IC50: 50% inhibitory concentration
IC80: 80% inhibitory concentration
ID50: 50% infectious dose
ID80: 80% infectious dose
IgG: immunoglobulin G
IMC: infectious molecular clone
IND: Investigational New Drug
IV: intravenous
IVCA: infectious virion capture assay
Luc: luciferase
mAb: monoclonal antibody
mAbs: monoclonal antibodies
MITT: modified intent-to-treat
MSD: Meso Scale Discovery
NHP: nonhuman primate
NIAID: National Institute of Allergy and Infectious Diseases
NIH: National Institutes of Health
P3: placebo group 3
PK: pharmacokinetic
PP: per-protocol
PSRT: Protocol Safety Review Team
RLU: Relative Luminescence Units
RSV: respiratory syncytial virus
SAE: serious adverse event
SC: subcutaneous
SCHARP: Statistical Center for HIV/AIDS Research & Prevention
SD: standard deviation
SDMC: Statistical Data Monitoring Center
SHIV: simian-human immunodeficiency virus
SMB: Safety Monitoring Board
T1: treatment group 1
T2: treatment group 2
T3: treatment group 3
T4: treatment group 4
T5: treatment group 5
VRC: Vaccine Research Center
