# Supplementary material for: Safety, pharmacokinetics, and immunological activities of multiple intravenous or subcutaneous doses of an anti-HIV monoclonal antibody, VRC01, administered to HIV-uninfected adults: Results of a phase 1 randomized trial
Source: PLoS Med. 2017 Nov 14;14(11):e1002435. doi: 10.1371/journal.pmed.1002435 (PMC5685476; doi:10.1371/journal.pmed.1002435)
Supplement: S1 Protocol — HVTN, HIV Vaccine Trials Network. (PDF) [file pmed.1002435.s002.pdf]

THIS PROTOCOL IS BEING PROVIDED TO ACCOMPANY THE MANUSCRIPT REPORTING RESULTS FROM THE HVTN 104 TRIAL. FOR ANY OTHER USE, PLEASE CONTACT VTN.RESEARCH@HVTN.ORG

PROTOCOL

HVTN 104

A phase 1 clinical trial to evaluate the safety and drug levels of a human monoclonal antibody, VRC-HIVMAB060-00-AB (VRC01) administered in multiple doses intravenously and subcutaneously in different dosing schedules to healthy, HIV-uninfected adults

DAIDS DOCUMENT ID 11969

IND 113, 611 - HELD BY DAIDS

CLINICAL TRIAL SPONSORED BY

Division of AIDS (DAIDS)
National Institute of Allergy and Infectious Diseases (NIAID)
National Institutes of Health (NIH)
Department of Health and Human Services (DHHS)
Bethesda, Maryland, USA

STUDY PRODUCTS PROVIDED BY

Vaccine Research Center/NIAID/NIH
Bethesda, MD

March 19, 2015
HVTN 104, Version 2.0 FOR REVIEW ONLY

FOR REVIEW ONLY

Contents

1 Ethical considerations 5

2 IRB/EC review considerations 7
2.1 Minimized risks to participants 7
2.2 Reasonable risk/benefit balance 7
2.3 Equitable subject selection 7
2.4 Appropriate informed consent 8
2.5 Adequate safety monitoring 8
2.6 Protect privacy/confidentiality 8

3 Overview 9
3.1 Protocol Team 12

4 Background 13
4.1 Rationale for trial concept 14
4.2 Mucosal specimen collection 17
4.3 Anti-VRC01 antibodies 17
4.4 VRC01: VRC-HIVMAB060-00-AB 18
4.5 Placebo for VRC01: VRC-PLAMAB068-00-AB 19
4.6 Sodium chloride placebo 19
4.7 Trial design rationale 19
4.8 Plans for future product development and testing 25
4.9 Preclinical studies 25
4.10 Clinical studies 30
4.11 Potential risks of study products and administration 34

5 Objectives and endpoints 36
5.1 Primary objectives and endpoints 36
5.2 Secondary objectives and endpoints 36
5.3 Exploratory objectives 37

6 Statistical considerations 39
6.1 Accrual and sample size calculations 39
6.2 Randomization 42
6.3 Blinding 42
6.4 Statistical analysis 42

7 Selection and withdrawal of participants 47
7.1 Inclusion criteria 47
7.2 Exclusion criteria 49
7.3 Participant departure from the study product administration schedule or withdrawal 52

8 Study product preparation and administration 55
8.1 Study product regimen 55
8.2 Study product formulation 56
8.3 Preparation of study products 57
8.4 Administration 61
8.5 Acquisition of study products 62
8.6 Pharmacy records 63
8.7 Final disposition of study products 63

9 Clinical procedures 64

FOR REVIEW ONLY

9.1 Informed consent 64
9.2 Pre-enrollment procedures 66
9.3 Enrollment and study product administration visits 67
9.4 Follow-up visits 68
9.5 Mucosal secretion sampling 70
9.6 HIV counseling and testing 71
9.7 Contraception status 72
9.8 Urinalysis 73
9.9 Assessments of reactogenicity 73
9.10 Visit windows and missed visits 74
9.11 Early termination visit 75
9.12 Pregnancy 75

10 Laboratory 76
10.1 HVTN CRS laboratory procedures 76
10.2 Total blood volume 76
10.3 Primary timepoint 76
10.4 Drug levels 76
10.5 Endpoint assays: humoral 77
10.6 Genotyping 77
10.7 Exploratory studies 78
10.8 Other use of stored specimens 78
10.9 Biohazard containment 78

11 Safety monitoring and safety review 79
11.1 Safety monitoring and oversight 79
11.2 Safety reporting 80
11.3 Safety pause and prompt PSRT AE review 82
11.4 Review of cumulative safety data 83
11.5 Study termination 83

12 Protocol conduct 84
12.1 Social impacts 85
12.2 Emergency communication with study participants 85

13 Version history 86

14 Document references (other than literature citations) 88

15 Acronyms and abbreviations 90

16 Literature cited 93

Appendix A Sample informed consent form 98

Appendix B Approved birth control methods (for sample informed consent form) .. 117

Appendix C Sample consent form for use of samples and information in other studies
119

Appendix D Table of procedures (for sample informed consent form) 123

Appendix E Laboratory procedures for Group 1 126

Appendix F Laboratory procedures for Groups 2, 4, and 5 127

Appendix G Laboratory procedures for Group 3 128

Appendix H Procedures at HVTN CRS for Group 1 129

Appendix I Procedures at HVTN CRS for Groups 2, 4, and 5 131

FOR REVIEW ONLY

Appendix J Procedures at HVTN CRS for Group 3 133

Appendix K Added Criteria to the DAIDS AE Grading Table - Immune System Disorders 135

FOR REVIEW ONLY

1 Ethical considerations

Multiple candidate HIV vaccines will need to be studied simultaneously in different populations around the world before a successful HIV preventive vaccine is found. It is critical that universally accepted ethical guidelines are followed at all sites involved in the conduct of these clinical trials. The HIV Vaccine Trials Network (HVTN) has addressed ethical concerns in the following ways:

- HVTN trials are designed and conducted to enhance the knowledge base necessary to find a preventive vaccine, using methods that are scientifically rigorous and valid, and in accordance with Good Clinical Practice (GCP) guidelines.

- HVTN scientists and operational staff incorporate the philosophies underlying major codes [1-3], declarations, and other guidance documents relevant to human subjects research into the design and conduct of HIV vaccine clinical trials.

- HVTN scientists and operational staff are committed to substantive community input—into the planning, conduct, and follow-up of its research—to help ensure that locally appropriate cultural and linguistic needs of study populations are met. Community Advisory Boards (CAB) are required by DAIDS and supported at all HVTN research sites to ensure community input.

- HVTN clinical trial staff counsel study participants routinely on how to reduce HIV risk. Participants who become HIV-infected during the trial are provided counseling on notifying their partners and about HIV infection according to local guidelines. Staff members will also counsel them about reducing their risk of transmitting HIV to others.

- Participants who become HIV-infected during the trial are referred to medical practitioners to manage their HIV infection and to identify potential clinical trials they may want to join.

- The HVTN provides training so that all participating sites similarly ensure fair participant selection, protect the privacy of research participants, and obtain meaningful informed consent. During the study, participants will have their wellbeing monitored, and to the fullest extent possible, their privacy protected. Participants may withdraw from the study at any time.

- Prior to implementation, HVTN trials are rigorously reviewed by scientists who are not involved in the conduct of the trials under consideration.

- HVTN trials are reviewed by local and national regulatory bodies and are conducted in compliance with all applicable national and local regulations.

- The HVTN designs its research to minimize risk and maximize benefit to both study participants and their local communities. For example, HVTN protocols provide enhancement of participants' knowledge of HIV and HIV prevention, as well as counseling, guidance, and assistance with any social impacts that may result from research participation. HVTN protocols also include careful medical review of each

FOR REVIEW ONLY

research participant's health conditions and reactions to study products while in the study.

- HVTN research aims to benefit local communities by directly addressing the health and HIV prevention needs of those communities and by strengthening the capacity of the communities through training, support, shared knowledge, and equipment. Researchers involved in HVTN trials are able to conduct other critical research in their local research settings.

- The HVTN recognizes the importance of institutional review and values the role of in country Institutional Review Boards (IRBs) and Ethics Committees (ECs) as custodians responsible for ensuring the ethical conduct of research in each setting.

FOR REVIEW ONLY

2 IRB/EC review considerations

US Food and Drug Administration (FDA) and other US federal regulations require IRBs/ECs to ensure that certain requirements are satisfied on initial and continuing review of research (Title 45, Code of Federal Regulations (CFR), Part 46.111(a) 1-7; 21 CFR 56.111(a) 1-7). The following section highlights how this protocol addresses each of these research requirements. Each HVTN Investigator welcomes IRB/EC questions or concerns regarding these research requirements.

2.1 Minimized risks to participants

45 CFR 46.111 (a) 1 and 21 CFR 56.111 (a) 1: Risks to subjects are minimized.

This protocol minimizes risks to participants by (a) correctly and promptly informing participants about risks so that they can join in partnership with the researcher in recognizing and reporting harms; (b) respecting local/national blood draw limits; (c) performing direct observation of participants postinfusion and collecting information regarding side effects for several days postinfusion; (d) having staff properly trained in administering study procedures that may cause physical harm or psychological distress, such as blood draws, infusions, HIV testing and counseling and HIV risk reduction counseling; (e) providing HIV risk reduction counseling and checking on contraception use (for women); and (f) providing safety monitoring.

2.2 Reasonable risk/benefit balance

45 CFR 46.111 (a) 2 and 21 CFR 56 (a) 2: Risks to subjects are reasonable in relation to anticipated benefits, if any, to subjects, and the importance of the knowledge that may reasonably be expected to result.

In all public health research, the risk-benefit ratio may be difficult to assess because the benefits to a healthy participant are not as apparent as they would be in treatment protocols, where a study participant may be ill and may have exhausted all conventional treatment options. However, this protocol is designed to minimize the risks to participants while maximizing the potential value of the knowledge it is designed to generate.

2.3 Equitable subject selection

45 CFR 46.111 (a) 3 and 21 CFR 56.111 (a) 3: Subject selection is equitable

This protocol has specific inclusion and exclusion criteria for investigators to follow in admitting participants into the protocol. Participants are selected because of these criteria and not because of positions of vulnerability or privilege. Investigators are required to maintain screening and enrollment logs to document volunteers who screened into and out of the protocol and for what reasons.

FOR REVIEW ONLY

2.4 Appropriate informed consent

45 CFR 46.111 (a) 4 & 5 and 21 CFR 56.111 (a) 4 & 5: Informed consent is sought from each prospective subject or the subject's legally authorized representative as required by 45 CFR 46.116 and 21 CFR Part 50; informed consent is appropriately documented as required by 45 CFR 46.117 and 21 CFR 50.27

The protocol specifies that informed consent must be obtained before any study procedures are initiated and assessed throughout the trial (see Section 9.1). Each site is provided training in informed consent by the HVTN as part of its entering the HVTN. The HVTN requires a signed consent document for documentation, in addition to chart notes or a consent checklist.

2.5 Adequate safety monitoring

45 CFR 46.111 (a) 6 and 21 CFR 56.111 (a) 6: There is adequate provision for monitoring the data collected to ensure the safety of subjects.

This protocol has extensive safety monitoring in place (see Section 11). Safety is monitored daily by HVTN Core and routinely by the HVTN 104 Protocol Safety Review Team (PSRT). In addition, the HVTN Safety Monitoring Board (SMB) or a Data and Safety Monitoring Board (DSMB) periodically reviews study data.

2.6 Protect privacy/confidentiality

45 CFR 46.111 (a) 7 and 21 CFR 56.111 (a) 7: There are adequate provisions to protect the privacy of subjects and maintain the confidentiality of data.

Privacy refers to an individual's right to be free from unauthorized or unreasonable intrusion into his/her private life and the right to control access to individually identifiable information about him/her. The term "privacy" concerns research participants or potential research participants as individuals whereas the term "confidentiality" is used to refer to the treatment of information about those individuals. This protocol respects the privacy of participants by informing them about who will have access to their personal information and study data (see Appendix A). The privacy of participants is protected by assigning unique identifiers in place of the participant's name on study data and specimens. In the United States, research participants in HVTN protocols are protected by a Certificate of Confidentiality from the US NIH, which can prevent disclosure of study participation even when that information is requested by subpoena. Participants are told of the use and limits of the certificate in the study consent form. In addition, each staff member at each study site in this protocol signs a Confidentiality Agreement with the HVTN and each study site participating in the protocol is required to have a standard operating procedure on how the staff members will protect the confidentiality of study participants.

FOR REVIEW ONLY

3 Overview

Title

A phase 1 clinical trial to evaluate the safety and drug levels of a human monoclonal antibody, VRC-HIVMAB060-00-AB (VRC01) administered in multiple doses intravenously and subcutaneously in different dosing schedules to healthy, HIV-uninfected adults

Primary objectives

Primary objective 1:

To evaluate the safety and tolerability of VRC01, administered intravenously (IV) and subcutaneously (SC), at multiple timepoints.

Primary objective 2 (Groups 1-3):

To evaluate the serum levels of VRC01, administered IV and SC in 3 different regimens, at Month 6.

Primary objective 3 (Groups 4 and 5):

To evaluate the serum levels of VRC01 at 2 timepoints after each IV administration.

Study products and routes of administration

- VRC01: human monoclonal antibody (mAb) VRC-HIVMAB060-00-AB in formulation buffer at pH 5.8. Administered IV in 100 mL of normal saline (Sodium Chloride for Injection 0.9%, USP) or administered SC by needle and syringe injection.

- SC placebo for VRC01: the placebo for VRC01 (VRC-PLAMAB068-00-AB) is a sterile, buffered aqueous solution of 25 mM Sodium Citrate, 50 mM Sodium Chloride, 150 mM L-Arginine Hydrochloride, 10% Dextran 40 (w/w), and 0.005% Polysorbate 80 (w/w) at pH 5.8 administered SC by needle and syringe injection.

- IV placebo for VRC01: Sodium Chloride for Injection 0.9%, USP administered IV in 100 mL of normal saline (Sodium Chloride for Injection 0.9%, USP).

FOR REVIEW ONLY

Table 3-1 Schema

Dose Groups		Study product administration schedule in months (days)											
**Group**	**N**	**0**	**0.5 (14)**	**1 (28)**	**1.5 (42)**	**2 (56)**	**2.5 (70)**	**3 (84)**	**3.5 (98)**	**4 (112)**	**4.5 (126)**	**5 (140)**	**5.5 (154)**
1	20	VRC01 40mg/kg IV		VRC01 20mg/kg IV		VRC01 20mg/kg IV		VRC01 20mg/kg IV		VRC01 20mg/kg IV		VRC01 20mg/kg IV	
2	20	VRC01 40mg/kg IV				VRC01 40mg/kg IV				VRC01 40mg/kg IV			
3	20	VRC01 40mg/kg IV	VRC01 5mg/kg SC	VRC01 5mg/kg SC	VRC01 5mg/kg SC	VRC01 5mg/kg SC	VRC01 5mg/kg SC	VRC01 5mg/kg SC	VRC01 5mg/kg SC	VRC01 5mg/kg SC	VRC01 5mg/kg SC	VRC01 5mg/kg SC	VRC01 5mg/kg SC
	4	IV placebo for VRC01	SC placebo for VRC01	SC placebo for VRC01	SC placebo for VRC01	SC placebo for VRC01	SC placebo for VRC01	SC placebo for VRC01	SC placebo for VRC01	SC placebo for VRC01	SC placebo for VRC01	SC placebo for VRC01	SC placebo for VRC01
4	12	VRC01 10mg/kg IV				VRC01 10mg/kg IV				VRC01 10mg/kg IV			
5	12	VRC01 30mg/kg IV				VRC01 30mg/kg IV				VRC01 30mg/kg IV			
Total	88	Intravenous (IV) doses administered in 100 mL of normal saline over 1 hr<br>Subcutaneous (SC) doses administered by needle and syringe injection											

Note: Groups 1-3 will enroll simultaneously. Groups 1 and 2 will be randomized together but not blinded, while Group 3 will be randomized separately and will be blinded. With the implementation of Version 2.0, Groups 4 and 5 will be randomized together and will enroll simultaneously.

Participants

88 healthy, HIV–uninfected volunteers aged 18 to 50 years

Design

Multicenter randomized trial. Groups 1, 2, 4, and 5 are open-label and Group 3 is double-blinded and placebo-controlled.

Duration per participant

8 months of scheduled clinic visits

Estimated total study duration

18 months (includes enrollment and follow-up)

Investigational New Drug (IND) sponsor

DAIDS, NIAID, NIH, DHHS (Bethesda, Maryland, USA)

Study product provider

Vaccine Research Center/NIAID/NIH (Bethesda, MD)

FOR REVIEW ONLY

Core operations

HVTN Vaccine Leadership Group/Core Operations Center, Fred Hutchinson Cancer Research Center (FHCRC) (Seattle, Washington, USA)

Statistical and data management center (SDMC)

Statistical Center for HIV/AIDS Research and Prevention (SCHARP), FHCRC (Seattle, Washington, USA)

HIV diagnostic laboratory

University of Washington Virology Specialty Laboratory (UW-VSL) (Seattle, Washington, USA)

Endpoint assay laboratories

- NIAID Vaccine Immune T-Cell and Antibody Laboratory (NVITAL) (Gaithersburg, MD)
- FHCRC/University of Washington (Seattle, WA)
- Duke Human Vaccine Institute (DHVI), Duke University Medical Center (Durham, NC)
- Neutralizing Antibody Assay Laboratory (Duke-NAB), Duke University Medical Center (Durham, NC)

Study sites

HVTN Clinical Research Sites (HVTN CRSs) to be specified in the Site Announcement Memo

Safety monitoring

HVTN 104 PSRT; HVTN Safety Monitoring Board (SMB)

FOR REVIEW ONLY

3.1 Protocol Team

Protocol leadership

Chair	Kenneth Mayer<br>The Fenway Institute	*Statistician*	Yunda Huang<br>SCHARP, FHCRC
Cochair	Kelly Seaton<br>Duke Human Vaccine Institute	*Medical officer*	Mary Allen<br>DAIDS, NIAID
Protocol Team leader	Nicole Grunenberg<br>HVTN Core, FHCRC		

Other contributors to the original protocol

Core medical monitor	Nicole Grunenberg<br>HVTN Core, FHCRC	*Clinical trials manager*	Marianne Hansen<br>HVTN Core, FHCRC
Vaccine developer representative	Barney Graham<br>VRC	*SDMC project manager*	Lisa Sunner<br>SCHARP, FHCRC
Vaccine developer representative	Julie Ledgerwood<br>VRC	*HVTN program manager*	Gina Escamilla<br>SCHARP, FHCRC
Vaccine developer representative	Mary E. Enama<br>VRC	*Protocol development coordinator*	Ryan Jensen<br>HVTN Core, FHCRC
Laboratory Program representative	Eva Chung<br>HVTN Laboratory Program, FHCRC	*Community engagement unit representative*	Gail Broder<br>HVTN Core, FHCRC
Regulatory affairs	Janet Quade<br>HVTN Core, FHCRC	*Community educator/recruiter*	Samuel Cohen<br>Brigham and Women's Hospital
Clinic coordinator	Charlie Gregor<br>Fenway Community Health	*Technical editor*	Adi Ferrara<br>HVTN Core, FHCRC
Community Advisory Board (CAB) members	David Crawford<br>Chicago CAB	*Clinical safety specialist*	Jill Zeller<br>HVTN Core, FHCRC
	Steve Csipke<br>Boston CAB	*Statistical research associate*	Doug Grove<br>SCHARP, FHCRC
DAIDS protocol pharmacist	Scharla Estep<br>DAIDS, NIAID		

FOR REVIEW ONLY

4 Background

Although the global incidence of new HIV infections peaked in the mid-1990s, UNAIDS has reported that 2.5 million new HIV infections occurred last year, for a global total of more than 34 million people living with HIV [4]. The wider availability of antiretroviral (ARV) therapy, mother-to-child transmission prevention programs, and a diverse array of other prevention programs have all contributed to turning the tide of the epidemic, but the magnitude of new infections remains a major concern, warranting the need for the development of safe and effective preventive vaccines.

The search for a globally effective HIV vaccine, as well as elucidation of biomarkers predictive of vaccine efficacy, continues. Modest success in preventing HIV acquisition has been achieved by the RV144 trial in Thailand with 31% efficacy [5]. Neutralizing antibodies (nAbs) were not substantially elicited by this vaccine regimen and were not found to be a correlate of risk in this modestly effective vaccine regimen [6]. However, most licensed vaccines elicit protective nAbs that correlate with vaccine efficacy. For HIV, it is possible that multiple immune mechanisms will be needed to prevent acquisition. In nonhuman primate (NHP) models, the presence of nAbs has been shown to prevent SHIV acquisition [7]. However, to date HIV vaccines have not been successful in generating nAbs effective against a wide variety of infecting strains [8-10]. In recent years, research has made progress in the discovery of broad and potent nAbs found in the sera of chronically HIV infected donors [11-14]. Knowledge gained from such discoveries holds promise for the development of new immunogens capable of eliciting broadly nAbs (bnAbs) at titers which could be potentially effective in protection against HIV-1[15].

The Vaccine Research Center (VRC), NIAID, NIH has developed VRC01, a broadly neutralizing human mAbs which is targeted against the HIV-1 CD4 binding site [13]. This mAb was originally discovered in a participant infected with HIV-1 for more than 15 years who maintained viral control without use of ARV therapy [16]. By applying a novel method of isolating B cells that produce a specific antibody, and using recombinant DNA technology, the heavy and light chains encoding VRC01 were cloned and sequenced, allowing the synthetic production of codon-optimized genes encoding the variable region that was inserted into proprietary immunoglobulin G1 (IgG1) background sequences [13]. In the interim since the isolation of the VRC01 antibody, subsequent work evaluating longitudinal serum collected from HIV-1 infected individuals has indicated that although antibodies capable of binding to VRC01-like epitopes may be induced during HIV-1 infection, they occur in only a minority of HIV-infected individuals and may take years to develop [17].

The structure of VRC01 bound to HIV gp120 core has been determined. It binds to the HIV-1 gp120 envelope protein. VRC01 displays several unusual structural features. It is highly affinity-matured, has a disulfide link between complementarity-determining region (CDR) H1 and H3 and has a glycan in the variable (V) region of the light chain. However, none of these features appears to be required for binding affinity or neutralization [14]. VRC01 does not have an unusually long CDR-H3 region like some other HIV-1 nAbs. It is not self- or poly- reactive and lacks anti-phospholipid antibody activity [18]; these features are consistent with the hypothesis that VRC01 will be safe for human administration.

FOR REVIEW ONLY

By *in vitro* testing, VRC01 has a half-maximal inhibitory concentration (IC_{50}) of < 50 mcg/mL against 91% of primary isolates of various HIV-1 clades and < 1 mcg/mL against 72% of these isolates. Several proof-of concept studies have been conducted to determine whether the *in vitro* neutralization capabilities of VRC01 translate into the ability to protect NHPs from challenge with virulent chimeric simian-human immunodeficiency virus (SHIV), which contains the HIV envelope in an SIV background. Protection against a single high-dose SHIV-SF162P3 (a CCR5 tropic strain of HIV) rectal challenge was demonstrated at a 20 mg/kg dose level and partial protection at 5 mg/kg in Rhesus macaques. Protection against vaginal challenge was also demonstrated at 20 mg/kg dose level, given intravenously (IV) [NHP studies by J. Mascola, et al; personal communication]. As tested in the SHIV model in infant macaques, passive transfer of IgG1b12 nAbs was protective, supporting the use of neutralizing antibodies in perinatal settings [19]. In addition, VRC01 at 20 mg/kg protected infant macaques from an oral SHIV challenge [Nancy Haigwood, personal communication].

The clinical use of mAbs to prevent the establishment of viral infections has been previously demonstrated. Monthly injections of palivizumab, a mAb that blocks respiratory syncytial virus (RSV) binding to pulmonary epithelia were found to be safe and well-tolerated, and effective in protecting neonates and infants with underlying pulmonary disease from developing clinically significant RSV infection necessitating hospital admission [20].

4.1 Rationale for trial concept

The rationale for HVTN 104 has evolved during the course of its development and implementation. Initially, in Version 1.0 with Groups 1-3, this trial was intended to collect safety and VRC01 trough level data from multiple doses of VRC01 administered over 6 months in dosing regimens hypothesized to result in drug levels consistently within the protective range, based on preclinical data. These goals are in support of potential future efforts in HIV prevention in infants (PMTCT) and adults. Subsequently, further clarity on the potential and distinct contributions that VRC01 may allow for the HIV vaccine field have come to the forefront and drive the modification of HVTN 104 to Version 2, with the inclusion of Groups 4 and 5 as well as the addition of more mucosal sampling timepoints. In these new groups, rather than maintain a consistently high VRC01 level, the goal is to allow the bnAb levels (i.e. neutralization titers) to drop to levels that are hypothesized to be within a range potentially inducible by a vaccine. For these groups, optimizing inter-subject variability between dosing intervals while maintaining VRC01 neutralization titers estimated within an IC50 <1mcg/ml (see Section 4.7) is the goal.

VRC01 drug levels and functional activity in serum and mucosal fluids will be evaluated by using multiple assays. Assessment of VRC01 drug levels in serum for the primary endpoint analysis will use an anti-idiotype assay specific for VRC01 (described in Section 10.4.1). Secondary endpoint analyses will assess VRC01 binding to Env antigens using the validated Binding Antibody Multiplex Assay (BAMA) (also described in Section 10.4.1). Additionally, we plan to assess the functional activity of VRC01 using the validated TZM-bl neutralization assay (described in Section 10.5.2).

FOR REVIEW ONLY

Rationale for Version 1.0 (Groups 1-3):

VRC01 has successfully prevented SHIV acquisition in the NHP challenge protection model. The next step is testing the concept in humans. The VRC is preparing for a large scale international test-of-concept study in infants assessing prevention of mother-to-child transmission of HIV-1. The VRC is conducting initial first-in-human dose escalation studies for safety, tolerability and pharmacokinetic assessments in HIV infected (VRC 601) and HIV un-infected (VRC 602) adults. Upon confirmation of maximum dose acceptability, additional safety and pharmacokinetic (PK) data collection from multiple doses in a larger cohort of adults interrogating a range of dosing regimens is then needed to support future test of concept studies.

HVTN 104 will provide additional supportive data for planned and postulated uses of VRC01 for prevention of HIV acquisition. The primary goal is to further validate the safety and tolerability of VRC01 administered multiple times over 6 months. The data from HVTN 104 will help support the dosing schedule and rationale for a planned test of concept infant study; which will be a staged phase 1/2b, randomized, double-blind, placebo-controlled, multi-dose, international trial in breastfed infants born to HIV infected mothers. The first stage of the infant study will assess safety and tolerability and PK parameters of multiple dosing while the second stage will assess efficacy with a primary endpoint being the prevention of HIV transmission at 24 weeks of life. The preliminary dosing planned is monthly SC administration of 20mg/kg after an initial loading dose of 40mg/kg administered shortly after birth.

The NIH Vaccine Research Center's VRC 601 and 602 clinical trials will provide initial safety and pharmacokinetic data for 1 mg/kg, 5 mg/kg, 20mg/kg, and 40mg/kg IV doses as well as 5 mg/kg SC doses in a small number of adult participants. In adults, the SC dose evaluation is limited to 5 mg/kg because the volume of product that would be needed to be delivered for a dosage ≥ 20 mg/kg SC is too large to be accommodated by the SC route. HVTN 104 is being conducted in a larger cohort of adults and began when safety data from the maximum tolerated dose tested in VRC 602 was available. Group 1 in HVTN 104 is testing the same regimen proposed in the phase 1/2b infant study, but is being administered IV in adults. Groups 2 and 3 in HVTN 104 will also test alternate dosing regimens that may be considered for later studies in infants or adults.

Another goal of this study is to assess the VRC01 trough levels reached by administering different doses of VRC01 in different frequencies and routes. Based on the preclinical studies, achievement of trough serum levels of approximately 40 or 50 mcg/mL may be sufficient for HIV prevention. Assessing the kinetics of a higher dose administered less frequently (bimonthly) alongside a moderate dose administered more frequently (monthly) by IV is useful to determine if either or both schedules can achieve trough levels in this range. There are obvious logistical and operational advantages to less frequent dosing intervals if this can achieve trough levels hypothesized to provide protection, for any route method, but especially for IV. For this objective, we propose Group 1 to be dosed at 40 mg/kg IV at Month 0 and then 20 mg/kg at Months 1, 2, 3, 4, 5, and Group 2 to be dosed at 40 mg/kg at Months 0, 2, 4. Due to the expected long half-life for VRC01 in humans, a loading dose is anticipated to be required to allow trough levels to reach adequate levels earlier than without a loading dose. Therefore, a loading dose is planned for Groups 1 and 3. Since the maximum dose planned for testing in humans is 40mg/kg, a higher loading dose will not be administered for Group 2.

FOR REVIEW ONLY

In addition, evaluation of the kinetics of a smaller dosage of VRC01 given SC at more frequent intervals merits exploration. Studies on the kinetics of IV and SC administration of IgG immunotherapy in patients with immunodeficiency disorders have compared high doses given IV every 3 weeks to smaller doses given SC weekly. IVIG dosing results in high peak serum levels (up to 1000 mg/dL) that fall rapidly over few days and with a further slow decline from catabolism. However, use of smaller SC doses allowing a slow diffusion of IgG into the vasculature and lymphatics results in stable higher trough IgG serum levels which remain constant between consecutive SC IG infusions [21]. Both routes of administration display similar half-lives. Trough levels, rather than peak levels, are of greatest importance, rather than peak, for sufficient prevention of HIV acquisition during passive immunotherapy, and thus smaller doses given more frequently via the SC route may have the potential to result in sufficient and consistent trough levels as compared to higher IV doses given less frequently. For this objective, we are proposing a third active arm of 5mg/kg SC biweekly (after a loading dose of 40mg/kg IV).

VRC01 is formulated at 100 ± 10 mg/mL. Subcutaneous dosing in infants is the expected route of administration and is feasible at the doses planned as the volume administered will be sufficiently small to be administered in 1 or 2 SC injections. Due to the concentration of the VRC01, larger volumes are required for adult dosing at the higher doses; therefore IV administration will be used for the higher dosing in these initial studies.

Considering long term that SC dosing can be potentially self-administered (as it is with IgG immunotherapy), then biweekly SC dosing may be more accessible, less burdensome, less expensive, and preferred by patients (as it is for many IgG immunotherapy patients) than monthly or even bimonthly IV dosing. Reactogenicity has not been a significant concern with SCIG [21].

Rationale for Version 2.0 (Groups 4 and 5):

Major questions with respect to bnAbs and development of HIV vaccines include:

- What is the range of protection afforded by a bnAb that blocks the binding of HIV gp120 to the cellular CD4 binding site? What is the dynamic range in concentration of antibody and neutralizing activity associated with protection? Can lower levels of neutralization activity, including levels potentially inducible by an HIV vaccine, afford protection or does in vivo protection require only high concentrations of antibodies to HIV CD4 binding site?

- Are non-neutralizing effector functions also predictive of efficacy in addition to neutralizing activity? What are the kinetics and functional activities (non-neutralizing) that are seen at low neutralization titers for VRC01?

- What is the PK profile of VRC01 in relation to different levels of neutralization over time? Do compartment-specific differences in blood and mucosal fluids affect the neutralizing and non-neutralizing functions of VRC01? These levels will allow us to model the HIV acquisition rates through a wide dynamic range of neutralization levels for VRC01.

To answer these questions, it will be necessary to evaluate bnAbs in later phase trials, evaluating protection and HIV acquisition endpoints at varying antibody levels in at-risk populations. Neutralizing antibodies to HIV have been shown to protect against

FOR REVIEW ONLY

experimental challenge in the NHP SHIV model [22-29]. In these studies the degree of protection has varied with the neutralizing potency of the antibodies and with the dose, route, and sensitivity of the challenge stocks. Low antibody concentrations have in some instances been quite protective, especially against repeat low dose mucosal challenges [30,31]. Recent studies have suggested that VRC01 concentrations as low as ~1-2 mcg/ml can achieve sterilizing immunity (see Section 4.9.4 and [32-35]). By adding Groups 4 and 5 to the HVTN 104 protocol, the study team will be able to assess the levels of VRC01 in blood and mucosal secretions when administered intravenously at 10 mg/kg or 30 mg/kg. Therefore, Groups 4 and 5 will inform the dose-selection for a phase 2b correlates trial aiming to evaluate the efficacy of a bnAb in high risk adults and to establish markers of passive immunoprophylaxis that correlate with protection against HIV infection. By using lower doses every 2 months (10 mg/kg IV in Group 4 and 30 mg/kg IV in Group 5), these newly added groups are evaluating dosing regimens that are expected to result in lower trough levels with large inter-subject variability between dosing intervals (see Section 4.7), necessary to accomplish these correlates goals.

4.2 Mucosal specimen collection

Mucosal samples will be collected in order to assess the dynamics of transudation of VRC01 into mucosal secretions, where the protective effects of the mAb against founder virions would be expected to be most significant. Collection will include salivary and rectal secretions in men and women, the collection of semen in men and cervicovaginal secretions in women. The mucosal specimens will be collected on day 84, to assess early levels of VRC01 in the mucosal compartments, as well as at day 168 and day 224, which correspond to final trough and late timepoints. With Version 2.0, mucosal secretion sampling is being added at additional timepoints: 3, 14 and 21 days after the 2nd or last VRC01 dose administration (the 21 day collection timepoint for all groups except Group 3). These additional samplings will allow for better potential semi-quantitative analysis of serum:mucosal surface VRC01 levels and intra- as well as inter-individual variability for mucosal expression. Additionally, functional and neutralizing Ab activity within the mucosal secretions may potentially be assessed at differing VRC01 levels.

4.3 Anti-VRC01 antibodies

Production of anti-VRC01 antibodies may occur in some participants, yet are not expected to elicit hypersensitivity reactions. The package insert for Synagis® (palivizumab), the licensed mAb product most analogous to the intended clinical use of the VRC mAb [20], describes the incidence of anti-palivizumab antibody production following the fourth injection to be 1.1% in the placebo group and 0.7% in the active group in the single season clinical trial of 1502 children. In children receiving Synagis® for a second season, one of the fifty-six children had transient, low titer reactivity. This reactivity was not associated with adverse events or alteration in serum concentrations. Immunogenicity of Synagis® was also assessed in a trial involving another 379 children that compared liquid to lyophilized formulations and observed 0.3% incidence of anti-palivizumab antibodies. These data reflect the percentage of children whose test results were considered positive for antibodies to palivizumab using an enzyme-linked immunosorbent assay (ELISA) which has substantial limitations in detecting anti-palivizumab antibodies in the presence of palivizumab. Immunogenicity samples tested with the ELISA assay were likely to have contained palivizumab at levels that may have interfered with the detection of anti- palivizumab antibodies. An electrochemical

FOR REVIEW ONLY

luminescence (ECL) based immunogenicity assay, with a higher tolerance for palivizumab presence compared to the ELISA, was used to evaluate the presence of anti-palivizumab antibodies in subject samples from two additional clinical trials. The rates of anti-palivizumab antibody positive results in these trials were 1.1% and 1.5%. One objective of HVTN 104 is to assess for the presence of anti-VRC01 antibodies and if present, determine if they interfere with VRC01 levels. An electrochemical luminescence assay rather than ELISA will be used for the detection of anti-VRC01 antibodies (see Section 10.5.1).

4.4 VRC01: VRC-HIVMAB060-00-AB

VRC01 is a human mAb targeted against the HIV-1 CD4 binding site developed by VRC/NIAID/NIH. The bulk lot of the drug substance was manufactured under cGMP in a Chinese Hamster Ovary (CHO) cell line and the drug product vials were filled and labeled at the VRC, Vaccine Pilot Plant (Frederick, MD) operated by Leidos Biomedical Research, Inc. (formerly SAIC-Frederick), Frederick, MD. Each product vial contains 2.25 mL ± 0.10 mL volume at a concentration of 100 ± 10 mg/mL VRC01 in formulation buffer containing 25 mM Sodium Citrate, 50 mM Sodium Chloride, and 150 mM L-Arginine Hydrochloride at pH 5.8. VRC01 was produced using recombinant DNA technology. Briefly, using polymerase chain reaction (PCR) amplification and cloning of the heavy and light chain variable region genes, a mAb was initially isolated from a single B cell from an HIV-1 infected subject who displayed broadly neutralizing antibodies. VRC01 is an IgG1 antibody and is highly somatically mutated from the germ-line precursor. The heavy chain CDR3 region is 14 amino acids long, which is an average length relative to natural antibodies and the glycosylation pattern is derived from its production in a CHO cell line.

The potency and breadth of neutralization by VRC01 compared with b12 (mAb derived from an HIV-1 clade B-infected donor) were assessed on a comprehensive panel of viral envelope pseudoviruses. The panel of 190 viral strains represented the major circulating HIV-1 genetic subtypes (clades) and included viruses derived from acute and chronic stages of HIV-1 infection. The ability of VRC01 and b12 mAbs to neutralize such pseudoviruses is reported on the neutralization dendrograms in Figure 4-1 [13].

VRC01 has an IC50 of <50 mcg/mL against 91% of primary HIV-1 isolates and IC50 <1 mcg/mL against 72% of HIV-1 isolates tested *in vitro* Of Clade B and Clade C isolates, 95% and 97%, respectively, are sensitive *in vitro* at a VRC01 concentration of 50 mcg/ml, 94% and 96% respectively at 10 mcg/ml, and 80% and 82% respectively at 1 mcg/ml [13].

FOR REVIEW ONLY

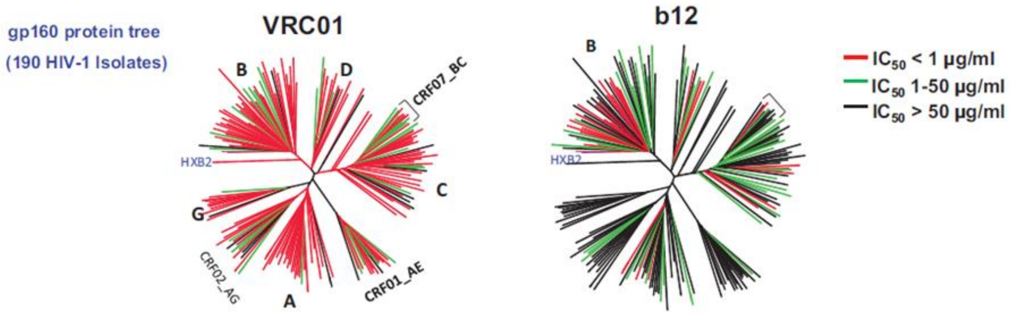

Virus clade	Number of viruses	IC_{50} < 50 μg/ml		IC_{50} < 1 μg/ml	
		VRC01	b12	VRC01	b12
A	22	100%	45%	95%	23%
B	49	96%	63%	80%	39%
C	38	87%	47%	66%	13%
D	8	88%	63%	50%	25%
CRF01_AE	18	89%	6%	61%	0%
CRF02_AG	16	81%	19%	56%	0%
G	10	90%	0%	90%	0%
CRF07_BC	11	100%	27%	45%	9%
Other	18	83%	33%	78%	6%
Total	190	91%	41%	72%	17%

Figure 4-1 Analysis of neutralization by VRC01 and b12 antibodies against a panel of 190 pseudoviruses representing the major circulating clades of HIV-1.

More details on VRC01 composition and manufacturing can be found in the IB.

4.5 Placebo for VRC01: VRC-PLAMAB068-00-AB

Placebo for VRC01 (VRC-PLAMAB068-00-AB) is a sterile, buffered aqueous solution of 25 mM Sodium Citrate, 50 mM Sodium Chloride, 150 mM L-Arginine Hydrochloride, 10% Dextran 40 (w/w), and 0.005% Polysorbate 80 (w/w) at pH 5.8. Placebo is supplied in 3 mL glass vials with 2.25 ± 0.10 mL fill volume. Vials contain a clear to colorless liquid with no visible particles.

More details on the placebo composition and manufacturing can be found in the IB.

4.6 Sodium chloride placebo

Sodium Chloride for Injection 0.9%, USP administered IV in 100 mL of normal saline (Sodium Chloride for Injection 0.9%, USP).

4.7 Trial design rationale

HVTN 104 will evaluate the safety profiles and trough levels of 5 different regimens for the IV and SC administration of VRC01. For adults, the advantage of the IV regimens is they allow administration of a larger volume of VRC01 to achieve the higher dosage levels that may be needed for prevention of HIV acquisition. However, an advantage of the SC regimens is the shorter infusion time, potential for reduced technical burden of a needle and syringe injection compared to IV product administration. This is important in

FOR REVIEW

a resource-constrained environment, as well as for product administration to infants. In the long term development of the product, a SC injection regimen allows potential for self-administration. Testing both IV and SC administered regimens of VRC01 is also important because each route may have particular advantages in different prevention indications. To prevent mother-to-child HIV transmission in breast feeding infants, repeated SC dosing might be beneficial to maintain consistent levels given frequent exposure to HIV, while for postexposure prophylaxis after a sexual assault in an adult, a single higher dose administration may be of most value.

The dose considerations for the evaluation of drug levels were informed primarily by prior studies with Synagis® (palivizumab), which is the only FDA-licensed mAb for which the target is a viral pathogen, but consideration was also given to other investigational mAbs directed at pathogens and preclinical studies with VRC01. For prevention of severe RSV, infants are treated with 15 mg/kg IM every month throughout the RSV season [20]. In the NHP preclinical studies with VRC01, dosing at 20 mg/kg was associated with prevention of SHIV infection (see Table 4-3 and the IB). Other investigational mAbs directed at pathogens that have been safely taken into efficacy trials include a mAb directed at *Clostridium difficile* toxin administered at a 10 mg/kg dosage [36] and a mAb directed at hepatitis C virus administered at a 50 mg/kg dosage [37]. Thus, the dose escalation plan in the first Phase 1 study (VRC 601) starts at least 10-fold lower than typical dosages for other mAb directed at pathogens and increases in a 5-fold, 4-fold, 2-fold dosage increase plan to the 40 mg/kg dose level, which is twice the level associated with VRC01 prevention of SHIV in an NHP model.

Importantly, with regard to the potential use of VRC01 to further reduce mother-to-child HIV transmission, the use of palivizumab for prevention of RSV in infants also provides a well-established record of dosage levels that are effective against a viral pathogen in infants and a model for safe evaluation of a mAb in an infant population.

In adult volunteers Synagis® had a pharmacokinetic profile similar to a human IgG1 antibody with regard to the volume of distribution and the half-life (mean 18 days). In pediatric patients, monthly intramuscular doses of 15 mg/kg achieved mean ±SD 30 day trough serum drug concentrations of 37 ± 21 mcg/mL after the first injection, 57 ± 41 mcg/mL after the second injection, 68 ± 51 mcg/mL after the third injection and 72 ± 50 mcg/mL after the fourth injection [20]. Thus, the selected monthly IV administration of 20 mg/kg or bimonthly IV administration of 40 mg/kg of VRC01 for evaluation in human clinical trials was selected based on an expectation that this would maintain trough concentrations at levels hypothesized to be in the effective range in humans.

Because SC administration of VRC01 is the planned route for the future infant efficacy trials which will utilize a placebo group, the placebo for VRC01 was developed only for SC administration to match the viscosity of VRC01 to prevent unblinding. The SC arm in HVTN 104 will include a placebo control group to enhance the safety and tolerability evaluations of this route and of both of these products. Normal saline is the placebo for IV administration because the higher doses of IV infused VRC01 will be diluted in normal saline. In order to be as parsimonious as possible, for Group 3 the randomization will be 5:1, i.e. 20 participants receiving VRC01 and 4 participants receiving the comparable placebo for VRC01. Since VRC01 levels will only be present in VRC01 recipients, a placebo group is not necessary for the PK goals of this trial.

However, a placebo group for each of the IV arms in this study is not considered necessary or particularly informative for safety assessments of the IV administrations.

FOR REVIEW ONLY

The VRC 601 and 602 trials (see Section 4.10) will provide the initial safety data in participants receiving IV infusions and this trial will serve to confirm those findings.

Rationale for dose selections for Groups 1-5:

Mathematical simulations were conducted to inform dosing regimen selection for Groups 1-5. These simulations used a standard two compartment PK model with parameter values estimated from the combined VRC 601 and VRC 602 data of 19 IV-infusion participants and 6 SC-injection participants. These simulations assumed linear pharmacokinetics and PK parameters proportional to body weight. These assumptions appear to be reasonable in the preliminary individual-subject PK modeling of the drug concentration data in VRC 601 and VRC 602. Figure 4-2 shows the predicted median drug concentration for dosing regimens evaluated in Groups 1-3. Figure 4-3 shows the predicted drug concentration levels (5th, 50th and 95th percentiles) for Group 4 dosing regimen (10 mg/kg IV every 8 weeks – no loading dose). These simulation results demonstrated that reasonable trough levels could be achieved in all five groups; the most sparse regimen with 10 mg/kg IV every 2 months (Group 4) has approximately 90% chance of reaching a trough level > 1 mcg/mL (Dr. Edmund Capparelli, personal communication). In support of Group 5, interim PK data collected after one or two infusions of 20 mg/kg IV from a small number of subjects in VRC 602 suggest that trough levels are maintained above 10 mcg/mL for more than 8 weeks (Dr. Barney Graham, personal communication and Figure 4-4). At 30 mg/kg IV every 8 weeks, the trough level is anticipated to be intermediate compared to 20 mg/kg IV and 40 IV mg/kg every 8 weeks and spares product relative to the highest dose.

With respect to the loading dose considerations, Groups 1-3 have included the same loading dose of 40 mg/kg IV. This was done in order to allow the VRC01 drug levels to reach steady state more quickly to reflect application of a bnAb in a preventive setting. However, for Groups 4 and 5, in which the purpose is to understand the kinetics of drug concentrations and corresponding neutralization titers in the adult population, a slower achievement to a certain trough level is allowable and therefore no loading dose is included in Groups 4 or 5. In addition, the effect of a loading dose is abrogated in dosing regimens with the longer 8 week dosing interval (in Groups 2, 4 and 5).

FOR REVIEW ONLY

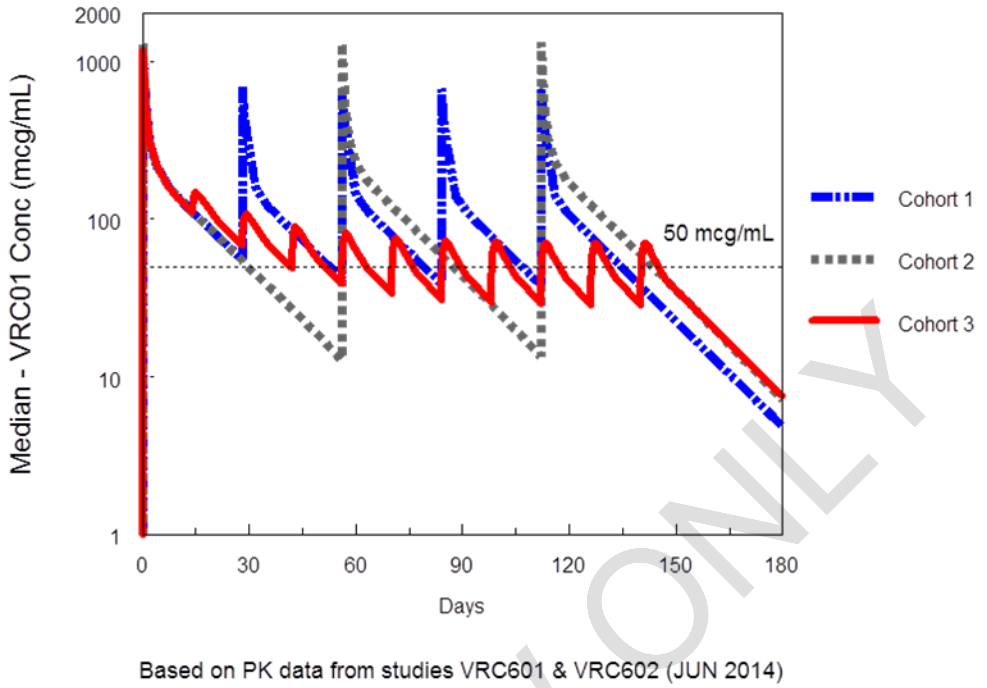

Figure 4-2: Simulated Model of VRC01 levels in Cohorts 1-3. Cohort 1 entails 20mg/kg IV infusion every 4 weeks with a loading dose of 40 mg/kg, Cohort 2 40mg/kg IV infusion every 8 weeks, and Cohort 3 5mg/kg SC injection every 2 weeks. Initial PK estimates based on the combined VRC 601 and VRC 602 data of 19 IV-infusion participants and 6 SC-injection participants were used in the simulations.

FOR REVIEW ONLY

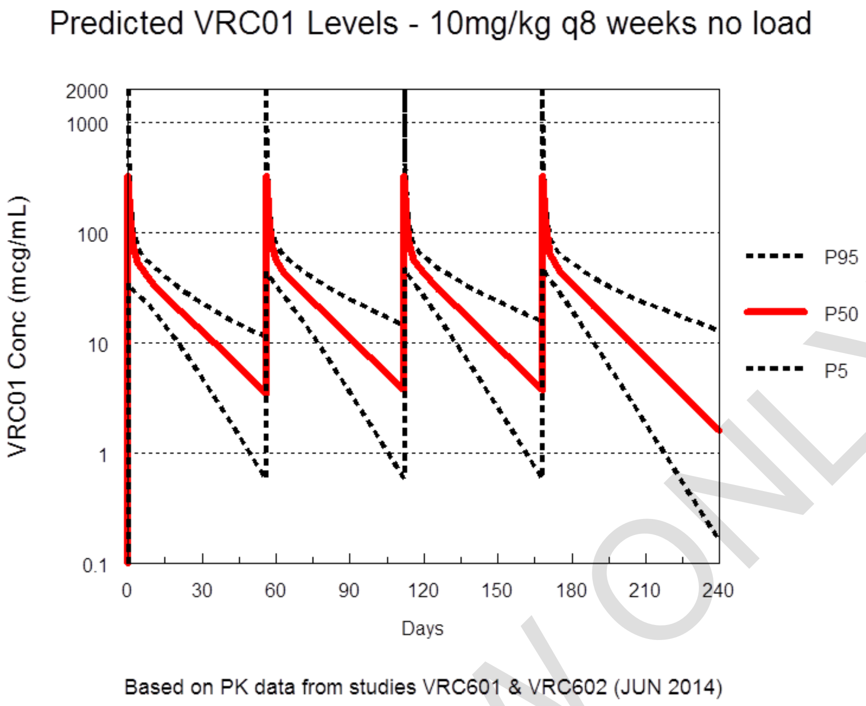

Figure 4-3: Simulated Model of VRC01 levels in Group 4. Group 4 entails 10mg/Kg IV infusion every 8 weeks with no loading dose. Initial PK estimates based on the combined VRC 601 and VRC 602 data of 19 IV-infusion participants were used in the simulations.

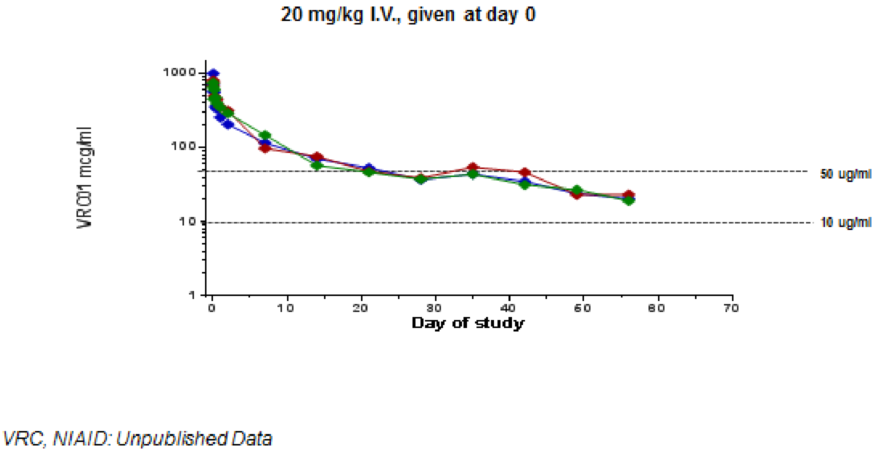

Figure 4-4: VRC01 levels from 3 participants in VRC 602 given a single dose of 20mg/kg IV at Day 0 and followed for 8 weeks.

As noted in Section 4.4, VRC01 has an IC50 of <50 mcg/mL against 91% of primary HIV-1 isolates and IC50 <1 mcg/mL against 72% of HIV-1 isolates tested in vitro. Also, the median IC50 against viruses neutralized with an IC50 is 0.32 for all clades, but varies somewhat by clade and by study (Table 4-1) (www.bnaber.org).

FOR REVIEW ONLY

Table 4-1: Median IC50 in mcg/ml against viruses neutralized with an IC50 <50 mcg/ml

Study	A	B	C	D	F	G	AE	AG	All clades
Walker2011	0.25*	0.24*	0.49*	0.44*	0.39*	0.1*	0.57*	0.12*	0.31*
Chuang2013	0.12*	0.35*	0.31*	0.47*	NA	NA	0.3*	0.2*	0.23*
Huang2012	0.09*	0.36*	0.26*	0.5*	NA	NA	0.33*	0.16*	0.24*
Scheid2011	0.09*	0.22*	0.05*	0.08*	NA	0.17*	0.27*	0.56*	0.09*
Liao2013	0.09*	0.37*	0.26*	NA	NA	NA	NA	NA	0.23*
Georgiev2013	0.12*	0.24*	0.27*	0.11*	NA	NA	0.33*	0.07*	0.2*
Wu2010	0.11*	0.35*	0.34*	0.57*	NA	0.21*	0.38*	0.14*	0.33*
Wu2011	0.11*	0.28*	0.32*	0.46*	NA	NA	0.37*	0.23*	0.25*

How the in vitro data correlates with in vivo VRC levels and nAb function is unknown, however, some limited NHP challenge data suggest that VRC01 serum levels between 75 mcg/ml and 10 mcg/ml would span a wide range of HIV virus sensitivities in vivo (John Mascola, VRC, personal communication). A dosing schedule of 10 mg/kg IV every 8 weeks is anticipated to result in a sufficiently variable range of VRC01 levels and corresponding nAb titers to inform dosing schedules for future phase 2b trials.

4.7.1 Dose and schedule

The proposed dosages for VRC 601 and 602 are based on the preclinical proof-of-concept studies performed to date with VRC01, as well as publicly available data from human clinical trials experience with other mAb developed for use in prevention or treatment of human diseases, with attention to those directed at viral pathogens. In particular, the licensed product Synagis® is directed against the viral pathogen, respiratory syncytial virus (RSV) [20,38], and the method and schedule by which it is administered to infants provide a model for the product development plan.

Group 1 comprises an IV infusion of 40 mg/kg VRC01 administered in 100 mL of normal saline at week 0, followed by IV infusions of 20 mg/kg of VRC01 administered in 100 mL of normal saline over 1 hour at weeks 4, 8, 12, 16, and 20.

Group 2 comprises an IV infusion of 40 mg/kg VRC01 administered in 100 mL of normal saline at weeks 0, 8, and 16.

Group 3 comprises an IV infusion of 40 mg/kg VRC01 or sodium chloride placebo administered in 100 mL of normal saline at week 0, followed by SC injection of 5 mg/kg VRC01 or placebo for VRC01 administered by injection every 2 weeks for 20 weeks.

Group 4 comprises an IV infusion of 10 mg/kg VRC01 administered in 100 mL of normal saline at weeks 0, 8, and 16.

Group 5 comprises an IV infusion of 30 mg/kg VRC01 administered in 100 mL of normal saline at weeks 0, 8, and 16.

FOR REVIEW ONLY

4.7.2 Choice of control

The placebo for VRC01, VRC-PLAMAB068-00-AB, is comprised of components that are generally recognized as safe (GRAS), but the fully constituted placebo for VRC01 has not been tested in humans prior to the initiation of the VRC 602 study. This study agent is intended for use only as the placebo for SC evaluation of VRC01 and was developed to match the viscosity characteristics of VRC01 when formulated as a 100 mg/mL product.

The sodium chloride placebo used for the first IV infusion timepoint in the Group 3 placebo recipients will contain Sodium Chloride for injection 0.9%, USP serving as an inert control.

4.8 Plans for future product development and testing

The data that will be generated from this study will help to establish the safety of VRC01 in HIV-uninfected men and women and will help to inform the decisions about appropriate dosing regimens for future studies of the use of VRC01 to prevent mother-to-child HIV transmission, design of future vaccine trials, or other immuno-prophylaxis indications. Ultimately, data from future passive immunization trials of highly selective mAbs will further inform future HIV vaccine development by determining if HIV-specific antibodies are capable of preventing HIV acquisition in humans, the levels or titers necessary for doing so and the quality of the antibodies that may be successful. In addition, research is ongoing to explore the design of increasingly specific immunogens strategically employed in a series of successive vaccinations in order to expeditiously mimic the development of broadly nAbs as observed in HIV chronic infection [39].

The collection of simultaneous blood and anogenital secretion samples will advance the understanding of the diffusion of VRC01 between compartments, which may be relevant for selection of specific vaccine dosing regimens designed to generate broadly nAbs. It is conceivable that VRC01 could be part of a combined non-vaccine prevention modality (eg, antiretroviral prophylaxis) and vaccine approach, which would provide additional protection against HIV until an effective host immune response against HIV is generated. Finally, it is anticipated that there will be enough product for a phase 2b study in high risk/high incidence adult populations, and there is interest within the HVTN to participate in this endeavor.

4.9 Preclinical studies

4.9.1 Preclinical toxicology study of VRC01 in rats

A repeat dose toxicity study of IV and SC administration and a single dose pharmacokinetics (PK) study was performed by SRI International (Menlo Park, CA) with VRC01 in male and female Sprague-Dawley rats in accordance with U.S. FDA "Good Laboratory Practice (GLP) for Nonclinical Laboratory Studies." This study was conducted with a pre-GMP pilot lot of VRC01 manufactured at smaller scale using a similar purification process to that of the GMP clinical grade drug product.

For the safety assessment, various doses of VRC01 (4 mg/kg, 40 mg/kg, or 400 mg/kg) or a comparable vehicle was administered by tail vein infusion on Days 1 and 8 to Groups 1

FOR REVIEW ONLY

through 4, respectively. An additional group (Group 5) received 40 mg/kg VRC01 via SC administration to the dorsal scapular region on Days 1 and 8. Each group contained 10 male and 10 female rats. Five animals per sex were sacrificed on Day 9, one day after the second administration, and the remaining animals were sacrificed on Day 30, 22 days after the second administration.

Results obtained showed that both routes of administration were well tolerated in the rats. All animals survived until their scheduled necropsy. No findings or changes were seen in clinical observation, body weight, food consumption, body temperature, infusion site irritation, hematology, coagulation, or organ weight evaluations that are attributed to administration of VRC01. VRC01 administration resulted in small, transient, dose-dependent increases in aspartate aminotransferase (AST) and alkaline phosphatase (ALP) on Day 9. By Day 30, AST values had returned to normal, and ALP values were returning to normal.

Other than red discoloration of the administration site in one male in the SC group on Day 9, there were no other gross necropsy observations attributable to VRC01 administration. There were no histopathology findings that were considered related to IV administration of VRC01. However, histopathology evaluation revealed sub-acute inflammation at the SC injection site on Day 9, one day after infusion in all 10 rats administered VRC01 SC; dermal inflammation was usually minimal or mild while subcutaneous inflammation was usually mild, moderate, or marked. By Day 30, this inflammation had completely resolved, and the SC dose site was normal in all rats.

The pre-specified IV dose studied in rats was 400 mg/kg and SC was 40 mg/kg, which will greatly exceed the dose levels in the adult clinical studies. A “no observed effect level” (NOEL) was not determined in this study because transient elevations of AST and ALP were observed on Day 9 after IV administration and transient inflammation at the dose site was observed on Day 9 after SC administration. Because the elevated AST and ALP levels were transient and minor and did not correlate with histopathology findings, the no observed adverse effect level (NOAEL) for VRC01 by the IV route of administration in rats was 400 mg/kg, the highest dose used in this study. The systemic NOAEL for the SC route of administration of VRC01 in rats was 40 mg/kg, the only SC dose level examined in this study.

For the PK analysis, a separate cohort of rats received VRC01 on Day 1 at 4 mg/kg followed by 40 mg/kg by the IV route of administration and at 40 mg/kg by the SC route of administration. VRC01 levels in serum were determined using an enzyme-linked immunosorbent assay (ELISA) with samples collected predose from each animal and from an additional 3 males and 3 females to provide untreated control serum. Blood was collected from 3 rats/sex/PK group for a total of 4–5 collections per PK animal at each of the following postdose time points: 1, 4, 8, 24, 48, and 72 hr and 7, 14, 21, and 29 days.

VRC01 administration by the IV route resulted in dose proportional exposure. The terminal elimination phase half-life was about 10 days, with clearance of approximately 20 mL/day/kg and volume of distribution that was about 0.28 l/kg, indicating that the drug was distributed primarily in the serum and eliminated slowly. VRC01 administration by the SC route resulted in mean peak serum levels at 7 days for male or 3 days for female animals. The maximum serum concentration and area under the concentration-time curve to the last time point values were lower when 40 mg/kg was administered by the SC route compared with the IV route. The bioavailability of 40 mg/kg VRC01 administered by the SC route was estimated to be 31.4% (males) and 42.3% (females).

FOR REVIEW ONLY

After the peak concentration of VRC01 was achieved in the SC group, the serum levels decreased much more rapidly from 7 to 14 days than they did in the IV groups, and VRC01 concentrations in the SC group were not quantifiable at time points after 14 days. These data indicate that clearance of VRC01 in rats was markedly enhanced when it was administered by the SC route. The development of anti-drug antibodies that contribute to an increased rate of clearance is often observed in preclinical safety studies of protein-based test articles when they are not tested in the species of origin. Although immunogenicity was not examined in this study, the presence of such antibodies might have possibly contributed to the increased rate of clearance of VRC01 after SC administration that was observed in this study [40,41].

4.9.2 Tissue Cross Reactivity GLP Study of VRC01 with Human Tissues In Vitro

A tissue cross-reactivity study of VRC01 using normal adult and neonatal human tissues in vitro (Testing Facility Study No. A255-12) was performed by Charles River Laboratories (Reno, NV) in accordance with U.S. FDA "Good Laboratory Practice for Nonclinical Laboratory Studies" (GLP). The tissue panels used as the test system for this *in vitro* cross-reactivity study included all of the tissues on the "Suggested list of human tissues to be used for immunohistochemical or cytochemical investigations of cross reactivity of monoclonal antibodies" in Annex I of the "European Medicines Agency Guideline on Development, Production, Characterization and Specifications for Monoclonal Antibodies and Related Product, Adopted by the Committee for Medicinal Products for Human Use on December 18, 2008" and all of the tissues recommended in the FDA/Center for Biologics Evaluation and Research "Points to Consider in the Manufacture and Testing of Monoclonal Antibody Products for Human Use (February 28, 1997)." In addition, the tissue cross-reactivity study used additional neonate/infant tissues suggested by the FDA to support future trials in infants.

To determine the cross-reactivity of VRC01 binding, VRC01 was applied to cryosections from a full panel of tissues from normal human adults and a limited panel of human neonatal tissues, immunohistochemically detected using a biotinylated rabbit anti-human IgG secondary antibody, and binding visualized with a streptavidin-horseradish peroxidase complex and a diaminobenzidine chromogen substrate. VRC01 binding was evaluated at concentrations of 5 and 50 mcg/mL.

Specific VRC01 staining was not observed in any normal adult human or neonatal human tissues evaluated. Therefore, *in vitro* evaluation of cross-reactivity in tissue specimens did not identify potential tissue sites or organ systems to more thoroughly evaluate in subsequent preclinical studies, and it supports the possible future use of VRC01 in humans.

4.9.3 Other Toxicity Studies

Several *in vitro* studies were conducted to assess for the demonstration of antibody activity against self-antigens by VRC01. Several anti-HIV neutralizing mAbs will cross-react to lipid or nuclear antigens or Hep-2 cells [18,42]. Anti-lipid binding activity is understandable when considering that the HIV-1 gp41 protein is membrane-spanning and the epitopes (MPER: Membrane-Proximal External Region) recognized by some mAbs (e.g. 4E10 and 2F5) are membrane-proximal and likely extend into the membrane itself. Therefore, the ability (or lack thereof) of VRC01 to cross-react with lipids was assessed in collaboration with Dr. Barton Haynes of Duke University. Binding of antibody to cardiolipin was assessed in a luminescent assay, expressed in relative units. VRC01 was

FOR REVIEW ONLY

compared to 4E10, an anti-gp41 mAb known to bind to cardiolipin and nuclear antigens, and Synagis®, a licensed anti-respiratory syncytial virus antibody used as a negative control. Synagis® is included because it is the licensed mAb product most analogous to the intended clinical use of the VRC mAb [20].

Individual studies are summarized in Table 4-2. Unlike other anti-HIV neutralizing mAbs, VRC01 does not react to phospholipids or anti-nuclear antigens or Hep-2 cells. Additional details are provided in the IB.

Table 4-2: *In Vitro* Preclinical Safety Studies

Study Purpose	Study Outcome
Assessment of anti-phospholipid reactivity	VRC01 does not react to phospholipids
Assessment of anti-nuclear antigen reactivity	VRC01 does not react with nuclear antigens
Assessment of anti-phospholipid characteristics by impact on activated partial thromboplastin time (aPTT)	VRC01 does not impact aPTT by binding phospholipids
Assessment of Binding to a Human Cell Line by Immunohistochemistry	Fluorescently labeled VRC01 does not bind Hep-2 cells

4.9.4 Nonhuman Primate (NHP) Studies of VRC01

Several non-GLP studies of VRC01 have been completed in NHP to assess for plasma and secretion concentrations and for preclinical evidence of potential efficacy for prevention of HIV infection. Table 4-3 is a brief summary of the studies performed and supports the plan to evaluate up to 40 mg/kg dose administered IV as a dose range of potential interest for a preventive indication. The current assay being used to detect VRC01 in serum has a lower limit of detection in the range of 1.8-2 mcg/mL. Detectable concentrations of VRC01 were measured in vaginal, rectal, nasal and saliva samples after IV administration of 20 mg/kg of VRC01. Concentrations of VRC01 were lower in mucosal samples after IV administration of 5 mg/kg of VRC01. Please refer to the VRC01 IB for more details.

FOR REVIEW ONLY

Table 4-3 Preclinical proof-of-concept studies performed with VRC01 mAb in NHP

Study Purpose	Study Outcome
Demonstration of plasma and secretion concentrations of VRC01 given intravenously at two dose levels in male rhesus macaques	Kinetics of decay of VRC01 administered IV at 5 mg/kg and 20 mg/kg in plasma, nasal and rectal secretions, and saliva established
Demonstration of plasma and secretion concentrations of VRC01 given by IV or SC routes in female rhesus macaques	Kinetics of decay of 40 mg/kg of VRC01 given IV or SC in plasma, rectal, vaginal and nasal secretions established
Demonstration of challenge-protection against intrarectal high-dose SHIV SF162P3 in male rhesus macaques	100% protection from challenge demonstrated at 20 mg/kg dose administered IV
Demonstration of challenge-protection against intravaginal high-dose SHIV SF162P3 in female rhesus macaques	100% protection from challenge demonstrated at 20 mg/kg dose administered IV
Demonstration of challenge-protection against intrarectal high-dose SHIV Ba-L in rhesus male macaques	100% protection from challenge demonstrated at 20 mg/kg dose administered IV

As shown in Table 4-4, Pegu et al demonstrated challenge-protection against intrarectal and intravaginal high-dose SHIV SF162P3 and SHIV BalP4 in rhesus macaques. Partial protection was still observed at the lowest dose evaluated (0.3 mg/kg) [33].

Table 4-4 Preclinical proof-of-concept studies performed with VRC01 mAb in NHP

VRC01 Dose	# of doses	Plasma levels day of challenge	Challenge timepoint	SHIV	Route	Rate of infection (% protected)
20 mg/kg IV	Xs 1	79.2 +/- 2.3 mcg/mL	2 days post dose	SF162P3	Rectal	0/4 (100%)
20 mg/kg IV	Xs 1	64.6 +/- 7.0 mcg/mL	2 days post dose	SF162P3	Vaginal	0/4 (100%)
*5 mg/kg IV	Xs 1	21.4 +/- 0.9 mcg/mL	2 days post dose	SF162P3	Rectal	2/4 (50%)
20 mg/kg IV	Xs 1	60.9 +/- 2.4 mcg/mL	2 days post dose	BalP4	Rectal	0/6 (100%)
5 mg/kg IV	Xs 1	22.2 +/- 1.4 mcg/mL	2 days post dose	BalP4	Rectal	0/6 (100%)
0.3 mg/kg IV	Xs 1	1.3 +/- 0.1 mcg/mL	2 days post dose	BalP4	Rectal	6/10 (40%)

* Data from this challenge-protection group has not yet been published (personal communication from A. Pegu)

SHIV BaL is a more neutralization sensitive strain used in the rectal challenge study and showed partial efficacy at dosing of 0.3 mg/kg and serum VRC01 levels of 1-2 mcg/ml. SHIV SF162P3 is a more neutralization resistant strain, and results of that study indicated that a higher dose and serum level of VRC01 is needed to completely protect NHP from a rectal challenge (Table 4-4). While the *in vitro* neutralization data and the NHP challenge studies are intriguing, the applicability of these results remains yet to be determined in humans.

FOR REVIEW ONLY

4.10 Clinical studies

DAIDS is the sponsor of the investigational new drug application (IND) to evaluate the potential clinical uses of VRC01. VRC01's first evaluations in humans began in September 2013. The VRC01 initial development plan is directed towards an intended indication of prevention of HIV-1 infection through maternal transmission at birth or during breastfeeding. The safety, tolerability, dose effect, and PK of the VRC01 are being assessed in phase 1 trials in HIV-1 infected adults (in VRC 601) and in healthy HIV-uninfected adults (in VRC 602) prior to evaluating the safety in infants in the US and internationally. Once the initial safety and PK data have been collected from these smaller dose escalation studies in adults, collection of additional safety data in larger numbers of adults from repeat doses will be used to support the infant studies.

4.10.1 VRC 601

VRC 601 (NCT01950325) titled, "*A Phase 1, Open-Label, Dose-Escalation Study of the Safety and Pharmacokinetics of a Human Monoclonal Antibody, VRC-HIVMAB060-00-AB (VRC01), with Broad HIV-1 Neutralizing Activity, Administered Intravenously or Subcutaneously to HIV-Infected Adults*."

VRC 601 is the first study of the VRC01 mAb in HIV-infected participants. It is a dose-escalation study to examine safety, tolerability, dose, PK, and anti-antibody immune responses. The hypothesis is that VRC01 will be safe for administration to HIV-1-infected adults by the IV and SC routes and will not elicit hypersensitivity reactions. A secondary hypothesis is that VRC01 will be detectable in human sera and mucosal secretions with a definable half-life.

As shown in Table 4-5, there are 4 dose escalation groups for IV administration and 1 group for SC administration at 5 mg/kg planned for the study. During the dose escalation part of the study, each VRC01 infusion was administered in an inpatient unit and followed by intensive collection of samples for pharmacokinetic (PK) analysis. The study plan includes clinical blood tests for safety, clinician assessment of local reactions, solicitation of systemic symptoms for 3 days after each administration, HIV viral load, CD4, and blood samples to assess whether any human anti-VRC01 antibody is induced. In Groups 2, 3, 4 and 5, subjects who agree may have oral and rectal fluid samples collected and women may also have cervical fluid samples collected to determine if VRC01 is detectable in these mucosal samples.

FOR REVIEW ONLY

Table 4-5 VRC 601 study schema

VRC 601 Dose Groups		VRC01 Administration Schedule	
Group	No. of evaluable subjects*	Day 0***	Week 4
1	at least 3	1 mg/kg IV	1 mg/kg IV
2	at least 3	5 mg/kg IV	5 mg/kg IV
3	at least 3	5 mg/kg SC	5 mg/kg SC
4	at least 3	20 mg/kg IV	20 mg/kg IV
5	at least 3	40 mg/kg IV	40 mg/kg IV
Total	15-30**	IV doses administered in 100 mL of normal saline over 30 to 60 mins.<br>SC doses administered in the minimum volume at 15 mL/hr.<br>*Only subjects who begin infusion are evaluable.<br>**The final enrollment may be greater than 3 evaluable subjects per group.<br>***Viremic subjects will receive only the Day 0 dose.	

VRC 601 is a single site study at the NIH Clinical Center, Bethesda, Maryland. The first infusion at 1 mg/kg IV was administered in the VRC 601 study on September 30, 2013. The first 40 mg/kg IV administration in this study occurred May 12, 2014.

4.10.2 VRC 602

VRC 602 (NCT01993706) titled, "*A Phase 1 Dose-Escalation Study of the Safety and Pharmacokinetics of a Human Monoclonal Antibody, VRC-HIVMAB060-00-AB (VRC01), Administered Intravenously or Subcutaneously to Healthy Adults.*"

VRC 602 is the first study in healthy, HIV uninfected, adults of the VRC01 mAb. It is a dose-escalation study to examine safety, tolerability, dose, and pharmacokinetics of VRC01. The hypothesis is that VRC01 will be safe for administration to healthy adults by the IV and SC routes and will not elicit hypersensitivity reactions. A secondary hypothesis is that VRC01 will be detectable in human sera and mucosal secretions with a definable half-life. The SC route evaluation will be placebo-controlled and conducted in a single-blind manner to evaluate safety and tolerability of VRC01 and placebo (VRC-PLAMAB068-00-AB).

As shown in Table 4-6, there are 3 open-label, dose escalation groups (Groups 1, 2, and 3) for IV administration and 1 double-blinded, placebo-controlled group (Group 4) for SC administration. Enrollment started with subject randomization to Groups 1 and 4 in a 1:2 ratio. Within Group 4, subjects are randomized to SC infusions of VRC01 or placebo in a 1:1 ratio. After establishing the tolerability of the initial SC dose administered by a slow infusion with a controlled rate pump, the VRC 602 protocol was amended to allow the option of SC administration by needle and syringe injection with dividing the volume, as needed, into 2 or 3 SC injection sites. The study includes clinical blood tests for safety, clinical assessment of local reactions, solicitation of systemic symptoms for 3 days after each administration, and blood samples to assess whether any human anti-VRC01 antibody is induced. In all groups, when the subject agrees, oral and rectal fluid samples are obtained and women may also have cervical fluid samples collected to determine if VRC01 is detectable in these mucosal samples.

FOR REVIEW ONLY

Table 4-6 VRC 602 study schema

VRC 602<br>VRC01 Administration Schedule				
**Group**	**Dose**	**N<br>Day 0**	**N<br>Week 4**	**N**
1	5 mg/kg IV	5	5	5
2	20 mg/kg IV	8	5	8
3	40 mg/kg IV	5	5	5
4A	5 mg/kg SC	5	5	5
4B	Placebo SC	5	4	5
VRC01 recipients		23	20	23

IV doses administered in 100 mL of normal saline over 1 hr.
First SC dose administered at about 15 mL/hr via SC infusion pump; subject option for second dose administration (Week 4) by direct SC injection with needle and syringe.
Group 2 received 1 or 2 doses
23 subjects received VRC01 at different dosage levels and 5 subjects received placebo administered SC.

VRC 602 is a single site study at the NIH Clinical Center, Bethesda, Maryland. The first infusion at 5 mg/kg IV was administered in the VRC 602 study on December 9, 2013. The first infusion at 40 mg/kg IV in this study was administered March 4, 2014. The final VRC01 administration was August 27, 2014.

The dose escalation to 40 mg/kg IV in the VRC 601 and VRC 602 studies was completed in a similar timeframe. As of January 9, 2015, cumulatively there have been 86 product administrations in 49 subjects in these two trials; including 21 subjects in VRC 601and 28 subjects in VRC 602. Of these 49 individuals, 9 subjects completed the one dose schedule, and of 40 subjects on a two dose schedule, 37 received two doses and 3 discontinued after one dose. Of the 3 subjects discontinued from study product administration, 1 was lost to follow-up, 1 was unable to comply with the study schedule and 1 experienced an unrelated adverse event (Streptococcal pharyngitis) that precluded timely administration.

Data from both HIV-infected and HIV-uninfected adults informs the questions of how to dose VRC01 to maintain a blood level in a range thought to be associated with a biological effect and to assess the safety of repeated exposure to VRC01. Both the IV and SC routes of administration are being evaluated. Ultimately, the best route for clinical use may depend upon the age of the recipient (adult or infant), stage of product development, formulation and important considerations related to volume needed and maintenance of a target VRC01 blood level considered to be in the therapeutic range.

4.10.3 Current status of HVTN 104

The first participant enrolled in HVTN 104 on September 9, 2014. As of January 20, 2015, a total of 42 participants (27 in Groups 1 and 2 and 15 in Group 3) have been enrolled across 6 sites in the US. The study product has been well tolerated with most participants experiencing no or mild reactions and there have been no SAEs.

FOR REVIEW ONLY

Two participants in Group 3 (VRC01 or placebo) had mild symptoms resulting in study product discontinuation. One participant had a mild itchy rash in several places a few days after the injection considered related to study product. The rash lasted a few hours and went away on its own without any other symptoms. The other participant experienced a tight feeling in the chest with wheezing upon auscultation that started shortly after the injection and went away on its own and was considered related to study product.

4.10.4 Safety summary of VRC01

Cumulatively, across all studies as of January 9, 2015, there were no expedited safety reports to the FDA or study safety pauses for adverse events and, no reactions during the VRC01 or placebo product administration that resulted in an incomplete administration.

VRC01 SC administrations are sometimes associated with mild local reactions during the infusions, including pruritus (itchiness), redness and swelling, which resolve within a few minutes to a few hours after the administration is completed. The largest diameter for erythema or swelling events observed during infusions ranged up to about 5 cm.

Solicited local and systemic signs and symptoms following administration of VRC01 or placebo are generally none to mild. Less than 20% of subjects reported any moderate or greater solicited reactions after any product administration.

Adverse events attributed to study product administration for which study product administration was discontinued included one subject with chest discomfort and one subject with rash.

Other adverse events attributed to study product administration have included AST, ALT and creatinine elevation, decreased neutrophil count and pruritus at the administration site. The mild or moderate elevated transaminases were reported in about 20% of HIV-infected subjects (in association with ARVs, as well as, strenuous exercise and/or alcohol intake), and infrequently in HIV-uninfected subjects. These laboratory changes and pruritus events resolved and did not require discontinuation of study product administration.

Overall, VRC01 administration in the dose range from 1 to 40 mg/kg IV and at 5 mg/kg SC have been assessed as well-tolerated and safe for further evaluation.

4.10.5 Future studies of VRC01

The data from HVTN 104 will help inform the dosing schedule and rationale for several studies including a phase 2b trial exploring the range of protection from HIV infection by a bnAb in high risk adults. In addition, a trial to assess the safety of VRC01 administered to infants born to HIV-infected mothers in the US is being planned by the IMPAACT network. Another study of VRC01 is being planned by USMHRP in Thai participants who are acutely-infected with HIV. An efficacy trial evaluating VRC01 in infants born to HIV-infected African women is planned assuming that early studies of VRC01 demonstrate an adequate safety profile.

FOR REVIEW ONLY

4.11 Potential risks of study products and administration

There is limited human experience with administration of the VRC01. VRC 601 and VRC 602 will evaluate the study agent prior to the start of HVTN 104.

In a preclinical study performed in rats, there was a small dose-dependent, but transient, increase in aspartate aminotransferase (AST) and alkaline phosphatase (ALP), but not in alanine aminotransferase (ALT) following IV administration. In rats, there were no histopathology findings following IV administration, but following SC administration there was minimal to marked dermal and SC irritation at the SC infusion site.

Thus far in VRC 601, there have been no SAEs or other safety concerns. Administration of mAb may have a risk of immune reactions such as acute anaphylaxis, serum sickness and the generation of antibodies, however, these reactions are rare and more often associated with mAb targeted to human proteins or with the use of murine mAbs which would have a risk of human anti-mouse antibodies [43]. In this regard, as VRC01 is targeted to a viral antigen and is a human mAb, it is expected to have a low risk of such side effects.

Typically, the side effects of mAbs are mild but may include fever, flushing, chills, rigors, nausea, vomiting, pain, headache, dizziness, shortness of breath, bronchospasm, hypotension, hypertension, pruritus, rash, urticaria, angioedema, diarrhea, tachycardia or chest pain. Clinical use of mAbs that are targeted to cytokines or antigens associated with human cells may be associated with an increased risk of infections [43]; however, this is not expected to be a risk for a mAb targeted to a viral antigen.

It is known from published experience with human mAb directed against the cell surface targets on lymphocytes, that infusion of a mAb may be associated with cytokine release, causing a reaction known as “cytokine release syndrome” (CRS) [44]. Most infusion-related events occur within the first 24 hours after beginning administration. Severe reactions; such as anaphylaxis, angioedema, bronchospasm, hypotension, and hypoxia, are infrequent and more often associated with mAbs targeted to human proteins or when a non-human mAb, such as a murine mAb, is used [43]. Specifically, with regard to CRS reactions, these most commonly occur within the first few hours of beginning the infusion and are more common with the first mAb infusion received. This is because the cytokine release is associated with lysis of the cells targeted by the mAb and the burden of target cells is greatest at the time of the first mAb treatment. With licensed therapeutic mAbs, CRS is managed by temporarily stopping the infusion, administration of histamine blockers and restarting the infusion at a slower rate [45].

Delayed allergic reactions to a mAb may include a serum sickness type of reaction, which is characterized by urticaria, fever, lymph node enlargement, and joint pains. These symptoms may not appear until several days after the exposure to the mAb and is noted to be more common with chimeric types of mAb [43].

There are several FDA-licensed mAb for which reactions related to the rate of infusion have been described. Some symptoms may be treated by slowing or stopping the infusion. Supportive treatment may also be indicated for some signs and symptoms.

The published experience with mAb administered by the SC route is limited but there is experience with the SC route of administration of immunoglobulins, such as Hizentra® (CSL Behring LLC, Kankakee, IL), 20% immune serum globulin, for patients with

FOR REVIEW ONLY

primary immunodeficiency diseases. Comparison of the safety and PK of IVIG to subcutaneous immunoglobulin (SCIG) has been reported. The SC route of administration has a good safety profile. Tissue reactions are common but usually mild and tend to decline over time with repeated administrations [46]. The package insert for Hizentra® notes that the most common adverse reactions (observed in ≥5% of study subjects) in clinical trials were local reactions (such as swelling, redness, heat, pain and itching at the infusion site), headache, vomiting, pain, and fatigue.

VRC01 is an antibody to an HIV protein. Therefore, it may be theoretically possible for a standard antibody-based HIV diagnostic test to detect VRC01 for a short time period postinfusion or postinjection. However, based on HVTN laboratory testing of HIV uninfected plasma samples spiked with VRC01 in the range of concentrations that mimic the likely range of concentrations (200 mcg/mL, 50 mcg/mL, and 1 mcg/mL) that may be observed in a clinical trial, VRC01 did not cause a positive test result in several standard antibody-based HIV-1/2 diagnostic tests used in the US.

Risks of Blood Drawing: Blood drawing may cause pain and bruising and may, infrequently, cause a feeling of lightheadedness or fainting. Rarely, it may cause infection at the site where the blood is taken. Problems from use of an IV for blood drawing are generally mild and may include pain, bruising, minor swelling or bleeding at the IV site and rarely, infection, vein irritation (called phlebitis), or blood clot.

Risks of Mucosal Sample Collection: Collection of samples by swabs and wicks by rubbing them over the mucosal surfaces can cause momentary discomfort and, in some cases, minor bleeding.

Risks of Intravenous Infusion: The placement of an intravenous catheter can allow for the development of bacteremia because of the contact between the catheter and unsterile skin when it is inserted. This will be prevented through careful decontamination of local skin prior to catheter placement and through the use of infection control practices during infusion. Product contamination will be prevented by the use of aseptic technique in the pharmacy and universal precautions during product administration.

FOR REVIEW ONLY

5 Objectives and endpoints

5.1 Primary objectives and endpoints

Primary objective 1:

To evaluate the safety and tolerability of VRC01 administered IV and SC at multiple timepoints.

Primary endpoints 1:

Local and systemic reactogenicity signs and symptoms, laboratory measures of safety, and AEs and SAEs.

Early discontinuation of infusions and reason(s) for discontinuation and early study termination.

Primary objective 2 (Groups 1-3):

To evaluate the serum levels of VRC01 at Month 6 administered IV and SC in 3 different regimens.

Primary endpoint 2:

Serum concentration of VRC01 in Groups 1-3 at Month 6.

Primary objective 3 (Groups 4 and 5):

To evaluate the serum levels of VRC01 at 2 timepoints after each IV administration.

Primary endpoint 3:

Serum concentration of VRC01 28 and 56 days after each IV administration in Groups 4 and 5.

5.2 Secondary objectives and endpoints

Secondary objective 1:

To evaluate the kinetics of *in vitro* neutralization in serum of a single VRC01 sensitive virus isolate

Secondary endpoint 1:

Magnitude of serum neutralization of a single VRC01 sensitive virus isolate as measured in the TZMbl assay at multiple timepoints

Secondary objective 2:

FOR REVIEW ONLY

To further assess the serum levels of VRC01.

Secondary endpoint 2:

Serum concentration of VRC01 in each group at multiple timepoints.

Secondary objective 3:

To determine whether anti-idiotypic antibody (AIA) can be detected and whether there is a correlation of VRC01 levels and AIA levels in serum.

Secondary endpoint 3:

Serum concentration of anti-VRC01 antibodies in each group at multiple timepoints compared to corresponding VRC01 levels.

Secondary objective 4:

To determine if measurable levels of VRC01 can be found in genital, rectal, and oral secretions.

Secondary endpoint 4:

Mucosal levels of VRC01 in each group at multiple timepoints.

Secondary objective 5:

To evaluate the kinetics of *in vitro* neutralization in mucosal secretions of a single VRC01 sensitive virus isolate.

Secondary endpoint 5:

Magnitude of neutralization in genital, rectal, and oral secretions of a single VRC01 sensitive virus isolate as measured in the TZMbl assay at multiple timepoints.

Secondary objective 6:

To assess binding of VRC01 to multiple Env proteins.

Secondary endpoint 6:

Binding antibody multiplex assay will be used to assess VRC01 binding in serum and genital, rectal, and oral secretions to multiple Env proteins in each group at multiple timepoints.

5.3 Exploratory objectives

Exploratory objective 1:

FOR REVIEW ONLY

To evaluate the breadth of *in vitro* neutralization in the serum and mucosal secretions if detected with the primary VRC01 sensitive isolate.

Exploratory objective 2:

To compare the serum levels of VRC01 between groups at multiple timepoints

Exploratory objective 3:

To evaluate additional functional humoral activities in serum and genital, rectal, and oral secretions.

Exploratory objective 4:

To evaluate the effect of participant characteristics (i.e. sex and BMI) on serum levels of VRC01.

Exploratory objective 5:

To describe the acceptability of study product administration on all study participants

FOR REVIEW ONLY

6 Statistical considerations

6.1 Accrual and sample size calculations

Recruitment will target enrolling 88 healthy, HIV-uninfected adult participants into five groups. Groups 1, 2, 4, and 5 entail IV administration of VRC01 on two different schedules with a randomization ratio of 1:1 for n=20 participants in Groups 1 and 2 and n=12 participants in Groups 4 and 5. Group 3 entails one initial IV administration followed by multiple SC administrations of VRC01 (n=20) or placebo (n=4) with a treatment:control randomization ratio of 5:1. Sites will be encouraged to enroll at least approximately 40% of each sex within Groups 1 and 2 combined, Groups 4 and 5 combined, and separately within Group 3. Groups 1 and 2 are randomized together, Groups 4 and 5 are randomized together. Group 3 undergoes a separate randomization process from the other groups because Group 3 participants will need to specifically agree to participate in a more intense dosing and visit schedule.

Since enrollment is concurrent with receiving the first complete or incomplete study infusion, all participants will provide some safety data. However, for PK analyses, it is possible that data may be missing for various reasons, such as participants terminating from the study early or problems in shipping specimens. For this reason, the sample size calculations in Section 6.1.2 account for 10% enrolled participants having missing data for the primary lab data endpoint.

6.1.1 Sample size calculations for safety

The goal of the safety evaluation for HVTN 104 is to identify safety concerns associated with product administration. The ability of the study to detect SAEs can be expressed by the true event rate above which at least 1 SAE would likely be observed and the true event rate below which no events would likely be observed. Specifically, for an active arm of n=12, there is a 90% chance of observing at least 1 event if the true rate of such an event is 17.46% or more; and there is a 90% chance of observing no events if the true rate is 0.88% or less. For each active arm of n=20 , there is a 90% chance of observing at least 1 event if the true rate of such an event is 10.87% or more; and there is a 90% chance of observing no events if the true rate is 0.52% or less. For active IV administration arms combined (n=64), there is a 90% chance of observing at least 1 event if the true rate of such an event is 3.53% or more; and there is a 90% chance of observing no events if the true rate is 0.17% or less. For all active arms combined (n=84), there is a 90% chance of observing at least 1 event if the true rate of such an event is 2.70 % or more; and there is a 90% chance of observing no events if the true rate is 0.13 % or less. As a reference, in HVTN vaccine trials from December 2000 through December 2012, about 4% of participants who received placebos experienced an SAE.

Probabilities of observing 0, 1 or more, and 2 or more events among single arms or combined arms of sizes 12, 20, 64, and 84 are presented in Table 6-1 for a range of possible true AE or SAE rates. These calculations provide a more complete picture of the sensitivity of this study design to identify potential safety problems with the product.

FOR REVIEW ONLY

Table 6-1 Probability of observing 0 events, 1 or more events, and 2 or more events, among arms of size 12, 20, 64, and 84, for a range of true event rates

True event rate (%)	Arm Size	0 Events	1+ Events	2+ Events
1	12	0.89	0.11	0.01
	20	0.82	0.18	0.02
	64	0.53	0.47	0.13
	84	0.43	0.57	0.21
4	12	0.61	0.39	0.08
	20	0.44	0.56	0.19
	64	0.07	0.93	0.73
	84	0.03	0.97	0.85
10	12	0.28	0.72	0.34
	20	0.12	0.88	0.61
	64	<0.01	>0.99	0.99
	84	<0.01	>0.99	>0.99
20	12	0.07	0.93	0.73
	20	0.01	0.99	0.93
	64	<0.01	>0.99	>0.99
	84	<0.01	>0.99	>0.99
30	12	0.01	0.99	0.91
	20	<0.01	>0.99	0.99
	64	<0.01	>0.99	>0.99
	84	<0.01	>0.99	>0.99

An alternative way of describing the statistical properties of the study design is in terms of the 95% confidence interval for the true rate of an adverse event based on the observed data. Table 6-2 shows the 2-sided 95% confidence intervals for the probability of an event based on a particular observed rate. Calculations are done using the score test method [47]. If none of 12 participants in an active arm (groups 4 or 5) experience a safety event, the 95% 2-sided upper confidence bound for the true rate of such events in the total vaccinated population is 24.25%. If none of 20 participants in an active arm (Groups 1, 2, or 3) experience a safety event, the 95% 2-sided upper confidence bound for the true rate of such events is 16.11%. If none of the 64 combined participants in Groups 1, 2, 4, and 5 experience a safety event, the 95% 2-sided upper confidence bound for the true rate of such events is 5.66%. For the total 84 participants in the active arms, the 95% 2-sided upper confidence bound for this rate is 4.37%.

FOR REVIEW ONLY

Table 6-2 Two-sided 95% confidence intervals based on observing a particular rate of safety endpoints for single or combined arms of size 12, 20, 64, and 84

Observed event rate	Confidence interval (%)
0/12	(0.00, 24.25)
1/12	(0.43, 35.39)
2/12	(4.70, 44.80)
0/20	(0.00, 16.11)
1/20	(0.26, 23.61)
2/20	(2.79, 30.10)
0/64	(0.00, 5.66)
1/64	(0.08, 8.33)
2/64	(0.86, 10.70)
0/84	(0.00, 4.37)
1/84	(0.06, 6.44)
2/84	(0.66, 8.27)

6.1.2 Sample size calculations for drug levels of VRC01

The main goal of HVTN 104 regarding PK drug levels involves a preliminary estimation of the mean *trough drug concentration* after the administration series for each active arm. The precision with which the true concentration level can be estimated from the observed data depends on the standard deviation of measurements and the sample size. Two-sided 95% confidence intervals for the true mean drug level based on different observed average drug concentration levels in the active arms of size 12 and 20 are shown in Table 6-3, assuming a normal distribution for the log-transformed levels. The confidence intervals in the table are based on arm sizes of 10 and 18, which accounting for an assumed 10% loss of data from the original arms sizes. For example, if an average drug concentration of ln(15) and a standard deviation of 1.0 are observed based on the log-transformed drug levels among 18 participants, the 95% 2-sided upper confidence bound for the true mean drug concentration level is 24 mcg/mL. These calculations assume an approximate normal distribution for the mean of log-transformed drug concentration levels. The assumed mean and standard deviation are based on limited unpublished data from a study of a similar mAb built on the same isotype construct (personal communications). Since no individual-level data are available, first order Taylor expansions are used to approximate the mean and variance of the log-transformed data based on the reported mean and variance of the original scale data. Specifically, the natural log of the mean of the original scale data approximates the mean of the natural log-transformed data; the squared ratio between the variance and mean of the original scale data approximates the variance of the natural log-transformed data.

FOR REVIEW ONLY

Table 6-3 Two-sided 95% confidence intervals based on observing a particular average drug level in participants in any of the active arms (n=10, n=18)

Observed average $\log_e$ drug level ($\log_e$ mcg/mL)	Standard Deviation of $\log_e$ drug level ($\log_e$ mcg/mL)	Confidence interval (mcg/mL) (N=10)	Confidence interval (mcg/mL) (N=18)
ln(15) = 2.71	0.5	(11, 20)	(12, 19)
ln(30) = 3.40		(22, 41)	(24, 38)
ln(60) = 4.09		(44, 82)	(48, 76)
ln(120) = 4.79		(88, 164)	(95, 151)
ln(15) = 2.71	1.0	(8, 28)	(9, 24)
ln(30) = 3.40		(16, 56)	(19, 48)
ln(60) = 4.09		(32, 112)	(38, 95)
ln(120) = 4.79		(65, 223)	(76, 190)

6.2 Randomization

The randomization sequence will be obtained by computer-generated random numbers and provided to each HVTN CRS through the SDMC via a Web-based randomization system. Groups 1-3 will be enrolled simultaneously. With the implementation of Version 2.0, Groups 4 and 5 will enroll simultaneously. The randomization will be done in blocks to ensure balance across Groups 1 and 2, across Groups 4 and 5, and within Group 3. Because the schedule for Group 3 is more intensive, participants will be given the choice of being randomized in Group 3 or, separately, into one of Groups 1, 2, 4, or 5. At each institution, the pharmacist with primary responsibility for dispensing study products is charged with maintaining security of the treatment assignments.

6.3 Blinding

Participants and site staff (except for site pharmacists) will be unblinded as to participant treatment assignments between Groups 1, 2, 4, and 5, but blinded as to treatment assignment (active vs. placebo) for Group 3. Study product assignments are accessible to those HVTN CRS pharmacists, DAIDS protocol pharmacists and contract monitors, and SDMC staff who are required to know this information in order to ensure proper trial conduct. Any discussion of study product assignment between pharmacy staff and any other HVTN CRS staff is prohibited. The HVTN SMB members also are unblinded to treatment assignment in order to conduct review of trial safety.

When a participant leaves the trial prior to study completion, the participant will be told he or she must wait until all participants are unblinded to learn his or her treatment assignment.

Emergency unblinding decisions will be made by the site investigator. If time permits, the HVTN 104 PSRT should be consulted before emergency unblinding occurs.

6.4 Statistical analysis

This section describes the final study analysis, unblinded as to treatment arm assignment. All data from enrolled participants will be analyzed according to the initial randomization assignment regardless of how many infusions they received. The analysis is a modified intent-to-treat analysis in that individuals who are randomized but not enrolled do not

FOR REVIEW ONLY

contribute data and hence are excluded. Because of blinding and the brief length of time between randomization and enrollment—typically no more than 4 working days—very few such individuals are expected.

Analyses will be performed using SAS and R. Other software may be used to perform additional exploratory pharmacokinetics analyses..

No formal multiple comparison adjustments will be employed for multiple safety endpoints.

6.4.1 Analysis variables

The analysis variables consist of baseline participant characteristic, safety, and laboratory measurements for primary- and secondary-objective analyses.

6.4.2 Baseline comparability

Treatment arms will be compared for baseline participant characteristics using descriptive statistics.

6.4.3 Safety/tolerability analysis

Since enrollment is concurrent with receiving the first infusion, all participants will have received at least 1 infusion and therefore will provide some safety data.

6.4.3.1 Reactogenicity

The number and percentage of participants experiencing each type of reactogenicity sign or symptom will be tabulated by severity and treatment arm and the percentages displayed graphically by arm. For a given sign or symptom, each participant's reactogenicity will be counted once under the maximum severity for all infusion visits. In addition, to the individual types of events, the maximum severity of local pain or tenderness, induration or erythema, and of systemic symptoms will be calculated. Wilcoxon rank-sum tests will be used to test for differences in severity between Groups 1 and 2.

6.4.3.2 AEs and SAEs

AEs will be summarized using MedDRA System Organ Class and preferred terms. Tables will show by treatment arm the number and percentage of participants experiencing an AE within a System Organ Class or within preferred term category by severity or by relationship to study product. For the calculations in these tables, a participant with multiple AEs within a category will be counted once under the maximum severity or the strongest recorded causal relationship to study product. Formal statistical testing comparing arms is not planned since interpretation of differences must rely heavily upon clinical judgment.

A listing of SAEs reported to the DAIDS Regulatory Support Center (RSC) Safety Office will provide details of the events including severity, relationship to study product, time between onset and last infusion, and number of infusions received.

FOR REVIEW ONLY

6.4.3.3 Local laboratory values

Boxplots of local laboratory values will be generated for baseline values and for values measured during the course of the study by treatment arm and visit. Each boxplot will show the first quartile, the median, and the third quartile. Outliers (values outside the boxplot) will also be plotted. If appropriate, horizontal lines representing boundaries for abnormal values will be plotted.

For each local laboratory measure, summary statistics will be presented by treatment arm and time point, as well as changes from baseline for postenrollment values. In addition, the number (percentage) of participants with local laboratory values recorded as meeting Grade 1 AE criteria or above as specified in the DAIDS AE Grading Table (see Section 9.9) will be tabulated by treatment arm for each postinfusion time point. Reportable clinical laboratory abnormalities without an associated clinical diagnosis will be included in the tabulation of AEs described above.

6.4.3.4 Reasons for discontinuation of study product administration and early study termination

The number and percentage of participants who discontinue study product administration and who terminate the study early will be tabulated by reason and treatment arm.

6.4.3.5 Acceptability of study product or procedure

Acceptability of study product administration and injection procedures will be tabulated by reason and treatment arm.

6.4.4 Analysis of Antibody-level Endpoints

6.4.4.1 General approach

For the statistical analysis of endpoints, data from enrolled participants will be used according to the initial randomization assignment regardless of how many infusions they received. Additional analyses may be performed, limited to participants who received all scheduled infusions per protocol. Assay results that are unreliable, from specimens collected outside of the visit window, or from HIV-infected participants postinfection will be excluded. Since the exact date of HIV infection is unknown, any assay data from blood draws 4 weeks prior to an infected participant's last seronegative sample and thereafter may be excluded. If an HIV-infected participant does not have a seronegative sample postenrollment, then all data from that participant may be excluded from the analysis.

For continuous assay data (eg, serum concentration of VRC01), graphical and tabular summaries of the distributions by treatment arm and timepoint will be made. The difference of continuous assay data at the primary time-points of interest (Month 6) between groups will be tested with a nonparametric Wilcoxon rank sum test if the data are not normally distributed and with a 2-sample t-test if the data appear to be normally distributed. An appropriate data transformation (e.g., log transformation) may be applied prior to testing to better satisfy assumptions of symmetry and homoscedasticity (constant variance).Inference from these analyses would be limited by the small sample sizes of the groups.

More sophisticated analyses of drug-level data and other assay data collected over time employing repeated measures methodology (for example, nonlinear mixed effects models

FOR REVIEW ONLY

or generalized estimating equations) may be utilized to incorporate outcome responses over several timepoints and to account for subject heterogeneity. In addition, non-compartment PK analysis will be performed to estimate PK parameters from individual time-concentration curves. All statistical tests will be 2-sided and will be considered statistically significant if $p \leq 0.05$.

For qualitative assay variables (e.g., positive or negative), the analyses will be performed by tabulating the frequency of positive responses for each assay by group at each time-point at which an assessment is performed. Crude response rates will be presented with their corresponding 95% confidence interval estimates calculated using the score test method [47] .

For the analysis of correlation between two continuous assay variables over time, graphical summary and tabular summary of the sample correlation at each given time-point will be made. Cross-correlation of the two variables with different time-lags may also be calculated and visually displayed if there are at least 10 participants with no missing data over time from both variables. More details of the statistical analysis approaches will be described in a separate Statistical Analysis Plan document.

Based upon previous AIDS Vaccine Evaluation Group (AVEG) and HVTN trials, missing 10% of research samples' results for a specific assay is common due to study participants terminating from the study early, problems in shipping specimens, or low cell viability of processed peripheral blood mononuclear cells (PBMCs). To achieve unbiased statistical estimation and inferences with nonparametric tests and generalized linear models fit by generalized estimating equation (GEE) methods, missing data need to be missing completely at random (MCAR). MCAR assumes that the probability of an observation being missing does not depend upon the observed responses or upon any unobserved covariates but may depend upon covariates included in the model (eg, missing more among whites than nonwhites). When missing data are minimal (specifically if no more than 20% of participants are missing any values), then nonparametric tests and GEE methods will be used, because violations of the MCAR assumption will have little impact on the estimates and hypothesis tests. These models will include as covariates all available baseline predictors of the missing outcomes.

If a substantial amount of antibody-level data are missing (at least 1 value missing from more than 20% of participants), then using the methods that require the MCAR assumption may give misleading results. In this situation, analyses of the endpoints at a specific timepoint will be performed using parametric generalized linear models fit by maximum likelihood. These methods provide unbiased estimation and inferences under the parametric modeling assumptions and the assumption that the missing data are missing at random (MAR). MAR assumes that the probability of an observation being missing may depend upon the observed responses and upon observed covariates, but not upon any unobserved factors. Generalized linear models for response rates will use a binomial error distribution and for quantitative endpoints, a normal error distribution. For assessing repeated immunogenicity measurement, linear mixed effects models will be used. If the immunological outcomes are left- and/or right- censored, then the linear mixed effects models of Hughes [48] will be used, because they accommodate the censoring. In addition, secondary analyses of repeated immunogenicity measurements may be done using weighted GEE [49] methods, which are valid under MAR. All of the models described above will include as covariates all available baseline predictors of the missing outcomes.

FOR REVIEW ONLY

6.4.5 Analyses prior to end of scheduled follow-up visits

Any analyses conducted prior to the end of the scheduled follow-up visits should not compromise the integrity of the trial in terms of participant retention or safety or immunogenicity endpoint assessments. In particular, early unblinded analyses by treatment assignment require careful consideration and should be made available on a need to know basis only.

6.4.5.1 Safety

During the course of the trial, unblinded analyses of safety data will be prepared approximately every 4 months during the main study, as defined in Section 3, for review by the SMB. Ad hoc safety reports may also be prepared for SMB review at the request of the HVTN 104 PSRT. The HVTN leadership must approve any other requests for unblinded safety data prior to the end of the scheduled follow-up visits.

6.4.5.2 Anti-VRC01 and other Laboratory Assessments

For Group 3, an unblinded statistical analysis by treatment assignment of a primary laboratory endpoint may be performed when all participants have completed the corresponding visit and data are available for analysis from at least 80% of these participants. Similarly, an unblinded statistical analysis by treatment assignment of a secondary or exploratory endpoint may be performed when all participants have completed the corresponding visit and data are available for analysis from at least 80% of these participants. The Laboratory Program will review the analysis report prior to distribution to the protocol chairs, DAIDS, vaccine developer, and other key HVTN members and investigators. Distribution of reports will be limited to those with a need to know for the purpose of informing future trial-related decisions. The HVTN leadership must approve any other requests for HVTN laboratory data analyses prior to the end of the scheduled follow-up visits.

FOR REVIEW ONLY

7 Selection and withdrawal of participants

Participants will be healthy, HIV–uninfected (seronegative) adults who comprehend the purpose of the study and have provided written informed consent. Volunteers will be recruited and screened; those determined to be eligible, based on the inclusion and exclusion criteria, will be enrolled in the study. Final eligibility determination will depend on results of laboratory tests, medical history, physical examinations, and answers to self-administered and/or interview questions.

Investigators should always use good clinical judgment in considering a volunteer's overall fitness for trial participation. Some volunteers may not be appropriate for enrollment even if they meet all inclusion/exclusion criteria. Medical, psychiatric, occupational, or other conditions may make evaluation of safety and/or immunogenicity difficult, and some volunteers may be poor candidates for retention.

Determination of eligibility, taking into account all inclusion and exclusion criteria, must be made within 56 days prior to enrollment unless otherwise noted in sections 7.1 and 7.2.

7.1 Inclusion criteria

General and Demographic Criteria

1. **Age** of 18 to 50 years

2. **Weight** ≥ 53 kg and ≤ 115 kg

3. **Access to a participating HVTN CRS** and willingness to be followed for the planned duration of the study

4. Ability and willingness to provide **informed consent**

5. **Assessment of understanding**: volunteer demonstrates understanding of this study; completes a questionnaire prior to first infusion with verbal demonstration of understanding of all questionnaire items answered incorrectly

6. **Agrees not to enroll in another study** of an investigational research agent until completion of the last study visit

7. **Good general health** as shown by medical history, physical exam, and screening laboratory tests

HIV-Related Criteria:

8. Willingness to receive **HIV test results**

9. Willingness to discuss **HIV infection risks**, amenable to **HIV risk reduction counseling**, and committed to maintaining behavior consistent with low risk of HIV exposure through the last required protocol clinic visit

FOR REVIEW ONLY

10. Assessed by the clinic staff as being at **"low risk" for HIV infection** and committed to maintaining behavior consistent with low risk of HIV exposure through the last required protocol clinic visit

Laboratory Inclusion Values

Hemogram/CBC

11. **Hemoglobin** ≥ 11.0 g/dL for volunteers who were born female, ≥ 13.0 g/dL for volunteers who were born male

12. **White blood cell count** = 2,500 to 12,000 cells/mm^3

13. **Total lymphocyte count** ≥ 800 cells/mm^3

14. **Remaining differential** either within institutional normal range or with site physician approval

15. **Platelets** = 125,000 to 550,000/mm^3

Chemistry

16. **Chemistry panel**: ALT, AST, and alkaline phosphatase < 1.25 times the institutional upper limit of normal; creatinine ≤ institutional upper limit of normal.

Virology

17. **Negative HIV-1 and -2 blood test**: US volunteers must have a negative FDA-approved enzyme immunoassay (EIA).

18. **Negative Hepatitis B surface antigen (HBsAg)**

19. **Negative anti-Hepatitis C virus antibodies (anti-HCV)**, or negative HCV polymerase chain reaction (PCR) if the anti-HCV is positive

Urine

20. **Normal urine**:

    - Negative urine glucose, and
    - Negative or trace urine protein, and
    - Negative or trace urine hemoglobin (if trace hemoglobin is present on dipstick, a microscopic urinalysis with red blood cells levels within institutional normal range).

Reproductive Status

21. **Volunteers who were born female**: negative serum or urine beta human chorionic gonadotropin (β-HCG) pregnancy test performed prior to infusion on the day of initial infusion. Persons who are not of reproductive potential due to having undergone total

FOR REVIEW ONLY

hysterectomy with bilateral oophorectomy (verified by medical records), are not required to undergo pregnancy testing.

22. **Reproductive status**: A volunteer who was born female must:

- Agree to consistently use effective contraception (see Appendix A) for sexual activity that could lead to pregnancy from at least 21 days prior to enrollment through the last required protocol clinic visit. Effective contraception is defined as using any of the following methods:
  - Condoms (male or female) with or without a spermicide,
  - Diaphragm or cervical cap with spermicide,
  - Intrauterine device (IUD),
  - Hormonal contraception,
  - Any other contraceptive method approved by the HVTN 104 PSRT,
  - Successful vasectomy in the male partner (considered successful if a volunteer reports that a male partner has [1] documentation of azoospermia by microscopy, or [2] a vasectomy more than 2 years ago with no resultant pregnancy despite sexual activity postvasectomy);
- Or not be of reproductive potential, such as having reached menopause (no menses for 1 year) or having undergone hysterectomy, bilateral oophorectomy, or tubal ligation;
- Or be sexually abstinent.

23. **Volunteers who were born female must also agree not to seek pregnancy through alternative methods**, such as artificial insemination or in vitro fertilization until after the last required protocol clinic visit

7.2 Exclusion criteria

General

1. **Blood products** received within 120 days before first infusion, unless eligibility for earlier enrollment is determined by the HVTN 104 PSRT
2. **Investigational research agents** received within 30 days before first infusion
3. **Intent to participate in another study** of an investigational research agent during the planned duration of the HVTN 104 study
4. **Pregnant or breastfeeding**

FOR REVIEW ONLY

Vaccines and other Injections

5. **HIV vaccine(s)** received in a prior HIV vaccine trial. For volunteers who have received control/placebo in an HIV vaccine trial, the HVTN 104 PSRT will determine eligibility on a case-by-case basis.

6. **Non-HIV experimental vaccine(s) received within the last 6 months** in a prior vaccine trial. Exceptions may be made for some vaccines and vaccine trials. For volunteers who have received an experimental vaccine(s) less than 6 months ago, eligibility for enrollment will be determined by the HVTN 104 PSRT on a case-by-case basis.

7. **Live attenuated vaccines** other than influenza vaccine received within 10 days before first infusion and with no evidence of residual inflammation; or scheduled within 10 days after first infusion (eg, measles, mumps, and rubella [MMR]; oral polio vaccine [OPV]; varicella; yellow fever).

8. **Previous receipt of humanized or human mAbs** whether licensed or investigational**.**

Immune System

9. **Immunosuppressive medications** received within 30 days before first infusion. (Not excluded: [1] corticosteroid nasal spray; [2] inhaled corticosteroids; [3] topical corticosteroids for mild, uncomplicated dermatitis; or [4] a single course of oral/parenteral corticosteroids at doses < 2 mg/kg/day and length of therapy < 11 days with completion at least 30 days prior to enrollment.

10. **Serious adverse reactions to vaccines or to vaccine components** including history of anaphylaxis and related symptoms such as hives, respiratory difficulty, angioedema, and/or abdominal pain. (Not excluded: a volunteer who had a nonanaphylactic adverse reaction to pertussis vaccine as a child.)

11. **Immunoglobulin** received within 90 days before first infusion, unless eligibility for earlier enrollment is determined by the HVTN 104 PSRT.

12. **Autoimmune disease** (Not excluded: Volunteer with mild, stable and uncomplicated autoimmune disease that does not require immunosuppressive medication and that, in the judgment of the site investigator, is likely not subject to exacerbation and likely not to complicate reactogenicity and AE assessments)

13. **Immunodeficiency**

Clinically significant medical conditions

14. **Untreated or incompletely treated syphilis infection**

15. **Clinically significant medical condition**, physical examination findings, clinically significant abnormal laboratory results, or past medical history with clinically significant implications for current health. A clinically significant condition or process includes but is not limited to:

    - A process that would affect the immune response,

FOR REVIEW ONLY

- A process that would require medication that affects the immune response,
- Any contraindication to repeated infusions or blood draws,
- A condition that requires active medical intervention or monitoring to avert grave danger to the volunteer's health or well-being during the study period,
- A condition or process for which signs or symptoms could be confused with reactions to vaccine, or
- Any condition specifically listed among the exclusion criteria below.

16. **Any medical, psychiatric, occupational, or other condition** that, in the judgment of the investigator, would interfere with, or serve as a contraindication to, protocol adherence, assessment of safety or reactogenicity, or a volunteer's ability to give informed consent

17. **Psychiatric condition that precludes compliance with the protocol**. Specifically excluded are persons with psychoses within the past 3 years, ongoing risk for suicide, or history of suicide attempt or gesture within the past 3 years.

18. **Current anti-tuberculosis (TB) prophylaxis or therapy**

19. **Asthma** other than mild, well-controlled asthma. (Symptoms of asthma severity as defined in the most recent National Asthma Education and Prevention Program (NAEPP) Expert Panel report).

    Exclude a volunteer who:

    - Uses a short-acting rescue inhaler (typically a beta 2 agonist) daily, or
    - Uses moderate/high dose inhaled corticosteroids, or
    - In the past year has either of the following:
        - Greater than 1 exacerbation of symptoms treated with oral/parenteral corticosteroids;
        - Needed emergency care, urgent care, hospitalization, or intubation for asthma.

20. **Diabetes mellitus** type 1 or type 2, including cases controlled with diet alone. (Not excluded: history of isolated gestational diabetes.)

21. **Hypertension**:

    - If a person has been found to have elevated blood pressure or hypertension during screening or previously, exclude for blood pressure that is not well controlled. Well-controlled blood pressure is defined as consistently ≤ 140 mm Hg systolic and ≤ 90 mm Hg diastolic, with or without medication, with only isolated, brief instances of higher readings, which must be ≤ 150 mm Hg systolic and ≤ 100 mm Hg diastolic. For these volunteers, blood pressure must be ≤ 140 mm Hg systolic and ≤ 90 mm Hg diastolic at enrollment.

FOR REVIEW ONLY

- If a person has NOT been found to have elevated blood pressure or hypertension during screening or previously, exclude for systolic blood pressure ≥ 150 mm Hg at enrollment or diastolic blood pressure ≥ 100 mm Hg at enrollment.

22. **Bleeding disorder** diagnosed by a doctor (eg, factor deficiency, coagulopathy, or platelet disorder requiring special precautions)

23. **Malignancy** (Not excluded: Volunteer who has had malignancy excised surgically and who, in the investigator's estimation, has a reasonable assurance of sustained cure, or who is unlikely to experience recurrence of malignancy during the period of the study)

24. **Seizure disorder:** History of seizure(s) within past three years. Also exclude if volunteer has used medications in order to prevent or treat seizure(s) at any time within the past 3 years.

25. **Asplenia**: any condition resulting in the absence of a functional spleen

26. History of hereditary **angioedema**, acquired angioedema, or idiopathic angioedema.

7.3 Participant departure from the study product administration schedule or withdrawal

This section concerns an individual participant's departure from the study product administration schedule. Pause rules for the trial as a whole are described in Section 11.3.

7.3.1 Delaying study product administration for a participant

Under certain circumstances, a participant's scheduled study product administration will be delayed. The factors to be considered in such a decision include but are not limited to the following:

- Within 10 days prior to any study product administration
  - Receipt of live attenuated vaccines other than influenza vaccine. The delay may be reduced if residual inflammation from receiving the vaccine has resolved sooner
- Within 14 days prior to any study product administration
  - Receipt of systemic glucocorticoids (eg, prednisone or other glucocorticoid) or other immunomodulators (other than nonsteroidal anti-inflammatory drugs [NSAIDs])
- Prestudy product administration abnormal vital signs or clinical symptoms that may mask assessment of a study product reaction.
- Intercurrent illness that is not expected to resolve prior to the next scheduled study product administration which is assessed by the site principal investigator (or designee) to require delay or withdrawal from the study product administration schedule. The investigator may consult the HVTN 104 PSRT.

FOR REVIEW ONLY

Study product administration should not be administered outside the visit window period specified in the HVTN 104 Study Specific Procedures.

In order to avoid study product administration delays and missed study product administrations, participants who plan to receive live attenuated licensed vaccines other than influenza vaccine, or systemic glucocorticoids should be counseled to schedule receipt of these substances, when possible, outside the intervals indicated above. The effects of these substances on safety and immunogenicity assessments and their interactions with study products are unknown.

7.3.2 Participant departure from study product administration schedule

Every effort should be made to follow the study product administration schedule per the protocol. If a participant misses a study product administration and the visit window period for the study product administration has passed, that study product administration cannot be given. The participant should be asked to continue study visits. The participant should resume the study product administration schedule with the next study product administration unless there are circumstances that require further delay or permanent discontinuation of study product administration (see Sections 7.3.1 and 7.3.3).

7.3.3 Discontinuing study product administration for a participant

Under certain circumstances, an individual participant's study product administrations will be permanently discontinued. Specific events that will result in stopping a participant's study product administration schedule include:

- Co-enrollment in a study with an investigational research agent (rare exceptions allowing for the continuation of study product administration may be granted with the unanimous consent of the HVTN 104 PSRT).

- Clinically significant condition (ie, a condition that affects the immune system or for which continued study product administration and/or blood draws may pose additional risk), including but not limited to the following:
  - Pregnancy (regardless of outcome);
  - Any grade 4 local or systemic reactogenicity symptom, lab abnormality, or AE that is subsequently considered to be related to study product administration;
  - Any grade 3 lab abnormality or other clinical AE (exception: fever or vomiting and subjective local and systemic symptoms) that is subsequently considered to be related to study product administration; or
  - Clinically significant type 1 hypersensitivity reaction associated with study product administration. Consultation with the HVTN 104 PSRT is required prior to subsequent study product administration following any type 1 hypersensitivity reaction associated with study product administrations; or

- Investigator determination in consultation with HVTN 104 Protocol Team leadership (eg, for repeated nonadherence to study staff instructions).

Such participants should be counseled on the importance of continuing with the study and strongly encouraged to participate in follow-up visits and protocol-related procedures per the protocol for the remainder of the trial, unless medically contraindicated.

FOR REVIEW ONLY

In addition, study product administration will be stopped for participants diagnosed with HIV infection. HIV-infected participants will not continue in the trial after completing a 28 day safety follow-up period to the last prior study product administration, but will be monitored through other HVTN protocols (see Sections 7.3.4).

7.3.4 Participant termination from the study

Under certain circumstances, an individual participant may be terminated from participation in this study. Specific events that will result in early termination include:

- Participant refuses further participation,
- Participant relocates and remote follow-up or transfer to another HVTN CRS is not possible,
- HVTN CRS determines that the participant is lost to follow-up,
- Participant becomes HIV infected, or
- Investigator decides, in consultation with Protocol Team leadership, to terminate participation (eg, if participant exhibits inappropriate behavior toward clinic staff).
- Any condition where termination from the study is required by applicable regulations.

FOR REVIEW ONLY

8 Study product preparation and administration

CRS pharmacists should consult the Pharmacy Guidelines and Instructions for DAIDS Clinical Trials Networks for standard pharmacy operations. The protocol schema is shown in Table 3-1. See the IB for further information about study products.

8.1 Study product regimen

The schedule of infusion is shown in Section 3 and additional information is given below.

Group 1 (OPEN-LABEL)

Treatment 1 (T1): VRC-HIVMAB060-00-AB 40 mg/kg to be administered IV in 100 mL of Sodium Chloride for Injection USP, 0.9% at Month 0.

THEN

VRC-HIVMAB060-00-AB 20 mg/kg to be administered IV in 100 mL of Sodium Chloride for Injection USP, 0.9% at Months 1, 2, 3, 4, and 5.

Group 2 (OPEN-LABEL)

Treatment 2 (T2): VRC-HIVMAB060-00-AB 40 mg/kg to be administered IV in 100 mL of Sodium Chloride for Injection USP, 0.9% at Months 0, 2, and 4.

Group 3 (DOUBLE-BLIND / PLACEBO CONTROLLED)

Treatment 3 (T3): VRC-HIVMAB060-00-AB 40 mg/kg to be administered IV in 100 mL of Sodium Chloride for Injection USP, 0.9% at Month 0

THEN

VRC-HIVMAB060-00-AB 5 mg/kg to be administered SC every 2 weeks at Months 0.5, 1, 1.5, 2, 2.5, 3, 3.5, 4, 4.5, 5, and 5.5.

Placebo 3 (P3): IV Placebo for VRC01 (Sodium Chloride for Injection USP, 0.9%) to be administered IV in 100 mL of Sodium Chloride for Injection USP, 0.9% at Month 0

THEN

SC Placebo for VRC01 (VRC-PLAMAB068-00-AB) to be administered SC (subcutaneously) every 2 weeks at Months 0.5, 1, 1.5, 2, 2.5, 3, 3.5, 4, 4.5, 5, and 5.5.

Group 4 (OPEN-LABEL)

Treatment 4 (T4): VRC-HIVMAB060-00-AB 10 mg/kg to be administered IV in 100 mL of Sodium Chloride for Injection USP, 0.9% at Months 0, 2, and 4.

Group 5 (OPEN-LABEL)

FOR REVIEW ONLY

Treatment 5 (T5): VRC-HIVMAB060-00-AB 30 mg/kg to be administered IV in 100 mL of Sodium Chloride for Injection USP, 0.9% at Months 0, 2, and 4.

8.2 Study product formulation

VRC-HIVMAB060-00-AB [VRC01, Labeled as VRC01 HIV MAb Drug Product VRC-HIVMAB060-00-AB]

VRC01 will be provided in a 3 mL clear glass vial containing 2.25 mL (± 0.1 mL) of a sterile clear, colorless to yellow isotonic solution with no visible particles. Each mL contains 100 mg (± 10 mg) of VRC-HIVMAB060-00-AB in formulation buffer. The formulation buffer is composed of 25 mM sodium citrate, 50 mM sodium chloride, and 150 mM L-arginine hydrochloride at pH 5.8. Vials are intended for single use only and do NOT contain a preservative.

VRC01 is a highly concentrated protein solution and may develop white-to-translucent particles after thawing. These particles have been observed in approximately 1-3% of the vials and generally disappear over a few hours at room temperature. This particle formation has no effect on product quality. If these particles are observed after the minimum of one hour thaw time as described in Section 8.3, the vials should be held at room temperature for another 30 to 60 minutes to enhance the rate of particle dissolution. Product, free of particles, stored at room temperature, or 2-8° C, should be administered within 12 hours.

NOTE: All calculations should be based on a concentration of VRC-HIVMAB060-00-AB of 100 mg/mL and a volume of 2 mL (equal to 200 mg) can be withdrawn from a vial.

The product label designates the long-term storage temperature as -35°C to -15°C. However, the Investigator’s Brochure states that “Clinical site storage in a qualified, continuously monitored, temperature-controlled freezer with a temperature range of -45°C to -10°C is acceptable”. The study products are described in further detail in the Investigator’s Brochure (IB).

SC Placebo for VRC01 [Labeled *as* Placebo for VRC01HIV MAb VRC-PLAMAB068-00-AB]

SC Placebo for VRC01 (VRC-PLAMAB068-00-AB) will be provided in a 3 mL clear glass vial containing 2.25 mL (±0.1 mL) of a sterile clear, colorless solution with no visible particles. The sterile, buffered aqueous solution is composed of 25 mM Sodium Citrate, 50 mM Sodium Chloride, 150 mM L-Arginine Hydrochloride, 10% Dextran 40 (w/w), and 0.005% Polysorbate 80 (w/w). Vials are intended for single use only and do NOT contain a preservative.

The product label designates the long-term storage temperature as -35°C to -15°C. However, the Investigator’s Brochure states that “Clinical site storage in a qualified, continuously monitored, temperature-controlled freezer with a temperature range of -45°C to -10°C is acceptable”. The study products are described in further detail in the Investigator’s Brochure (IB).

IV Placebo for VRC01 [Labeled *as* Sodium Chloride for Injection USP, 0.9%]

FOR REVIEW ONLY

Sodium Chloride for Injection USP, 0.9% will be used as the IV Placebo for VRC01. It must be stored as directed by the manufacturer.

8.3 Preparation of study products

A new prescription MUST be sent to the pharmacy after the participant is weighed on the day of each visit during which study product will be administered. The prescription MUST contain the subject's weight and dose level prior to being sent to the pharmacy (this may NOT be communicated verbally). If this information is NOT on the prescription, the prescription will be returned to the clinic from the pharmacy to be completed appropriately prior to the pharmacist beginning preparation of study product.

8.3.1 VRC-HIVMAB060-00-AB (40mg/kg IV) - OPEN LABEL ONLY (Group 1 Day 0 only and Group 2 at Months 0, 2 and 4)

To prepare an IV infusion, the pharmacist will verify the dose [total milligrams needed (*40 mg x participant's weight in kg*)] and thaw the minimum number of vials needed to obtain the full dose. The pharmacist will determine the number of vials by dividing the total milligrams needed by 200. The result will be rounded UP to the next whole number. Note: It is expected that each vial may be used for about 2 mL withdrawal volume (200 mg VRC01).

Each vial must be thawed for a minimum of 1 hour at room temperature after removal from the freezer. After the 1 hour minimum, the pharmacist should gently swirl the vials before inspecting for particles. DO NOT SHAKE VIAL. Once swirled, the pharmacist, using aseptic technique, will add the calculated total milligrams needed to a 100 mL bag of normal saline (Sodium Chloride for Injection USP, 0.9%). Up to 50 mL of VRC01 may be added to a 100 mL bag of normal saline.

The pharmacist must note on the IV bag label the dose calculated (in mg) for the participant (eg, 2800 mg for a 70kg individual) as well as the final total volume of fluid in the IV bag (eg, 131 mL for the 70 kg individual). The IV bag should be labeled with a 12-hour expiration date and time from the time the vial is removed from the freezer. The IV bag should be stored at room temperature or refrigerated (2° - 8° C) until administration.

Note: The Sodium Chloride for Injection bags referred to as "100 mL bags" in the IV administration instructions will typically have 103 mL volume before any VRC01 is added and this is acceptable in the context of the instructions.

Any empty vials, unused portion of entered vials, or unused IV solution which contains study product should be discarded in a biohazard containment bag and incinerated or autoclaved in accordance with institutional or pharmacy policy.

8.3.2 VRC-HIVMAB060-00-AB (20mg/kg IV) - OPEN LABEL ONLY (Group 1 Months 1 through 5)

To prepare an IV infusion, the pharmacist will verify the dose [total milligrams needed *(20 mg x participant's weight in kg)*] and thaw the minimum number of vials needed to obtain the full dose. The pharmacist will determine the number of vials by dividing the total milligrams needed by 200. The result will be rounded UP to the next whole number.

FOR REVIEW ONLY

Note: It is expected that each vial may be used for about 2 mL withdrawal volume (200 mg VRC01).

Each vial must be thawed for a minimum of 1 hour at room temperature after removal from the freezer. After the 1 hour minimum, the pharmacist should gently swirl the vials before inspecting for particles. DO NOT SHAKE VIAL. Once swirled, the pharmacist, using aseptic technique will add the calculated total milligrams needed to a 100 mL bag of Sodium Chloride for Injection USP, 0.9%. Up to 50 mL of VRC01 may be added to a 100 mL bag of normal saline.

The pharmacist must note on the IV bag label the dose calculated (in mg) for the participant (e.g. 1400 mg for a 70 kg individual) as well as the final total volume of fluid in the IV bag (e.g. 117 mL for the 70 kg individual). The IV bag should be labeled with a 12-hour expiration date and time from the time the vial is removed from the freezer . The IV bag should be stored at room temperature or refrigerated (2° - 8° C) until administration.

Note: The Sodium Chloride for Injection bags referred to as “100 mL bags” in the IV administration instructions will typically have 103 mL volume before any VRC01 is added and this is acceptable in the context of the instructions.

Any empty vials, unused portion of entered vials, or unused IV solution which contains study product should be discarded in a biohazard containment bag and incinerated or autoclaved in accordance with institutional or pharmacy policy.

8.3.3 VRC-HIVMAB060-00-AB (40mg/kg IV) - DOUBLE BLIND (Group 3 T3)

To prepare an IV infusion, the pharmacist will verify the total dose [milligrams needed (*40 mg x participant’s weight in kg*)] and thaw the minimum number of vials needed to obtain the full dose. The pharmacist will determine the number of vials by dividing the total milligrams needed by 200. The result will be rounded UP to the next whole number. Note: It is expected that each vial may be used for about 2 mL withdrawal volume (200 mg VRC01).

Each vial must be thawed for a minimum of 1 hour at room temperature after removal from the freezer. After the 1 hour minimum, the pharmacist should gently swirl the vials before inspecting for particles. After thawing, the vials should be gently swirled for 30 seconds to avoid foaming. DO NOT SHAKE VIAL. Once swirled, the pharmacist, using aseptic technique will add the calculated total milligrams needed to a 100 mL bag of normal saline (Sodium Chloride for Injection USP, 0.9%). Up to 50 mL of VRC01 may be added to a 100 mL bag of normal saline.

The pharmacist must note on the IV bag label the volume of study product added to the bag as well as the final total volume of fluid in the IV bag (eg, 28 mL VRC01 or Placebo (IV) and Total Volume of 131 mL for the 70kg individual). Label the IV bag with a 12-hour expiration date and time from the time the vial is removed from the freezer . The IV bag should be stored at room temperature or refrigerated (2° - 8° C) until administration.

Note: The Sodium Chloride for Injection bags referred to as “100 mL bags” in the IV administration instructions will typically have 103 mL volume before any VRC01 is added and this is acceptable in the context of the instructions.

FOR REVIEW ONLY

Any empty vials, unused portion of entered vials, or unused IV solution which contains study product should be discarded in a biohazard containment bag and incinerated or autoclaved in accordance with institutional or pharmacy policy.

8.3.4 IV Placebo for VRC01 - DOUBLE BLIND (Group 3 C3)

To prepare an IV infusion, the pharmacist will calculate the volume of Sodium Chloride for Injection USP, 0.9% needed by multiplying the participant's weight in kg by 0.4 mL. The pharmacist, using aseptic technique will add the calculated volume of Sodium Chloride for Injection USP, 0.9% needed to a 100 mL bag of normal saline (Sodium Chloride for Injection USP, 0.9%). Up to 50 mL of Sodium Chloride for Injection USP, 0.9% may be added to a 100 mL bag of normal saline.

The pharmacist must note on the IV bag label the volume of study product added to the bag as well as the final total volume of fluid in the IV bag (eg, 28 mL VRC01 or Placebo (IV) and Total Volume of 131 mL for the 70kg individual). The IV bag should be labeled with a 12-hour expiration date and time. The IV bag should be stored at room temperature or refrigerated (2° - 8° C) until administration.

Note: The Sodium Chloride for Injection bags referred to as "100 mL bags" in the IV administration instructions will typically have 103 mL volume before any Sodium Chloride for Injection USP, 0.9% is added and this is acceptable in the context of the instructions.

Any unused IV solution which contains study product should be discarded in a biohazard containment bag and incinerated or autoclaved in accordance with institutional or pharmacy policy.

8.3.5 VRC-HIVMAB060-00-AB (5 mg/kg SC) DOUBLE BLIND (Group 3 T3)

To prepare a SC administration, the pharmacist will verify the total dose [milligrams needed *(5 mg x participant's weight in kg)*] and thaw the minimum number of vials needed to obtain the full dose. The pharmacist will determine the number of vials by dividing the total milligrams needed by 200. The result will be rounded UP to the next whole number. Note: It is expected that each vial may be used for about 2 mL withdrawal volume (200 mg VRC01).

Each vial must be thawed for a minimum of 1 hour at room temperature after removal from the freezer. After the 1 hour minimum, the pharmacist should gently swirl the vials before inspecting for particles. DO NOT SHAKE VIAL. Once swirled, the pharmacist, using aseptic technique will draw the calculated total milliliters needed into sterile syringe. If more than one syringe is needed, then the total dose should be divided as equally as possible between 2-4 syringes. The pharmacist will apply an overlay to each syringe.

The pharmacist must note on the label the final total volume of fluid in each syringe. The syringe(s) should be labeled with a 12-hour expiration date and time from the time the vial is removed from the freezer . The syringe should be stored at room temperature or refrigerated (2° - 8° C) until administration.

FOR REVIEW ONLY

Any empty vials, unused portion of entered vials, or expired prefilled syringes which contain study product should be discarded in a biohazard containment bag and incinerated or autoclaved in accordance with institutional or pharmacy policy.

8.3.6 SC Placebo for VRC01 (VRC-PLAMAB068-00-AB) DOUBLE BLIND (Group 3 C3)

To prepare a SC administration, the pharmacist will calculate the total volume needed *(0.05 mL x participant's weight in kg)* and thaw the minimum number of vials needed to obtain the full dose. Note: It is expected that each vial may be used for about 2 mL withdrawal volume.

Each vial must be thawed for a minimum of 1 hour at room temperature after removal from the freezer. After the 1 hour minimum, the pharmacist should gently swirl the vials before inspecting for particles. DO NOT SHAKE VIAL. Once swirled, the pharmacist, using aseptic technique will draw the calculated total milliliters needed into a sterile syringe. If more than one syringe is needed, then the total dose should be divided as equally as possible between 2-4 syringes. The pharmacist will apply an overlay to each syringe.

The pharmacist must note on the label the final total volume of fluid in each syringe. The syringe should be labeled with a 12-hour expiration date and time from the time the vial is removed from the freezer . The syringe should be stored at room temperature or refrigerated (2° - 8° C) until administration.

Any empty vials, unused portion of entered vials, or expired prefilled syringes which contain study product should be discarded in a biohazard containment bag in accordance with institutional or pharmacy policy.

8.3.7 VRC-HIVMAB060-00-AB (10mg/kg IV) - OPEN LABEL ONLY (Group 4 Months 0, 2, and 4)

To prepare an IV infusion, the pharmacist will verify the dose [total milligrams needed *(10 mg x participant's weight in kg)*] and thaw the minimum number of vials needed to obtain the full dose. The pharmacist will determine the number of vials by dividing the total milligrams needed by 200. The result will be rounded UP to the next whole number. Note: It is expected that each vial may be used for about 2 mL withdrawal volume (200 mg VRC01).

Each vial must be thawed for a minimum of 1 hour at room temperature after removal from the freezer. After the 1 hour minimum, the pharmacist should gently swirl the vials before inspecting for particles. DO NOT SHAKE VIAL. Once swirled, the pharmacist, using aseptic technique will add the calculated total milligrams needed to a 100 mL bag of Sodium Chloride for Injection USP, 0.9%. Up to 50 mL of VRC01 may be added to a 100 mL bag of normal saline.

The pharmacist must note on the IV bag label the dose calculated (in mg) for the participant (e.g. 700 mg for a 70 kg individual) as well as the final total volume of fluid in the IV bag (e.g. 110 mL for the 70 kg individual). The IV bag should be labeled with a 12-hour expiration date and time from the time the vial is removed from the freezer . The IV bag should be stored at room temperature or refrigerated (2° - 8° C) until administration.

FOR REVIEW ONLY

Note: The Sodium Chloride for Injection bags referred to as “100 mL bags” in the IV administration instructions will typically have 103 mL volume before any VRC01 is added and this is acceptable in the context of the instructions.

Any empty vials, unused portion of entered vials, or unused IV solution which contains study product should be discarded in a biohazard containment bag and incinerated or autoclaved in accordance with institutional or pharmacy policy.

8.3.8 VRC-HIVMAB060-00-AB (30mg/kg IV) - OPEN LABEL ONLY (Group 5 Months 0, 2, and 4)

To prepare an IV infusion, the pharmacist will verify the dose [total milligrams needed *(30 mg x participant’s weight in kg)*] and thaw the minimum number of vials needed to obtain the full dose. The pharmacist will determine the number of vials by dividing the total milligrams needed by 200. The result will be rounded UP to the next whole number. Note: It is expected that each vial may be used for about 2 mL withdrawal volume (200 mg VRC01).

Each vial must be thawed for a minimum of 1 hour at room temperature after removal from the freezer. After the 1 hour minimum, the pharmacist should gently swirl the vials before inspecting for particles. DO NOT SHAKE VIAL. Once swirled, the pharmacist, using aseptic technique will add the calculated total milligrams needed to a 100 mL bag of Sodium Chloride for Injection USP, 0.9%. Up to 50 mL of VRC01 may be added to a 100 mL bag of normal saline.

The pharmacist must note on the IV bag label the dose calculated (in mg) for the participant (e.g. 2100 mg for a 70 kg individual) as well as the final total volume of fluid in the IV bag (e.g. 124 mL for the 70 kg individual). The IV bag should be labeled with a 12-hour expiration date and time from the time the vial is removed from the freezer . The IV bag should be stored at room temperature or refrigerated (2° - 8° C) until administration.

Note: The Sodium Chloride for Injection bags referred to as “100 mL bags” in the IV administration instructions will typically have 103 mL volume before any VRC01 is added and this is acceptable in the context of the instructions.

Any empty vials, unused portion of entered vials, or unused IV solution which contains study product should be discarded in a biohazard containment bag and incinerated or autoclaved in accordance with institutional or pharmacy policy.

8.4 Administration

For additional information regarding study product administration refer to the *HVTN 104 Study Specific Procedures*.

<u>VRC01 (Intravenously)</u>

For Groups 1, 2, 4, and 5 (open label): The IV bag prepared by the pharmacy will include the total amount (mg) of VRC01 added to the 100 mL normal saline bag and the final volume of the bag. The clinician responsible for administration and another clinician will each check the bag label and confirm that the identifier is correct and that the correct

FOR REVIEW ONLY

total milligrams to be administered is shown based on subject weight and dosage level before beginning the IV administration. The investigational study product solution will typically be administered IV over 60 minutes using a volumetric pump. The rate of infusion (mL/hr) will vary based on the total volume needed to administer the full dose. The total time needed to administer the dose may be longer based on factors such as subject tolerance.

VRC01 or IV Placebo (Intravenously)

For Group 3 Month 0 (blinded): The IV bag prepared by the pharmacy will include the total amount (mL) of VRC01 or IV Placebo for VRC01 (Sodium Chloride for Injection 0.9%, USP) added to the 100 mL normal saline bag and the final volume of the bag. The clinician responsible for administration and another clinician will each check the bag label and confirm that the identifier is correct and that the correct total milliliters to be administered is shown based on subject weight and dosage level before beginning the IV administration.

The investigational study product solution will typically be administered IV over 60 minutes using a volumetric pump. The rate of infusion (mL/hr) will vary based on the total volume needed to administer the full dose. The total time needed to administer the dose may be longer based on factors such as subject tolerance.

VRC01 or SC Placebo (Subcutaneously)

The SC syringe(s) prepared by the pharmacy will include the total amount (mL) of VRC01 or SC Placebo (VRC-PLAMAB068-00-AB). At the time of administration, the clinician and participant will again discuss the administration site(s) (abdomen, upper arm, or thigh) and deem the one selected to be acceptable.

If more than one syringe is needed, the ziplock bag containing the syringes will show the total volume to be administered. The clinician responsible for administration and another clinician will each check the syringe/ziplock bag label and confirm that the identifier is correct and that the correct total milliliters to be administered is shown based on the participant's weight and dosage level before administering the SC injection(s).

All SC injections must be at least 2 inches apart.

When preparing a dose in a syringe and administering the dose, consideration should be given to the volume of solution in the needle before and after the dose is administered. Particularly if the needle used to withdraw the product is replaced prior to product administration, consideration should be given to conserving the full dose of product. The pharmacy and clinic staff members are encouraged to work together to administer the dose specified in the protocol.

8.5 Acquisition of study products

VRC-HIVMAB060-00-AB and VRC-PLAMAB068-00-AB are provided by the VRC/DAIDS/NIAID.

IV Placebo for VRC01 (Sodium Chloride for Injection USP, 0.9%) will not be provided through the protocol and must be obtained by the site.

FOR REVIEW ONLY

Once an HVTN CRS is protocol registered, the pharmacist can obtain study products from the NIAID Clinical Research Products Management Center (CRPMC) by following the ordering procedures given in Pharmacy Guidelines and Instructions for DAIDS Clinical Trials Networks.

8.6 Pharmacy records

The HVTN CRS pharmacist is required to maintain complete records of all study products. The pharmacist of record is responsible for maintaining randomization codes and randomization confirmation notices for each participant in a secure manner.

8.7 Final disposition of study products

All unused study products must be returned to the CRPMC after the study is completed or terminated unless otherwise instructed by the CRPMC. The procedures and relevant form are included in the Pharmacy Guidelines and Instructions for DAIDS Clinical Trials Networks.

FOR REVIEW ONLY

9 Clinical procedures

The schedules of clinical procedures are shown in Appendix H and Appendix I and Appendix J.

9.1 Informed consent

Informed consent is the process of ensuring that participants fully understand what will and may happen to them while participating in a research study. The HVTN informed consent form documents that a participant (1) has been informed about the potential risks, benefits, and alternatives to participation, and (2) is willing to participate in an HVTN study. Informed consent encompasses all written or verbal study information HVTN CRS staff provide to the participant, before and during the trial. HVTN CRS staff will obtain informed consent of participants according to HVTN policies and procedures.

The informed consent process continues throughout the study. Key study concepts should be reviewed periodically with the participant and the review should be documented. At each study visit, HVTN CRS staff should consider reviewing the procedures and requirements for that visit and for the remaining visits. Additionally, if any new information is learned that might affect the participants' decisions to stay in the trial, this information will be shared with trial participants. If necessary, participants will be asked to sign revised informed consent forms.

An HVTN CRS may employ recruitment efforts prior to the participant consenting. For example, some HVTN CRSs use a telephone script to prescreen people before they come to the clinic for a full screening visit. Participants must sign a screening or protocol-specific consent before any procedures are performed to determine eligibility. HVTN CRSs must submit recruitment and prescreening materials to IRB/EC and any applicable Regulatory Entity (RE) for human subjects protection review and approval.

Note: As defined in the DAIDS Protocol Registration Manual, an RE is "Any group other than the local IRB/EC responsible for reviewing and/or approving a clinical research protocol and site-specific ICFs prior to implementation at a site." CRSs are responsible for knowing the requirements of their applicable REs.

9.1.1 Screening consent form

Without a general screening consent, screening for a specific study cannot take place until the site receives protocol registration from the DAIDS Protocol Registration Office's Regulatory Support Center (RSC).

Some HVTN CRSs have approval from their IRB/EC and any applicable RE to use a general screening consent form that allows screening for an unspecified HIV prevention trial. In this way, HVTN CRS staff can continually screen potential participants and, when needed, proceed quickly to obtain protocol-specific enrollment consent. Sites conducting general screening or prescreening approved by their IRB/EC and any applicable RE may use the results from this screening to determine eligibility for this protocol, provided the tests are conducted within the time periods specified in the eligibility criteria.

FOR REVIEW ONLY

9.1.2 Protocol-specific consent forms

The protocol-specific consent forms describe the study products to be used and all aspects of protocol participation, including screening and enrollment procedures. A sample protocol-specific consent form for the main study is located in Appendix A. A separate sample consent form for other uses of specimens is located in Appendix C.

Each HVTN CRS is responsible for developing a protocol-specific consent form(s) for local use, based on the sample protocol-specific consent forms in Appendix A and Appendix C. The consent form(s) must be developed in accordance with requirements of the following:

- CRS's IRB/EC,
- CRS's institution and any applicable REs, and
- Elements of informed consent as described in Title 45, Code of Federal Regulations (CFR) Part 46 and Title 21 CFR, Part 50, and in the International Conference on Harmonisation (ICH) E6, Good Clinical Practice: Consolidated Guidance 4.8.

Study sites are strongly encouraged to have their local CABs review their sites-specific consent forms. This review should include, but should not be limited to, issues of cultural competence, local language considerations, and the level of understandability.

The sample informed consent form includes instructions throughout the document for developing specific content.

Sites should follow the instructions in the Protocol-specific Official Memo distributed along with this protocol regarding when they may begin using their site-specific protocol consent forms.

Regarding protocol registration, sites should follow procedures outlined in the current version of the DAIDS Protocol Registration Manual.

9.1.3 Assessment of Understanding

Study staff are responsible for ensuring that participants fully understand the study before enrolling them. This process involves reviewing the informed consent form with the participant, allowing time for the participant to reflect on the procedures and issues presented, and answering all questions completely.

An Assessment of Understanding is used to document the participant's understanding of key concepts in this clinical trial. The participant must complete the Assessment of Understanding before enrollment. Staff may provide assistance in reading and understanding the questions and responses, if necessary. Participants must verbalize understanding of all questions answered incorrectly. This process and the participant's understanding of the key concepts should be recorded in source documentation at the site.

IRB/EC and any applicable RE may require that a participant has signed either a screening or protocol-specific consent document prior to administering the Assessment of Understanding. The consent process (including the use of the Assessment of

FOR REVIEW ONLY

Understanding) should be explained thoroughly to the IRB/EC and any applicable RE, whose recommendations should be followed.

9.2 Pre-enrollment procedures

Screening may occur over the course of several contacts/visits, up to and including before infusion on day 0. All inclusion and exclusion criteria must be assessed within 56 days before enrollment, unless otherwise specified in the eligibility criteria (or below in this section).

After the appropriate informed consent has been obtained and before enrollment, the following procedures are performed:

- Medical history, documented in the case history record;
- Assessment of whether the volunteer is at low risk for HIV infection;
- Complete physical examination, including height, weight, vital signs, and clinical assessments of head, ears, eyes, nose, and throat; neck; lymph nodes; heart; chest; abdomen; extremities; neurological function; and skin;
- Assessment of concomitant medications the volunteer is taking, including prescription and nonprescription drugs, vitamins, topical products, alternative/complementary medicines (eg, herbal and health food supplements), recreational drugs, vaccinations, and allergy shots (record the complete generic name for all medications);
- Laboratory tests as defined in the inclusion and exclusion criteria, including:
  - Screening HIV test,
  - HBsAg,
  - Anti-HCV Abs,
  - Syphilis test,
  - Complete blood count (CBC) with differential and platelets,
  - Chemistry panel (ALT, AST, alkaline phosphatase, creatinine),
  - Urine dipstick (urinalysis if indicated; see Section 9.8),
  - Urine or serum pregnancy test (volunteers who were born female); Persons who are not of reproductive potential due to having undergone total hysterectomy with bilateral oophorectomy (verified by medical records), are not required to undergo pregnancy testing;
- Administration of behavioral risk assessment questionnaire;
- Obtaining of volunteer demographics in compliance with the NIH Policy on Reporting Race and Ethnicity Data: Subjects in Clinical Research, Aug. 8, 2001 (available at http://grants.nih.gov/grants/guide/notice-files/NOT-OD-01-053.html);

FOR REVIEW ONLY

- Counseling on HIV testing and risk reduction, performed in compliance with the US Centers for Disease Control and Prevention (CDC)'s current guidelines or other local guidelines for HIV counseling, testing, and referral as described in Section 9.5; and

- Discussion of pregnancy prevention. A pregnant or breastfeeding person may not be enrolled in this trial. Specific criteria and assessment of contraception and pregnancy status are described in study inclusion criteria. Discussion of pregnancy prevention includes advising a participant who was born female and who reports no current sexual activity that could lead to that participant becoming pregnant to have a plan to begin adequate birth control. This plan would be put to use if, during the study, the participant becomes sexually active in a way that could lead to that participant becoming pregnant.

9.2.1 Use of screening results from another HVTN study

If a participant screens for an HVTN study at the same HVTN CRS but then does not join that study, screening results from that effort may be applied to the screening for this protocol, as long as the screening was done under participant consent, the participant has signed a consent form to begin screening for this study, and the tests were conducted within the time periods specified in the eligibility criteria (see Sections 7.1 and 7.2).

9.3 Enrollment and study product administration visits

Enrollment is simultaneous with first infusion. The time interval between randomization and enrollment should not exceed 4 working days. The HVTN CRS registers the participant by scheduling the day 0 visit (enrollment) via the Web-based randomization system, and requests the randomization assignment. Circumstances may require a participant's enrollment visit to be changed. This may exceed the 4-day randomization time limit.

At all study product administration visits, the following procedures are performed before study product administration:

- Abbreviated physical examination, including weight, vital signs, and a symptom-directed evaluation by history and/or appropriate physical exam based on participant self-reported symptoms or complaints;

- Assessment of baseline reactogenicity parameters;

- Assessment of concomitant medications (as described in Section 9.2);

- Assessment of any new or unresolved AEs/intercurrent illnesses; and

- Urine or serum pregnancy test (for participants who were born female). Persons who are not of reproductive potential due to having undergone total hysterectomy with bilateral oophorectomy (verified by medical records), are not required to undergo pregnancy testing.

FOR REVIEW ONLY

Following completion of all procedures in the preceding list and results indicate that study product administration may proceed, the study products are prepared and administered (see Sections 8.3 and 8.4).

Immediately following study product administration, the participant remains in the clinic for observation. An initial reactogenicity assessment is made at a target of 60 minutes after all IV infusions and the first SC injection (visit 3) in Group 3, with an acceptable range of 60-120 minutes. For subsequent SC injections in Group 3, an initial reactogenicity assessment is made at a target of 30 minutes, with an acceptable range of 25-60 minutes. Before leaving the clinic, the participant is given the postproduct symptom log and is instructed on how to complete it. The site will make arrangements to obtain daily reports of reactogenicity events from the participant during the reactogenicity period (as described in Section 9.9).

The following procedures will be performed at all study product administration visits. These procedures may be performed prior to or following study product administration:

- Risk reduction counseling (as described in Section 9.6);
- Pregnancy prevention assessment (as described in Section 9.2 and 9.7); and
- Assessment of new or unresolved social impacts (site staff will ask participant about the status of any unresolved social impacts and if s/he has experienced any new social impacts as a result of the trial participation).

The following procedure will be performed at all study product administration visits. This procedure must be performed following study product administration:

- Administration of the acceptability questionnaire (level of discomfort with the procedures, willingness to undergo procedures in a "real world" setting; level of pain, and level of anxiety).

Additional procedures will be performed at scheduled visits as specified in Appendix H and Appendix I and Appendix J:

- Confirm that participants received HIV test results from previous visit. If not, provide test results and post-test counseling as appropriate; and
- Specimen collection (to be completed prior to study product administration).

FOR REVIEW ONLY

9.4 Follow-up visits

The following procedures are performed at all scheduled follow-up visits:

- Risk reduction counseling (as described in Section 9.6);
- Pregnancy prevention assessment (as described in Section 9.2 and 9.7); and

- Assessment of new or unresolved social impacts (site staff will ask participant about the status of any unresolved social impacts and if s/he has experienced any new social impacts as a result of the trial participation);

- Administration of the acceptability questionnaire (level of discomfort with the procedures, willingness to undergo procedures in a "real world" setting; level of pain, and level of anxiety);

- Assessment of new or continuing concomitant medications (as described in Section 9.2); and

- Assessment of new or unresolved AEs/intercurrent illnesses.

Additional procedures will be performed at scheduled follow-up visits as specified in Appendix H and Appendix I and Appendix J:

- Administration of the social impact assessment questionnaire (types of impacts assessed involve personal relationships, health insurance, life insurance, educational or employment opportunities, housing, immigration, or travel);

- Participants in Group 3 will also be asked whether they believe they received the active study product or the control;

- HIV infection assessment including pretest counseling. A subsequent follow-up contact is conducted to provide posttest counseling and to report results to participant;

- Confirm that participants received HIV test results from previous visit. If not, provide test results and post-test counseling as appropriate;

- Abbreviated physical examination including weight, vital signs, and a symptom-directed evaluation by history and/or appropriate physical exam based on participant self-reported symptoms or complaints;

- Complete physical examination, including weight, vital signs, and clinical assessments of head, ears, eyes, nose, and throat; neck; lymph nodes; heart; chest; abdomen; extremities; neurological function; and skin;

- Specimen collection;

- Clinical laboratory tests including:
  - CBC with differential and platelet count,
  - Chemistry panel (see Section 9.2), and
  - Urine dipstick (urinalysis if appropriate; see Section 9.8); and

- Urine or serum pregnancy test (for participants who were born female); Persons who are not of reproductive potential due to having undergone total hysterectomy with bilateral oophorectomy (verified by medical records), are not required to undergo pregnancy testing.

FOR REVIEW ONLY

9.5 Mucosal secretion sampling

Mucosal secretion samples will be collected from all study participants who agree to these procedures at timepoints indicated Appendix H and Appendix I and Appendix J (see also HVTN 104 *Study Specific Procedures*). These samples include saliva, rectal secretions, semen (only for people born male), or cervical secretions (only for people born female).

Mucosal sampling must be performed prior to study product administration.

For participants born female providing cervical and/or rectal samples, a pregnancy test must be performed and must be negative prior to any mucosal sampling. Persons who are not of reproductive potential due to having undergone total hysterectomy with bilateral oophorectomy (verified by medical records), are not required to undergo pregnancy testing.

The following conditions apply to mucosal sampling. Study participants are advised of these in the study informed consent form (see Appendix A):

Salivary fluid sampling: Participants willing to provide salivary samples should abstain from smoking, eating, or drinking anything other than water, brushing their teeth, using mouthwash, chewing gum or tobacco, or engaging in intimate oral activity for one hour prior to sample collection.

Rectal fluid sampling: Participants who consent to provide rectal samples are subject to the following exclusion criteria:

- Participant is pregnant. For participants born female, a pregnancy test must be performed and be negative prior to any rectal mucosal sampling.
- Participant has engaged in receptive anal intercourse or insertion of any foreign object or substance into the anus for 48 hours prior to sample collection. Foreign objects include, but are not limited to cleaning products (creams, gels, lotions, pads, etc.), lubricant, enemas, and douching, even with water.
- Participant has used any perianal or intra-anal steroid or other anti-inflammatory creams for 48 hours prior to sample collection.
- Participant has any active infection or inflammation of the colorectal area (such as an HSV-2 outbreak or inflamed hemorrhoids or colitis/diarrhea).
- Participant has any active Genital Tract Infections (GTI), including untreated syphilis. If participant has received treatment for syphilis, documentation of resolution must be obtained prior to sampling.

Cervicovaginal secretion sampling: Participants who consent to provide cervicovaginal samples are subject to the following exclusion criteria:

- Participant is pregnant.
- Participant is menstruating.

FOR REVIEW ONLY

- Participant has engaged in vaginal intercourse or insertion of any foreign object or substance into the vagina for 48 hours prior to sample collection.

- Participant has used any spermicide, lubricants or topical/intravaginal medications (e.g. topical yeast infection treatments), including douching, within 48 hours prior to sample collection.

- Presence of an active ulcerative genital lesion.

- Participant has abnormal Pap smear - participants 21 years of age or older must report having had a Pap smear within the 3 years prior to enrollment, with the latest result reported as normal or ASCUS (atypical squamous cells of undetermined significance) [see USPSTF guidelines].

Semen sampling: Participants born male who consent to provide semen samples are requested to refrain from ejaculation for at least 48 hours prior to specimen collection. In addition, participants are subject to the following exclusion criteria:

- Participant has any active Genital Tract Infections (GTI), including untreated syphilis. If participant has received treatment for syphilis, documentation of resolution must be obtained prior to sampling.

9.6 HIV counseling and testing

HIV counseling will be performed in compliance with the CDC's guidelines or other local guidelines for HIV counseling and referral. HIV testing will be performed in accordance with the current HVTN HIV testing algorithm following enrollment.

Participants will be counseled routinely during the trial on the avoidance of HIV infection and on the potential negative social impacts of the theoretical possibility of testing antibody positive due to the study product. They will also be counseled on the risks of HIV antibody testing outside of the HVTN CRSs and will be discouraged from doing so during study participation and/or during any period of study product reactive serology.

Study staff will take particular care to inform study participants of the likelihood of routine HIV testing being offered or performed outside the study CRS at emergency rooms, clinics, and medical offices. Such testing has become more likely due to the CDC's revised guidelines for HIV counseling and testing, as well as policy changes in many countries to make HIV testing more frequent and routine. CRS staff should inform participants of their right to opt out of HIV testing outside the study site. CRS staff should inform study participants if local and/or state policies and regulations permit medical providers to perform HIV testing without first informing patients. If this is the case, then CRS staff should advise study participants that they may decline testing preemptively. CRS staff should also inform participants if positive results must be reported to local public health authorities. CRS staff should also inform participants of the need to maintain study blinding by getting HIV testing only at the study CRS. CRS staff should provide participants with CRS contact information and should encourage participants to ask medical providers to contact the CRS. The CRS can verify that the participant is in an HIV vaccine clinical trial and should only be tested at the study CRS.

FOR REVIEW ONLY

Potential participants identified as being HIV infected during screening are not enrolled. All participants who become HIV infected during the study will be terminated from this study. Potential and enrolled participants identified as HIV infected will be referred for medical treatment, counseling, and management of the HIV infection. These individuals may also be referred to appropriate ongoing clinical trials or observational studies.

9.6.1 Distinguishing intercurrent HIV infection from study product reactive serology

The following procedures will be conducted in order to assess study product reactive serology:

- Participants will have physical examinations at visits specified in Appendix H and Appendix I and Appendix J. Signs or symptoms of an acute HIV infection syndrome, an intercurrent illness consistent with HIV-1 infection, or probable HIV exposure would prompt a diagnostic workup per the HVTN algorithm for Recent Exposure/Acute Infection Testing to determine HIV infection.

- HIV testing will be performed at multiple timepoints throughout the study (see Appendix E and Appendix F and Appendix G). The Laboratory Program (or approved diagnostic laboratory) will follow the HVTN HIV testing algorithm (as described in the HVTN Site Lab Reference Manual), which is able to distinguish study product reactive serology responses from actual HIV infections.

- All participants can receive HIV-1 diagnostic testing from the site following their last scheduled visit until they are told that they did not receive VRC01 or that antibody-based HIV diagnostic tests are not reactive.

- All participants who received VRC01 and who have VRC01-induced positive or indeterminate HIV-1 serology (as measured by the standard anti-HIV antibody screening tests) at or after the study is unblinded will be offered poststudy HIV-1 diagnostic testing (per the HVTN poststudy HIV-1 testing algorithm) periodically and free of charge as medically/socially indicated (approximately every 6 months).

9.7 Contraception status

Contraception status is assessed and documented at every scheduled clinic visit for a participant who was born female and who is sexually active in a way that could cause that participant to become pregnant. Prior to enrollment and throughout the study, staff will ask participants to verbally confirm their use of adequate contraceptive methods. A participant who was born female and is sexually active in a way that could cause that participant to become pregnant should be reminded at all scheduled clinic visits of the importance of using contraception and should be referred to specific counseling, information, and advice as needed. (Specific contraception requirements are listed in Section 7.1). This reminder should be documented in the participant's study record.

Self-reported infertility—including having reached menopause (no menses for 1 year) or having undergone hysterectomy, bilateral oophorectomy, or tubal ligation—must be documented in the participant's study record.

FOR REVIEW ONLY

9.8 Urinalysis

Dipstick testing may be performed in the clinic or the lab, as long as the required elements (glucose, protein, and hemoglobin) are tested. The examination is performed on urine obtained by clean catch.

If the screening dipstick is transiently abnormal due to menses or infection, document this issue in the participant's source documentation. For infection, provide appropriate treatment and/or referral. Following resolution, repeat the dipstick and, if within the eligibility limits specified in the protocol, the participant may be enrolled.

Follow-up urinalysis should be deferred if a participant is menstruating, but should be performed as soon as possible. If a follow-up dipstick is abnormal due to a participant's menstrual period, document in the comment section of the case report form (CRF) and repeat the dipstick once the participant is no longer menstruating; a micro-urinalysis is not required.

9.9 Assessments of reactogenicity

For all participants, baseline assessments are performed before and reactogenicity assessments are performed after each study product administration. All reactogenicity symptoms are followed until resolution and graded according to the Division of AIDS Table for Grading the Severity of Adult and Pediatric Adverse Events (DAIDS AE Grading Table), Version 1.0, December 2004 (Clarification August 2009).

The reactogenicity assessment period is 3 full days following each study product administration per the assessment schedule shown in Table 9-1. Participants are instructed to record symptoms using a post product symptom log and to contact the site daily during the assessment period. Clinic staff will follow new or unresolved reactogenicity symptoms present at day 3 to resolution. Participants are instructed to contact the clinic for events that arise during the period between study product administration and the next scheduled visit. In general, a participant who self-reports any post administration reaction greater than mild is seen by a clinician within 48 hours after onset, unless the reaction is improving and/or has completely resolved.

Reactogenicity events are reported using CRFs that correspond to the time of assessment in Table 9-1. Reactogenicity assessments include assessments of systemic and local symptoms, and study product-related lesions. Events not listed on a CRF, or with an onset after the reactogenicity assessment period (day of study product administration and 3 full days after), or those meeting SAE/adverse events requiring expedited reporting to DAIDS criteria, are recorded on an adverse experience log form.

FOR REVIEW ONLY

Table 9-1 Schedule of reactogenicity assessments

Day	Time	Performed by
0[a]	Baseline: before study product administration	HVTN CRS staff
	Early: 60-120 minutes after all IV infusions and first SC injection in Group 3; 25-60 minutes after subsequent SC injections in Group 3	HVTN CRS staff
	Between early assessment and 11:59pm day 0	HVTN CRS staff or participant
1	Between 12:00am and 11:59pm day 1	HVTN CRS staff or participant
2	Between 12:00am and 11:59pm day 2	HVTN CRS staff or participant
3[b]	Between 12:00am and 11:59pm day 3	HVTN CRS staff or participant

[a] Day of study product administration

[b] New or unresolved reactogenicity symptoms present on day 3 are followed until resolution

9.9.1 Assessment of systemic and local symptoms

Typical study product administration site reactions are erythema/induration/swelling/edema. The maximum horizontal and maximum vertical measurements for all study product administrationsite reactions are recorded.

Body temperature is measured by oral or infrared thermometry and reported in degrees Celsius. If temperature is measured in Fahrenheit, the conversion to Celsius should be documented in the participant’s chart note. A measurement is taken once daily during the assessment period and should be repeated if participant is feeling feverish.

9.9.2 Assessment of the study product administration site

Typical study product administration site reactions are erythema/induration/swelling/edema. The maximum horizontal and maximum vertical measurements for all study product administration site reactions are recorded.

All study product administration site reactions are monitored until resolution. Areas greater than 25 cm^2 are followed daily; otherwise, the frequency of follow-up is based on clinician judgment.

9.10 Visit windows and missed visits

Visit windows are defined in HVTN 104 Study Specific Procedures. For a visit not performed within the window period, a Missed Visit form is completed. If the missed visit is one that required safety assessments or local safety labs, HVTN CRS staff should attempt to bring the participant in for an interim visit as soon as possible.

Procedures performed at an interim visit are usually toxicity/safety assessments (including local safety labs) and HIV testing. With the exception of HIV testing, these procedures are performed only if they were required at the missed visit or if clinically indicated. HIV testing may be performed as deemed appropriate by the study staff. Blood samples for immunogenicity assays are not typically collected at interim visits.

If a missed visit required study product administration, please refer to Section 7.3.2 and Section 7.3.3 for resolution.

FOR REVIEW ONLY

9.11 Early termination visit

In the event of early participant termination, site staff should consider if the following assessments are appropriate: a final physical examination, clinical laboratory tests (including urine dipstick, CBC with differential, platelet count, and chemistry panel), pregnancy testing, social impact assessment, and HIV test.

9.12 Pregnancy

If a participant becomes pregnant during the course of the study, no more administrations of study product will be given, but remaining visits and study procedures should be completed unless medically contraindicated or applicable regulations require termination from the study. In case of required termination, enrollment in an observational study should be offered to the participant. If the participant terminates from the study prior to the pregnancy outcome, the site should make every effort to keep in touch with the participant in order to ascertain the pregnancy outcome.

FOR REVIEW ONLY

10 Laboratory

10.1 HVTN CRS laboratory procedures

The HVTN Site Lab Reference Manual provides further guidelines for operational issues concerning the clinical and processing laboratories. The manual includes guidelines for general specimen collection, special considerations for phlebotomy, specimen labeling, whole blood processing, HIV screening/diagnostic testing, and general screening and safety testing.

Tube types for blood collection are specified in Appendix E and Appendix F and Appendix G. For tests performed locally, the local lab may assign appropriate tube types.

In specific situations, the blood collection tubes may be redirected to another laboratory or may require study-specific processing techniques. In these cases, laboratory special instructions will be posted on the protocol-specific section of the HVTN website.

10.2 Total blood volume

Required blood volumes per visit are shown in Appendix E and Appendix F and Appendix G. The FHCRC laboratory will further specify the tube type and collection volumes in special instructions posted to the protocol-specific section of the HVTN website.) Not shown is any additional blood volume that would be required if a safety lab needs to be repeated, or if a serum pregnancy test needs to be performed. The additional blood volume would likely be minimal. The total blood volume drawn for each participant will not exceed 500 mL in any 56-day (8-week) period.

10.3 Primary timepoint

The primary timepoint in this study is at visit 19 (day 168) (ie, 4 weeks after the 6th study product administration) for Group 1; visit 19 (day 168) (ie, 8 weeks after the 3rd study product administration) for Groups 2, 4, and 5; and visit 19 (day 168) (2 weeks after the 12th study product administration) for Group 3. Other time points may also be examined to better understand how serum concentrations of VRC01 change. The schedules are shown in Appendix E and Appendix F and Appendix G.

10.4 Drug levels

10.4.1 VRC01 mAb levels

VRC01 levels will be measured in serum and mucosal secretions including saliva, semen, cervical secretions, and rectal secretions. An ELISA will be used to determine the concentration of the VRC01 antibody in the serum. The ELISA employs the VRC01 Fab-specific 5C9 mAb, which is an anti-idiotype antibody cloned from a single B cell that was sorted by flow cytometry using a VRC01 scFv probe. ELISA will also be used to detect the presence of VRC01 antibody in various mucosal secretions. The 4-parameter

FOR REVIEW ONLY

logistic curve regression of a standard curve of VRC01 covering the range from 0.031 to 1.0 mcg/mL is utilized in this assay to quantitate the sample concentrations based upon the average of sample dilutions within the range of the assay. This assay has been qualified but not formally validated. The functional sensitivity for the generation of the ELISA assay format, which is currently used at NVITAL, is 2 mcg/ml; and as the technology for this assay continues to develop, an updated assay may be utilized

Binding multiplex antibody assay (BAMA) may also be used to assess binding of VRC01 from serum and mucosal secretions to various Env proteins, including Consensus gp120, Consensus gp140 and CD4 binding site proteins. The lower limit of detection will be determined for the matched lot of VRC01 study product prior to assessment of VRC01 binding in HVTN 104 participants. However, historical control assays carried out under GCLP validated assay conditions indicate that the lower limit of detection for VRC01 is at least 1-10 ng/ml.

10.5 Endpoint assays: humoral

10.5.1 Anti-VRC01 antibody assay

Assessment for development of anti-VRC01 antibodies in subjects will be performed using the Forte Bio Octet BioLayer Interferometry (BLI) technology. The assay uses VRC01 immobilized to a biosensor. The biosensor is dipped into patient serum samples and antibodies against VRC01 are directly measured. The binding response is directly proportional to the anti-drug concentration as determined against a calibration curve using the 5C9 antibody.

10.5.2 Neutralizing antibody assay

Depending upon the concentrations measured in collected specimens, further evaluation of the research samples to assess for functional capacity to neutralize HIV in blood and mucosal secretions may be evaluated by an *in vitro* cell-based virus neutralization assay [50-52] using pseudotyped viruses.

One or more viruses that are among the most sensitive to VRC01 (e.g. MN.3 and MW965.26) will be assayed. The IC50 of VRC01 against both of these viruses is 0.01 – 0.03 mcg/ml and the IC90 of VRC01 against both of these viruses is 0.2 mcg/ml. The TZM-bl assay is validated for this level of sensitivity.

10.6 Genotyping

Molecular human leukocyte antigen (HLA) typing may be performed on enrolled participants using cryopreserved PBMC collected at baseline. Other participants (including control recipients) may be HLA-typed to support future studies of immunological interest at the discretion of the HVTN Laboratory Program. Other markers, such as genes associated with immune responses or HIV-1 disease progression may also be assessed.

FOR REVIEW ONLY

10.7 Exploratory studies

Samples may be used for other testing and research related to furthering the understanding of HIV immunology or vaccines. In addition, cryopreserved samples may be used to perform additional assays to support standardization and validation of existing or newly developed methods.

10.8 Other use of stored specimens

The HVTN stores specimens from all study participants indefinitely, unless a participant requests that specimens be destroyed or if required by IRB/EC, or RE.

Other use of specimens is defined as studies not described in the protocol.

This research may relate to HIV, vaccines, the immune system, and other diseases. This could include limited genetic testing and, potentially, genome-wide studies. This research is done only to the extent authorized in each study site's informed consent form, or as otherwise authorized under applicable law. Other testing on specimens will occur only after review and approval by the HVTN, the IRB/EC of the researcher requesting the specimens, and the CRS's IRBs/ECs if required.

The protocol sample informed consent form is written so that the participant either explicitly allows or does not allow their samples to be used in other research when they sign the form. Participants who initially agree to other use of their samples may rescind their approval once they enter the study; such participants will remain in this study and their samples will only be used for the studies described in this protocol. If a participant decides against allowing other research using his or her samples, or at any time rescinds prior approval for such other use, the study site investigator or designee must notify HVTN Regulatory Affairs in writing. In either case, HVTN Regulatory Affairs directs the HVTN Lab Program not to use samples from these participants for such other uses.

CRSs must notify HVTN Regulatory Affairs if institutional or local governmental requirements pose a conflict with or impose restrictions on other use of specimens.

10.9 Biohazard containment

As the transmission of HIV and other blood-borne pathogens can occur through contact with contaminated needles, blood, and blood products, appropriate precautions will be employed by all personnel in the drawing of blood and shipping and handling of all specimens for this study, as currently recommended by the CDC and the NIH or other applicable agencies.

All dangerous goods materials, including Biological Substances, Category A or Category B, must be transported according to instructions detailed in the International Air Transport Association Dangerous Goods Regulations.

FOR REVIEW ONLY

11 Safety monitoring and safety review

11.1 Safety monitoring and oversight

11.1.1 HVTN 104 PSRT

The HVTN 104 PSRT is composed of the following members:

- DAIDS medical officer representative,
- Protocol chair and cochair,
- Protocol Team leader,
- Core medical monitor, and
- Clinical safety specialist.

The clinician members of HVTN 104 PSRT are responsible for decisions related to participant safety.

The Protocol Team clinic coordinator, project manager, vaccine developer representative, clinical trial manager, and others may also be included in HVTN 104 PSRT meetings.

11.1.2 HVTN SMB

The SMB is a multidisciplinary group consisting of biostatisticians, clinicians, and experts in HIV vaccine research that, collectively, has experience in the conduct and monitoring of vaccine and drug trials. Members of the SMB are not directly affiliated with the protocols under review.

The SMB reviews safety data, unblinded as to treatment arm, approximately every 4 months. The reviews consist of evaluation of cumulative reactogenicity events, AE, laboratory safety data, and individual reports of adverse events requiring expedited reporting to DAIDS. To increase the sensitivity for detecting potential safety problems, the SMB will review safety data aggregated across multiple protocols that use the same or similar vaccine candidates. The SMB conducts additional special reviews at the request of the HVTN 104 PSRT.

Study sites will receive SMB summary minutes and are responsible for forwarding them to their IRB/EC and any applicable RE.

11.1.3 SDMC roles and responsibilities in safety monitoring

The roles and responsibilities of the SDMC in relation to safety monitoring include:

- Maintaining a central database management system for HVTN clinical data;

FOR REVIEW ONLY

- Providing reports of clinical data to appropriate groups such as the HVTN 104 PSRT and HVTN SMB (see Section 11.1.2).

11.1.4 HVTN Core roles and responsibilities in safety monitoring

The roles and responsibilities of HVTN Core in relation to safety monitoring include:

- Daily monitoring of clinical data for events that meet the safety pause and HVTN 104 PSRT AE review criteria (see Section 11.3);

- Notifying HVTN CRSs and other groups when safety pauses or planned holds are instituted and lifted (see Section 11.3);

- Querying HVTN CRSs for additional information regarding reported clinical data; and

- Providing support to the HVTN 104 PSRT.

11.2 Safety reporting

11.2.1 Submission of safety forms to SDMC

Sites must submit all safety forms (eg, reactogenicity, adverse experience, urinalysis, local lab results, and concomitant medications) before the end of the next business day after receiving the information. The forms should not be held in anticipation of additional information at a later date. If additional information is received at a later date, the forms should be updated and refaxed before the end of the next business day after receiving the new information.

11.2.2 AE reporting

An AE is any untoward medical occurrence in a clinical investigation participant administered a study product/procedure(s) and which does not necessarily have a causal relationship with this treatment. An AE can therefore be any unfavorable and unintended sign (including an abnormal laboratory finding), symptom, or disease temporally associated with the use of an investigational study product/procedure(s), whether or not related to the investigational study product/procedure(s). All AEs are graded according to the Division of AIDS Table for Grading the Severity of Adult and Pediatric Adverse Events (DAIDS AE Grading Table), Version 1.0, December 2004 (Clarification dated August 2009), available on the RSC website at http://rsc.tech-res.com/safetyandpharmacovigilance/, except that unintentional weight loss of less than 10% loss in body weight from baseline is not required to be reported as an adverse event. As shown in Appendix K, two additional parameters have been added to the DAIDS AE Grading Table for HVTN 104.

All AEs are reported to the SDMC on the appropriate CRF. Clinic staff should evaluate every AE to determine if (1) the AE meets the requirements for expedited reporting to DAIDS (Section 11.2.3) and (2) if the AE meets the criteria for a safety pause/prompt AE review (Section 11.3).

FOR REVIEW ONLY

Sites are expected to notify the CSS of any serious safety concern requiring their attention (see Table 11-1). Telephone numbers and email addresses are listed in the Key Resource Guide of the HVTN 104 Study Specific Procedures. Concerns requiring immediate HVTN Core clinical safety staff attention should be communicated by calling the clinical safety phone.

In the case of email notification, the CSS will reply during working hours (US Pacific Time) to confirm that the email has been received and reviewed. If email service is not available, the HVTN CRS should notify the CSS of the event by telephone, then submit CRFs.

In addition, site investigators are required to submit AE information in accordance with IRB/EC and any applicable RE requirements.

11.2.3 Expedited reporting of adverse events to DAIDS

Requirements, definitions and methods for expedited reporting of AEs are outlined in Version 2.0 (January 2010) of the *Manual for Expedited Reporting of Adverse Events* to DAIDS (DAIDS EAE Manual), which is available on the RSC website at http://rsc.tech-res.com/safetyandpharmacovigilance/. The SAE Reporting Category will be used for this study.

The internet-based DAIDS Adverse Event Reporting System (DAERS) must be used for expedited AE reporting to DAIDS. In the event of system outages or technical difficulties, expedited AE reports may be submitted via the DAIDS EAE Form. For questions about DAERS, please contact DAIDS-ESSupport@niaid.nih.gov or from within the DAERS application itself.

Sites where DAERS has not been implemented will submit expedited AE reports by documenting the information on the current DAIDS EAE Form. This form is available on the RSC website: http://rsc.tech-res.com/safetyandpharmacovigilance/. For questions about expedited AE reporting, please contact the RSC (DAIDSRSCSafetyOffice@tech-res.com).

The study products for which expedited reporting are required are:

- VRC01
- Placebo for VRC01
- Sodium chloride placebo

While the participant is in the study reporting period (See Section 3), the SAE Reporting Category will be used.

After the protocol-defined AE reporting period for the study, unless otherwise noted, only Suspected, Unexpected Serious Adverse Reactions as defined in Version 2.0 of the DAIDS EAE Manual must be reported to DAIDS, if the study staff become aware of the events.

The NIAID/DAIDS will report all unexpected SAEs related to the study products observed in this clinical trial to the FDA in accordance with 21 CFR 312.32 (IND Safety

FOR REVIEW ONLY

Reports). However, because safety is a primary study endpoint, the Sponsor Medical Officer will not be unblinded to study treatment assignment when there is an assessment of relatedness of the SAE with the study product(s); and the safety report will be sent to the FDA based on the blinded attribution assessment.

If the PSRT believes unblinding of the site PI to treatment assignment will assist with the clinical management of the SAE, the PSRT will consult the independent HVTN SMB for a recommendation. In the event the HVTN SMB determines that unblinding is indicated, the SMB will inform the site physician of the participant's treatment assignment in such a manner as to maintain the study blind of the PSRT and study team. For additional impact and management of SAEs on the study, refer to Section 11.3.

11.3 Safety pause and prompt PSRT AE review

When a trial is placed on safety pause, all enrollment and administration with the product related to the event that triggered the pause will be held until further notice. The AEs that will lead to a safety pause or prompt HVTN 104 PSRT AE review are summarized in Table 11-1. Study product administrations may be suspended for safety concerns other than those described in the table, or before pause rules are met, if, in the judgment of the HVTN 104 PSRT, participant safety may be threatened. Criteria for an individual participant's departure from the schedule of study product administrations are listed in Section 7.3.

Table 11-1 AE notification and safety pause/AE review rules

Event and relationship to study products	Severity	HVTN CRS action	HVTN Core action
SAE, related	Grade 5 or Grade 4	Phone immediately, email and fax forms immediately[a]	Immediate pause
SAE, not related	Grade 5	Phone immediately, email and fax forms immediately	Immediate HVTN 104 PSRT notification
SAE, related	Grade 3	Email and fax forms immediately	Prompt HVTN 104 PSRT AE review to consider pause
AE[b], related	Grade 4 or 3	Email and fax forms immediately	Prompt HVTN 104 PSRT AE review to consider pause

Phone numbers and email addresses are listed in HVTN 104 Study Specific Procedures, Key Resource Guide.

[b] Does not include subjective reactogenicity symptoms (study product administration site pain, tenderness, fatigue/malaise, myalgia, arthralgia, chills, headache, and nausea).

For all safety pauses, HVTN Core notifies the HVTN 104 PSRT, HVTN Regulatory Affairs, DAIDS Pharmaceutical Affairs Branch (PAB), DAIDS Regulatory Affairs Branch (RAB), DAIDS Safety and Pharmacovigilance Team (SPT), and participating HVTN CRSs. When an immediate safety pause is triggered, HVTN Core also notifies the HVTN SMB.

Once a trial is paused, the HVTN 104 PSRT reviews safety data and decides whether the pause can be lifted or permanent discontinuation of study product administration is appropriate, consulting the SMB if necessary. HVTN Core notifies the participating HVTN CRSs, HVTN Regulatory Affairs, DAIDS PAB, DAIDS RAB, and DAIDS SPT

FOR REVIEW ONLY

of the decision regarding resumption or discontinuation of study product administrations. Based on the HVTN 104 PSRT assessment, DAIDS RAB notifies the FDA as needed.

If an immediate HVTN 104 PSRT notification or prompt HVTN 104 PSRT AE review is triggered, HVTN Core notifies the HVTN 104 PSRT as soon as possible during working hours (US Pacific Time)—or, if the information was received during off hours, by the morning of the next work day. If a prompt HVTN 104 PSRT AE review cannot be completed within 72 hours of notification (excluding weekends and US federal holidays), an automatic safety pause occurs.

The HVTN requires that each CRS submit to its IRB/EC protocol-related safety information (such as IND safety reports, notification of vaccine holds due to the pause rules, and notification of other unplanned safety pauses). CRSs must also follow all applicable RE reporting requirements.

In addition, all other AEs are reviewed routinely by the HVTN 104 PSRT (see Section 11.4.2).

11.4 Review of cumulative safety data

Routine safety review occurs at the start of enrollment and then throughout the study.

Reviews proceed from a standardized set of protocol-specific safety data reports. These reports are produced by the SDMC and include queries to the HVTN CRSs. Events are tracked by internal reports until resolution.

11.4.1 Daily review

Blinded daily safety reviews are routinely conducted by HVTN Core for events requiring expedited reporting to DAIDS, and events that meet safety pause criteria or prompt HVTN 104 PSRT AE review criteria.

11.4.2 Weekly review

During the study product administration phase of the trial, the HVTN 104 PSRT reviews clinical safety reports on a weekly basis and conducts calls to review the data as appropriate. After the study product administrations and the final 2-week safety visits are completed, less frequent reporting and safety reviews may be conducted at the discretion of the HVTN 104 PSRT. HVTN Core reviews reports of clinical and laboratory AEs. Events identified during the review that are considered questionable, inconsistent, or unexplained are referred to the HVTN CRS clinic coordinator for verification.

11.5 Study termination

This study may be terminated early by the determination of the HVTN 104 PSRT, HVTN SMB, FDA, NIH, Office for Human Research Protections (OHRP), or study product developer. In addition, the conduct of this study at an individual HVTN CRS may be terminated by the determination of the IRB/EC and any applicable RE.

FOR REVIEW ONLY

12 Protocol conduct

This protocol and all actions and activities connected with it will be conducted in compliance with the principles of GCP (ICHe6), and according to DAIDS and HVTN policies and procedures as specified in the *HVTN Manual of Operations*, DAIDS Clinical Research Policies and Standard Procedures Documents including procedures for the following:

- Protocol registration, activation, and implementation;
- Informed consent, screening, and enrollment;
- Study participant reimbursement;
- Clinical and safety assessments;
- Safety monitoring and reporting;
- Data collection, documentation, transfer, and storage;
- Participant confidentiality;
- Study follow-up and close-out;
- Unblinding of staff and participants;
- Quality control;
- Protocol monitoring and compliance;
- Advocacy and assistance to participants regarding negative social impacts associated with the vaccine trial;
- Risk reduction counseling;
- Specimen collection, processing, and analysis;
- Ancillary studies, and
- Destruction of specimens.

Any policies or procedures that vary from DAIDS and HVTN standards or require additional instructions (eg, instructions for randomization specific to this study) will be described in the HVTN 104 *Study Specific Procedures.*

FOR REVIEW ONLY

12.1 Social impacts

Participants in this study risk experiencing discrimination or other personal problems, resulting from study participation. The HVTN CRS is obliged to provide advocacy for and assistance to participants regarding these negative social impacts associated with the vaccine trial. If HVTN CRS staff have questions regarding ways to assist a participant dealing with a social impact, a designated NIAID or HVTN Core representative can be contacted.

Social harms are tabulated by the SDMC and are subjected to descriptive analysis. The goal is to reduce their incidence and enhance the ability of study staff to mitigate them when possible.

Summary tables of social impact events will be generated weekly, and made available for review by the protocol chairs, protocol team leader, and the designated NIAID representative.

12.2 Emergency communication with study participants

As in all clinical research, this study may generate a need to reach participants quickly to avoid imminent harm, or to report study findings that may otherwise concern their health or welfare.

When such communication is needed, the CRS will request that its IRB/EC and any applicable RE expedite review of the message. If this review cannot be completed in a timeframe consistent with the urgency of the required communication, the site should contact the participant first, and then notify the IRB/EC and any applicable RE of the matter as soon as possible.

FOR REVIEW ONLY

13 Version history

The Protocol Team may modify the original version of the protocol. Modifications are made to HVTN protocols via clarification memos, letters of amendment, or full protocol amendments.

The version history of, and modifications to, Protocol HVTN 104 are described below.

Protocol history and modifications

Date: March 19, 2015

Protocol version: 2.0
Protocol modification: Full Protocol Amendment 1

Item 1 Added: Group 4 and Group 5 (n=12 each) evaluating IV administration of VRC01 with dosing schedule of 10 mg/kg (Group 4) and 30 mg/kg (Group 5) at M0, M2, and M4.

Item 2 Added: Visits to Groups 1-3 at M6.5, M7, and M7.5 and to Group 2 at D70 to evaluate VRC01 drug levels and function

Item 3 Added: "Conditional" visits to evaluate VRC01 drug levels and collection of mucosal secretions (cervical/vaginal, semen, rectal, saliva)

Item 4 Added: timepoints to Groups 1-3 for collection of mucosal secretions (cervical/vaginal, semen, rectal, saliva)

Item 5 Added: Increased blood draw volumes by 8.5 mLs at every visit in Groups 1-3 to include storage of serum.

Item 6 Added: 42.5mL blood draw for PBMC storage at 3 days post the 2nd infusion/injection for Groups 1-3.

Item 7 Revised: Total number of SC injections to allow up to 4 injections per participant per timepoint

Item 8 Deleted: Language in *Item 17* in Appendix A, SICF because participants in HVTN 104 will not be included in the VISP registry.

Item 9 Revised: Protocol leadership revised in Section 3.1

Item 10 Updated: Relevant protocol sections with new data from the VRC studies and current status of HVTN 104

Item 11 Deleted the acceptability questionnaire from the last scheduled clinic visit in all groups.

Item 12 Note: this questionnaire was added to the last scheduled clinic visit in clarification memo 1 (per Item 12) but is now being deleted with this amendment. (Per Clarification Memo 1) Revised: Appendices H-J, *Procedures at HVTN CRS for Groups 1-3, "Acceptability questionnaire*" row so that the frequency of the acceptability questionnaire administration is limited to only study product administration visits and the last scheduled study visit for each group

Item 13 (Per Clarification Memo 1) Clarified: footnote "h" in Appendices H-J, *Procedures at the HVTN CRS for Groups 1-3*

FOR REVIEW ONLY

Item 14 (Per Clarification Memo 1) Corrected: exclusion criterion #7

Item 15 Removed the behavioral risk assessment in Appendix I and Appendix J at visits 20, 21, 22.

Item 16 Added several new literature references to Section 16

Item 17 Corrected text in section 7.3.1 to clarify receipt of a vaccination and not from the study product infusion.

Item 18 Per review of the revised IB and the protocol by the DAIDS Protocol Pharmacist, several changes have been made throughout Section 8, *Study product preparation and administration*

Item 19 Revised criteria for requiring a Pap smear to apply only for participants 21 years or older who consent to provide cervicovaginal samples

Date: July 29, 2014

Protocol version: 1.0
Protocol modification: Clarification memo 1

Item 1 Revised Appendices H-J, *Procedures at HVTN CRS for Groups 1-3*, "*Acceptability questionnaire*" row so that the frequency of the acceptability questionnaire administration is limited to only study product administration visits and the last scheduled study visit for each group

Item 2 Clarified footnote "h" in Appendices H-J, *Procedures at the HVTN CRS for Groups 1-3*

Item 3 Corrected exclusion criterion #7

Date: April 15, 2014

Protocol version: 1.0
Protocol modification: Original protocol

FOR REVIEW ONLY

14 Document references (other than literature citations)

Other documents referred to in this protocol, and containing information relevant to the conduct of this study, include:

- Assessment of Understanding. Accessible through the HVTN protocol-specific website.
- Current CDC Guidelines. Revised Recommendations for HIV Testing of Adults, Adolescents, and Pregnant Women in Health-Care Settings. Available at http://www.cdc.gov/mmwr/PDF/rr/rr5514.pdf.
- Division of AIDS (DAIDS) Clinical Research Policies and Standard Procedures Documents. Available at http://www3.niaid.nih.gov/research/resources/DAIDSClinRsrch/
- Division of AIDS Protocol Registration Manual. Available at http://www.niaid.nih.gov/LabsAndResources/resources/DAIDSClinRsrch/Documents/prmanual.pdf
- Division of AIDS Table for Grading the Severity of Adult and Pediatric Adverse Events. Version 1.0, December 2004. (Clarification dated August 2009) Available at http://rsc.tech-res.com/safetyandpharmacovigilance/gradingtables.aspx
- The Manual for Expedited Reporting of Adverse Events to DAIDS. Version 2.0, January 2010. Available at http://rsc.tech-res.com/safetyandpharmacovigilance/manualforexpeditedreporting.aspx
- HVTN Certificate of Confidentiality. Accessible through the HVTN website.
- HVTN 104 Special Instructions. Accessible through the HVTN protocol-specific website.
- HVTN 104 Study Specific Procedures. Accessible through the HVTN protocol-specific website.
- HVTN Site Lab Reference Manual. Accessible through the HVTN website.
- HVTN Manual of Operations. Accessible through the HVTN website.
- Dangerous Goods Regulations (updated annually), International Air Transport Association. Available for purchase at http://www.iata.org/ps/publications/dgr/Pages/index.aspx.
- Lab assay algorithm
- HVTN algorithm for diagnosis of HIV infections. Part of the HVTN Site Lab Reference Manual (see above).

FOR REVIEW ONLY

- International Conference on Harmonisation (ICH) E6 (R1), Guideline for Good Clinical Practice: Section 4.8, Informed consent of trial subjects. Available at http://www.ich.org/fileadmin/Public_Web_Site/ICH_Products/Guidelines/Efficacy/E6_R1/Step4/E6_R1__Guideline.pdf

- Participants' Bill of Rights and Responsibilities. Accessible through the HVTN website.

- NIH Guidelines for Research Involving Recombinant DNA Molecules. Available at http://oba.od.nih.gov/rdna/nih_guidelines_oba.html.

- NIH Policy on Reporting Race and Ethnicity Data: Subjects in Clinical Research. Available at http://grants1.nih.gov/grants/guide/notice-files/NOT-OD-01-053.html.

- Pharmacy Guidelines and Instructions for DAIDS Clinical Trials Networks, July 2008.

- Requirements for Source Documentation in DAIDS Funded and/or Sponsored Clinical Trials. Available at https://phacs.nichdclinicalstudies.org/publicDocs/DAIDS_SourceDocPolicy.pdf

- Title 21, Code of Federal Regulations, Part 50. Available at http://ecfr.gpoaccess.gov/cgi/t/text/text-idx?c=ecfr&sid=2e2429c70115b7df5635f222901ae8f7&rgn=div5&view=text&node=21:1.0.1.1.19&idno=21

- Title 45, Code of Federal Regulations, Part 46. Available at http://ecfr.gpoaccess.gov/cgi/t/text/text-idx?c=ecfr&sid=2e2429c70115b7df5635f222901ae8f7&rgn=div5&view=text&node=45:1.0.1.1.25&idno=45

See Section 16 for literature cited in the background and statistics sections of this protocol.

FOR REVIEW ONLY

15 Acronyms and abbreviations

Ab	antibody
Ad	adenovirus
AE	adverse event
ALT	alanine aminotransferase
ANOVA	analysis of variance
ART	antiretroviral therapy
AST	aspartate aminotransferase
AVEG	AIDS Vaccine Evaluation Group
β-HCG	beta human chorionic gonadotropin
BMI	body mass index
CAB	Community Advisory Board
CBC	complete blood count
CDC	US Centers for Disease Control and Prevention
CDR	complementarity-determining region
CFR	Code of Federal Regulations
CIOMS	Council for International Organizations of Medical Sciences
CI	confidence intervals
CRF	case report form
CRPMC	NIAID Clinical Research Products Management Center
CRS*	clinical research site
CTL	cytotoxic T lymphocyte
DAERS	DAIDS Adverse Event Reporting System
DAIDS	Division of AIDS (US NIH)
DHHS	US Department of Health and Human Services
DSMB	NIAID Data and Safety Monitoring Board
EAE	adverse events requiring expedited reporting to DAIDS
EC	Ethics Committee
EIA	enzyme immunoassay
ELISA	enzyme-linked immunosorbent assay
ELISpot	enzyme-linked immunospot
FDA	US Food and Drug Administration
FHCRC	Fred Hutchinson Cancer Research Center
FPR	false positive rate
GCP	Good Clinical Practice
GEE	generalized estimating equation
HAART	highly active antiretroviral therapy
HBsAg	hepatitis B surface antigen
HCV	hepatitis C virus
HIPAA	Health Insurance Portability and Accountability Act

FOR REVIEW ONLY

HIV	human immunodeficiency virus
HLA	human leukocyte antigen
HVTN	HIV Vaccine Trials Network
IB	Investigator’s Brochure
IBC	Institutional Biosafety Committee
IC	inhibitory concentrationICH International Conference on Harmonisation
ICS	intracellular cytokine staining
IFN-γ	interferon gamma
IND	Investigational New Drug
IRB	Institutional Review Board
IUD	intrauterine device
IV	intravenous
LTFU	loss to follow-up
mAb	monoclonal antibody
MAR	missing at random
MCAR	missing completely at random
MMR	measles, mumps, and rubella
nAb	neutralizing antibody
NHP	nonhuman primate
NIAID	National Institute of Allergy and Infectious Diseases (US NIH)
NICD	National Institute for Communicable Diseases (Johannesburg, South Africa)
NIH	US National Institutes of Health
OBA	NIH Office of Biotechnology Activities
OHRP	US Office for Human Research Protections
OPV	oral polio vaccine
PAB	DAIDS Pharmaceutical Affairs Branch
PBMC	peripheral blood mononuclear cell
PBS	phosphate-buffered saline
PCR	polymerase chain reaction
PI	Principal Investigator
PK	pharmacokinetic
PSRT	Protocol Safety Review Team
PTE	potential T-cell epitope
RAB	DAIDS Regulatory Affairs Branch
RAC	NIH Recombinant DNA Advisory Committee
RE	regulatory entity
RSC	DAIDS Regulatory Support Center
RSV	respiratory syncytial virus
SAE	serious adverse event
SC	subcutaneous
SCHARP	Statistical Center for HIV/AIDS Research and Prevention
SHIV	simian-human immunodeficiency virus

FOR REVIEW ONLY

SDMC	statistical and data management center
SFC	spot-forming cell
SFU	spot-forming unit
SIV	simian immunodeficiency virus
SMB	Safety Monitoring Board
SPT	DAIDS Safety and Pharmacovigilance Team
TB	tuberculosis
UW-VSL	University of Washington Virology Specialty Laboratory
VRC	Vaccine Research Center (NIAID)

* CRSs were formerly referred to as HIV Vaccine Trial Units (HVTUs). Conversion to use of the term CRS is in process, and some HVTN documents may still refer to HVTUs.

FOR REVIEW ONLY

16 Literature cited

1. UNAIDS. Ethical considerations in biomedical HIV prevention trials. **2007**.

2. The National Commission for the Protection of Human Subjects of Biomedical and Behavioral Research. The Belmont Report: Ethical Principles and Guidelines for the Protection of Human Subjects of Research. **1979**.

3. Council for International Organizations of Medical Sciences (CIOMS). International ethical guidelines for biomedical research involving human subjects. Bull Med Ethics **2002**;17-23.

4. UNAIDS. Report on the Global AIDS Epidemic, 2012 country progress reports. http://www.unaids.org/en/dataanalysis/knowyourresponse/countryprogressreports/2012countries/. 2013. Last accessed: 2-20-2013

5. Rerks-Ngarm S, Pitisuttithum P, Nitayaphan S, Kaewkungwal J, Chiu J, Paris R, Premsri N, Namwat C, de Souza M, Adams E, Benenson M, Gurunathan S, Tartaglia J, McNeil JG, Francis DP, Stablein D, Birx DL, Chunsuttiwat S, Khamboonruang C, Thongcharoen P, Robb ML, Michael NL, Kunasol P, Kim JH. Vaccination with ALVAC and AIDSVAX to prevent HIV-1 infection in Thailand. N Engl J Med **2009**;361:2209-20.

6. Haynes BF, Gilbert PB, McElrath MJ, Zolla-Pazner S, Tomaras GD, Alam SM, Evans DT, Montefiori DC, Karnasuta C, Sutthent R, Liao HX, DeVico AL, Lewis GK, Williams C, Pinter A, Fong Y, Janes H, DeCamp A, Huang Y, Rao M, Billings E, Karasavvas N, Robb ML, Ngauy V, de Souza MS, Paris R, Ferrari G, Bailer RT, Soderberg KA, Andrews C, Berman PW, Frahm N, De Rosa SC, Alpert MD, Yates NL, Shen X, Koup RA, Pitisuttithum P, Kaewkungwal J, Nitayaphan S, Rerks-Ngarm S, Michael NL, Kim JH. Immune-correlates analysis of an HIV-1 vaccine efficacy trial. N Engl J Med **2012**;366:1275-86.

7. Ferrantelli F, Rasmussen RA, Buckley KA, Li PL, Wang T, Montefiori DC, Katinger H, Stiegler G, Anderson DC, McClure HM, Ruprecht RM. Complete protection of neonatal rhesus macaques against oral exposure to pathogenic simian-human immunodeficiency virus by human anti-HIV monoclonal antibodies. J Infect Dis **2004**;189:2167-73.

8. Burton DR, Desrosiers RC, Doms RW, Koff WC, Kwong PD, Moore JP, Nabel GJ, Sodroski J, Wilson IA, Wyatt RT. HIV vaccine design and the neutralizing antibody problem. Nat Immunol **2004**;5:233-6.

9. Haynes BF, Montefiori DC. Aiming to induce broadly reactive neutralizing antibody responses with HIV-1 vaccine candidates. Expert Rev Vaccines **2006**;5:579-95.

10. Stamatatos L, Morris L, Burton DR, Mascola JR. Neutralizing antibodies generated during natural HIV-1 infection: good news for an HIV-1 vaccine? Nat Med **2009**;15:866-70.

11. Li Y, Migueles SA, Welcher B, Svehla K, Phogat A, Louder MK, Wu X, Shaw GM, Connors M, Wyatt RT, Mascola JR. Broad HIV-1 neutralization mediated by CD4-binding site antibodies. Nat Med **2007**;13:1032-4.

FOR REVIEW ONLY

12. Simek MD, Rida W, Priddy FH, Pung P, Carrow E, Laufer DS, Lehrman JK, Boaz M, Tarragona-Fiol T, Miiro G, Birungi J, Pozniak A, McPhee DA, Manigart O, Karita E, Inwoley A, Jaoko W, Dehovitz J, Bekker LG, Pitisuttithum P, Paris R, Walker LM, Poignard P, Wrin T, Fast PE, Burton DR, Koff WC. Human immunodeficiency virus type 1 elite neutralizers: individuals with broad and potent neutralizing activity identified by using a high-throughput neutralization assay together with an analytical selection algorithm. J Virol **2009**;83:7337-48.

13. Wu X, Yang ZY, Li Y, Hogerkorp CM, Schief WR, Seaman MS, Zhou T, Schmidt SD, Wu L, Xu L, Longo NS, McKee K, O'Dell S, Louder MK, Wycuff DL, Feng Y, Nason M, Doria-Rose N, Connors M, Kwong PD, Roederer M, Wyatt RT, Nabel GJ, Mascola JR. Rational design of envelope identifies broadly neutralizing human monoclonal antibodies to HIV-1. Science **2010**;329:856-61.

14. Zhou T, Georgiev I, Wu X, Yang ZY, Dai K, Finzi A, Kwon YD, Scheid JF, Shi W, Xu L, Yang Y, Zhu J, Nussenzweig MC, Sodroski J, Shapiro L, Nabel GJ, Mascola JR, Kwong PD. Structural basis for broad and potent neutralization of HIV-1 by antibody VRC01. Science **2010**;329:811-7.

15. Kwong PD, Mascola JR, Nabel GJ. Rational design of vaccines to elicit broadly neutralizing antibodies to HIV-1. Cold Spring Harb Perspect Med **2011**;1:a007278.

16. Wu X, Wang C, O'Dell S, Li Y, Keele BF, Yang Z, Imamichi H, Doria-Rose N, Hoxie JA, Connors M, Shaw GM, Wyatt RT, Mascola JR. Selection pressure on HIV-1 envelope by broadly neutralizing antibodies to the conserved CD4-binding site. J Virol **2012**;86:5844-56.

17. Lynch RM, Tran L, Louder MK, Schmidt SD, Cohen M, Dersimonian R, Euler Z, Gray ES, Abdool KS, Kirchherr J, Montefiori DC, Sibeko S, Soderberg K, Tomaras G, Yang ZY, Nabel GJ, Schuitemaker H, Morris L, Haynes BF, Mascola JR. The Development of CD4 Binding Site Antibodies During HIV-1 Infection. J Virol **2012**;86:7588-95.

18. Li Y, O'Dell S, Walker LM, Wu X, Guenaga J, Feng Y, Schmidt SD, McKee K, Louder MK, Ledgerwood JE, Graham BS, Haynes BF, Burton DR, Wyatt RT, Mascola JR. Mechanism of neutralization by the broadly neutralizing HIV-1 monoclonal antibody VRC01. J Virol **2011**;85:8954-67.

19. Ng CT, Jaworski JP, Jayaraman P, Sutton WF, Delio P, Kuller L, Anderson D, Landucci G, Richardson BA, Burton DR, Forthal DN, Haigwood NL. Passive neutralizing antibody controls SHIV viremia and enhances B cell responses in infant macaques. Nat Med **2010**;16:1117-9.

20. Palivizumab, a Humanized Respiratory Syncytial Virus Monoclonal Antibody, Reduces Hospitalization From Respiratory Syncytial Virus Infection in High-risk Infants. Pediatrics **1998**;102:531-7.

21. Misbah S, Sturzenegger MH, Borte M, Shapiro RS, Wasserman RL, Berger M, Ochs HD. Subcutaneous immunoglobulin: opportunities and outlook. Clin Exp Immunol **2009**;158 Suppl 1:51-9.

22. Parren PW, Marx PA, Hessell AJ, Luckay A, Harouse J, Cheng-Mayer C, Moore JP, Burton DR. Antibody protects macaques against vaginal challenge with a pathogenic R5

FOR REVIEW ONLY

simian/human immunodeficiency virus at serum levels giving complete neutralization in vitro. J Virol **2001**;75:8340-7.

23. Mascola JR, Stiegler G, VanCott TC, Katinger H, Carpenter CB, Hanson CE, Beary H, Hayes D, Frankel SS, Birx DL, Lewis MG. Protection of macaques against vaginal transmission of a pathogenic HIV-1/SIV chimeric virus by passive infusion of neutralizing antibodies. Nat Med **2000**;6:207-10.

24. Mascola JR, Lewis MG, Stiegler G, Harris D, VanCott TC, Hayes D, Louder MK, Brown CR, Sapan CV, Frankel SS, Lu Y, Robb ML, Katinger H, Birx DL. Protection of Macaques against pathogenic simian/human immunodeficiency virus 89.6PD by passive transfer of neutralizing antibodies. J Virol **1999**;73:4009-18.

25. Mascola JR. Defining the protective antibody response for HIV-1. Curr Mol Med **2003**;3:209-16.

26. Shibata R, Igarashi T, Haigwood N, Buckler-White A, Ogert R, Ross W, Willey R, Cho MW, Martin MA. Neutralizing antibody directed against the HIV-1 envelope glycoprotein can completely block HIV-1/SIV chimeric virus infections of macaque monkeys. Nat Med **1999**;5:204-10.

27. Baba TW, Liska V, Hofmann-Lehmann R, Vlasak J, Xu W, Ayehunie S, Cavacini LA, Posner MR, Katinger H, Stiegler G, Bernacky BJ, Rizvi TA, Schmidt R, Hill LR, Keeling ME, Lu Y, Wright JE, Chou TC, Ruprecht RM. Human neutralizing monoclonal antibodies of the IgG1 subtype protect against mucosal simian-human immunodeficiency virus infection. Nat Med **2000**;6:200-6.

28. Hofmann-Lehmann R, Vlasak J, Rasmussen RA, Jiang S, Li PL, Baba TW, Montefiori DC, Bernacky BJ, Rizvi TA, Schmidt R, Hill LR, Keeling ME, Katinger H, Stiegler G, Cavacini LA, Posner MR, Ruprecht RM. Postnatal pre- and postexposure passive immunization strategies: protection of neonatal macaques against oral simian-human immunodeficiency virus challenge. J Med Primatol **2002**;31:109-19.

29. Nishimura Y, Igarashi T, Haigwood N, Sadjadpour R, Plishka RJ, Buckler-White A, Shibata R, Martin MA. Determination of a statistically valid neutralization titer in plasma that confers protection against simian-human immunodeficiency virus challenge following passive transfer of high-titered neutralizing antibodies. J Virol **2002**;76:2123-30.

30. Hessell AJ, Rakasz EG, Poignard P, Hangartner L, Landucci G, Forthal DN, Koff WC, Watkins DI, Burton DR. Broadly neutralizing human anti-HIV antibody 2G12 is effective in protection against mucosal SHIV challenge even at low serum neutralizing titers. PLoS Pathog **2009**;5:e1000433.

31. Hessell AJ, Poignard P, Hunter M, Hangartner L, Tehrani DM, Bleeker WK, Parren PW, Marx PA, Burton DR. Effective, low-titer antibody protection against low-dose repeated mucosal SHIV challenge in macaques. Nat Med **2009**;15:951-4.

32. Moldt B, Rakasz EG, Schultz N, Chan-Hui PY, Swiderek K, Weisgrau KL, Piaskowski SM, Bergman Z, Watkins DI, Poignard P, Burton DR. Highly potent HIV-specific antibody neutralization in vitro translates into effective protection against mucosal SHIV challenge in vivo. Proc Natl Acad Sci U S A **2012**;109:18921-5.

FOR REVIEW ONLY

33. Pegu A, Yang ZY, Boyington JC, Wu L, Ko SY, Schmidt SD, McKee K, Kong WP, Shi W, Chen X, Todd JP, Letvin NL, Huang J, Nason MC, Hoxie JA, Kwong PD, Connors M, Rao SS, Mascola JR, Nabel GJ. Neutralizing antibodies to HIV-1 envelope protect more effectively in vivo than those to the CD4 receptor. Sci Transl Med **2014**;6:243ra88.

34. Ko SY, Pegu A, Rudicell RS, Yang ZY, Joyce MG, Chen X, Wang K, Bao S, Kraemer TD, Rath T, Zeng M, Schmidt SD, Todd JP, Penzak SR, Saunders KO, Nason MC, Haase AT, Rao SS, Blumberg RS, Mascola JR, Nabel GJ. Enhanced neonatal Fc receptor function improves protection against primate SHIV infection. Nature **2014**;514:642-5.

35. Rudicell RS, Kwon YD, Ko SY, Pegu A, Louder MK, Georgiev IS, Wu X, Zhu J, Boyington JC, Chen X, Shi W, Yang ZY, Doria-Rose NA, McKee K, O'Dell S, Schmidt SD, Chuang GY, Druz A, Soto C, Yang Y, Zhang B, Zhou T, Todd JP, Lloyd KE, Eudailey J, Roberts KE, Donald BR, Bailer RT, Ledgerwood J, Mullikin JC, Shapiro L, Koup RA, Graham BS, Nason MC, Connors M, Haynes BF, Rao SS, Roederer M, Kwong PD, Mascola JR, Nabel GJ. Enhanced potency of a broadly neutralizing HIV-1 antibody in vitro improves protection against lentiviral infection in vivo. J Virol **2014**;88:12669-82.

36. Lowy I, Molrine DC, Leav BA, Blair BM, Baxter R, Gerding DN, Nichol G, Thomas WD, Jr., Leney M, Sloan S, Hay CA, Ambrosino DM. Treatment with monoclonal antibodies against Clostridium difficile toxins. N Engl J Med **2010**;362:197-205.

37. Chung RT, Gordon FD, Curry MP, Schiano TD, Emre S, Corey K, Markmann JF, Hertl M, Pomposelli JJ, Pomfret EA, Florman S, Schilsky M, Broering TJ, Finberg RW, Szabo G, Zamore PD, Khettry U, Babcock GJ, Ambrosino DM, Leav B, Leney M, Smith HL, Molrine DC. Human monoclonal antibody MBL-HCV1 delays HCV viral rebound following liver transplantation: a randomized controlled study. Am J Transplant **2013**;13:1047-54.

38. Groothuis JR. Safety and tolerance of palivizumab administration in a large Northern Hemisphere trial. Northern Hemisphere Expanded Access Study Group. Pediatr Infect Dis J **2001**;20:628-30.

39. Bonsignori M, Alam SM, Liao HX, Verkoczy L, Tomaras GD, Haynes BF, Moody MA. HIV-1 antibodies from infection and vaccination: insights for guiding vaccine design. Trends Microbiol **2012**;20:532-9.

40. Ponce R, Abad L, Amaravadi L, Gelzleichter T, Gore E, Green J, Gupta S, Herzyk D, Hurst C, Ivens IA, Kawabata T, Maier C, Mounho B, Rup B, Shankar G, Smith H, Thomas P, Wierda D. Immunogenicity of biologically-derived therapeutics: assessment and interpretation of nonclinical safety studies. Regul Toxicol Pharmacol **2009**;54:164-82.

41. Lobo ED, Hansen RJ, Balthasar JP. Antibody pharmacokinetics and pharmacodynamics. J Pharm Sci **2004**;93:2645-68.

42. Haynes BF, Fleming J, St Clair EW, Katinger H, Stiegler G, Kunert R, Robinson J, Scearce RM, Plonk K, Staats HF, Ortel TL, Liao HX, Alam SM. Cardiolipin polyspecific autoreactivity in two broadly neutralizing HIV-1 antibodies. Science **2005**;308:1906-8.

43. Hansel TT, Kropshofer H, Singer T, Mitchell JA, George AJ. The safety and side effects of monoclonal antibodies. Nat Rev Drug Discov **2010**;9:325-38.

FOR REVIEW ONLY

44. Bugelski PJ, Achuthanandam R, Capocasale RJ, Treacy G, Bouman-Thio E. Monoclonal antibody-induced cytokine-release syndrome. Expert Rev Clin Immunol **2009**;5:499-521.

45. Vogel WH. Infusion reactions: diagnosis, assessment, and management. Clin J Oncol Nurs **2010**;14:E10-E21.

46. Haddad E, Barnes D, Kafal A. Home therapy with subcutaneous immunoglobulins for patients with primary immunodeficiency diseases. Transfus Apher Sci **2012**;46:315-21.

47. Agresti A, Coull BA. Approximate is better than "exact" for interval estimation of binomial proportions. Am Stat **1998**;52:119-26.

48. Hughes JP. Mixed effects models with censored data with application to HIV RNA levels. Biometrics **1999**;55:625-9.

49. Rotnitzky A, Robins J. Analysis of semi-parametric regression models with non-ignorable non-response. Stat Med **1997**;16:81-102.

50. Montefiori DC. Measuring HIV neutralization in a luciferase reporter gene assay. Methods Mol Biol **2009**;485:395-405.

51. Todd CA, Greene KM, Yu X, Ozaki DA, Gao H, Huang Y, Wang M, Li G, Brown R, Wood B, D'Souza MP, Gilbert P, Montefiori DC, Sarzotti-Kelsoe M. Development and implementation of an international proficiency testing program for a neutralizing antibody assay for HIV-1 in TZM-bl cells. J Immunol Methods **2012**;375:57-67.

52. Ozaki DA, Gao H, Todd CA, Greene KM, Montefiori DC, Sarzotti-Kelsoe M. International technology transfer of a GCLP-compliant HIV-1 neutralizing antibody assay for human clinical trials. PLoS ONE **2012**;7:e30963.

FOR REVIEW ONLY

Appendix A Sample informed consent form

Title: A phase 1 clinical trial to evaluate the safety and drug levels of a human monoclonal antibody, VRC-HIVMAB060-00-AB (VRC01) administered in multiple doses intravenously and subcutaneously in different dosing schedules to healthy, HIV-uninfected adults

HVTN protocol number: HVTN 104

Site: [Insert site name]

Thank you for your interest in our research study. Please read this consent form or ask someone to read it to you. If you decide to join the study, we will ask you to sign or make your mark on this form. We will offer you a copy to keep. We will ask you questions to see if we have explained everything clearly. You can also ask us questions about the study.

Research is not the same as treatment or medical care. The purpose of a research study is to answer scientific questions.

About the study

The HIV Vaccine Trials Network (HVTN) and [Insert site name] are doing a study to test an antibody against HIV. HIV is the virus that causes AIDS. Antibodies are one of the ways the human body fights infection. Antibodies are natural proteins that the body can make to prevent infectious agents such as bacteria and viruses from making you sick. Antibodies can also be manufactured like a drug and infused or injected into the body. This approach has been successfully used to prevent or treat some other diseases.

About 88 people will take part in this study at multiple sites. The researcher in charge of this study at this clinic is [Insert name of site PI]. The US National Institutes of Health (NIH) is paying for the study.

1. We are doing this study to answer several questions.

- Are the study products safe to give to people?
- Are people able to take the study products without becoming too uncomfortable?
- How do people's bodies respond to the study products?
- How much of the antibody remains in your body as time passes?
- How does the body's response to the study products change depending on the amount and timing of the doses?
- Does the method of giving the antibody change the body's response?

2. The study products cannot give you HIV.

It is impossible for the study products to give you HIV. Also, they cannot cause you to give HIV to someone else. However, we do not know if the study products will decrease,

FOR REVIEW ONLY

increase, or not change your chance of becoming infected with HIV if you are exposed to the virus.

3. These study products are experimental.

The antibody being tested is called VRC-HIVMAB060-00-AB. It is an antibody against the HIV virus. From here on, we will call it VRC01 or the antibody. We will also be testing the placebo. The placebo is made from inactive ingredients made to look like the antibody. Together we will call them the study products.

They are experimental. That means we do not know if they will be safe to use in people, or if the antibody will work to prevent HIV infection. They are used only in research studies.

The study products were developed by the NIH. They were both made using the controlled, sterile conditions used for drug manufacturing.

In laboratory and animal studies, VRC01 attached to and disabled many kinds of HIV viruses. We do not know if the antibody will act the same way when given to people. It will take many studies to learn if the products will be useful for prevention of HIV or treatment of HIV. This study alone will not answer these questions.

Risks of VRC01:

VRC01 has been given to more than 40 participants in clinical trials at the NIH Clinical Center. The study products have been tested in one study with HIV-infected participants and in one study with HIV-uninfected participants. Over 40 participants have received VRC01 in HVTN 104 also. So far the antibody is generally well tolerated; the majority of participants had no or mild side effects; a few reported moderate or severe subjective symptoms such as malaise/fatigue or muscle aches. When injected into skin there may be mild redness, swelling, and itching at the injection site that resolves within a few minutes to hours. Some participants had a temporary change in a laboratory test that required additional tests. These lab changes did not cause symptoms and went away on their own.

Two participants had some mild side effects after injections. One person had a mild itchy rash in several places a few days after the injection. The rash lasted a few hours and went away on its own. The other person had a tight feeling in their chest and some wheezing that started shortly after the injection and went away on its own. We do not know yet if these participants got VRC01 or the placebo, so we decided not to give them any more of the study products.

With antibody products, most side effects tend to occur within the first 24 hours.

In general, side effects of antibody products are mild, but may include fever, chills, shaking, nausea, vomiting, pain, headache, dizziness, trouble breathing, high or low blood pressure, itchiness, rash, hives, lip or face swelling, diarrhea, racing heartbeat or chest pain.

In a study where VRC01 was given to animals, there was a small increase in two liver lab tests. This increase lasted for a short period of time. No sign of liver damage was seen in

FOR REVIEW ONLY

the animals. There were no abnormal findings in the animals' organs except for irritation at the location where the antibody was given.

VRC01 may have other side effects that we do not know about yet.

Some antibody products have a small risk of causing serious drug reactions. These reactions may be life-threatening.

- One type of reaction may occur soon after an antibody product is given. It includes difficulty breathing possibly leading to low blood oxygen, low blood pressure, hives or rash, and/or swelling in the mouth and face.

- A second type of reaction may occur several days to three weeks after an antibody product is given. It includes having hives or a rash, fever, big lymph nodes, muscle pains, joint pains, chest discomfort and shortness of breath.

When antibodies are given to a person by infusion or injection they do not last in the body more than a few months. Any antibody given to you in this study will be gone from your body several months after your last dose.

Risks of placebo:

The placebo does not contain antibodies and is made of inactive ingredients. These are generally recognized as safe but there may be unknown risks associated with the placebo.

Joining the study

4. It is completely up to you whether or not to join the study.

Take your time in deciding. If it helps, talk to people you trust, such as your doctor, friends or family. If you decide not to join this study, or if you leave it after you have joined, your other care at this clinic and the benefits or rights you would normally have will not be affected.

If you join this study, you may not be allowed to join other HIV vaccine or HIV prevention studies now or in the future. You cannot be in this study while you are in another study where you receive a study product. Also during the study, you should not donate blood or tissue.

If you choose not to join this study, you may be able to join another study.

Site: Remove item 5 if you use a separate screening consent that covers these procedures.

5. If you decide to join the study, we will screen you to see if you are eligible.

Screening involves a physical exam, HIV test and health history. A physical exam may include, but is not limited to:

- Checking your weight, and height, temperature and blood pressure

- Looking in your mouth and throat

FOR REVIEW ONLY

- Listening to your heart and lungs
- Feeling your abdomen (stomach and liver)
- Checking your veins to assess how easy it might be to start an IV

We will also do blood and urine tests. These tests tell us about some aspects of your health, such as how healthy your kidneys, liver, and immune system are. We will ask you about medications you are taking. We will also test you for syphilis, Hepatitis B, and Hepatitis C. We will ask you about behaviors that might put you at risk for getting HIV. If you were born female, we will test you for pregnancy. People who have had a complete hysterectomy (removal of the uterus and ovaries, verified by medical records), are not required to have a pregnancy test.

We will review the screening results with you, and offer you counseling and referral if you need medical care. We will not pay for this medical care. The screening results may show you are not eligible to join the study, even if you want to.

6. **If you were born female and could become pregnant, you must agree to use birth control to join this study.**

Site: List approved birth control methods here if you do not want to hand out the separate Approved Birth Control Methods sheet.

You should not become pregnant during the study because we do not know how the study products could affect the developing baby. You must agree to use effective birth control from 21 days before you first receive study products until your last required clinic visit. We will talk to you about effective birth control methods. They are listed on a handout that we will give to you. *Site: Delete the preceding sentence if you include the birth control sheet in this consent form.* If you join the study, we will test you for pregnancy at some visits.

Being in the study

If you meet the study requirements and want to join, here is what will happen:

7. **You will come to the clinic for scheduled visits about 15-21 times over about 8 months depending on which group you are in.**

Site: Insert number of visits and range of visit lengths. (There is site-specific variation in screening protocols and in the number of possible follow-up visits between protocol-mandated visits.)

Visits can last from [#] to [#] hours.

You may have to come for more visits if you have a lab or health issue.

We may contact you after the main study ends (for example, to tell you about the study results).

FOR REVIEW ONLY

8. We will give you [Site: Insert compensation] for each study visit you complete.

This amount is to cover the costs of [Site: Insert text]

Site: Insert any costs to participants (eg, birth control costs for female participants who could become pregnant).

Payments you receive for being in the study may be taxable. This happens if we pay you more than $600 between January 1 and December 31 of the same year. We may need to ask you for your Social Security number for tax reasons.

You do not have to pay anything to be in this study.

9. We will give you the study products on a schedule.

You will be in one of 5 groups. Each group will have a different schedule for getting the antibody or placebo and will receive different doses of the antibody or placebo.

Groups 1, 2, 4, and 5

People in Groups 1, 2, 4, and 5 will get all of their doses by intravenous (IV) infusion. IV infusion is done by putting a needle into a vein in your arm.

- Each IV infusion will take about one hour.
- You will have to wait in the clinic for about another hour after each infusion to be sure you don't have any problems.

Group 3

People in Group 3 will get their first dose by IV infusion but the other doses by subcutaneous (SC) injection. SC injections are done by putting a needle under the skin on your arm, abdomen or thigh.

- The first dose by IV infusion will take about one hour.
- You will have to wait in the clinic for about another hour after the infusion to be sure you don't have any problems.
- The SC injections will only take a few minutes.
- Depending on the size of the dose, we may need to give you 1-4 SC injections at each visit.
- You will have to wait in the clinic for about an hour after the first SC injection to be sure you don't have any problems.
- If you don't have any problems with the first SC injection, you will only have to wait in the clinic for about ½ hour after the rest of the SC injections.

FOR REVIEW ONLY

The high, medium, and low doses of the study products that are used in all groups will be adjusted for your body weight. We will weigh you on the day of each dose to determine the amount you will get.

**Groups**		**Infusion and Injection Schedules**											
		**1st visit**	**Time after the 1st visit**										
**Group**	**Number of people**		**2 weeks**	**1 month**	**1.5 months**	**2 months**	**2.5 months**	**3 months**	**3.5 months**	**4 months**	**4.5 months**	**5 months**	**5.5 months**
1	20	VRC01 high dose by IV		VRC01 medium dose by IV		VRC01 medium dose by IV		VRC01 medium dose by IV		VRC01 medium dose by IV		VRC01 medium dose by IV	
2	20	VRC01 high dose by IV				VRC01 high dose by IV				VRC01 high dose by IV			
3	20	VRC01 high dose by IV	VRC01 low dose by SC	VRC01 low dose by SC	VRC01 low dose by SC	VRC01 low dose by SC	VRC01 low dose by SC	VRC01 low dose by SC	VRC01 low dose by SC	VRC01 low dose by SC	VRC01 low dose by SC	VRC01 low dose by SC	VRC01 low dose by SC
	4	placebo IV	placebo SC	placebo SC	placebo SC	placebo SC	placebo SC	placebo SC	placebo SC	placebo SC	placebo SC	placebo SC	placebo SC
4	12	VRC01 low dose by IV				VRC01 low dose by IV				VRC01 low dose by IV			
5	12	VRC01 medium dose by IV				VRC01 medium dose by IV				VRC01 medium dose by IV			
Total	88												

The night after each infusion or injection, and for three more days, you will need to write down how you are feeling and if you have any symptoms. Contact the clinic staff if you have any issues or concerns after receiving an infusion or injection. If you have a problem, we will continue to check on you until it goes away.

10. We will give you either the antibody or the placebo.

Not everyone in this study will get the antibody.

Groups 1, 2, 4, and 5:
All of the people in these groups will get the antibody.

Group 3:
20 of the people in group 3 will get the antibody. The other 4 people in group 3 will get the placebo.

In Group 3, there is one placebo for the infusion given by IV, and another placebo for the SC injection. Neither you nor the clinic staff will know if you are getting the antibody or placebo. Only the pharmacist at your site will have this information while the study is going on. We will compare the results from people who got the placebo with results from people who got the antibody.

We will also compare the results from people who got the antibody in all 5 groups.

If you are in Group 3, you will have to wait until everyone completes their final study visits to find out whether you got the antibody or the placebos. This could be more than a

FOR REVIEW ONLY

year. But, if you have a serious medical problem and need to know what you got before the end of the study, we can tell you.

11. You will be assigned to a group.

Based on your screening visit and schedule, you and the study staff will discuss which group is best for you. Group 3 has more study visits, so you will need to decide if this fits your schedule. If you prefer a less frequent visit schedule, we will assign you to Group 1, 2, 4, or 5 randomly, like flipping a coin. You will be in the same group the whole time you are in the study.

12. In addition to giving you the antibody or placebo, we will:

- Do regular HIV testing, as well as counseling on your results and on how to avoid getting HIV;
- Perform physical exams;
- Take blood and urine samples;
- Do pregnancy tests if you were born female; people who have had a complete hysterectomy (removal of the uterus and ovaries, verified by medical records), are not required to have pregnancy tests;
- Ask questions about your health, including medications you may be taking;
- Ask questions about your experience of getting the antibody or placebo;
- Ask questions about any personal problems or benefits you may have from being in the study.

When we take blood, the amount will depend on the lab tests we need to do. It will be some amount between 20 mL and 120 mL (a little more than 1 tablespoon to about ½ cup). Your body will make new blood to replace the blood we take out.

Site: You may want to add a sentence to the end of the previous paragraph contextualizing the blood volumes described (eg, "To compare, people who donate blood in the US can give a total of about 500 mL in an 8-week period."). Modify the example for cultural relevance and alter blood volumes as necessary.

Site: Paste table of procedures in this section or distribute it as a separate sheet if it is helpful to your study participants.

We will be looking for side effects. We will review the results of these procedures and tests with you at your next visit, or sooner if necessary. If any of the results are important to your health, we will tell you. We will also offer you counseling and referral for needed care.

FOR REVIEW ONLY

13. If you agree, we will also collect saliva, rectal and semen or cervical samples.

Because most people are exposed to HIV in their mouth, or on their rectum, penis, or vagina, it is important to learn if antibodies are found in these locations after getting infusions or injections. For this reason, we want to collect saliva, rectal and semen or cervical samples to look for antibodies. Depending on which group you are in, we will collect these samples at 7 or 8 visits. We will do this only if you agree and are able to provide the samples.

At the end of this consent form, we will ask if you allow us to collect these samples. You can decide not to give us these samples and still be in the study. You can decide to provide some of these samples and not others. If you agree to provide these samples, you can change your mind at any time during the study.

About saliva samples

For participants who agree to give saliva samples, we will ask you to spit into a container. We want to collect about a teaspoon of saliva. Please avoid the following for 1 hour before your visit:

- smoking,
- eating,
- chewing gum or tobacco,
- drinking anything but water,
- intimate oral activity, and
- brushing your teeth or using mouthwash.

About rectal samples

For participants who agree to give rectal samples, we will collect rectal fluids by placing a small absorbent sponge in the rectum using a plastic tube about as wide as a pencil. This will take about 5 minutes. We will not collect the sample if you have an active infection, inflamed hemorrhoids, or colitis/diarrhea. We will:

- perform a pregnancy test for participants born female; people who have had a total hysterectomy (removal of the uterus and ovaries, verified by medical records), are not required to undergo pregnancy testing. We do not do most study procedures on pregnant women, so we will do a pregnancy test first.
- not collect the sample if you have had receptive anal intercourse or inserted anything into your anus for 48 hours (2 days) before the visit.
- not collect the sample if you have used steroids or other anti-inflammatory creams in or around your anus for 48 hours (2 days) before the visit.

FOR REVIEW ONLY

About semen samples

For participants who were born male and who agree to give semen samples, you may provide the samples at home or at the clinic. We will ask you:

- not to ejaculate for 48 hours (2 days) before the visit. This will help make sure the samples you provide give accurate lab readings.
- to ejaculate into a plastic cup that we will give to you.
- to bring the semen sample to the clinic within 2 hours after collection, if the sample is collected outside of the clinic.

About cervical samples

For participants who were born female and who agree, we will collect cervical fluid. To collect cervical fluid, we will insert a speculum (a device that opens the vagina) into your vagina. Then we will place a small sponge in the opening of the cervix for about a minute to absorb the fluid. We will not collect the sample if you have any active genital infections or sores. We will:

- perform a pregnancy test for participants born female; persons who have had a total hysterectomy (removal of the uterus and ovaries, verified by medical records), are not required to undergo pregnancy testing. We do not do most study procedures on pregnant women, so we will do a pregnancy test first.
- not collect the sample if you are menstruating.
- not collect the sample if you have had vaginal intercourse or inserted anything into your vagina for 48 hours (2 days) before the visit.
- not collect the sample if you have used any spermicide, lubricants or topical/intravaginal medications (such as topical yeast infection treatments), including douching, within 48 hours (2 days) before the visit.
- require a Pap smear if you have not had one within 3 years before enrollment (depending on your age), with the latest result reported as normal. We can give you a Pap smear if you have not had one within that timeframe.

14. We will counsel you on avoiding HIV infection.

We will ask you personal questions about your HIV risk factors such as sexual behavior and drug use. We will talk with you about ways to keep your risk of getting HIV low. Some topics we may discuss include:

- What you think may cause risky behavior for you.
- Methods to avoid getting HIV.

FOR REVIEW ONLY

These may include not having sex, using condoms, or behavior changes, such as cutting down on alcohol. We will talk with you about new methods of HIV prevention and can give you information on how to access them.

15. We will test your samples for this study.

We will send your samples (without your name) to a lab to see how your body responds to the antibody or placebo. The researchers may:

- Take cells from your samples and grow more of them. We may grow more of your cells over time, so that they can continue to contribute to this study.
- Do limited genetic testing. Your genes are passed to you from your birth parents. They affect how you look and how your body works. The difference in people's genes can help explain why some people get a disease while others do not. Limited genetic testing involves only some of your genes, not all of your genes (your genome). The researchers will not look at all of your genes, only the genes related to the immune system and diseases.

These tests are for research purposes only. The lab will not give the results to you or this clinic, and the results will not become part of your study record.

Site: Delete next section if using separate Other Use of Specimen consent

16. When we take samples from you for this study, we take extra samples in case we have to repeat tests. When samples are no longer needed for this study, the HVTN wants to keep them for use in other studies. We will call these "extra samples."

This section gives you information so you can decide if you want your extra samples and information used in other studies. You will mark your decision at the end of the form. If you have any questions, please ask.

Do I have to agree? No. You are free to say yes or no, or to change your mind after you sign this form. At your request, we will destroy all samples that we have. Your decision will not affect your being in this study or have any negative consequences here.

Where are the samples stored? Extra samples are stored in a secure central place called a repository. *[Site: insert specific information if your regulatory authority requires it.]* The central repositories for the HVTN are located in the United States.

How long will the samples be stored? There is no limit on how long your extra samples will be stored. *[Site: insert limits if your regulatory authority imposes them.]*

Will I be paid for the use of my samples? No. Also, a researcher may make a new scientific discovery or product based on the use of your samples. If this happens, there is no plan to share any money with you. The researcher is not likely to ever know who you are.

Will I benefit from allowing my samples to be used in other studies? Probably not. Results from these other studies are not given to you, this clinic, or your doctor. They are not needed for your medical care. They are not part of your medical record. The studies are only being done for research purposes.

FOR REVIEW ONLY

Will the HVTN sell my samples and information? No, but the HVTN may share your samples with other researchers. Once we share your samples and information, we will not be able to get them back.

How do other researchers get my samples and information? When a researcher wants to use your samples and/or information, their research plan must be approved by the HVTN. Also, the researcher's institutional review board (IRB) or ethics committee (EC) will review their plan. *[Site: insert review by your institution's IRB/EC, if applicable.]* IRBs/ECs protect the rights and well-being of people in research. The HVTN keeps track of your decision about how your samples and information can be used.

What information is shared with other researchers? The samples and limited information will be labeled with a code number. Your name will not be part of the information. However, some information that we share may be personal, such as your race, ethnicity, gender, health information from the study, and HIV status. We may share information about the study products you received and how your body responded to the study products.

What kind of studies might be done with my extra samples and information? The studies will be related to HIV, vaccines, the immune system and other diseases. The researchers may:

- Take cells from your samples and grow more of them. This means the researchers may keep your cells growing over time.

- Do limited genetic testing, which involves only looking at some of your genes, not all of your genes.

If you agree, your samples could also be used for genome wide studies. In these studies, researchers will look at all of your genes (your genome). The researchers compare the genomes of many people, looking for common patterns of genes that could help them understand diseases. The researchers may put the information from the genome-wide studies into a protected database so that other researchers can access it. Usually, no one would be able to look at your genome and link it to you as a person. However, if another database exists that also has information on your genome and your name, someone might be able to compare the databases and identify you. If others found out, it could lead to discrimination or other problems. The risk of this is very small.

Who will have access to my information in studies using my extra samples?

People who may see your information are:

- Researchers who use your stored samples and limited information for other research

- Government agencies that fund or monitor the research using your samples or information

- The researcher's Institutional Review Board or Ethics Committee

- The people who work with the researcher

FOR REVIEW ONLY

All of these people will do their best to protect your information. The results of any new studies that use your extra samples or information may be published. No publication will use your name or identify you personally.

17. We will do our best to protect your private information.

Sites: Check HIPAA authorization for conflicts with this section.

Your study records and samples will be kept in a secure location. We will label all of your samples and most of your records with a code number, not your name or other personal information. However, it is possible to identify you, if necessary. We will not share your name with the lab that does the tests on your samples, or with anyone else who does not need to know your name.

Clinic staff will have access to your study records. Your records may also be reviewed by groups who watch over this study to see that we are protecting your rights, keeping you safe, and following the study plan. These groups include:

- The US National Institutes of Health and its study monitors,
- The US Food and Drug Administration,
- [Insert name of local IRB/EC] ,
- [Insert name of local and/or national regulatory authority as appropriate],
- National Institutes of Health and people who work for them,
- The HVTN and people who work for them,
- The HVTN Safety Monitoring Board, and,
- The US Office for Human Research Protections.

All reviewers will take steps to keep your records private.

We cannot guarantee absolute privacy. At this clinic, we have to report the following information:

Site: Include any public health or legal reporting requirements. Bulleted examples should include all appropriate cases (reportable communicable disease, risk of harm to self or others, etc.).

- [Item 1]
- [Item 2]
- [Item 3]

We have a Certificate of Confidentiality from the US government, to help protect your privacy. With the certificate, we do not have to release information about you to someone

FOR REVIEW ONLY

who is not connected to the study, such as the courts or police. Sometimes we can't use the certificate. Since the US government funds this research, we cannot withhold information from it. Also, you can still release information about yourself and your study participation to others.

The results of this study may be published. No publication will use your name or identify you personally.

We may share information from the study with other researchers. We will not share your name or information that can identify you.

When the study is done, we may share the information from the study with others so they can see it and use it. We will not share any information that will let someone identify you.

18. We may stop your infusions or injections or take you out of the study at any time. We may do this even if you want to stay in the study and even if you were scheduled for additional infusions or injections.

This may happen if:

- you do not follow instructions,
- the researcher thinks that staying in the study might harm you,
- you get HIV,
- you enroll in a different research study where you receive another study product, or
- the study is stopped for any reason.

If we stop your infusions or injections, we may ask you to stay in the study to complete other study procedures.

19. If you become pregnant during the study, we will continue with some procedures but not infusions or injections.

We will do this for as long as it is safe for you and your developing baby.

If you leave the study while you are still pregnant, we will contact you after your due date to ask some questions about your pregnancy and delivery.

20. If you get infected with HIV during the study, we will help you get care and support.

You will not be able to stay in this study. We will counsel you about your HIV infection and about telling your partner(s). We will tell you where you can get support and medical care, and about other studies you may want to join. *Site: Modify the following sentence as appropriate.* We will not provide or pay for any of your HIV care directly.

FOR REVIEW ONLY

Other Risks

21. There are other risks to being in this study.

This section describes the other risks and restrictions we know about. There may also be unknown risks, even serious ones. We will tell you if we learn anything new that may affect your willingness to stay in the study.

Risks of routine medical procedures:

In this study, we will do some routine medical procedures like taking blood. These procedures can cause bleeding, bruising, pain, fainting, soreness, redness, swelling, itching, muscle damage, and (rarely) infection where the needle was inserted or blood clot. Taking blood can cause a low blood cell count (anemia), making you feel tired.

Risks of IV infusion and SC injection procedures:

Receiving an infusion or injection through a needle may cause stinging, discomfort, pain, soreness, redness, bruising, itching, rash and swelling at the location where the needle goes into the skin. Rarely, needlesticks can result in infections.

Risks of sampling saliva, rectal and genital fluids

We will ask you to stop some behaviors related to your mouth, rectum, and genitals for a short time before we collect samples from these areas. You may find this inconvenient. These sample collections may also cause some anxiety, temporary discomfort, and embarrassment. For women, the collection of cervical fluid may cause discomfort similar to what happens during a Pap smear. We will try to make you as comfortable as possible.

Personal problems/discrimination/testing HIV antibody positive:

About 10 to 20% of people who join HVTN studies report personal problems or discrimination because of joining an HIV vaccine study. Although this is not a vaccine study, it may raise similar concerns. Family or friends may worry, get upset or angry, or assume that you are infected with HIV or at high risk and treat you unfairly as a result. Rarely, a person has lost a job because the study took too much time away from work, or because their employer thought they had HIV.

If someone assumes you are infected with HIV, even if you are not, you could face discrimination and other problems. For example, you could be denied medical or dental care, employment, insurance, a visa, or entry into the military. Your family and friends may treat you differently.

An HIV antibody test is the usual way to test for HIV infections. In HIV vaccine studies, getting the study vaccine can cause you to test positive on some types of HIV antibody tests. This study is different, because you will not get an HIV vaccine. Based on lab tests, we do not expect the study product to cause a positive result on standard HIV antibody tests. However, we still ask you to get HIV tests only at this clinic during the study. Our tests can always tell the difference between true HIV infection and a positive result that is caused by a study product.

FOR REVIEW ONLY

If you become pregnant while you still have the antibody in your body, we don't know if this antibody could be passed to your baby.

Embarrassment/anxiety:

You may feel embarrassed when we ask about your HIV risks, such as having sex and using drugs. Also, waiting for your HIV test results or other health test results could make you feel anxious. You could feel worried if your test results show that you are infected with HIV. If you feel embarrassed or anxious, please tell us and we will try to help you.

Risks of disclosure of your personal information:

We will take several steps to protect your personal information. Although the risk is very low, it is possible that your personal information could be given to someone who should not have it. If that happened, you could face discrimination, stress, and embarrassment. We can tell you more about how we will protect your personal information if you would like it.

Risks of genetic testing:

The genetic testing could show you may be at risk for certain diseases. If others found out, it could lead to discrimination or other problems. However, it is almost impossible for you or others to know your test results from the genetic testing. The results are not part of your study records and are not given to you.

In the very unlikely event that your genetic information becomes linked to your name, a federal law called the Genetic Information Nondiscrimination Act (GINA) helps protect you. GINA keeps health insurance companies and employers from seeing results of genetic testing when deciding about giving you health insurance or offering you work. GINA does not help or protect you against discrimination by companies that sell life, disability or long-term care insurance.

Unknown risks:

We do not know if the study products will increase, decrease, or not change your risk of becoming infected with HIV if you are exposed to the virus. If you get infected with HIV, we do not know how the study products might affect your HIV infection or how long it takes to develop AIDS.

We do not know if getting these study products will affect how you respond to a future approved HIV vaccine. It could be that a future HIV vaccine may not work as well for you because you got these study products. Currently, no HIV vaccine has been approved for use.

We do not know how the study products will affect a pregnant participant or a developing baby.

FOR REVIEW ONLY

Benefits

22. The study may not benefit you.

We do not know whether getting the study products might benefit you in any way. However, being in the study might still help you in some ways. The counseling that you get as part of the study may help you avoid getting HIV. The lab tests and physical exams that you get while in this study might detect health problems you don’t yet know about.

This study may help in the search for a way to prevent HIV. However, if the study products later become approved and sold, there are no plans to share any money with you.

Your rights and responsibilities

23. If you join the study, you have rights and responsibilities.

You have many rights that we will respect. You also have responsibilities. We list these in the Participant’s Bill of Rights and Responsibilities. It was written for participants in HIV vaccine studies. We will give you a copy of it and will tell you how some of the rights and responsibilities are different because you are not getting a vaccine in this study.

Leaving the study

24. Tell us if you decide to leave the study.

You are free to leave the study at any time and for any reason. Your care at this clinic and your legal rights will not be affected, but it is important for you to let us know.

We will ask you to come back to the clinic one last time for a physical exam, and we may ask to take some blood and urine samples. We will also ask about any personal problems or benefits you have experienced from being in the study. We believe these steps are important to protecting your health, but it is up to you whether to complete them.

Injuries

25. If you get sick or injured during the study, contact us immediately.

Your health is important to us. We will help you get the medical care you need.

You could get sick or injured by the study products and/or procedures. If this happens, the HVTN has limited funds from the U.S. government to pay for your treatment.

If someone gets sick or injured in an HVTN study, the HVTN decides whether the injury is probably related to the study products and/or procedures. If the HVTN decides it was more likely due to the study products and/or procedures than any other cause, then the HVTN will use its funds to pay for treatment. The HVTN expects to cover the entire costs for the treatment of simple, temporary study related injuries. If your injuries are more severe or chronic, the HVTN funds may not be enough. If needed, the HVTN will seek more funds, but cannot guarantee them. If the HVTN cannot pay the entire cost of

FOR REVIEW ONLY

your treatment, you or your health insurance company would be responsible for any additional costs. Some health insurance companies will not pay for study related injuries. *[Sites: insert locale- appropriate medical insurance language in the preceding paragraph.]*

If the HVTN decides the injury is likely not due to the study products and/or procedures, then you or your health insurance would be responsible for treatment costs. *[Sites: insert locale- appropriate medical insurance language in the preceding sentence.]*You may disagree with the decision the HVTN makes about your injuries. At your request the HVTN will ask experts who are not connected with the HVTN to review its decision. No matter what, you still have the right to use the court system to address payment for your injuries if you are not satisfied.

Some injuries are not physical. For example, someone might be harmed psychologically or emotionally by being in an HIV related study. Or they might lose wages from injuries because they could not go to work. No funds have been set aside to pay for nonphysical injuries, even if they are related to participation in the study.

Questions

26. If you have questions or problems at any time during your participation in this study, use the following important contacts.

If you have questions about this study, contact [name and telephone number of the investigator or other study staff].

If you have any symptoms that you think may be related to this study, contact [name and telephone number of the investigator or other study staff].

If you have questions about your rights as a research participant, or problems or concerns about how you are being treated in this study, contact [name/title/phone of person on IRB or other appropriate organization].

If you want to leave this study, contact [name and telephone number of the investigator or other study staff].

FOR REVIEW ONLY

Your permissions and signature

27. In Section 13 of this form, we told you about collecting saliva, rectal, and semen or cervical samples. Please write your initials or make your mark in the boxes next to the options you choose.

☐ I agree to provide rectal samples.

☐ I do not agree to provide rectal samples.

☐ I agree to provide semen or cervical samples.

☐ I do not agree to provide semen or cervical samples.

☐ I agree to provide saliva samples.

☐ I do not agree to provide saliva samples.

Site: Delete this section if using a separate consent for Other Use of Specimen

28. In Section 16 of this form, we told you about possible other uses of your extra samples and limited information, outside this study. Please write your initials or make your mark in the box next to the option you choose.

FOR REVIEW ONLY

☐ I allow my extra samples combined with limited information for other studies related to HIV, vaccines, the immune system, and other diseases. This may include limited genetic testing and keeping my cells growing over time.

OR

☐ I agree to the option above and also to allow my extra samples combined with limited information to be used in the genome wide studies.

OR

☐ I do not allow my extra samples to be used in any other studies. This includes not allowing limited genetic testing, growing more of my cells, or genome wide studies.

29. If you agree to join this study, you will need to sign or make your mark below. Before you sign or make your mark on this consent form, make sure of the following:

- You have read this consent form, or someone has read it to you.
- You feel that you understand what the study is about and what will happen to you if you join. You understand what the possible risks and benefits are.
- You have had your questions answered and know that you can ask more.
- You agree to join this study.

You will not be giving up any of your rights by signing this consent form.

Participant's name (print)	Participant's signature or mark	Date	Time
Clinic staff conducting consent discussion (print)	Clinic staff signature	Date	Time

For participants who are unable to read or write, a witness should complete the signature block below:

Witness's name (print)	Witness's signature	Date	Time

*Witness is impartial and was present for the consent process.

FOR REVIEW ONLY

Appendix B Approved birth control methods (for sample informed consent form)

Title: A phase 1 clinical trial to evaluate the safety and drug levels of a human monoclonal antibody, VRC-HIVMAB060-00-AB (VRC01) administered in multiple doses intravenously and subcutaneously in different dosing schedules to healthy, HIV-uninfected adults

HVTN protocol number: HVTN 104

Site: [Insert site name]

You should not become pregnant during the study because we do not know how the study products could affect the developing baby.

If you were born female and are sexually active in a way that could lead you to get pregnant, you must agree to use effective birth control from 21 days before your first injection or infusion until after your last required clinic visit.

Effective birth control means using any of the following methods every time you have sex:

- Birth control drugs that prevent pregnancy—given by pills, shots, patches, vaginal rings, or inserts under the skin;
- Male or female condoms, with or without a cream or gel that kills sperm;
- Diaphragm or cervical cap with a cream or gel that kills sperm;
- Intrauterine device (IUD); or
- Any other contraceptive method approved by the researchers.

You do not have to use birth control if:

- You are only having sex with a partner or partners who have had a vasectomy. (We will ask you some questions to confirm that the vasectomy was successful.);
- You have reached menopause, with no menstrual periods for one year;
- You have had a hysterectomy (your uterus removed);
- You have had your ovaries removed;
- You have a tubal ligation (your “tubes tied”) or confirmed successful placement of a product that blocks the fallopian tubes;
- You are having sex only with a female partner or partners;
- You only have oral sex; or,

FOR REVIEW ONLY

- You are sexually abstinent (no sex at all).

Remember: If you are having sex, you need to use male or female condoms to protect yourself from HIV infection.

FOR REVIEW ONLY

Appendix C Sample consent form for use of samples and information in other studies

Title: A phase 1 clinical trial to evaluate the safety and drug levels of a human monoclonal antibody, VRC-HIVMAB060-00-AB (VRC01) administered in multiple doses intravenously and subcutaneously in different dosing schedules to healthy, HIV-uninfected adults<br><br>HVTN protocol number: HVTN 104<br><br>Site: [Insert site name]

When samples are no longer needed for this study, the HVTN wants to keep them for use in other studies. We will call these "extra samples."

This form gives you information so you can decide if you want your extra samples and information used in other studies. You will mark your decision at the end of the form. If you have any questions, please ask.

1. Do I have to agree?

No. You are free to say yes or no, or to change your mind after you sign this form. Your decision will not affect your being in this study or have any negative consequences here.

2. Where are the samples stored?

Extra samples are stored in a secure central place called a repository. *[Site: insert specific information if your regulatory authority requires it.]* The central repositories for the HVTN are located in the United States.

3. How long will the samples be stored?

There is no limit on how long your extra samples will be stored. *[Site: insert limits if your regulatory authority imposes them.]*

4. Will I be paid for the use of my samples?

No. Also, a researcher may make a new scientific discovery or product based on the use of your samples. If this happens, there is no plan to share any money with you. The researcher is not likely to ever know who you are.

5. Will I benefit from allowing my samples to be used in other studies?

Probably not. Results from these other studies are not given to you, this clinic, or your doctor. They are not needed for your medical care. They are not part of your medical record. The studies are only being done for research purposes.

6. Will the HVTN sell my samples and information?

No, but the HVTN may share your samples with other researchers. Once we share your samples, we will not be able to get them back.

FOR REVIEW ONLY

7. How do other researchers get my samples and information?

When a researcher wants to use your samples and/or information, their research plan must be approved by the HVTN. Also, the researcher's institutional review board (IRB) or ethics committee (EC) will review their plan. *[Site: insert review by your institution's IRB/EC, if applicable.]* IRBs/ECs protect the rights and well-being of people in research. The HVTN keeps track of your decision about how your samples and information can be used.

8. What information is shared with other researchers?

The samples and limited information they receive will be labeled with a code number. Your name will not be part of the information. However, some information that we share may be personal, such as your race, ethnicity, gender, health information from the study, and HIV status. We may share information about the study products you received and how your body responded to the study products.

9. What kind of studies might be done with my extra samples and information?

The studies will be related to HIV, vaccines, the immune system and other diseases. The researchers may also:

- Take cells from your samples and grow more of them. This means the researchers may keep your cells growing over time.

- Do limited genetic testing, which involves only looking at some of your genes, not all of your genes.

If you agree, your samples could also be used for genome wide studies. In these studies, researchers will look at all of your genes (your genome). The researchers compare the genomes of many people, looking for common patterns of genes that could help them understand diseases. The researchers may put the information from the genome-wide studies into a protected database so that other researchers can access it. Usually, no one would be able to look at your genome and link it to you as a person. However, if another database exists that also has information on your genome and your name, someone might be able to compare the databases and identify you. If others found out, it could lead to discrimination or other problems. The risk of this is very small.

10. What are the risks of genetic testing?

The genetic testing could show you may be at risk for certain diseases. If others found out, it could lead to discrimination or other problems. However, it is almost impossible for you or others to know your test results from the genetic testing. The results are not part of your study records and are not given to you.

In the very unlikely event that your genetic information becomes linked to your name, a federal law called the Genetic Information Nondiscrimination Act (GINA) helps protect you. GINA keeps health insurance companies and employers from seeing results of genetic testing when deciding about giving you health insurance or offering you work. GINA does not help or protect you against discrimination by companies that sell life, disability or long-term care insurance.

FOR REVIEW ONLY

11. Who will have access to my information in studies using my extra samples?

Some people will be able to see the research records from any new study that uses your extra samples and information. Remember that your name will not be part of the information.

People who may see your information are:

- Researchers who use your stored samples and limited information for other research
- Government agencies that fund or monitor the research using your samples or information
- The researcher's Institutional Review Board or Ethics Committee
- The people who work with the researcher

All reviewers will take steps to keep your records private. The results of any new studies that use your extra samples or information may be published. No publication will use your name or identify you personally.

Questions

12. If you have questions or problems about allowing your samples and information to be used in other studies, use the following important contacts.

If you have questions about the use of your samples or information or if you want to change your mind about their use, contact [name and telephone number of the investigator or other study staff].

If you think you may have been harmed because of studies using your samples or information, contact [name and telephone number of the investigator or other study staff].

If you have questions about your rights as a research participant, contact [name/title/phone of person on IRB or other appropriate organization].

13. Please write your initials or make your mark in the box next to the option you choose.

FOR REVIEW ONLY

☐ I allow my extra samples combined with limited information for other studies related to HIV, vaccines, the immune system, and other diseases. This may include limited genetic testing and keeping my cells growing over time.

OR

☐ I agree to the option above and also to allow my extra samples combined with limited information to be used in genome wide studies.

OR

☐ I do not allow my extra samples to be used in any other studies. This includes not allowing limited genetic testing, growing more of my cells, or genome wide studies.

Participant's name (print)	Participant's signature or mark	Date	Time
Clinic staff conducting consent discussion (print)	Clinic staff signature	Date	Time

For participants who are unable to read or write, a witness should complete the signature block below:

Witness's name (print)	Witness's signature	Date	Time

*Witness is impartial and was present for the consent process.

FOR REVIEW ONLY

Appendix D Table of procedures (for sample informed consent form)

Group 1

Procedure	Screening visit	First infusion visit	Time after first infusion															
			3 days	2 weeks	1 month	1 month + 3 days	1 ½ months	2 months	3 months	4 months	5 months	5 months + 3 days	5 ½ months	6 months	6½ months	7 months	7 ½ months	8 months
IV Infusion		√			√			√	√	√	√							
Medical history	√																	
Complete physical	√																	√
Brief physical		√	√	√	√	√	√	√	√	√	√	√	√	√	√	√	√	
Urine test	√			√					√					√				
Blood drawn	√	√	√	√	√	√	√	√	√	√	√ (2x)[d]	√	√	√	√	√	√	√
Pregnancy test (participants born female)[a]	√	√			√	√[c]	√[c]	√	√	√	√	√[c]	√[c]	√[c]		√[c]		√
HIV testing & pretest counseling	√								√					√				√
Risk reduction counseling	√	√	√	√	√	√	√	√	√	√	√	√	√	√	√	√	√	√
Interview/ questionnaire	√	√	√	√	√	√	√	√	√	√	√	√	√	√	√	√	√	√
Pap smear (if needed)[b]																		
Cervical fluid sample (optional)					√	√	√	√	√		√	√	√	√		√		√
Rectal fluid sample (optional)					√	√	√	√	√		√	√	√	√		√		√
Semen sample (optional)					√	√	√	√	√		√	√	√	√		√		√
Saliva sample (optional)					√	√	√	√	√		√	√	√	√		√		√

Light grayed out columns and cells = these visits are only for those participants who consent to provide optional mucosal samples that were not collected at the corresponding visits after the 2nd infusion.

[a] Persons who have had a total hysterectomy (removal of the uterus and ovaries, verified by medical records), are not required to undergo pregnancy testing.

[b] Only for volunteers born female who consent to provide cervical secretion samples. Participants 21 years of age or older must have had a Pap smear within 3 years prior to giving cervical samples, with the latest result as normal or ASCUS, and the clinic staff will check your medical records to confirm this.

[c] Pregnancy testing will only occur at this visit if the participant consents to cervical and/or rectal sampling.

[d] Blood will be drawn 2 times during this visit. Once before the infusion and again 1 hour after the infusion is finished.

FOR REVIEW ONLY

Groups 2, 4, and 5

Procedure	Screening visit	First infusion visit	Time after first infusion															
			3 days	2 weeks	1 month	2 months	2 months + 3 days	2 ½ months	3 months	4 months	4 months + 3 days	4 ½ months	5 months	6 months	6 ½ months	7 months	7 ½ months	8 months
IV Infusion		√				√				√								
Medical history	√																	
Complete physical	√																	√
Brief physical		√	√	√	√	√	√	√	√	√	√	√	√	√	√	√	√	
Urine test	√			√					√					√				
Blood drawn	√	√	√	√	√	√	√	√	√	√ (2x)[d]	√	√	√	√	√	√	√	√
Pregnancy test (participants born female)[a]	√	√				√	√[c]	√[c]	√	√	√[c]	√[c]	√[c]	√[c]		√[c]		√
HIV testing & pretest counseling	√								√					√				√
Risk reduction counseling	√	√	√	√	√	√	√	√	√	√	√	√	√	√	√	√	√	√
Interview/ questionnaire	√	√	√	√	√	√	√	√	√	√	√	√	√	√	√	√	√	√
Pap smear (if needed)[b]																		
Cervical fluid sample (optional)						√	√	√	√	√	√	√	√	√		√		√
Rectal fluid sample (optional)						√	√	√	√	√	√	√	√	√		√		√
Semen sample (optional)						√	√	√	√	√	√	√	√	√		√		√
Saliva sample (optional)						√	√	√	√	√	√	√	√	√		√		√

Light grayed out columns and cells = these visits are only for those participants who consent to provide optional mucosal samples that were not collected at the corresponding visits after the 2nd infusion.

[a] Persons who have had a total hysterectomy (removal of the uterus and ovaries, verified by medical records), are not required to undergo pregnancy testing

[b] Only for volunteers born female who consent to provide cervical secretion samples. Participants 21 years of age or older must have had a Pap smear within 3 years prior to giving cervical samples, with the latest result as normal or ASCUS, and the clinic staff will check your medical records to confirm this.

[c] Pregnancy testing will only occur at this visit if the participant consents to cervical and/or rectal sampling.

[d] Blood will be drawn 2 times during this visit. Once before the infusion and again 1 hour after the infusion is finished.

FOR REVIEW ONLY

Group 3

Procedure	Screening visit	First infusion visit	Time after first infusion																		
			3 days	2 weeks	2 weeks + 3 days	1 month	1½ months	2 months	2½ months	3 months	3½ months	4 months	4½ months	5 months	5½ months	5½ months + 3 days	6 months	6 ½ months	7 months	7 ½ months	8 months
IV Infusion		√																			
SC injection				√		√	√	√	√	√	√	√	√	√	√						
Medical history	√																				
Complete physical	√																				√
Brief physical		√	√	√	√	√	√	√	√	√	√	√	√	√	√	√	√	√	√	√	
Urine test	√			√						√							√				
Blood drawn	√	√	√	√	√	√	√	√	√	√	√	√	√	√	√	√	√	√	√	√	√
Pregnancy test (participants born female)[a]	√	√		√	√[c]	√	√	√	√	√	√	√	√	√	√	√[c]	√[c]		√[c]		√
HIV testing & pretest counseling	√									√							√				√
Risk reduction counseling	√	√	√	√	√	√	√	√	√	√	√	√	√	√	√	√	√	√	√	√	√
Interview\ questionnaire	√	√	√	√	√	√	√	√	√	√	√	√	√	√	√	√	√	√	√	√	√
Pap smear (if needed)[b]																					
Cervical fluid sample (optional)				√	√	√	√			√					√	√	√		√		√
Rectal fluid sample (optional)				√	√	√	√			√					√	√	√		√		√
Semen sample (optional)				√	√	√	√			√					√	√	√		√		√
Saliva sample (optional)				√	√	√	√			√					√	√	√		√		√

Light grayed out cells = these are only for those participants who consent to provide optional mucosal samples that were not collected at the corresponding visits after the 1st SC injection.

[a] Persons who have had a total hysterectomy (removal of the uterus and ovaries, verified by medical records), are not required to undergo pregnancy testing

[b] Only for volunteers born female who consent to provide cervical secretion samples. Participants 21 years of age or older must have had a Pap smear within 3 years prior to giving cervical samples, with the latest result as normal or ASCUS, and the clinic staff will check your medical records to confirm this.

[c] Pregnancy testing will only occur at this visit if the participant consents to cervical and/or rectal sampling.

Not shown in this table is a time after all study participants have completed their last scheduled visit when you can find out what products you received.

FOR REVIEW ONLY

Appendix E   Laboratory procedures for Group 1

				Visit:	1	2	3	4	5	6	7	8	9	10	11	12	13	14	14.5	15	16	16.5	17	18	19	20	21	22	23	
				Day:	Screening visit[3]	D0	D3	D14	D17	D28	D31	D42	D56	D59	D70	D84	D98	D112	D115	D126	D140	D143	D154	D157	D168	D182	D196	D210	D224	
				Month:		M0		M0.5		M1		M1.5	M2		M2.5	M3	M3.5	M4		M4.5	M5		M5.5		M6	M6.5	M7	M7.5	M8	
						**Inf#1**				**Inf#2**			**Inf#3**			**Inf#4**		**Inf#5**			**Inf#6**									
**Description**	**Ship to[1,2]**	**Assay location[2]**	**Tube[4]**	**Tube size (vol capacity)[4]**																										**Total**
BLOOD COLLECTION																														
Screening or diagnostic assays																														
Screening HIV test	Local lab	Local lab	SST	5mL	5	-	-	-	-	-	-	-	-	-	-	-	-	-	-	-	-	-	-	-	-	-	-	-	-	**5**
HBsAg/anti-HCV/Syphilis	Local lab	Local lab	SST	5mL	5	-	-	-	-	-	-	-	-	-	-	-	-	-	-	-	-	-	-	-	-	-	-	-	-	**5**
HIV diagnostic algorithm[8]	UW-VSL	UW-VSL	EDTA	10mL	-	-	-	-	-	-	-	-	-	-	-	10	-	-	-	-	-	-	-	-	10	-	-	-	20	**40**
Safety labs																														
CBC/ Diff/ platelets	Local lab	Local lab	EDTA	5mL	5	-	-	5	-	5	-	-	5	-	-	5	-	5	-	-	5	-	-	-	5	-	-	-	-	**40**
Chemistry Panel[5]	Local lab	Local lab	SST	5mL	5	-	-	5	-	5	-	-	5	-	-	5	-	5	-	-	5	-	-	-	5	-	-	-	-	**40**
Drug levels																														
VRC01 Ab levels	CSR	NVITAL	SST	8.5mL	-	8.5	8.5	8.5	-	8.5	8.5	8.5	8.5	-	-	8.5	-	8.5	-	-	17[11]	8.5	8.5	-	8.5	8.5	8.5	8.5	8.5	**153**
Immunogenicity assays																														
HLA typing[6]	CSR	FHCRC	ACD	8.5mL	—	17	—	—	-	—	—	-	—	-	-	—	-	—	-	-	—	—	-	-	—	-	-	-	—	17
Humoral Assays																														
VRC01 binding Ab assay	CSR	Duke-DHVI	SST	8.5mL	-	y	-	-	-	y	y	y	y	-	-	y	-	y	-	-	y	y	y	-	y	y	y	y	y	**0**
anti-VRC01 Ab levels	CSR	NVITAL	SST	8.5mL	-	-	-	-	-	8.5	-	-	8.5	-	-	8.5	-	8.5	-	-	8.5	-	-	-	8.5	-	-	-	8.5	**59.5**
Neutralizing Ab assay	CSR	NVITAL/Duke-NAB	SST	8.5ml	-	8.5	-	-	-	8.5	8.5	8.5	8.5	-	-	8.5	-	8.5	-	-	8.5	8.5	8.5	-	8.5	8.5	8.5	8.5	8.5	**127.5**
Storage																														
PBMC	CSR	—	ACD	8.5mL	-	42.5	-	-	-	-	42.5	-	-	-	-	-	-	-	-	-	-	-	-	-	42.5	-	-	-	-	**127.5**
Serum	CSR	—	SST	8.5mL	-	8.5	8.5	8.5	-	8.5	8.5	8.5	8.5	-	-	8.5	-	8.5	-	-	8.5	8.5	8.5	-	8.5	8.5	8.5	8.5	8.5	**144.5**
**Maximum Total**					**20**	**85**	**17**	**27**	-	**44**	**68**	**25.5**	**44**	-	-	**54**	-	**44**	-	-	**52.5**	**25.5**	**25.5**	-	**96.5**	**25.5**	**25.5**	**25.5**	**54**	759
**Maximum 56-Day Total**					**20**	**105**	**122**	**149**	-	**193**	**261**	**286.5**	**330.5**	-	-	**235.5**	-	**142**	-	-	**150.5**	**122**	**147.5**	-	**244**	**225.5**	**251**	**198.5**	**227**	
URINE COLLECTION																														
Urinalysis	Local lab	Local lab			X	-	-	X	-	-	-	-	-	-	-	X	-	-	-	-	-	-	-	-	X	-	-	-	-	
Pregnancy Test[7]	Local lab	Local lab			X	X	-	-	-	X	X[9]	X[9]	X	-	-	X	-	X	-	-	X	X[9]	X[9]	-	X[9]	-	X[9]	-	X[9]	
MUCOSAL SPECIMEN COLLECTION (OPTIONAL)[10]																														
Saliva	CSR	NVITAL/Duke-DHVI			-	-	-	-	-	X	X	X	X	-	-	X	-	-	-	-	X	X	X	-	X	-	X	-	X	
Semen	CSR	NVITAL/Duke-DHVI			-	-	-	-	-	X	X	X	X	-	-	X	-	-	-	-	X	X	X	-	X	-	X	-	X	
Cervical Secretions	CSR	NVITAL/Duke-DHVI			-	-	-	-	-	X	X	X	X	-	-	X	-	-	-	-	X	X	X	-	X	-	X	-	X	
Rectal Secretions	CSR	NVITAL/Duke-DHVI			-	-	-	-	-	X	X	X	X	-	-	X	-	-	-	-	X	X	X	-	X	-	X	-	X	

Dark grayed out columns = visits not required for Group 1

Light grayed out columns and cells = visits 16.5 and 17 will only occur for participants who agree to provide mucosal specimens and if mucosal specimens were not collected at visits 7 and 8, respectively. Mucosal specimen collections at visit 16 indicate collections will occur at that visit if participants consent to provide mucosal specimens and specimens were not collected at visit 6.

Inf = infusion

y = 8.5mL of SST blood collected for VRC01 drug levels will also cover specimen needs for VRC01 humoral assays; no separate blood draw is needed.

[1] CSR = central specimen repository

[2] HVTN Laboratory Program includes laboratories at UW-VSL, Duke-DHVI, Duke-NAB, and FHCRC. UW-VSL = University of Washington Virology Specialty Laboratory (Seattle, Washington, USA); Duke-DHVI = Duke Human Vaccine Institute, Duke University Medical Center (Durham, North Carolina, USA); Duke-NAB = Duke Neutralizing Antibody Laboratory (Durham, North Carolina, USA); FHCRC = Fred Hutchinson Cancer Research Center (Seattle, Washington, USA). Non-HVTN laboratories include NVITAL = NIAID Vaccine Immune T-Cell and Antibody Laboratory (NVITAL) (Gaithersburg, MD, USA).

[3] Screening may occur over the course of several contacts/visits up to and including day 0 prior to infusion.

[4] Local labs may assign appropriate alternative tube types for locally performed tests.

[5] Chemistry panels are defined in section 9.2.

[6] Genotyping may be performed on enrolled participants using cryopreserved PBMC collected at baseline.

[7] Pregnancy test may be performed on blood specimens. Persons who are not of reproductive potential due to having undergone total hysterectomy with bilateral oophorectomy (verified by medical records), are not required to undergo pregnancy testing.

[8] At an early termination visit for a withdrawn or terminated participant (see Section 9.11), blood should be drawn for HIV diagnostic testing, as shown for visit 23 above.

[9] Pregnancy testing will only occur at this visit if the participant consents to cervical and/or rectal sampling.

[10] Optional mucosal specimens may be collected once the participant has been found to have met mucosal specimen collection criteria specified in the SSP.

[11] During visit 16, 8.5 mL of SST blood will be collected before the infusion and 8.5 mL of SST blood will be collected 1 hour after the infusion is complete.

FOR REVIEW

Appendix F Laboratory procedures for Groups 2, 4, and 5

				Visit:	1	2	3	4	5	6	7	8	9	10	11	12	13	14	14.5	15	16	16.5	17	18	19	20	21	22	23	
				Day:	Screening visit[3]	D0	D3	D14	D17	D28	D31	D42	D56	D59	D70	D84	D98	D112	D115	D126	D140	D143	D154	D157	D168	D182	D196	D210	D224	
				Month:		M0		M0.5		M1		M1.5	M2		M2.5	M3	M3.5	M4		M4.5	M5		M5.5		M6	M6.5	M7	M7.5	M8	
						**Inf#1**							**Inf#2**					**Inf#3**												
**Description**	**Ship to[1,2]**	**Assay location[2]**	**Tube[4]**	**Tube size (vol capacity)[4]**																										**Total**
BLOOD COLLECTION																														
Screening or diagnostic assays																														
Screening HIV test	Local lab	Local lab	SST	5mL	5	-	-	-	-	-	-	-	-	-	-	-	-	-	-	-	-	-	-	-	-	-	-	-	-	**5**
HBsAg/anti-HCV/Syphilis	Local lab	Local lab	SST	5mL	5	-	-	-	-	-	-	-	-	-	-	-	-	-	-	-	-	-	-	-	-	-	-	-	-	**5**
HIV diagnostic algorithm[8]	UW-VSL	UW-VSL	EDTA	10mL	-	-	-	-	-	-	-	-	-	-	-	10	-	-	-	-	-	-	-	-	10	-	-	-	20	**40**
Safety labs																														
CBC/ Diff/ platelets	Local lab	Local lab	EDTA	5mL	5	-	-	5	-	-	-	-	5	-	-	5	-	5	-	-	-	-	-	-	5	-	-	-	-	**30**
Chemistry Panel[5]	Local lab	Local lab	SST	5mL	5	-	-	5	-	-	-	-	5	-	-	5	-	5	-	-	-	-	-	-	5	-	-	-	-	**30**
Drug levels																														
VRC01 Ab levels	CSR	NVITAL	SST	8.5mL	-	8.5	8.5	8.5	-	8.5	-	-	8.5	8.5	8.5	8.5	-	17[11]	8.5	8.5	8.5	-	-	-	8.5	8.5	8.5	8.5	8.5	**153**
Immunogenicity assays																														
HLA typing[6]	CSR	FHCRC	ACD	8.5mL	—	17	—	—	-	-	-	-	—	—	-	—	-	—	—	-	-	-	-	-	—	-	-	-	-	**17**
Humoral Assays																														
VRC01 binding Ab assay	CSR	Duke-DHVI	SST	8.5mL	-	y	-	-	-	-	-	-	y	y	y	y	-	y	y	y	y	-	-	-	y	y	y	y	y	**0**
anti-VRC01 Ab levels	CSR	NVITAL	SST	8.5mL	-	-	-	-	-	-	-	-	8.5	-	-	8.5	-	8.5	-	-	-	-	-	-	8.5	-	-	-	8.5	**42.5**
Neutralizing Ab assay	CSR	NVITAL/Duke-NAB	SST	8.5ml	-	8.5	-	-	-	-	-	-	8.5	8.5	8.5	8.5	-	8.5	8.5	8.5	8.5	-	-	-	8.5	8.5	8.5	8.5	8.5	**119**
Storage																														
PBMC	CSR	—	ACD	8.5mL	-	42.5	-	-	-	-	-	-	-	42.5	-	-	-	-	-	-	-	-	-	-	42.5	-	-	-	-	**127.5**
Serum	CSR	—	SST	8.5mL	-	8.5	8.5	8.5	-	8.5	-	-	8.5	8.5	8.5	8.5	-	8.5	8.5	8.5	8.5	-	-	-	8.5	8.5	8.5	8.5	8.5	**144.5**
**Maximum Total**					**20**	**85**	**17**	**27**	-	**17**	-	-	**44**	**68**	**25.5**	**54**	-	**52.5**	**25.5**	**25.5**	**25.5**	-	-	-	**96.5**	**25.5**	**25.5**	**25.5**	**54**	**713.5**
**Maximum 56-Day Total**					**20**	**105**	**122**	**149**	-	**166**	-	-	**210**	**173**	**181.5**	**208.5**	-	**244**	**225.5**	**183**	**183**	-	-	-	**225.5**	**173**	**173**	**173**	**227**	
URINE COLLECTION																														
Urinalysis	Local lab	Local lab			X	-	-	X	-	-	-	-	-	-	-	X	-	-	-	-	-	-	-	-	X	-	-	-	-	
Pregnancy Test[7]	Local lab	Local lab			X	X	-	-	-	-	-	-	X	X[9]	X[9]	X[9]	-	X	X[9]	X[9]	X[9]	-	-	-	X[9]	-	X[9]	-	X[9]	
MUCOSAL SPECIMEN COLLECTION (OPTIONAL)[10]																														
Saliva	CSR	NVITAL/Duke-DHVI			-	-	-	-	-	-	-	-	X	X	X	X	-	X	X	X	X	-	-	-	X	-	X	-	X	
Semen	CSR	NVITAL/Duke-DHVI			-	-	-	-	-	-	-	-	X	X	X	X	-	X	X	X	X	-	-	-	X	-	X	-	X	
Cervical Secretions	CSR	NVITAL/Duke-DHVI			-	-	-	-	-	-	-	-	X	X	X	X	-	X	X	X	X	-	-	-	X	-	X	-	X	
Rectal Secretions	CSR	NVITAL/Duke-DHVI			-	-	-	-	-	-	-	-	X	X	X	X	-	X	X	X	X	-	-	-	X	-	X	-	X	

Dark grayed out columns = visits not required for Groups 2, 4, and 5

Light grayed out columns = visits 14.5, 15, and 16 will only occur for participants who agree to provide mucosal specimens and if mucosal specimens were not collected at visits 10 and 11 and 12, respectively. Mucosal specimen collections at visit 14 indicate collections will occur at that visit if participants consent to provide mucosal specimens and specimens were not collected at visit 9.

Inf = infusion

y = 8.5mL of SST blood collected for VRC01 drug levels will also cover specimen needs for VRC01 humoral assays; no separate blood draw is needed.

[1] CSR = central specimen repository

[2] HVTN Laboratory Program includes laboratories at UW-VSL, Duke-DHVI, Duke-NAB, and FHCRC. UW-VSL = University of Washington Virology Specialty Laboratory (Seattle, Washington, USA); Duke-DHVI = Duke Human Vaccine Institute, Duke University Medical Center (Durham, North Carolina, USA); Duke-NAB = Duke Neutralizing Antibody Laboratory (Durham, North Carolina, USA); FHCRC = Fred Hutchinson Cancer Research Center (Seattle, Washington, USA). Non-HVTN laboratories include NVITAL = NIAID Vaccine Immune T-Cell and Antibody Laboratory (NVITAL) (Gaithersburg, MD, USA).

[3] Screening may occur over the course of several contacts/visits up to and including day 0 prior to infusion.

[4] Local labs may assign appropriate alternative tube types for locally performed tests.

[5] Chemistry panels are defined in section 9.2.

[6] Genotyping may be performed on enrolled participants using cryopreserved PBMC collected at baseline.

[7] Pregnancy test may be performed on blood specimens. Persons who are not of reproductive potential due to having undergone total hysterectomy with bilateral oophorectomy (verified by medical records), are not required to undergo pregnancy testing.

[8] At an early termination visit for a withdrawn or terminated participant (see Section 9.11), blood should be drawn for HIV diagnostic testing, as shown for visit 23 above.

[9] Pregnancy testing will only occur at this visit if the participant consents to cervical and/or rectal sampling.

[10] Optional mucosal specimens may be collected once the participant has been found to have met mucosal specimen collection criteria specified in the SSP.

[11] During visit 14, 8.5 mL of SST blood will be collected before the infusion and 8.5 mL of SST blood will be collected 1 hour after the infusion is complete.

FOR REVIEW

Appendix G Laboratory procedures for Group 3

				Visit:	1	2	3	4	5	6	7	8	9	10	11	12	13	14	14.5	15	16	16.5	17	18	19	20	21	22	23	
				Day:	Screening visit[3]	D0	D3	D14	D17	D28	D31	D42	D56	D59	D70	D84	D98	D112	D115	D126	D140	D143	D154	D157	D168	D182	D196	D210	D224	
				Month:		M0		M0.5		M1		M1.5	M2		M2.5	M3	M3.5	M4		M4.5	M5		M5.5		M6	M6.5	M7	M7.5	M8	
						Inf#1		Inj#2		Inj#3		Inj#4	Inj#5		Inj#6	Inj#7	Inj#8	Inj#9		Inj#10	Inj#11		Inj#12							
**Description**	**Ship to[1,2]**	**Assay location[2]**	**Tube[4]**	**Tube size (vol capacity)[4]**																										**Total**
BLOOD COLLECTION																														
Screening or diagnostic assays																														
Screening HIV test	Local lab	Local lab	SST	5mL	5	-	-	-	-	-	-	-	-	-	-	-	-	-	-	-	-	-	-	-	-	-	-	-	-	**5**
HBsAg/anti-HCV/Syphilis	Local lab	Local lab	SST	5mL	5	-	-	-	-	-	-	-	-	-	-	-	-	-	-	-	-	-	-	-	-	-	-	-	-	**5**
HIV diagnostic algorithm[8]	UW-VSL	UW-VSL	EDTA	10mL	-	-	-	-	-	-	-	-	-	-	-	10	-	-	-	-	-	-	-	-	10	-	-	-	20	**40**
Safety labs																														
CBC/ Diff/ platelets	Local lab	Local lab	EDTA	5mL	5	-	-	5	-	5	-	-	5	-	-	5	-	5	-	-	5	-	-	-	5	-	-	-	-	**40**
Chemistry Panel[5]	Local lab	Local lab	SST	5mL	5	-	-	5	-	5	-	-	5	-	-	5	-	5	-	-	5	-	-	-	5	-	-	-	-	**40**
Drug levels																														
VRC01 Ab levels	CSR	NVITAL	SST	8.5mL	-	8.5	8.5	8.5	8.5	8.5	-	8.5	8.5	-	8.5	8.5	8.5	8.5	-	8.5	8.5	-	8.5	8.5	8.5	8.5	8.5	8.5	8.5	**170**
Immunogenicity assays																														
HLA typing[6]	CSR	FHCRC	ACD	8.5mL	—	17	—	—	—	—	-	—	—	-	—	—	—	—	-	—	—	-	—	—	—	-	-	-	—	**17**
Humoral Assays																														
VRC01 binding Ab assay	CSR	Duke-DHVI	SST	8.5mL	-	y	-	y	y	y	-	y	y	-	y	y	y	y	-	y	y	-	y	y	y	y	y	y	y	**0**
anti-VRC01 Ab levels	CSR	NVITAL	SST	8.5mL	-	-	-	8.5	-	8.5	-	8.5	8.5	-	8.5	8.5	8.5	8.5	-	8.5	8.5	-	8.5	-	8.5	-	-	-	8.5	**110.5**
Neutralizing Ab assay	CSR	NVITAL/Duke-NAB	SST	8.5ml	-	8.5	-	8.5	8.5	8.5	-	8.5	8.5	-	8.5	8.5	8.5	8.5	-	8.5	8.5	-	8.5	8.5	8.5	8.5	8.5	8.5	8.5	**161.5**
Storage																														
PBMC	CSR	—	ACD	8.5mL	-	42.5	-	-	42.5	-	-	-	-	-	-	-	-	-	-	-	-	-	-	42.5	42.5	-	-	-	-	**170**
Serum	CSR	—	SST	8.5mL	-	8.5	8.5	8.5	8.5	8.5	-	8.5	8.5	-	8.5	8.5	8.5	8.5	-	8.5	8.5	-	8.5	8.5	8.5	8.5	8.5	8.5	8.5	**170**
**Maximum Total**					**20**	**85**	**17**	**44**	**68**	**44**	-	**34**	**44**	-	**34**	**54**	**34**	**44**	-	**34**	**44**	-	**34**	**68**	**96.5**	**25.5**	**25.5**	**25.5**	**54**	**929**
**Maximum 56-Day Total**					**20**	**105**	**122**	**166**	**234**	**278**	-	**312**	**356**	-	**268**	**210**	**200**	**210**	-	**200**	**210**	-	**190**	**224**	**320.5**	**302**	**293.5**	**275**	**227**	
URINE COLLECTION																														
Urinalysis	Local lab	Local lab			X	-	-	X	-	-	-	-	-	-	-	X	-	-	-	-	-	-	-	-	X	-	-	-	-	
Pregnancy Test[7]	Local lab	Local lab			X	X	-	X	X[9]	X	-	X	X	-	X	X	X	X	-	X	X	-	X	X[9]	X[9]	-	X[9]	-	X[9]	
MUCOSAL SPECIMEN COLLECTION (OPTIONAL)[10]																														
Saliva	CSR	NVITAL/Duke-DHVI			-	-	-	X	X	X	-	X	-	-	-	X	-	-	-	-	-	-	X	X	X	-	X	-	X	
Semen	CSR	NVITAL/Duke-DHVI			-	-	-	X	X	X	-	X	-	-	-	X	-	-	-	-	-	-	X	X	X	-	X	-	X	
Cervical Secretions	CSR	NVITAL/Duke-DHVI			-	-	-	X	X	X	-	X	-	-	-	X	-	-	-	-	-	-	X	X	X	-	X	-	X	
Rectal Secretions	CSR	NVITAL/Duke-DHVI			-	-	-	X	X	X	-	X	-	-	-	X	-	-	-	-	-	-	X	X	X	-	X	-	X	

Dark grayed out columns = visits not required for Group 3.

Light grayed out cells = pregnancy test at visit 18 and optional mucosal specimen collections at visits 17 and 18 indicate test and specimen collections may occur at those visits if participants consent to provide mucosal specimens and specimens were not collected at visits 4 and 5, respectively.

Inf = Infusion

Inj = Injection

y = 8.5mL of SST blood collected for VRC01 drug levels will also cover specimen needs for VRC01 humoral assays; no separate blood draw is needed.

[1] CSR = central specimen repository

[2] HVTN Laboratory Program includes laboratories at UW-VSL, Duke-DHVI, Duke-NAB, and FHCRC. UW-VSL = University of Washington Virology Specialty Laboratory (Seattle, Washington, USA); Duke-DHVI = Duke Human Vaccine Institute, Duke University Medical Center (Durham, North Carolina, USA); Duke-NAB = Duke Neutralizing Antibody Laboratory (Durham, North Carolina, USA); FHCRC = Fred Hutchinson Cancer Research Center (Seattle, Washington, USA). Non-HVTN laboratories include NVITAL = NIAID Vaccine Immune T-Cell and Antibody Laboratory (NVITAL) (Gaithersburg, MD, USA).

[3] Screening may occur over the course of several contacts/visits up to and including day 0 prior to infusion.

[4] Local labs may assign appropriate alternative tube types for locally performed tests.

[5] Chemistry panels are defined in section 9.2.

[6] Genotyping may be performed on enrolled participants using cryopreserved PBMC collected at baseline.

[7] Pregnancy test may be performed on blood specimens. Persons who are not of reproductive potential due to having undergone total hysterectomy with bilateral oophorectomy (verified by medical records), are not required to undergo pregnancy testing.

[8] At an early termination visit for a withdrawn or terminated participant (see Section 9.11), blood should be drawn for HIV diagnostic testing, as shown for visit 23 above.

[9] Pregnancy testing will only occur at this visit if the participant consents to cervical and/or rectal sampling.

[10] Optional mucosal specimens may be collected once the participant has been found to have met mucosal specimen collection criteria specified in the SSP.

FOR REVIEW ONLY

Appendix H Procedures at HVTN CRS for Group 1

Visit:	01[a]	02	03	04	05	06	07	08	09	10	11	12	13	14	14.5	15	16	16.5	17	18	19	20	21	22	23	Post
Day:		D0	D3	D14	D17	D28	D31	D42	D56	D59	D70	D84	D98	D112	D115	D126	D140	D143	D154	D157	D168	D182	D196	D210	D224	
Month:		M0		M0.5		M1		M1.5	M2		M2.5	M3	M3.5	M4		M4.5	M5		M5.5		M6	M6.5	M7	M7.5	M8	
Procedure	Scr.	Inf#1				Inf#2			Inf#3			Inf#4		Inf#5			Inf#6									
**Study procedures[b]**																										
Signed screening consent (if used)	X	—	—	—	—	—	—	—	—	—	—	—	—	—	—	—	—	—	—	—	—	—	—	—	—	—
Assessment of understanding	X	—	—	—	—	—	—	—	—	—	—	—	—	—	—	—	—	—	—	—	—	—	—	—	—	—
Signed protocol consent	X	—	—	—	—	—	—	—	—	—	—	—	—	—	—	—	—	—	—	—	—	—	—	—	—	—
Medical history	X	—	—	—	—	—	—	—	—	—	—	—	—	—	—	—	—	—	—	—	—	—	—	—	—	—
Complete physical exam	X	—	—	—	—	—	—	—	—	—	—	—	—	—	—	—	—	—	—	—	—	—	—	—	X	—
Abbreviated physical exam	—	X	X	X	—	X	X	X	X	—	—	X	—	X	—	—	X	X	X	—	X	X	X	X	—	—
Risk reduction counseling	X	X	X	X	—	X	X	X	X	—	—	X	—	X	—	—	X	X	X	—	X	X	X	X	X	—
Pregnancy prevention assessmentc	X	X	X	X	—	X	X	X	X	—	—	X	—	X	—	—	X	X	X	—	X	X	X	X	X	—
Acceptability questionnaire	—	X	—	—	—	X	—	—	X	—	—	X	—	X	—	—	X	—	—	—	—	—	—	—	—	—
Behavioral risk assessment	X	—	—	—	—	—	—	—	—	—	—	X	—	—	—	—	—	—	—	—	X	—	—	—	—	—
Confirm eligibility, obtain demographics, randomize	X	—	—	—	—	—	—	—	—	—	—	—	—	—	—	—	—	—	—	—	—	—	—	—	—	—
Social impact assessment	—	X	X	X	—	X	X	X	X	—	—	X	—	X	—	—	X	X	X	—	X	X	X	X	X	—
Social impact assessment questionnaire	—	—	—	—	—	—	—	—	—	—	—	X	—	—	—	—	—	—	—	—	—	—	—	—	X	—
Concomitant medications	X	X	X	X	—	X	X	X	X	—	—	X	—	X	—	—	X	X	X	—	X	X	X	X	X	—
Intercurrent illness/adverse experience	—	X	X	X	—	X	X	X	X	—	—	X	—	X	—	—	X	X	X	—	X	X	X	X	X	—
HIV infection assessmentd	—	—	—	—	—	—	—	—	—	—	—	X	—	—	—	—	—	—	—	—	X	—	—	—	X	—
Confirm HIV test results provided to participant	—	X	—	—	—	—	—	—	—	—	—	—	—	X	—	—	—	—	—	—	—	X	—	—	—	X

Dark grayed out columns = visits not required for Group 1

Light grayed out columns = these visits are for collection of paired serum and mucosal secretion samples only in those participants who consent to provide optional mucosal samples. To be performed only if mucosal samples were not collected at the corresponding post-infusion #2 visits. Visit 16.5 corresponds to Visit 7. Visit 17 corresponds to Visit 8.

[a] Screening may occur over the course of several contacts/visits up to and including day 0 prior to infusion.

[b] For specimen collection requirements, see Appendix E.

[c] Pregnancy prevention compliance occurs only with participants who were born female and are capable of becoming pregnant.

[d] Includes pretest counseling. A subsequent follow-up contact is conducted to provide post-test counseling and to report results to participant.

FOR REVIEW ONLY

**Local lab assessment**																										
Screening HIV test	X	—	—	—	—	—	—	—	—	—	—	—	—	—	—	—	—	—	—	—	—	—	—	—	—	—
Urine dipstick	X	—	—	X	—	—	—	—	—	—	—	X	—	—	—	—	—	—	—	—	X	—	—	—	—	—
Pregnancy (urine or serum HCG)[e]	X	X	—	—	—	X	X[f]	X[f]	X	—	—	X	—	X	—	—	X	X[f]	X[f]	—	X[f]	—	X[f]	—	X	—
CBC, differential, platelet	X	—	—	X	—	X	—	—	X	—	—	X	—	X	—	—	X	—	—	—	X	—	—	—	—	—
Chemistry panel	X	—	—	X	—	X	—	—	X	—	—	X	—	X	—	—	X	—	—	—	X	—	—	—	—	—
Syphilis, Hepatitis B, Hepatitis C	X	—	—	—	—	—	—	—	—	—	—	—	—	—	—	—	—	—	—	—	—	—	—	—	—	—
Pap smear[g]	—	—	—	—	—	—	—	—	—	—	—	—	—	—	—	—	—	—	—	—	—	—	—	—	—	—
**Mucosal secretion collection (optional)**																										
Cervical, rectal, semen, saliva	—	—	—	—	—	X[h]	X[h]	X[h]	X[h]	—	—	X[h]	—	—	—	—	X[h]	X[h]	X[h]	—	X[h]	—	X[h]	—	X[h]	—
**Study product administration procedures**																										
IV infusion[i]	—	X	—	—	—	X	—	—	X	—	—	X	—	X	—	—	X	—	—	—	—	—	—	—	—	—
Reactogenicity assessments[j]	—	X	X	—	—	X	X	—	X	—	—	X	—	X	—	—	X	—	—	—	—	—	—	—	—	—

[e] For a participant who was born female, pregnancy test must be performed on the day of infusion prior to infusion. Pregnancy test to determine eligibility may be performed at screening or on day 0 prior to first infusion. Serum pregnancy tests may be used to confirm the results of, or substitute for, a urine pregnancy test. Persons who are not of reproductive potential due to having undergone total hysterectomy with bilateral oophorectomy (verified by medical records), are not required to undergo pregnancy testing.

[f] Pregnancy testing will only occur at this visit if the participant consents to cervical and/or rectal sampling.

[g] Only for volunteers born female who consent to provide cervical secretion samples. Per Section 9.5, women with a documented most recent normal (or ASCUS) Pap result within 3 years prior to sample collection, and women under the age of 21 years, do not need to have a Pap smear. If collection of a pap smear is required, this may be done at any time provided the results are available prior to the collection of the cervical secretion samples.

[h] Collection will be done only if the participant consents to provide optional mucosal samples. When collected on the day of an infusion visit, collection must be performed prior to infusion.

[i] Blood draws required at infusion visits must be performed prior to administration of study product; however, it is not necessary to have results prior to administration. Lab tests may be drawn within the 3 days prior to infusion.

[j] Reactogenicity assessments performed daily for at least 3 days postinfusion (see Section 9.9).

FOR REVIEW ONLY

Appendix I Procedures at HVTN CRS for Groups 2, 4, and 5

Visit:	01[a]	02	03	04	05	06	07	08	09	10	11	12	13	14	14.5	15	16	16.5	17	18	19	20	21	22	23	Post
Day:		D0	D3	D14	D17	D28	D31	D42	D56	D59	D70	D84	D98	D112	D115	D126	D140	D143	D154	D157	D168	D182	D196	D210	D224	
Month:		M0		M0.5		M1		M1.5	M2		M2.5	M3	M3.5	M4		M4.5	M5		M5.5		M6	M6.5	M7	M7.5	M8	
Procedure	Scr.	Inf#1							Inf#2					Inf#3												
**Study procedures[b]**																										
Signed screening consent (if used)	X	—	—	—	—	—	—	—	—	—	—	—	—	—	—	—	—	—	—	—	—	—	—	—	—	—
Assessment of understanding	X	—	—	—	—	—	—	—	—	—	—	—	—	—	—	—	—	—	—	—	—	—	—	—	—	—
Signed protocol consent	X	—	—	—	—	—	—	—	—	—	—	—	—	—	—	—	—	—	—	—	—	—	—	—	—	—
Medical history	X	—	—	—	—	—	—	—	—	—	—	—	—	—	—	—	—	—	—	—	—	—	—	—	—	—
Complete physical exam	X	—	—	—	—	—	—	—	—	—	—	—	—	—	—	—	—	—	—	—	—	—	—	—	X	—
Abbreviated physical exam	—	X	X	X	—	X	—	—	X	X	X	X	—	X	X	X	X	—	—	—	X	X	X	X	—	—
Risk reduction counseling	X	X	X	X	—	X	—	—	X	X	X	X	—	X	X	X	X	—	—	—	X	X	X	X	X	—
Pregnancy prevention assessment[c]	X	X	X	X	—	X	—	—	X	X	X	X	—	X	X	X	X	—	—	—	X	X	X	X	X	—
Acceptability questionnaire	—	X	—	—	—	—	—	—	X	—	—	—	—	X	X	X	X	—	—	—	—	—	—	—	—	—
Behavioral risk assessment	X	—	—	—	—	—	—	—	—	—	—	X	—	—	—	—	—	—	—	—	X	—	—	—	—	—
Confirm eligibility, obtain demographics, randomize	X	—	—	—	—	—	—	—	—	—	—	—	—	—	—	—	—	—	—	—	—	—	—	—	—	—
Social impact assessment	—	X	X	X	—	X	—	—	X	X	X	X	—	X	X	X	X	—	—	—	X	X	X	X	X	—
Social impact assessment questionnaire	—	—	—	—	—	—	—	—	—	—	—	X	—	—	—	—	—	—	—	—	—	—	—	—	X	—
Concomitant medications	X	X	X	X	—	X	—	—	X	X	X	X	—	X	X	X	X	—	—	—	X	X	X	X	X	—
Intercurrent illness/adverse experience	—	X	X	X	—	X	—	—	X	X	X	X	—	X	X	X	X	—	—	—	X	X	X	X	X	—
HIV infection assessment[d]	—	—	—	—	—	—	—	—	—	—	—	X	—	—	—	—	—	—	—	—	X	—	—	—	X	—
Confirm HIV test results provided to participant	—	X	—	—	—	—	—	—	—	—	—	—	—	X	—	—	—	—	—	—	—	X	—	—	—	X

Dark grayed out columns = visits not required for Group 2

Light grayed out columns = these visits are for collection of paired serum and mucosal secretion samples only in those participants who consent to provide optional mucosal samples. To be performed only if mucosal samples were not collected at the corresponding post-infusion #2 visits. Visit 14.5 corresponds to Visit 10. Visit 15 corresponds to Visit 11. Visit 16 corresponds to Visit 12.

[a] Screening may occur over the course of several contacts/visits up to and including day 0 prior to infusion.

[b] For specimen collection requirements, see Appendix F.

[c] Pregnancy prevention compliance occurs only with participants who were born female and are capable of becoming pregnant

[d] Includes pretest counseling. A subsequent follow-up contact is conducted to provide post-test counseling and to report results to participant.

FOR REVIEW

**Local lab assessment**																										
Screening HIV test	X	—	—	—	—	—	—	—	—	—	—	—	—	—	—	—	—	—	—	—	—	—	—	—	—	—
Urine dipstick	X	—	—	X	—	—	—	—	—	—	—	X	—	—	—	—	—	—	—	—	X	—	—	—	—	—
Pregnancy (urine or serum HCG)[e]	X	X	—	—	—	—	—	—	X	X[f]	X[f]	X	—	X	X[f]	X[f]	X[f]	—	—	—	X[f]	—	X[f]	—	X	—
CBC, differential, platelet	X	—	—	X	—	—	—	—	X	—	—	X	—	X	—	—	—	—	—	—	X	—	—	—	—	—
Chemistry panel	X	—	—	X	—	—	—	—	X	—	—	X	—	X	—	—	—	—	—	—	X	—	—	—	—	—
Syphilis, Hepatitis B, Hepatitis C	X	—	—	—	—	—	—	—	—	—	—	—	—	—	—	—	—	—	—	—	—	—	—	—	—	—
Pap smear[g]	—	—	—	—	—	—	—	—	—	—	—	—	—	—	—	—	—	—	—	—	—	—	—	—	—	—
**Mucosal secretion collection (optional)**																										
Cervical, rectal, semen, saliva	—	—	—	—	—	—	—	—	X[h]	X[h]	X[h]	X[h]	—	X[h]	X[h]	X[h]	X[h]	—	—	—	X[h]	—	X[h]	—	X[h]	—
**Study product administration procedures**																										
IV infusion[i]	—	X	—	—	—	—	—	—	X	—	—	—	—	X	—	—	—	—	—	—	—	—	—	—	—	—
Reactogenicity assessments[j]	—	X	X	—	—	—	—	—	X	X	—	—	—	X	—	—	—	—	—	—	—	—	—	—	—	—

[e] For a participant who was born female, pregnancy test must be performed on the day of infusion prior to infusion. Pregnancy test to determine eligibility may be performed at screening or on day 0 prior to first infusion. Serum pregnancy tests may be used to confirm the results of, or substitute for, a urine pregnancy test. Persons who are not of reproductive potential due to having undergone total hysterectomy with bilateral oophorectomy (verified by medical records), are not required to undergo pregnancy testing.

[f] Pregnancy testing will only occur at this visit if the participant consents to cervical and/or rectal sampling.

[g] Only for volunteers born female who consent to provide cervical secretion samples. Per Section 9.5, women with a documented most recent normal (or ASCUS) Pap result within 3 years prior to sample collection, and women under the age of 21 years, do not need to have a Pap smear. If collection of a pap smear is required, this may be done at any time provided the results are available prior to the collection of the cervical secretion samples.

[h] Collection will be done only if the participant consents to provide optional mucosal samples. When collected on the day of an infusion visit, collection must be performed prior to infusion.

[i] Blood draws required at infusion visits must be performed prior to administration of study product; however, it is not necessary to have results prior to administration. Lab tests may be drawn within the 3 days prior to infusion.

[j] Reactogenicity assessments performed daily for at least 3 days postinfusion (see Section 9.9).

FOR REVIEW

Appendix J Procedures at HVTN CRS for Group 3

Visit:	01[a]	02	03	04	05	06	07	08	09	10	11	12	13	14	14.5	15	16	16.5	17	18	19	20	21	22	23	Post
Day:		D0	D3	D14	D17	D28	D31	D42	D56	D59	D70	D84	D98	D112	D115	D126	D140	D143	D154	D157	D168	D182	D196	D210	D224	
Month:		M0		M0.5		M1		M1.5	M2		M2.5	M3	M3.5	M4		M4.5	M5		M5.5		M6	M6.5	M7	M7.5	M8	
Procedure	Scr.	Inf#1		Inj#2		Inj#3		Inj#4	Inj#5		Inj#6	Inj#7	Inj#8	Inj#9		Inj#10	Inj#11		Inj#12							
**Study procedures[b]**																										
Signed screening consent (if used)	X	—	—	—	—	—	—	—	—	—	—	—	—	—	—	—	—	—	—	—	—	—	—	—	—	—
Assessment of understanding	X	—	—	—	—	—	—	—	—	—	—	—	—	—	—	—	—	—	—	—	—	—	—	—	—	—
Signed protocol consent	X	—	—	—	—	—	—	—	—	—	—	—	—	—	—	—	—	—	—	—	—	—	—	—	—	—
Medical history	X	—	—	—	—	—	—	—	—	—	—	—	—	—	—	—	—	—	—	—	—	—	—	—	—	—
Complete physical exam	X	—	—	—	—	—	—	—	—	—	—	—	—	—	—	—	—	—	—	—	—	—	—	—	X	—
Abbreviated physical exam	—	X	X	X	X	X	—	X	X	—	X	X	X	X	—	X	X	—	X	X	X	X	X	X	—	—
Risk reduction counseling	X	X	X	X	X	X	—	X	X	—	X	X	X	X	—	X	X	—	X	X	X	X	X	X	X	—
Pregnancy prevention assessment[c]	X	X	X	X	X	X	—	X	X	—	X	X	X	X	—	X	X	—	X	X	X	X	X	X	X	—
Acceptability questionnaire	—	X	—	X	—	X	—	X	X	—	X	X	X	X	—	X	X	—	X	—	—	—	—	—	—	—
Behavioral risk assessment	X	—	—	—	—	—	—	—	—	—	—	X	—	—	—	—	—	—	—	—	X	—	—	—	—	—
Confirm eligibility, obtain demographics, randomize	X	—	—	—	—	—	—	—	—	—	—	—	—	—	—	—	—	—	—	—	—	—	—	—	—	—
Social impact assessment	—	X	X	X	X	X	—	X	X	—	X	X	X	X	—	X	X	—	X	X	X	X	X	X	X	—
Social impact assessment questionnaire	—	—	—	—	—	—	—	—	—	—	—	X	—	—	—	—	—	—	—	—	—	—	—	—	X	—
Belief questionnaire	—	—	—	—	—	—	—	—	—	—	—	X	—	—	—	—	—	—	—	—	—	—	—	—	X	—
Concomitant medications	X	X	X	X	X	X	—	X	X	—	X	X	X	X	—	X	X	—	X	X	X	X	X	X	X	—
Intercurrent illness/adverse experience	—	X	X	X	X	X	—	X	X	—	X	X	X	X	—	X	X	—	X	X	X	X	X	X	X	—
HIV infection assessment[d]	—	—	—	—	—	—	—	—	—	—	—	X	—	—	—	—	—	—	—	—	X	—	—	—	X	—
Confirm HIV test results provided to participant	—	X	—	—	—	—	—	—	—	—	—	—	X	—	—	—	—	—	—	—	—	X	—	—	—	X

Dark grayed out columns = visits not required for Group 3

[a] Screening may occur over the course of several contacts/visits up to and including day 0 prior to infusion.

[b] For specimen collection requirements, see Appendix G.

[c] Pregnancy prevention compliance occurs only with participants who were born female and are capable of becoming pregnant.

[d] Includes pretest counseling. A subsequent follow-up contact is conducted to provide post-test counseling and to report results to participant.

FOR REVIEW ONLY

**Local lab assessment**																										
Screening HIV test	X	—	—	—	—	—	—	—	—	—	—	—	—	—	—	—	—	—	—	—	—	—	—	—	—	—
Urine dipstick	X	—	—	X	—	—	—	—	—	—	—	X	—	—	—	—	—	—	—	—	X	—	—	—	—	—
Pregnancy (urine or serum HCG)[e]	X	X	—	X	X[f]	X	—	X	X	—	X	X	X	X	—	X	X	—	X	X[f]	X[f]	—	X[f]	—	X	—
CBC, differential, platelet	X	—	—	X	—	X	—	—	X	—	—	X	—	X	—	—	X	—	—	—	X	—	—	—	—	—
Chemistry panel	X	—	—	X	—	X	—	—	X	—	—	X	—	X	—	—	X	—	—	—	X	—	—	—	—	—
Syphilis, Hepatitis B, Hepatitis C	X	—	—	—	—	—	—	—	—	—	—	—	—	—	—	—	—	—	—	—	—	—	—	—	—	—
Pap smear[g]	—	—	—	—	—	—	—	—	—	—	—	—	—	—	—	—	—	—	—	—	—	—	—	—	—	—
**Mucosal secretion collection (optional)**																										
Cervical, rectal, semen, saliva	—	—	—	X[h]	X[h]	X[h]	—	X[h]	—	—	—	X[h]	—	—	—	—	—	—	X[i]	X[i]	X[h]	—	X[h]	—	X[h]	—
**Study product administration procedures**																										
IV infusion[j]	—	X	—	—	—	—	—	—	—	—	—	—	—	—	—	—	—	—	—	—	—	—	—	—	—	—
SC injection[j]	—	—	—	X	—	X	—	X	X	—	X	X	X	X	—	X	X	—	X	—	—	—	—	—	—	—
Reactogenicity assessments[k]	—	X	X	X	X	X	—	X	X	—	X	X	X	X	—	X	X	—	X	X	—	—	—	—	—	—
**Poststudy**																										
Unblind participant	—	—	—	—	—	—	—	—	—	—	—	—	—	—	—	—	—	—	—	—	—	—	—	—	—	X

[e] For a participant who was born female, pregnancy test must be performed on the day of infusion/injection prior to infusion/injection. Pregnancy test to determine eligibility may be performed at screening or on day 0 prior to first infusion. Serum pregnancy tests may be used to confirm the results of, or substitute for, a urine pregnancy test. Persons who are not of reproductive potential due to having undergone total hysterectomy with bilateral oophorectomy (verified by medical records), are not required to undergo pregnancy testing.

[f] Pregnancy testing will only occur at this visit if the participant consents to cervical and/or rectal sampling.

[g] Only for volunteers born female who consent to provide cervical secretion samples. Per Section 9.5, women with a documented most recent normal (or ASCUS) Pap result within 3 years prior to sample collection, and women under the age of 21 years, do not need to have a Pap smear. If collection of a pap smear is required, this may be done at any time provided the results are available prior to the collection of the cervical secretion samples.

[h] Collection will be done only if the participant consents to provide optional mucosal samples. When collected on the day of an injection visit, collection must be performed prior to injection.

[i] Collection at these timepoints will be done only if mucosal samples were not collected at the corresponding post-injection #2 visits. Visit 17 corresponds to Visit 4. Visit 18 corresponds to Visit 5. Collection will be done only if the participant consents to provide optional mucosal samples. When collected on the day of an injection visit, collection must be performed prior to injection.

[j] Blood draws required at infusion/injection visits must be performed prior to administration of study product; however, it is not necessary to have results prior to administration. Lab tests may be drawn within the 3 days prior to infusion.

[k] Reactogenicity assessments performed daily for at least 3 days postinfusion/postinjection (see Section 9.9).

FOR REVIEW ONLY

Appendix K Added Criteria to the DAIDS AE Grading Table - Immune System Disorders

ADDED CRITERIA – IMMUNE SYSTEM DISORDERS				
**PARAMETER**	**GRADE 1<br>MILD**	**GRADE 2<br>MODERATE**	**GRADE 3<br>SEVERE**	**GRADE 4<br>POTENTIALLY<br>LIFE-THREATENING**
Cytokine release syndrome	Mild reaction; infusion interruption not indicated; intervention not indicated	Therapy or infusion interruption indicated but responds promptly to symptomatic treatment; prophylactic medications indicated for 24 hours or less	Prolonged (i.e., not rapidly responsive to symptomatic medication or infusion interruption); recurrence of symptoms following initial improvement	Hospitalization needed for clinical sequelae. Life-threatening consequences; pressor or ventilatory support indicated.
Serum sickness	Mild reaction; intervention not indicated	Moderate signs and symptoms (e.g., arthralgia, fever, rash, urticaria); antihistamines indicated	Severe arthralgia or arthritis; extensive rash; steroids or IV fluids indicated	Hospitalization needed for clinical sequelae. Life-threatening consequences; pressor or ventilatory support indicated.

FOR REVIEW ONLY
