# Supplementary figures and images for: Safety, pharmacokinetics, and immunological activities of multiple intravenous or subcutaneous doses of an anti-HIV monoclonal antibody, VRC01, administered to HIV-uninfected adults: Results of a phase 1 randomized trial
Source: PLoS Med. 2017 Nov 14;14(11):e1002435. doi: 10.1371/journal.pmed.1002435 (PMC5685476; doi:10.1371/journal.pmed.1002435)

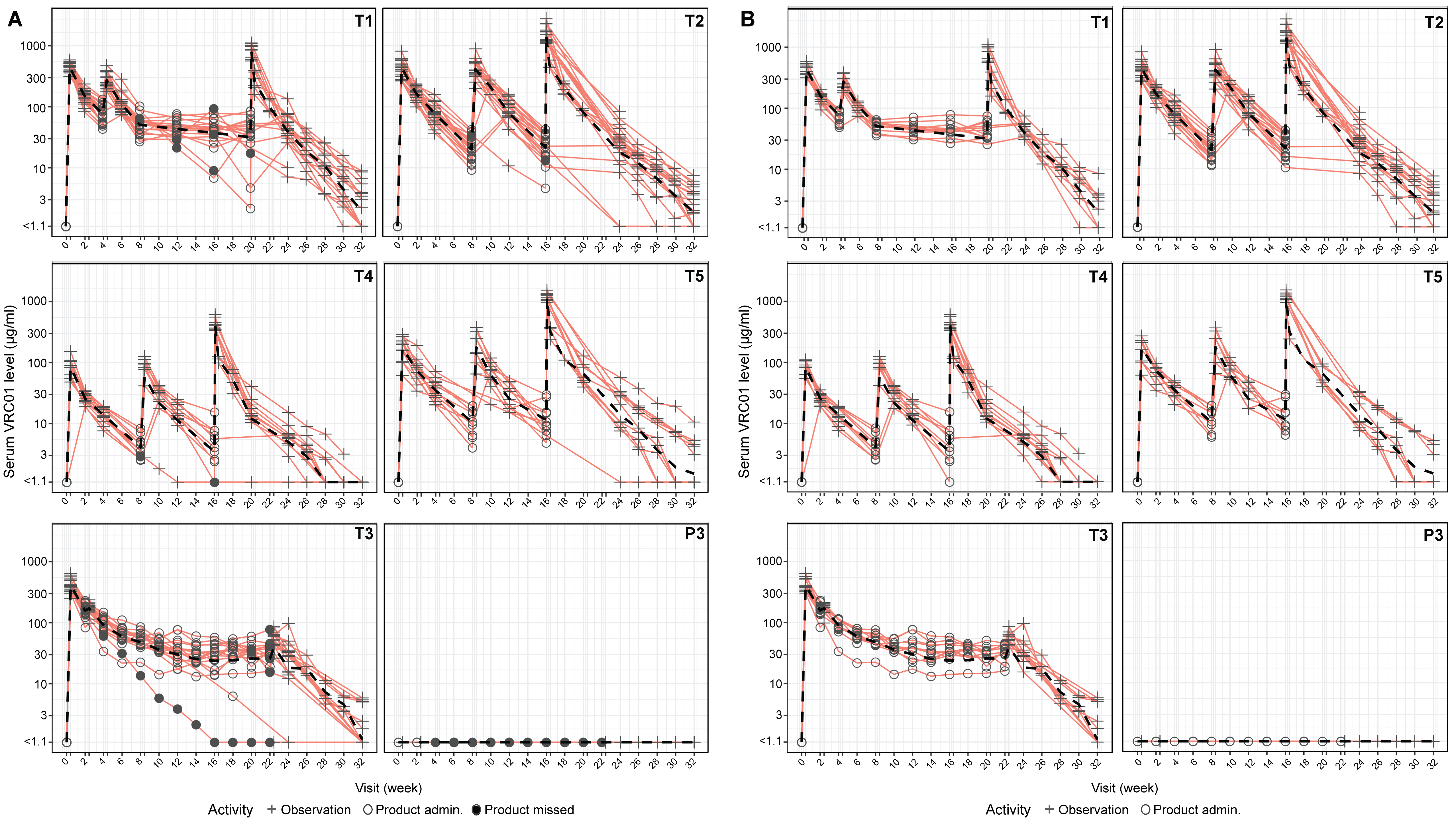

Supplement: S1 Fig — Data are presented for (A) the MITT cohort and (B) the PP cohort. A double tick on the x-axis denotes a time interval of 3 days, representing visit days 3, 17, 31, 59, 115, and/or 157. T1: 20 mg/kg IV q 4 weeks with 40 mg/kg IV loading; T2: 40 mg/kg IV q 8 weeks; T3/ (P3): 5 mg/kg SC q 2 weeks with 40 mg/kg IV loading; T4: 10 mg/kg IV q 8 weeks; T5: 30 mg/kg IV q 8 weeks. MITT, modified intent-to-treat; PP, per-protocol; P3, placebo group 3; q, quodque; SC, subcutaneous; T1, treatment group 1; T2, treatment group 2; T3, treatment group 3; T4, treatment group 4; T5, treatment group 5. (TIF) [file pmed.1002435.s003.tif]

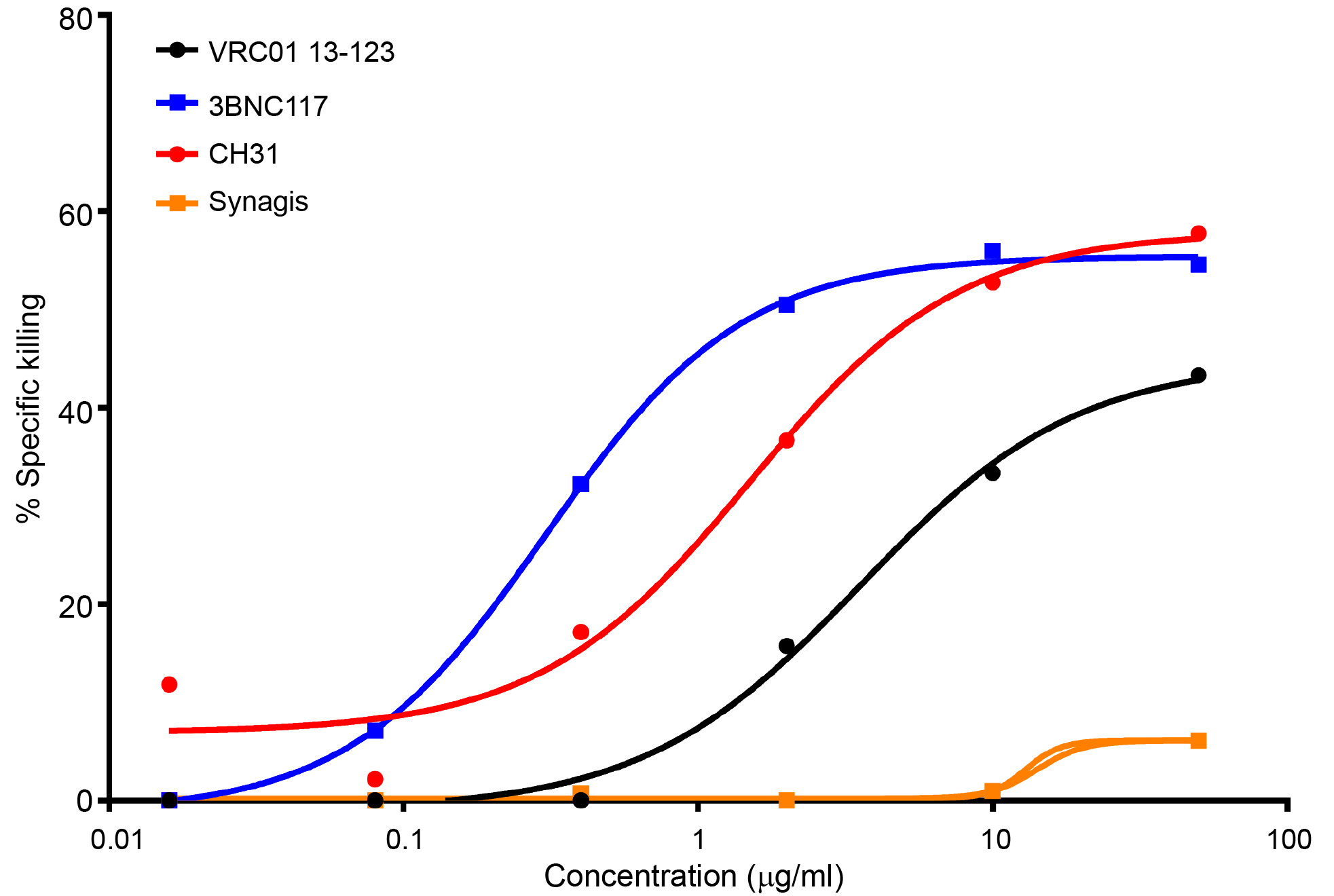

Supplement: S2 Fig — Shown is the percentage of specific killing using the Luciferase-HIV CH0505.LucR T2A.ecto/293T/17 assay. ADCC, antibody-dependent cellular cytotoxicity; bnAb, broadly neutralizing antibody; CD4bs, CD4 binding site. (TIF) [file pmed.1002435.s004.tif]
