# Supplementary material for: Safety, pharmacokinetics, and immunological activities of multiple intravenous or subcutaneous doses of an anti-HIV monoclonal antibody, VRC01, administered to HIV-uninfected adults: Results of a phase 1 randomized trial
Source: PLoS Med. 2017 Nov 14;14(11):e1002435. doi: 10.1371/journal.pmed.1002435 (PMC5685476; doi:10.1371/journal.pmed.1002435)
Supplement: S2 Table — T1: 20 mg/kg IV q 4 weeks with 40 mg/kg IV loading; T2: 40 mg/kg IV q 8 weeks; T3 (P3): 5 mg/kg SC q 2 weeks with 40 mg/kg IV loading; T4: 10 mg/kg IV q 8 weeks; T5: 30 mg/kg IV q 8 weeks. PP, per-protocol; P3, placebo group 3; q, quodque; SC, subcutaneous; T1, treatment group 1; T2, treatment group 2; T3, treatment group 3; T4, treatment group 4; T5, treatment group 5. (DOCX) [file pmed.1002435.s006.docx]

| **Treatment Group** | **Visit Number** | **Visit Day** | **Activity** | **N** | **N > 1.1 µg/ml** | **Geometric Mean** | **95%Confidence Interval** | **Median** | **Interquartile Range** | **Overall**  **Range** |
| --- | --- | --- | --- | --- | --- | --- | --- | --- | --- | --- |
| P3 | 2 | 0 | 1st infusion at 40 mg/kg | 2 | 0 | <1.1 |  | <1.1 |  |  |
|  | 3 | 3 | observation | 2 | 0 | <1.1 |  | <1.1 |  |  |
|  | 4 | 14 | 1st injection at 5 mg/kg | 2 | 0 | <1.1 |  | <1.1 |  |  |
|  | 5 | 17 | observation | 2 | 0 | <1.1 |  | <1.1 |  |  |
|  | 6 | 28 | 2nd injection at 5 mg/kg | 2 | 0 | <1.1 |  | <1.1 |  |  |
|  | 8 | 42 | 3rd injection at 5 mg/kg | 2 | 0 | <1.1 |  | <1.1 |  |  |
|  | 9 | 56 | 4th injection at 5 mg/kg | 2 | 0 | <1.1 |  | <1.1 |  |  |
|  | 11 | 70 | 5th injection at 5 mg/kg | 2 | 0 | <1.1 |  | <1.1 |  |  |
|  | 12 | 84 | 6th injection at 5 mg/kg | 2 | 0 | <1.1 |  | <1.1 |  |  |
|  | 13 | 98 | 7th injection at 5 mg/kg | 2 | 0 | <1.1 |  | <1.1 |  |  |
|  | 14 | 112 | 8th injection at 5 mg/kg | 2 | 0 | <1.1 |  | <1.1 |  |  |
|  | 15 | 126 | 9th injection at 5 mg/kg | 2 | 0 | <1.1 |  | <1.1 |  |  |
|  | 16 | 140 | 10th injection at 5 mg/kg | 2 | 0 | <1.1 |  | <1.1 |  |  |
|  | 17 | 154 | 11th injection at 5 mg/kg | 2 | 0 | <1.1 |  | <1.1 |  |  |
|  | 18 | 157 | observation | 2 | 0 | <1.1 |  | <1.1 |  |  |
|  | 19 | 168 | observation | 2 | 0 | <1.1 |  | <1.1 |  |  |
|  | 20 | 182 | observation | 2 | 0 | <1.1 |  | <1.1 |  |  |
|  | 21 | 196 | observation | 2 | 0 | <1.1 |  | <1.1 |  |  |
|  | 22 | 210 | observation | 2 | 0 | <1.1 |  | <1.1 |  |  |
|  | 23 | 224 | observation | 2 | 0 | <1.1 |  | <1.1 |  |  |
| T1 | 2 | 0 | 1st infusion at 40 mg/kg | 11 | 0 | <1.1 |  | <1.1 |  |  |
|  | 3 | 3 | observation | 11 | 11 | 421.8 | (366, 486) | 401.9 | (358.5, 510.2) | (314, 585.8) |
|  | 4 | 14 | observation | 11 | 11 | 146.6 | (127.1, 169.1) | 155.4 | (129, 168) | (93.6, 201) |
|  | 6 | 28 | 2nd infusion at 20 mg/kg | 11 | 11 | 68.5 | (58.5, 80.3) | 70.2 | (53.6, 88.3) | (48.7, 93.4) |
|  | 7 | 31 | observation | 10 | 10 | 260.4 | (216.4, 313.4) | 250.1 | (218.8, 310) | (179, 381.2) |
|  | 8 | 42 | observation | 10 | 10 | 98.7 | (84.9, 114.6) | 98.3 | (83.9, 122) | (73.4, 131) |
|  | 9 | 56 | 3rd infusion at 20 mg/kg | 11 | 11 | 50.7 | (44.7, 57.6) | 51.3 | (43.3, 58.4) | (35.7, 64.4) |
|  | 12 | 84 | 4th infusion at 20 mg/kg | 11 | 11 | 46 | (39.3, 53.8) | 46.1 | (40.1, 51.3) | (30.9, 70.8) |
|  | 14 | 112 | 5th infusion at 20 mg/kg | 11 | 11 | 45.1 | (37, 54.9) | 45.2 | (39.8, 53.9) | (26.8, 78.1) |
|  | 16 | 140 | 6th infusion at 20 mg/kg | 11 | 11 | 43.3 | (34.6, 54.1) | 43.8 | (32.8, 59) | (25.7, 74.6) |
|  | 16 | 140.5 | 1 hour post 6th infusion | 7 | 7 | 795.5 | (614.6, 1029.6) | 848 | (591.3, 1018.5) | (563, 1113) |
|  | 16.5 | 143^A^ | observation | 4 | 4 | 246.7 | (132.1, 461) | 248.1 | (183.3, 339.4) | (158, 391.2) |
|  | 17 | 154^A^ | observation | 4 | 4 | 95.3 | (64.6, 140.6) | 85.4 | (83.4, 111.9) | (82.4, 137.3) |
|  | 19 | 168 | observation | 10 | 10 | 46.2 | (38, 56.2) | 47 | (36.4, 55.4) | (30.7, 74.5) |
|  | 20 | 182 | observation | 5 | 5 | 20.6 | (12.1, 35) | 19.2 | (16.4, 25.8) | (12.2, 37.2) |
|  | 21 | 196 | observation | 7 | 7 | 12.2 | (8.5, 17.6) | 11 | (9.6, 15.7) | (7.5, 25.7) |
|  | 22 | 210 | observation | 8 | 7 | 4.7 | (2.1, 10.2) | 6.3 | (3.8, 8.2) | (<1.1, 10.6) |
|  | 23 | 224 | observation | 9 | 6 | 2 | (<1.1, 4.4) | 3 | (<1.1, 3.8) | (<1.1, 8.5) |
| T2 | 2 | 0 | 1st infusion at 40 mg/kg | 17 | 0 | <1.1 |  | <1.1 |  |  |
|  | 3 | 3 | observation | 17 | 17 | 441.2 | (379.1, 513.6) | 445.3 | (382.4, 503.4) | (267.2, 814.2) |
|  | 4 | 14 | observation | 17 | 17 | 167.5 | (148.7, 188.7) | 169.3 | (155.1, 182.2) | (99.4, 236.6) |
|  | 6 | 28 | observation | 17 | 17 | 81.4 | (67.2, 98.5) | 78 | (67.7, 106.3) | (36.8, 159.1) |
|  | 9 | 56 | 2nd infusion at 40 mg/kg | 17 | 17 | 20 | (16.3, 24.6) | 19.2 | (15.1, 25.1) | (11.1, 42.3) |
|  | 10 | 59 | observation | 14 | 14 | 413.5 | (331.1, 516.4) | 396.3 | (330.7, 527.6) | (199.3, 889.4) |
|  | 11 | 70 | observation | 3 | 3 | 217.2 | (107.6, 438.2) | 235.4 | (158.6, 274.3) | (158.6, 274.3) |
|  | 12 | 84 | observation | 16 | 16 | 92.3 | (75.9, 112.1) | 81.4 | (72.8, 127.4) | (42.7, 160.2) |
|  | 14 | 112 | 3rd infusion at 40 mg/kg | 17 | 17 | 24.2 | (19.4, 30.2) | 27.4 | (16.1, 34.8) | (10.4, 42.2) |
|  | 14 | 112.5 | 1 hour post 3rd infusion | 14 | 14 | 1548.9 | (1305.5, 1837.6) | 1398.3 | (1231.2, 1805.8) | (1123.5, 2815.1) |
|  | 14.5 | 115^A^ | observation | 3 | 3 | 485.8 | (328.7, 718.1) | 445.8 | (441.6, 582.6) | (441.6, 582.6) |
|  | 15 | 126^A^ | observation | 6 | 6 | 204.4 | (163.8, 255.1) | 209.6 | (163, 242.3) | (160.3, 264.5) |
|  | 16 | 140^A^ | observation | 3 | 3 | 83.7 | (57.9, 120.9) | 87.4 | (71, 94.5) | (71, 94.5) |
|  | 19 | 168 | observation | 17 | 17 | 26.9 | (19.7, 36.6) | 27.5 | (21, 35) | (8.3, 83.1) |
|  | 20 | 182 | observation | 13 | 13 | 11.8 | (8.7, 16.2) | 14.2 | (9.9, 16.4) | (3.6, 24.3) |
|  | 21 | 196 | observation | 13 | 12 | 6.6 | (3.9, 11.4) | 8.7 | (5.8, 10.7) | (<1.1, 18) |
|  | 22 | 210 | observation | 12 | 11 | 3.9 | (2.4, 6.5) | 5.2 | (3.3, 6.4) | (<1.1, 9.7) |
|  | 23 | 224 | observation | 15 | 12 | 2.4 | (1.5, 3.9) | 3 | (1.8, 4.9) | (<1.1, 7.5) |
| T3 | 2 | 0 | 1st infusion at 40 mg/kg | 11 | 0 | <1.1 |  | <1.1 |  |  |
|  | 3 | 3 | observation | 11 | 11 | 416.3 | (352.5, 491.7) | 391.8 | (330.8, 521.7) | (303.6, 637.8) |
|  | 4 | 14 | 1st injection at 5 mg/kg | 11 | 11 | 160.3 | (133.8, 192.1) | 164.6 | (143.1, 187.8) | (83.8, 229.2) |
|  | 5 | 17 | observation | 10 | 10 | 169.9 | (144.6, 199.7) | 181.3 | (154.3, 195.1) | (98.2, 212) |
|  | 6 | 28 | 2nd injection at 5 mg/kg | 11 | 11 | 84.6 | (66.8, 107) | 86.4 | (77.4, 113.5) | (33.8, 115.1) |
|  | 8 | 42 | 3rd injection at 5 mg/kg | 11 | 11 | 57.4 | (44.9, 73.3) | 61.4 | (53.9, 73.7) | (21.8, 80.3) |
|  | 9 | 56 | 4th injection at 5 mg/kg | 11 | 11 | 49.3 | (39.5, 61.6) | 46 | (43.8, 66.6) | (22.5, 75.6) |
|  | 11 | 70 | 5th injection at 5 mg/kg | 11 | 11 | 37.4 | (29.2, 47.9) | 39.5 | (33.2, 47) | (14.1, 55.2) |
|  | 12 | 84 | 6th injection at 5 mg/kg | 11 | 11 | 38.4 | (28, 52.6) | 37.8 | (25.2, 51.6) | (17.4, 77) |
|  | 13 | 98 | 7th injection at 5 mg/kg | 11 | 11 | 31.8 | (24, 42.1) | 32.6 | (24, 42.9) | (13.2, 60.4) |
|  | 14 | 112 | 8th injection at 5 mg/kg | 11 | 11 | 34.5 | (26.6, 44.7) | 33.8 | (29.1, 45.1) | (14.1, 57.6) |
|  | 15 | 126 | 9th injection at 5 mg/kg | 11 | 11 | 33.1 | (26.6, 41.3) | 37.6 | (27.8, 41.1) | (14.6, 45.1) |
|  | 16 | 140 | 10th injection at 5 mg/kg | 11 | 11 | 29.6 | (23.5, 37.2) | 31.8 | (26, 35.1) | (14.8, 51) |
|  | 17 | 154 | 11th injection at 5 mg/kg | 11 | 11 | 32.9 | (26.1, 41.4) | 36.1 | (30.1, 43.2) | (16.1, 45.9) |
|  | 18 | 157 | observation | 10 | 10 | 60 | (50.4, 71.6) | 61 | (50.5, 70.1) | (42.7, 86.2) |
|  | 19 | 168 | observation | 11 | 11 | 33.7 | (24.6, 46) | 32.5 | (28, 43) | (15.5, 96.7) |
|  | 20 | 182 | observation | 5 | 5 | 17.5 | (11.9, 25.9) | 14.8 | (14.6, 19) | (13.8, 29.3) |
|  | 21 | 196 | observation | 5 | 5 | 7.2 | (4.5, 11.5) | 6.5 | (5.7, 10.1) | (4.7, 11.3) |
|  | 22 | 210 | observation | 5 | 5 | 4.6 | (3.2, 6.4) | 4.2 | (3.6, 5.9) | (3.5, 6.3) |
|  | 23 | 224 | observation | 10 | 6 | 1.8 | (<1.1, 3.9) | 2.1 | (<1.1, 5.1) | (<1.1, 5.8) |
| T4 | 2 | 0 | 1st infusion at 10 mg/kg | 10 | 0 | <1.1 |  | <1.1 |  |  |
|  | 3 | 3 | observation | 9 | 9 | 84.4 | (70.3, 101.4) | 83.8 | (82.7, 104.8) | (54.4, 109.8) |
|  | 4 | 14 | observation | 10 | 10 | 25.2 | (21.7, 29.3) | 24.6 | (21.8, 29.6) | (18.8, 35.3) |
|  | 6 | 28 | observation | 10 | 10 | 13.4 | (10.6, 16.9) | 13.4 | (10.7, 18.4) | (7.6, 19.2) |
|  | 9 | 56 | 2nd infusion at 10 mg/kg | 10 | 10 | 4.2 | (3.1, 5.6) | 4 | (3.1, 5.2) | (2.5, 8.4) |
|  | 10 | 59 | observation | 8 | 8 | 87.1 | (65.8, 115.4) | 95.6 | (80.1, 104.9) | (41.5, 124.9) |
|  | 11 | 70 | observation | 8 | 8 | 29.5 | (23.1, 37.8) | 31.5 | (24, 37.3) | (17.6, 41.8) |
|  | 12 | 84 | observation | 10 | 10 | 15.3 | (12.2, 19.1) | 14.1 | (12.1, 20.6) | (9.4, 24.6) |
|  | 14 | 112 | 3rd infusion at 10 mg/kg | 10 | 9 | 4.2 | (2.2, 8) | 4.9 | (2.6, 7.7) | (<1.1, 15.5) |
|  | 14 | 112.5 | 1 hour post 3rd infusion | 9 | 9 | 419.5 | (356.3, 493.8) | 408.6 | (355.2, 451.3) | (332.4, 613) |
|  | 14.5 | 115^A^ | observation | 3 | 3 | 112.7 | (82.6, 153.7) | 114.7 | (98.7, 126.4) | (98.7, 126.4) |
|  | 15 | 126^A^ | observation | 4 | 4 | 52.4 | (27.9, 98.5) | 56.8 | (39.3, 71.5) | (31.5, 76.6) |
|  | 16 | 140^A^ | observation | 9 | 9 | 17 | (12.2, 23.6) | 14.8 | (12.9, 20.9) | (10.5, 40.8) |
|  | 19 | 168 | observation | 10 | 10 | 6.2 | (4.5, 8.5) | 6.1 | (4.8, 6.9) | (2.9, 15.3) |
|  | 20 | 182 | observation | 9 | 8 | 3.5 | (1.9, 6.3) | 3.7 | (3.3, 4.6) | (<1.1, 9) |
|  | 21 | 196 | observation | 8 | 3 | 1.1 | (<1.1, 2.6) | <1.1 | (<1.1, 2.7) | (<1.1, 6.7) |
|  | 22 | 210 | observation | 8 | 1 | <1.1 | (<1.1, 1.1) | <1.1 | (<1.1, 0.6) | (<1.1, 3.2) |
|  | 23 | 224 | observation | 9 | 0 | <1.1 |  | <1.1 |  |  |
| T5 | 2 | 0 | 1st infusion at 30 mg/kg | 8 | 0 | <1.1 |  | <1.1 |  |  |
|  | 3 | 3 | observation | 8 | 8 | 153.2 | (99.5, 236) | 185 | (103.1, 226.1) | (61.6, 270.3) |
|  | 4 | 14 | observation | 8 | 8 | 62.9 | (47.1, 83.8) | 68.5 | (50.5, 80.3) | (34.1, 95) |
|  | 6 | 28 | observation | 8 | 8 | 34.2 | (26.5, 44.1) | 34.9 | (27.8, 44.3) | (20.4, 49.6) |
|  | 9 | 56 | 2nd infusion at 30 mg/kg | 8 | 8 | 11.7 | (8, 17.3) | 12.9 | (8.1, 17.6) | (6, 19.6) |
|  | 10 | 59 | observation | 7 | 7 | 182.8 | (100.1, 334) | 245.8 | (99, 326.1) | (65.1, 377.9) |
|  | 11 | 70 | observation | 8 | 8 | 75.3 | (59.1, 96) | 76.3 | (57.3, 93.5) | (52.3, 118.9) |
|  | 12 | 84 | observation | 7 | 7 | 30.2 | (20.3, 44.9) | 33.7 | (17.6, 46.3) | (17.6, 49.3) |
|  | 14 | 112 | 3rd infusion at 30 mg/kg | 8 | 8 | 12.9 | (8, 20.8) | 12.2 | (8.3, 21.4) | (6.5, 29.7) |
|  | 14 | 112.5 | 1 hour post 3rd infusion | 8 | 8 | 1176.6 | (1032.9, 1340.2) | 1139.8 | (1056.7, 1315.9) | (954.8, 1535.3) |
|  | 14.5 | 115^A^ | observation | 1 | 1 | 241.5 |  | 241.5 |  |  |
|  | 16 | 140^A^ | observation | 5 | 5 | 59.8 | (37.5, 95.3) | 51.3 | (47.3, 84.2) | (39.8, 93.8) |
|  | 19 | 168 | observation | 8 | 8 | 16 | (9.6, 26.8) | 15.7 | (9.5, 29.6) | (7.6, 33.4) |
|  | 20 | 182 | observation | 8 | 8 | 8.6 | (4.9, 15.1) | 7.8 | (4.8, 17.5) | (3.8, 19.5) |
|  | 21 | 196 | observation | 8 | 6 | 3.7 | (1.3, 10.8) | 5.1 | (1.9, 10.9) | (<1.1, 12.2) |
|  | 22 | 210 | observation | 8 | 3 | 1.5 | (<1.1, 4.5) | <1.1 | (<1.1, 7.3) | (<1.1, 7.5) |
|  | 23 | 224 | observation | 8 | 3 | 1.2 | (<1.1, 2.9) | <1.1 | (<1.1, 3.8) | (<1.1, 5.3) |

**^A^** Visits at day 143 and 154 for T1 participants and day 115, 126, and 140 for T2, T4, and T5 participants occurred only for individuals who agreed to provide mucosal specimens, and if mucosal specimens were not collected at earlier visits
