# Supplementary material for: Safety, pharmacokinetics, and immunological activities of multiple intravenous or subcutaneous doses of an anti-HIV monoclonal antibody, VRC01, administered to HIV-uninfected adults: Results of a phase 1 randomized trial
Source: PLoS Med. 2017 Nov 14;14(11):e1002435. doi: 10.1371/journal.pmed.1002435 (PMC5685476; doi:10.1371/journal.pmed.1002435)
Supplement: S3 Table — T4: 10 mg/kg IV q 8 weeks; T5: 30 mg/kg IV q 8 weeks. AUC, area under the curve; PP, per-protocol; q, quodque; T4, treatment group 4; T5, treatment group 5. (DOCX) [file pmed.1002435.s007.docx]

| **Treatment Group** | **Measure** | **N 2^nd^/1^st^** | **Median of 2^nd^/1^st^** | **Mean +/- SD of 2^nd^/1^st^** | **N 3^rd^/2^nd^** | **Median of 3^rd^/2^nd^** | **Mean +/- SD of 3^rd^/2^nd^** | **N 3^rd^/1^st^** | **Median of 3^rd^/1^st^** | **Mean +/- SD of 3^rd^/1^st^** |
| --- | --- | --- | --- | --- | --- | --- | --- | --- | --- | --- |
| T4 | Trough (Observed) | 10 | 1.122 | 1.113 +/- 0.450 | 10 | 1.468 | 1.746 +/- 1.307 | 10 | 1.474 | 1.518 +/- 0.381 |
|  | AUC | 6 | 1.209 | 1.170 +/- 0.192 | 1 |  |  | 2 |  |  |
|  | Trough (Predicted) | 10 | 1.077 | 1.050 +/- 0.385 | 10 | 1.468 | 2.815 +/- 4.421 | 10 | 1.504 | 1.699 +/- 0.553 |
| T5 | Trough (Observed) | 8 | 1.305 | 1.218 +/- 0.539 | 8 | 1.199 | 1.335 +/- 0.556 | 8 | 1.528 | 1.434 +/- 0.456 |
|  | AUC | 6 | 1.168 | 1.129 +/- 0.180 | 0 |  |  | 0 |  |  |
|  | Trough (Predicted) | 8 | 1.374 | 1.241 +/- 0.488 | 8 | 1.289 | 1.370 +/- 0.347 | 8 | 1.750 | 1.587 +/- 0.41 |
