# Supplementary material for: Safety, pharmacokinetics, and immunological activities of multiple intravenous or subcutaneous doses of an anti-HIV monoclonal antibody, VRC01, administered to HIV-uninfected adults: Results of a phase 1 randomized trial
Source: PLoS Med. 2017 Nov 14;14(11):e1002435. doi: 10.1371/journal.pmed.1002435 (PMC5685476; doi:10.1371/journal.pmed.1002435)
Supplement: S1 Data — Participant characteristics and study/treatment adherence: participant_details.csv (masked participant details including demographics, treatment group, and reasons for treatment discontinuation and study termination); treatment.csv (received treatment details by SPA visit). Safety: reactogenicity_listing.csv (all local and systemic reactogenicity events); reactogenicity_summary.csv (reactogenicity events summarized at the maximum reportable severity grade by participant and visit number); ae_listing.csv (all nonreactogenicity AEs); ae_summary.csv (AEs summarized at the maximum severity and relatedness by participant, MedDRA Preferred Term, and System Organ Class); safety_laboratory_graded.csv (safety laboratory results and corresponding grades are listed by participant and visit collected [prior to study product administration]). Drug level and immune responses (functional activities): ada_assay.csv (processed ADA assay data, as described in the methods section, for ADA activity [end point variable name: result]); aly_assay.csv (processed ELISA assay data, as described in the methods section, for ELISA-based VRC01 serum-concentration [end point variable name: result]); nab_assay.csv (processed nAb assay data, as described in the methods section, for TZM-bl neutralization assay-based VRC01 serum concentration including both ID50 and ID80 values [end point variable names: concentration50 and concentration 80]); phagocytosis_assay.csv (processed ADCP assay data, as described in the methods section, for ADCP functional activity [end point variable names: avg_phagocytosis_score and response]); virus_capture_assay.csv (processed IVCA data, as described in the methods section, for infectious virion capture functional activity [end point variable names: avg_capture_percentage and response]); adcc_assay.csv (processed ADCC assay data, as described in the methods section, for ADCC functional activity [end point variable names: blsub_pct_specific_killing and response]). ADA, a [file pmed.1002435.s010.zip › data/S1 Data_Caption.docx]

**S1 Data. De-identified data underlying the reported findings in HVTN 104.**

1. Participant characteristics and study/treatment adherence

- participant_details.csv: masked participant details including demographics, treatment group, and reasons for treatment discontinuation and study termination.
- treatment.csv : received treatment details by study product administration (SPA) visit.

1. Safety

- reactogenicity_listing.csv: all local and systemic reactogenicity events.
- reactogenicity_summary.csv: reactogenicity events summarized at the maximum reportable severity grade by participant and visit number
- ae_listing.csv: all non-reactogenicity adverse events.
- ae_summary.csv: adverse events summarized at the maximum severity and relatedness by participant, MedDRA Preferred Term, and System Organ Class.
- safety_laboratory_graded.csv: safety laboratory results and corresponding grades are listed by participant and visit collected (prior to study product administration).

1. Drug level and immune responses (functional activities)

- ada_assay.csv: processed ADA assay data, as described in the methods section, for anti-drug antibody activity (endpoint variable name: *result*)
- aly_assay.csv: processed ELISA assay data, as described in the methods section, for ELISA-based VRC01 serum-concentration (endpoint variable name: *result*).
- nab_assay.csv: processed nAb assay data, as described in the methods section, for TZM-bl neutralization assay-based VRC01 serum concentration including both ID50 and ID80 values (endpoint variable names: *concentration50* and *concentration 80*).
- phagocytosis_assay.csv: processed ADCP assay data, as described in the methods section, for antibody-dependent cellular phagocytosis functional activity (endpoint variable names: *avg_phagocytosis_score* and *response*).
- virus_capture_assay.csv: processed IVCA assay data, as described in the methods section, for infectious virion capture functional activity (endpoint variable names: *avg_capture_percentage* and *response*).
- adcc_assay.csv: processed ADCC assay data, as described in the methods section, for antibody-dependent cellular cytotoxicity functional activity (endpoint variable names: *blsub_pct_specific_killing* and *response*).
